# Warm afterglow from the Toarcian Oceanic Anoxic Event drives the success of deep-adapted brachiopods

**DOI:** 10.1038/s41598-020-63487-6

**Published:** 2020-04-16

**Authors:** C. V. Ullmann, R. Boyle, L. V. Duarte, S. P. Hesselbo, S. A. Kasemann, T. Klein, T. M. Lenton, V. Piazza, M. Aberhan

**Affiliations:** 1 University of Exeter, Camborne School of Mines & Environment and Sustainability Institute, Penryn Campus, Treliever Road, Penryn, TR10 9FE United Kingdom; 2 University of Oxford, Department of Earth Sciences, South Parks Road, Oxford, OX1 3AN United Kingdom; 3 University of Exeter, Global Systems Institute, College of Life and Environmental Sciences, Exeter, EX4 4QE United Kingdom; 4 University of Coimbra, MARE, 3030-790 Coimbra, Portugal; 5 University of Bremen, Faculty of Geosciences and MARUM-Center for Marine Environmental Sciences, 28359 Bremen, Germany; 6 Museum für Naturkunde, Leibniz Institute for Evolution and Biodiversity Science, 10115 Berlin, Germany

**Keywords:** Carbon cycle, Palaeoceanography, Palaeoclimate

## Abstract

Many aspects of the supposed hyperthermal Toarcian Oceanic Anoxic Event (T-OAE, Early Jurassic, c. 182 Ma) are well understood but a lack of robust palaeotemperature data severely limits reconstruction of the processes that drove the T-OAE and associated environmental and biotic changes. New oxygen isotope data from calcite shells of the benthic fauna suggest that bottom water temperatures in the western Tethys were elevated by c. 3.5 °C through the entire T-OAE. Modelling supports the idea that widespread marine anoxia was induced by a greenhouse-driven weathering pulse, and is compatible with the OAE duration being extended by limitation of the global silicate weathering flux. In the western Tethys Ocean, the later part of the T-OAE is characterized by abundant occurrences of the brachiopod *Soaresirhynchia*, which exhibits characteristics of slow-growing, deep sea brachiopods. The unlikely success of *Soaresirhynchia* in a hyperthermal event is attributed here to low metabolic rate, which put it at an advantage over other species from shallow epicontinental environments with higher metabolic demand.

## Introduction

The Early Jurassic Toarcian Stage is characterized by possibly the largest global carbon cycle perturbation since the beginning of the Mesozoic (c. 250 Ma)^1,2^. A series of such perturbations, evidenced by large-scale carbon isotope excursions (CIE’s), through the late Pliensbachian to middle Toarcian stages (c. 184–180 Ma), are recorded globally by sedimentary strata. Organic matter and carbonate in bulk sediments^2–4^, as well as fossil wood^1^ and macrofossils^5,6^, show excursions in carbon isotope ratios which have not been matched in size since. This episode of environmental change was associated with widespread marine deposition of laminated, organic-rich mud in epicontinental seas, suggestive of at least regionally severe water de-oxygenation^7,8^, and has consequently become known as the Toarcian Oceanic Anoxic Event (T-OAE).

The evolution of environmental conditions across the T-OAE interval has been reconstructed in high temporal detail in previous studies. The earliest Toarcian is marked by the recovery from a negative CIE at the Pliensbachian-Toarcian boundary^5,9^ and linked to early magmatism of the Karoo-Ferrar large igneous province^10–13^. Within the T-OAE, regular, stepped changes in carbon isotope ratios resulted in a strong δ^13^C decrease, have been documented. These steps have been interpreted to signify repeated, astronomically paced, widespread release of methane from various sources^3,14–16^, probably also initially triggered by large scale volcanism of the Karoo-Ferrar Large Igneous Province. After the peak of the negative CIE, carbon isotope ratios rose strongly with δ^13^C values reaching up to around +6‰ in belemnite calcite in the UK sections^6,17^ and >+5‰ in brachiopod calcite in Portugal^5,18^. This positive shift of c. 5‰, in conjunction with coeval globally observed deposition of organic rich muds in marine^7^ and lacustrine strata^19^, can be taken as clear indication for large-scale organic matter burial preferentially removing ^12^C from the exogenic carbon cycle^7,13,14^.

Biotic consequences of the T-OAE were far-reaching reorganizations of marine and terrestrial flora and fauna, including severe extinctions and protracted recovery from the loss of biodiversity^20–24^. The magnitude and duration of the greenhouse warming that must have been associated with the large-scale release of methane and carbon dioxide into the atmosphere, however, has so far been hard to quantify reliably. The lack of robust palaeotemperature reconstructions is due to poor proxy calibrations from sedimentary successions of this age, as well as proxy resetting due to post-depositional processes. The best established proxy for Jurassic seawater temperatures, oxygen isotope ratios in marine biological carbonates, requires the continued presence of well-preserved fossils with known life habits in sedimentary archives, even within the strata representative of the most severe environmental upheaval. These prerequisites have proven challenging for reconstructing temperature change across the T-OAE. Most records rely on the calcite of belemnite rostra^6,25^ which are rare or absent from strata deposited during peak anoxic conditions, and for which complications arising from vital effects and changes in habitat depth cannot be discounted. Data on calcite secreting, sessile faunas of the seafloor on the other hand are sparse, particularly in the intervals of most extreme environmental conditions^5,18,26^.

**Figure 1:**
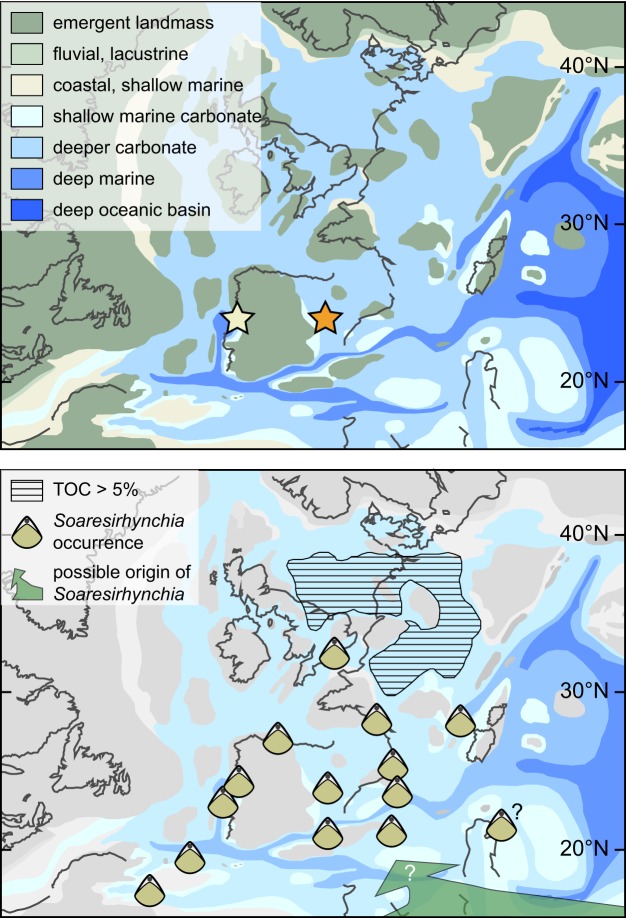
Upper panel: Toarcian palaeogeography after Ref. ^71^. with sampled stratigraphic successions marked with stars (yellow star, Fonte Coberta / Rabaçal; orange star: Barranco de la Cañada). Lower panel: Same palaeogeography overlain by early Toarcian occurrences of *Soaresirhynchia* taken from refs. ^22,60^, areal extent of organic rich mudstones with TOC > 5 wt %^72^ and putative deep water habitat and migration direction of *Soaresirhynchia*^62^.

In this contribution we make use of a new multiproxy dataset to provide a high-quality, temporally well-resolved record of oxygen and carbon isotope data through the early Toarcian. The aim is to use these data to provide guidance on the evolution of temperatures and carbon cycle and to assess first-order controls on Earth system responses. This large dataset comprises macrofossil ranges, shell structure, element/Ca, and C and O isotope ratios for multiple taxa of rhynchonellid brachiopods, the bivalve *Gryphaea*, belemnite calcite, diagenetic cements, and bulk rocks, of the Toarcian strata from the Barranco de la Cañada section in Spain (40°23′53.4″N, 1°30′07.4″W; Fig. 1, supplements). These 550 geochemical analyses are supplemented by equivalent results from 135 brachiopod, bivalve and bulk rock samples from a correlative succession of Toarcian strata at Fonte Coberta and Rabaçal, Portugal (Fonte Coberta: 40°03′36.5″N, 8°27′33.4″W; Rabaçal: 40°03′08.0″N, 8°27′30.5″W; Fig. 1). Taxonomic control as well as rigorous optical and chemical screening of the shells for diagenesis permit discussion of palaeotemperature evolution and its relation to the carbon cycle perturbation of the T-OAE with a high degree of quantitative detail. The biomineral structures and geochemical fingerprints of the brachiopods represented in the strata are also studied in detail. These data are used to establish novel proxies that help better understand the extraordinary success of the brachiopod genus *Soaresirhynchia* during times of severe environmental change.

## Results

### Carbon and oxygen isotope stratigraphy

**Figure 2:**
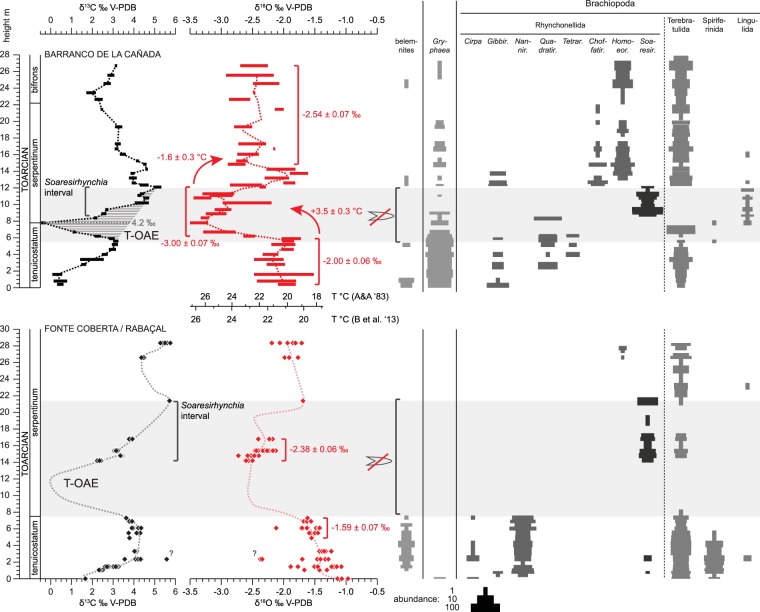
C and O isotope stratigraphy as well as species distribution data for Barranco de la Cañada and Fonte Coberta/Rabaçal. C and O isotope data for Barranco de la Cañada are shown as averages with error bars of 2 standard errors of the mean, and the dotted line for oxygen isotope ratios represents a three point running average. Absolute temperature changes are computed using the oxygen isotope thermometers of Brand *et al*.^39^ and Anderson and Arthur^69^ for comparison. For Fonte Coberta/Rabaçal, for which fewer data are available, all individual brachiopod measurements that passed screening for diagenesis are plotted. Dotted lines represent the schematic evolution of isotopic ratios expected for this section from observations in well studied sections in Peniche (Lusitanian Basin^1,5^) and analytical data from Barranco de la Cañada. The belemnite gap in Fonte Coberta/Rabaçal is based on observations and comparison to correlative strata in Peniche^1^. The duration of the T-OAE (light grey band) as indicated by the negative CIE and elevated temperatures coincides remarkably well with the extent of this belemnite gap.

Carbon and oxygen isotope ratios of well-preserved rhynchonellid brachiopods and the bivalve *Gryphaea* (see supplements for assessment of preservation and construction of combined isotope record) show distinct fluctuations through the Barranco de la Cañada section (Fig. 2).

In the lowest three Toarcian horizons, δ^13^C values of +0.3 to +0.5‰ are recorded, above which a rise to +3.1‰ occurs. Above 5.6 m, a strong decrease in δ^13^C values commences, which culminates in a value of −0.4‰ at 7.8 m height. Within sampling resolution, this position coincides with the boundary of the *tenuicostatum* and *serpentinum* ammonite zones. Above this minimum, δ^13^C values recover and reach very positive values averaging +5.1‰ at 12.1 m height. Upsection, a general decrease of δ^13^C to c. +2.5‰ with superimposed minor fluctuations is observed which reaches into the lower *bifrons* ammonite zone.

Over the same interval, δ^18^O values are initially stable, with averages from −2.2 to −1.9‰ up to 5.9 m. Here, a strong decrease occurs with average values of −3.4 to −2.3‰ recorded in the interval from 6.2 to 12.4 m, encompassing the entire negative carbon isotope excursion. After a stratigraphically narrow return to less negative δ^18^O values of −2.1 to −1.8‰ in the interval from 12.6 to 14.3 m, δ^18^O attains intermediate values of −2.8 to −2.1‰ for the remainder of the studied part of the succession.

Correlative strata from the Portuguese section at Fonte Coberta / Rabaçal show qualitatively the same trends, but rarity of benthic organisms in parts of the studied strata leads to a less detailed record (Fig. 2). The rise of δ^13^C values through the lowest Toarcian and the initiation of the subsequent negative CIE are well developed in the lowest 8 m, representing the *tenuicostatum* ammonite zone. Above an interval of c. 6 m barren in brachiopods, a prominent rise in δ^13^C values from +2.4 to +5.7‰ is observed which is followed upwards by comparatively positive δ^13^C values between +4.4 to +5.8‰.

Oxygen isotope ratios in the Tenuicostatum zone at Fonte Coberta / Rabaçal are comparatively positive averaging −1.59 ± 0.07‰ (2 standard errors of the mean = 2se, n = 19) in the interval from 4.9 to 7.3 m. Distinctly more negative values are recorded by brachiopods from the lower *serpentinum* ammonite zone, averaging −2.38 ± 0.06‰ (2 se, n = 32) in the interval from 14.2 to 16.8 m. The δ^18^O values higher up in the section are intermediate between these two extremes.

### Brachiopod ranges

In the studied interval, profound changes in the brachiopod assemblage are recorded in both successions which are consistent with previous reports^21,27^ (Fig. 2). The lowermost Toarcian strata in Barranco de la Cañada are characterized by the rhynchonellids *Gibbirhynchia* and *Quadratirhynchia* as well as sparse occurrences of *Tetrarhynchia*. Terebratulids are generally common and abundant in the uppermost part of the Tenuicostatum zone. This assemblage is abruptly replaced at the level of 8.7 m by the rhynchonellid *Soaresirhynchia* as the only brachiopod apart from rare occurrences of lingulids. The disappearance of *Soaresirhynchia* c. 3.5 m higher in the section is marked by the return of other rhynchonellids represented by *Gibbirhynchia*, *Choffatirhynchia* and *Homoeorhynchia* as well as terebratulids which are common in most collected beds of this interval. After initially abundant occurrence, the bivalve *Gryphaea* drops abruptly in abundance at 7 m, above which it occurs only sporadically and particularly seldom in the interval from 9.4 to 14.3 m. Belemnites are generally rare and entirely absent in the interval from 5.6 to 12.1 m.

In Fonte Coberta/Rabaçal the rhynchonellid genera *Cirpa* and *Nannirhynchia* are observed in the Tenuicostatum zone, together with common terebratulids, the last spiriferinids, and very rare finds of *Gibbirhynchia*, *Soaresirhynchia*, and lingulid brachiopods^27,28^ (Fig. 2). Above a brachiopod-barren interval between 7.9 and 14.2 m in this section, *Soaresishynchia* occurs together with sparse terebratulids and is followed by an impoverished fauna of terebratulids with a single find of a lingulid brachiopod and rare specimens of *Homoeorhynchia*.

### Brachiopod geochemistry and ultrastructure

Detailed characterisation of shell ultrastructure and investigation of a large number of brachiopod samples for their geochemical signatures allows establishment here of a series of novel proxies for palaeobiological function. These proxies help understanding brachiopod palaeoecology, especially in the context of major environmental change. Besides distinct differences in the morphology of shell fibres, we also employ an approximation of shell organic matter content, the intra-specimen isotopic variability, and element/Ca ratios, to inform about growth rate and metabolic rate.

**Figure 3:**
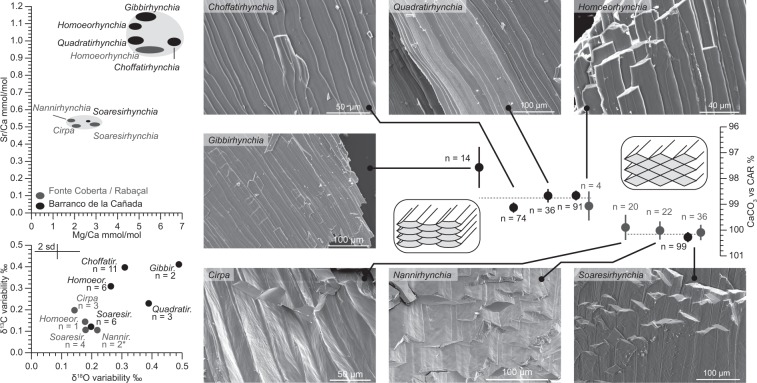
Ultrastructural and geochemical compositions of studied rhynchonellid brachiopods. Black symbols: Barranco de la Cañada, grey symbols: Fonte Coberta/Rabaçal. Top left: Average Mg/Ca and Sr/Ca data for studied taxa. The ellipses indicate 95% confidence intervals of the averages for each genus. Light grey areas delineate two clusters assigned to shallow water forms with higher Mg/Ca and Sr/Ca and deeper water forms with very low Sr/Ca and Mg/Ca ratios. Bottom left: Median 2 sd isotopic variability of individual brachiopods from different genera sampled from the two sections. Only specimens for which 4 or more analyses were available were used (*: One spurious datum excluded, supplements). Error bars indicate analytical uncertainty for single isotope measurements. Right hand side: Representative SEM images of different observed shell structures illustrating the diamond-shaped cross sections of *Cirpa*, *Nannirhynchia*, and *Soaresirhynchia*. Schematic morphologies of the two different groups are plotted next to measurements of their average computed CaCO_3_ concentration versus the in-house calcite standard CAR (Carrara Marble). Note the inverted axis to illustrate the lower non-carbonate fraction at high CO_2_ yields. The difference between the two groups is 1.4%. Error bars denote 2 standard errors of the mean (2 se).

Two distinct types of shell ultrastructure are observed for the secondary layer calcite of the studied rhynchonellid brachiopods (Fig. 3).

The fibres of *Soaresirhynchia*, *Cirpa* and *Nannirhynchia* have markedly diamond-shaped to rectangular cross sections with a distinct surface pattern of oblique, slightly curved bands that are laterally continuous across multiple shell fibres. The secondary layer calcite of other studied rhynchonellids in contrast comprises smooth fibres with typically flattened, hat-shaped cross section (Fig. 3).

The two brachiopod taxonomic clusters with shared ultrastructure characteristics are also distinct in their shell geochemical composition. The biomineral fibres of *Soaresirhynchia*, *Cirpa* and *Nannirhynchia* secondary shell layers have a c. 1.4% higher carbonate content than the other rhynchonellids (Fig. 3), suggesting that their shell calcite contains substantially less intracrystalline organic matter than that of *Gibbirhynchia*, *Homoeorhynchia*, *Quadratirhynchia* and *Choffatirhynchia*. Cation and anion impurities are also significantly lower in the former genera (Fig. 3), forming a cluster at 1.8 to 2.9 mmol/mol Mg/Ca and 0.51 to 0.54 mmol/mol Sr/Ca compared to 4.8 to 6.6 mmol/mol Mg/Ca and 0.95 to 1.14 mmol/mol Sr/Ca in the latter. Furthermore, *Soaresirhynchia* is the only studied brachiopod genus from the Barranco de la Cañada section whose Mg/Ca and Sr/Ca ratios are consistently lower than those of the bivalve *Gryphaea*. The difference is 0.3 ± 0.2 (2 se) mmol/mol for Mg/Ca and 0.21 ± 0.02 (2 se) mmol/mol for Sr/Ca.

## Early Jurassic environments constrained by brachiopods

Brachiopods were amongst the first animals to successfully adopt external hard parts during the biomineralisation revolution of the Proterozoic-Paleozoic transition around 540 million years ago. Today, they are found in the oceans across a wide range of latitudes and water depths, even though their relative contribution to the composition of marine faunal assemblages has declined since the Mesozoic^29^. Modern brachiopods are characterized by a remarkable diversity in biomineralisation strategies and multi-layered shell structures, which are formed by amorphous calcium carbonate, low magnesium calcite, high magnesium calcite and phosphate^30^. This wide range of structure and composition is an evolutionary asset acquired, broadened, and preserved by brachiopods over more than 500 million years during which they adapted to environmental change and climatic upheavals that at times, as during the T-OAE, were catastrophic for global faunal diversity.

### The early Toarcian carbon cycle perturbation

The most characteristic feature of the T-OAE carbon cycle perturbation is a pronounced negative CIE of several permil in magnitude commencing in the uppermost  *tenuicostatum* ammonite zone^1,3,4,7,13^ (Fig. 2). The exact nature of this excursion is still debated, as its magnitude has been hard to constrain robustly. Belemnite calcite records^6,25^ usually show hardly any negative CIE, which can at least conceptually be explained by faunal turnover and habitat shifts of these mobile, marine predators^6^. Bulk organic matter^3,4,7^ and wood^1,14,31^ records on the other hand commonly feature a very prominent negative CIE, reaching amplitudes as large as 8‰. This amplitude is thought to be inflated due to coincident changes in organic matter sources for bulk organic matter^2^ as well as physiological effects in terrestrial floras^1^. To account for these effects, an amplitude correction has been proposed that limits the size of the negative carbon isotope excursion to 3–4‰^2^. The newly generated macrofossil dataset does not suffer from the above problems as it is derived from benthic organisms that are known for their consistent biomineralisation behaviour devoid of vital effects^32,33^. The negative CIE observed in Barranco de la Cañada has an amplitude of 4.2‰ when compared against a hypothetical linear increase between the carbon isotope maxima bounding the CIE (Fig. 2). This magnitude may nevertheless be somewhat biased. Potentially, strata relating to the most negative carbon isotope ratios were either not deposited or did not yield calcite fossils. The δ^13^C of ambient dissolved inorganic carbon may have also been somewhat affected by potential changes in surface productivity^6^. Nevertheless, because all other means of reconstructing the magnitude of the negative CIE are affected by the same complications, the new magnitude estimate is thought to be the most reliable thus far.

A rough estimate of the mass of ^13^C-depleted carbon that must be introduced into the exchangeable ocean-atmosphere carbon pool in order to drive the observed negative CIE can be derived from simple mass balance equations (see supplement, ref. ^34^). Assuming an exchangeable carbon pool with an average δ^13^C value of +2.6‰ and a size two to four times as great as that of pre-industrial times^35^, the observed negative CIE would require the addition of c. 3,500 to 7,000 Gt of carbon derived from methane (supplements). However, this estimate needs to be taken as a minimum for the amount of carbon injected into the ocean-atmosphere system. More realistically, one should conceive of the carbon cycle perturbations as resulting from multiple carbon sources, rather than a discrete, singular perturbation represented by the above calculations^3,4,15,31,36^.

### Toarcian temperature evolution

The classification of the T-OAE as a major hyperthermal episode is not controversial (e.g. ref. ^37^), but the thermal evolution of the Earth surface system during the Toarcian is still poorly known. Large published belemnite datasets are likely affected by habitat and water mass changes^6,38^. Therefore, palaeotemperatures computed from the calcite of their fossil hard parts should not be taken as unequivocal reflection of sea surface water temperature. Data published on brachiopods^5,18,26^ on the other hand are hitherto sparse, lack resolution in the hyperthermal interval, and are partially based on terebratulids which are more prone than rhynchonellids to metabolically induced isotope disequilibrium^33^.

The newly gathered data from well-preserved fossils from the Barranco de la Cañada section now show that the beginning of the hyperthermal coincides precisely with the onset of the negative CIE (Fig. 2). The temperature increase is marked by a negative shift of 1.00 ± 0.09‰ in δ^18^O. Using the brachiopod oxygen isotope thermometer of Brand *et al*.^39^, this shift computes to a temperature increase of 3.5 ± 0.3 °C at the seafloor of the shallow epicontinental basin (Fig. 2; supplements), if confounding effects of sea-level change and salinity fluctuations can be discounted. This magnitude of temperature change is lower than previously inferred from brachiopod datasets from Portugal^5^ and Spain^26^, which can be explained by very different sample sizes. The earlier datasets are limited to a comparatively small number of analyses for which the inferred difference between coldest and warmest temperatures was taken to reflect T-OAE temperature change. Here, it is possible to quantify the increase of average temperatures from the earliest Toarcian into the T-OAE. This quantity is necessarily smaller than the difference between minimum pre-event and maximum event temperatures, but better reflects changes in the Earth surface system.

Besides temperature increase, more negative δ^18^O values could signify shallowing or partial freshening of the local seawater. Early Toarcian changes in sea-level have been recognized in Spanish^21^ and Portuguese basins^1,40,41^, generally pointing to an overall transgression and deepening. Inferring that sea-level is the primary driver of the observed oxygen isotope variability is therefore inconsistent with these reconstructions. Sea-level reconstructions neither suggest entirely parallel evolution in Portugal and Spain, nor a marked shallowing confined to the upper *tenuicostatum* and lower *serpentinum* zones. A gradual shallowing of a thermally stratified water body is also contradicted by the depositional environment of the studied sections^21,27^. Water mass stratification would likely have induced persistent bottom water anoxia for which there is no lithological or faunal indication in either of the studied successions.

Freshening has been proposed for UK and German basins^42,43^. However, the continuous presence of stenohaline organisms in both studied sections, particularly brachiopods^44^, makes such an interpretation unlikely. Salinity decrease in the European epicontinental sea has been interpreted to be much less noticeable in the Iberian region than the more northerly German and UK sites^42,43^. Substantial salinity fluctuations in the studied part of the western European epicontinental seas are also inconsistent with modelling results^35,45^ and thus appear much less plausible than a direct temperature forcing of the oxygen isotope signature.

Despite a gradual return to more positive δ^13^C values indicating the progressive drawdown of excess atmospheric greenhouse gases into sedimentary organic matter, temperatures remained stable and high. The first sign of a temperature reduction is only seen around the last occurrence of *Soaresirhynchia*, when δ^13^C values reached their global maximum. The temperature decrease thus lags several 100 kyr^3,46^ behind the minimum in δ^13^C values. A compatible pattern, albeit with less dense data coverage, is observed also in the Portuguese strata (Fig. 2). It therefore appears that at least regionally the T-OAE was associated with a climate shift into which the Earth system was locked.

### Model constraints on palaeoenvironmental change

The newly assembled high-fidelity record of palaeoenvironmental proxy data permits the extraction of further Earth system parameters from inversion models such as GEOCARB and GEOCARBSULF^47,48^ and forward modelling (COPSE, refs. ^49,50^, supplements). Particular interest is in carbon cycle dynamics, their relation to climate change, and global weathering feedbacks which are intimately linked with atmospheric carbon dioxide levels and temperature.

#### Inverse modelling

**Figure 4:**
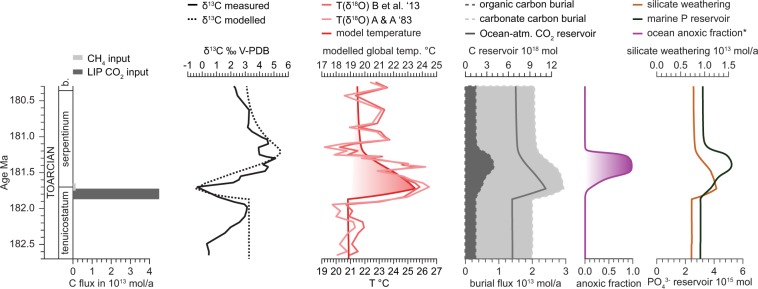
Modelling results for carbon cycle perturbation, temperature change, weathering and nutrient dynamics as well as intensity of ocean anoxia. Measured δ^13^C values are shown next to temperature reconstructions using temperature equations of Anderson and Arthur^69^ and Brand *et al*.^39^. The coarse features of the δ^13^C values are matched by a transient injection of thermogenic CO_2_ as well as CH_4_ in the COPSE forward biogeochemical model. *: The representation of the global ocean anoxic fraction depicted is defined as the fraction of the ocean surface area below which the oxygen saturation in the oxygen minimum zone would be below 10%^70^.

Weathering fluxes can be approximated from palaeotemperature and pCO_2_ when combined with Toarcian-specific estimates of various tectonic forcings^49,50^ (Fig. 4; supplements). The combination of these weathering estimates and the measured δ^13^C data can then be used to derive corresponding estimates of the organic and carbonate carbon burial fluxes as long as inorganic and organic carbon pools are in isotopic equilibrium. At steady state, the fraction of carbon that is buried in reduced form, (i.e., organic carbon) increases with increasing carbonate δ^13^C. Carbonate δ^13^C values greater than +3‰ through much of the *serpentinum* zone can be attributed to such an increased organic matter burial flux. However, given the large-scale perturbation associated with external introduction of ^13^C-depleted carbon during the T-OAE (e.g., Refs. ^3,7,14^), it is highly likely that the system deviates from steady state at least at the low point of the negative CIE, compromising the reliability of carbon flux estimates at this point.

In order to sustain high marine organic carbon burial, limiting nutrients supporting primary production have to be in high supply, and areas subject to ocean anoxia due to the increased oxygen consumption of organic carbon remineralization expanded^51^. Increased fluxes of elements to the ocean derived from continental weathering are well documented for the T-OAE^12,36,37,52^ even though timing and magnitude of these fluxes are not unequivocal. The ocean anoxic fraction and the quantities of limiting nutrients, which ultimately derive from such rock weathering, can be approximated using carbon burial flux estimates (supplements).

Important modulations of the Earth system’s response to climate perturbations arise on the basis of two distinct limiting factors on the silicate weathering flux^53,54^: kinetic factors (i.e., CO_2_ supply and temperature) versus substrate- related factors (i.e. supply of fresh rock through uplift). If silicate weathering is limited by CO_2_ then it is intimately connected with atmospheric carbon dioxide and thus planetary temperature via a homeostatic negative feedback^55^. By contrast, if supply of silicates is insufficient to counterbalance any increase in atmospheric CO_2_, this will prolong any associated hyperthermal greenhouse interval (and, in this case, any associated OAE). The nature of silicate weathering limitation has consequently been proposed as a key controlling factor on the duration of other hyperthermal intervals^56^.

#### Forward biogeochemical modelling

“Forward” biogeochemical models allow reproduction of the observed carbon cycle and climate perturbation of the T-OAE independent of the analysed data. We employ the COPSE (Carbon, Oxygen, Phosphorous, Sulphur, Evolution^49,50^) box model in order to mimic a carbon cycle perturbation driven by large igneous province (LIP)-derived CO_2_ and hydrate-derived CH_4_ (supplements; Fig. 4). Geochemical data extracted from fossils can be matched closely by this approach. Modelling these data provides further constraints on types, timing and magnitude of carbon injection and yields valuable information on the weathering feedback and nutrient supplies at the time^36,52,57^.

Within the COPSE model, the greenhouse perturbation leads to an increase in the input of phosphorous to the ocean via weathering, which boosts production and therefore the respiratory oxygen demand imposed by remineralization of sinking organic matter. This increases marine anoxia, leading to a reduction in marine phosphate burial which further amplifies organic carbon production, thus creating a short term positive feedback. Over longer timescales, the increase in production also leads to enhanced marine organic carbon burial. This increase in organic carbon burial ultimately causes a compensating increase in the global atmosphere-ocean oxygen reservoir, which may be of sufficient magnitude to create a long-term negative feedback that contributes to the system coming out of the OAE^58^. This sequence of events is reproduced by the model, and is sufficient to reproduce the coarse features of the δ^13^C curve when coupled to the carbon isotope equations (see supplement). However, the associated change in the global oxygen reservoir is modest, and CO_2_ consumption by weathering, relative to the magnitude of the initial greenhouse input, is the main controlling factor on the duration of the OAE.

The timing of anoxia lags behind that of the greenhouse gas input (Fig. 4), consistent with the lag between the lower limit of the δ^13^C curve and the maximum value of the marine organic carbon burial flux implied by the inversion model. As observed in the fossil δ^18^O data, the hyperthermal interval also lasts longer than the negative CIE, due to the time needed for weathering to consume the CO_2_ introduced via the perturbation. The duration of the OAE is ultimately dictated by the time it takes to consume the excess CO_2_ by silicate weathering, again raising the above issue of supply versus kinetic silicate weathering limitation. Figure 4 represents this using a formulation at which silicate weathering cannot exceed twice its current value (supplements).

The actual threshold at which silicate weathering becomes supply-limited is uncertain, and the formulation in the model relates the theoretical kinetically limited rate to a maximum weathering rate parameter. Some calculations place an upper limit on this maximum value of about 10 times the present rate, on the basis of crustal composition^56^, whereas lower estimates of c. 3 times the present flux have been derived within Precambrian modelling based on the maximum amount of CO_2_ consumable by rock flour^59^. Further empirical data may help determining such controls on the length of OAEs, including the Toarcian, with better reliability, and our model highlights the importance of such data.

### Environmental extremes and the faunal response of Soaresirhynchia

*Soaresirhynchia* is commonly associated with the later part of the T-OAE, and found in the lower *serpentinum* ammonite zone in western Tethyan sections where black shales are not extensively developed^21,22,60^. A singular *Soaresirhynchia* occurrence in the lowermost Toarcian of the Lusitanian Basin has previously been reported by Almeras^61^ which is here confirmed by rare finds in the *tenuicostatum* zone in Fonte Coberta (Fig. 2; supplements). These finds may indicate that *Soaresirhynchia* already partially took hold in the Lusitanian Basin before the T-OAE. This earlier occurrence coincides with the end of the Pliensbachian-Toarcian boundary perturbation, an event that has been described as a significant extinction event and thermal perturbation in its own right^9,11,20^. The sediments in Fonte Coberta are interpreted to represent deeper water environments than at Barranco de la Cañada^21,28^. In Fonte Coberta, *Soaresirhynchia* is found together with two other brachiopod genera showing very similar shell ultrastructure and geochemical composition, pointing at comparable biomineralisation styles, and suggesting that physicochemical conditions at Fonte Coberta aligned better with the ecological requirements of *Soaresirhynchia* than in basins east of the Iberian Massif (Fig. 3). Indeed, this morphologically simple but variable brachiopod is thought to be a representative of a group of deep water brachiopods that invaded the shallow shelf seas during times of environmental perturbation^62^.

The reduced isotopic heterogeneity in its shell calcite compared with other Toarcian rhynchonellids from shallow water habitats (Fig. 3) suggests that this genus grew comparatively slowly. Such an interpretation is further supported by the low Sr/Ca and Mg/Ca ratios in its shell (Fig. 3), which in modern brachiopods are preferentially observed for species from habitats below the photic zone^30,33^. In addition, the reduced shell organic matter content as compared to the shallow water brachiopods at Barranco de la Cañada signifies that *Soaresirhynchia* secreted shell calcite at very little energy expense^63^ and thus that metabolic rates were low.

*Soaresirhynchia* has the coarse shell fibres with isometric cross sections typical of the rhynchonellid superfamily Basilioloidea^64^, which is thought to thrive in warm waters^65^. The association of *Soaresirhynchia* with low palaeolatitudes with only rare finds beyond 35°N in palaeolatitude^22,60^ further suggests that this genus relied on warm waters. Indeed, its disappearance in the Barranco de la Cañada section is marked by the first indications for a return to cooler palaeotemperatures (Fig. 2). A thermophilic character of this genus, however, seems at odds with its supposed deeper water origin. One might rather hypothesize that *Soaresirhynchia* could tolerate a wide temperature range and thrive where competition with less well adapted species was reduced or absent^26^.

The advent of *Soaresirhynchia*, occupying a large part of western Tethyan shallow shelf seas and ranging far beyond its earliest Toarcian appearance in the Lusitanian Basin has been ascribed to the pioneering repopulation after severe benthic turnover of the T-OAE^22,66^. However, considering the protracted hyperthermal conditions along with major changes in ocean chemistry caused by expanded anoxia elsewhere in the global ocean and increased continental weathering fluxes^36,67^, one should not equate this repopulation phase with a return to normal environmental conditions. Besides the ability of *Soaresirhynchia* to withstand high temperatures it could also survive in seawater characterized by substantial changes in ion content, having been depleted in sulphate^52,68^ and enriched in nutrients^36,37^ (Fig. 4) as a consequence of the T-OAE. The observed faunal turnover in brachiopods across the T-OAE may serve as an analogue for similar scenarios of faunal changes that are possible outcomes of ongoing alterations in climate and oceanic nutrient balance^51^. Shallow marine environments might become occupied by similarly unlikely survivors, calling for particular study and conservation of deeper water faunas.

## Conclusions

Well-preserved benthic macrofossils from Toarcian strata of Portugal and Spain allow for the reconstruction of palaeoenvironmental conditions during the early Toarcian in unprecedented detail. The negative carbon isotope excursion of the T-OAE is constrained to a magnitude of c. 4.2‰. The associated palaeotemperature rise of c. 3.5 °C lasted until carbon isotope ratios recovered to maximum values in the studied sections.

Modelling of the newly generated data suggests that the carbon cycle perturbation of the T-OAE could have been caused by the injection of carbon primarily derived from the interaction of large igneous province volcanism with sedimentary organic matter and methane, leading to an approximately doubled atmospheric pCO_2_. The prolonged duration of the thermal perturbation associated with the T-OAE is likely to have been controlled primarily by the silicate weathering flux, making the distinction between silicate supply versus kinetic components of this flux during this and other OAEs an important focus for future research.

The rhynchonellid brachiopod genus *Soaresirhynchia* occurs abundantly during the rising limb of the T-OAE negative carbon isotope excursion, an interval characterised by persisting elevated temperatures. New geochemical and microscopic data corroborate that this genus likely originated in deeper water habitats. Its success during the stressful conditions of the early Toarcian can be linked to its ability to tolerate hot temperatures and its inferred low metabolic rates.

## Supplementary information

Supplementary Information.

## Data Availability

All geochemical data pertaining to this study are included in this published article (and its Supplementary Information files). SEM images of shell ultrastructures are available from the corresponding author on reasonable request. Fossil specimens from the Fonte Conberta/Rabaçal sections are curated and archived in the Museum für Naturkunde, Leibniz Institute for Evolution and Biodiversity Science, Berlin, Germany. Studied specimens from the Barranco de la Cañada section are curated and archived in the Museu de Ciencias Naturales, Zaragoza, Spain.
