# Supplementary material for: Warm afterglow from the Toarcian Oceanic Anoxic Event drives the success of deep-adapted brachiopods
Source: Sci Rep. 2020 Apr 16;10:6549. doi: 10.1038/s41598-020-63487-6 (PMC7162941; doi:10.1038/s41598-020-63487-6)
Supplement: Supplementary file 1 — Supplementary Information. [file 41598_2020_63487_MOESM1_ESM.pdf]

Supplementary Materials for

"Warm afterglow from the Toarcian Oceanic Anoxic Event drives the success of deep-adapted brachiopods"

Ullmann, C.V.*, Boyle, R., Duarte, L.V., Hesselbo, S.P., Kasemann, S., Klein, T., Lenton, T., Piazza, V., Aberhan, M.

*c.v.ullmann@gmx.net

Contents:

Geological background
Analytical methods
Assessment of fossil preservation
Geochemical assessment of shell preservation
Palaeoenvironmental significance of data
Modelling
SI References

Geological background

We studied the Toarcian succession of the NW Tethyan shelf at two representative localities in the surroundings of the Iberian Massif. These are the Barranco de la Cañada section (40°23'53.4"N 1°30'07.4"W) near Albarracín in the Iberian Basin, Spain, and the composite sections of Fonte Coberta (40°03'36.5"N 8°27'33.4"W) and Rabaçal (40°03'08.0"N 8°27'30.5"W) near Coimbra in the Lusitanian Basin, Portugal[1-4]. Both sections are fossiliferous, with brachiopods and bivalves being the dominant macrobenthic groups, are well constrained biostratigraphically, and record fairly continuous sedimentation without any obvious hiatuses.

Barranco de la Cañada is located in the Iberian Range, a NW trending fold- and thrust-belt in east-central Spain that, in the early Mesozoic, represented a system of shallow marine platforms connected to other European epicontinental basins northward and the Tethyan Ocean in the southeast[5]. The Toarcian succession is represented by the Turmiel Formation which marks a progressive Toarcian deepening of the platform until the mid-Toarcian Bifrons Zone[6,7]. The ca. 27-m-thick sampled interval ranges from the Pliensbachian/Toarcian boundary to the lower Bifrons Zone (middle Toarcian) and is biostratigraphically constrained by ammonites, brachiopods, and foraminifers[7,8]. It consists of rhythmic alternations of limestones and marlstones. The partly argillaceous limestones primarily comprise wackestones, mudstones, and floatstones indicative of low-energy conditions. Only very few packstone and rudstone beds suggest brief episodes of higher water energy. Deposition took place in a mid-ramp setting mostly below storm wave base at an estimated water depth of 40–70 m[7,9], with the occasional higher energy limestone variants being interpreted as distal storm flow beds[10]. In terms of sequence stratigraphy (summarized in Refs. 5,7,9,11) the studied interval is part of a second-order transgressive-regressive cycle (LJ-3 in Ref 5) ranging from the Pliensbachian Davoei Zone to the middle Toarcian Variabilis Zone. The transgressive phase occurred in three distinct pulses, of which the second (LJ3-2) and parts of the third (LJ3-3) cycle fall within the study interval. LJ3-2 starts in the early Tenuicostatum Zone with maximum flooding in the lower Semicelatum Subzone, whereas LJ3-3 starts in the lower Serpentinum Zone with maximum transgression in the Bifrons Zone[5].

At Fonte Coberta/Rabaçal, Portugal, the ca. 28-m-thick studied succession from the Pliensbachian/Toarcian boundary (base of the Polymorphum Zone = Tenuicostatum Zone of the Submediterranean Province) to the middle of the Levisoni Zone (=

Serpentinum Zone) comprises three members of the São Gião Formation[12,13]. Biostratigraphic control is provided by ammonites (e.g. Refs. 13,14), nannofossils[15], and dinoflagellates[16]. Similar to Barranco de la Cañada, the section is composed of an alternation of marlstones and partly argillaceous limestones (mudstones and wackestones). This hemipelagic sequence was deposited on a low-energy, middle to distal homoclinal ramp below storm wave base, at an estimated water depth of 80–120 m[5,17], i.e. in a slightly deeper water setting than the sediments at Barranco de la Cañada. In terms of sequence stratigraphy, the base of the Polymorphum Zone marks the basal Toarcian transgression, an isochronous event within the Lusitanian Basin[18,19]. The first lithological member (Marly limestones with *Leptaena* Fauna) is interpreted as a transgressive systems tract with low sedimentation rates. The second member (Thin nodular limestones Member) and the third member (Marls and marly limestones with *Hildaites* and *Hildoceras*) represent two phases of the same sequence, with the former being interpreted as low-stand systems tract related to tectonic activity[20] in the basin, and the latter as a transgressive systems tract. A somewhat different view was presented by Gahr (2005)[9], who argued for three different depositional sequences instead of two, with the second member being at least partly related to the basal Toarcian transgression. A conspicuous feature of both sections is the absence of black shales which commonly characterize the Toarcian Oceanic Anoxic Event elsewhere (e.g., Refs. 21-24). In contrast, the total organic carbon content is generally low[25,26], while body fossils and/or ichnofossils occur continuously throughout the sections[4,7,27]. Accordingly, the marine habitats at both localities were characterized by well oxygenated conditions throughout the early to middle Toarcian time interval[4,7]. Also the ichnofauna, while undergoing important changes, never vanishes entirely suggesting that bottom water oxygenation was never insufficient to sustain benthic life for extended periods of time[3,28].

Analytical methods

Fossil specimens were collected from sedimentary strata in Barranco de la Cañada and Fonte Coberta/Rabaçal and identified to species level (**Table S1**). A subset of these fossils was selected for geochemical analysis

Scanning Electron Microscope (SEM)

Calcite splinters of ca 1 mm width were extracted from the fossils and placed on SEM stubs (**Figs S1,2**). The stub was carbon coated and SEM observations carried out under high vacuum. Images were taken using an FEI Quanta 650 Field Emission Gun Scanning Electron Microscope (FEG SEM) at the University of Exeter, Penryn Campus, Environment and Sustainability Institute at 15 kV and typical working distance between 10 and 11 mm.

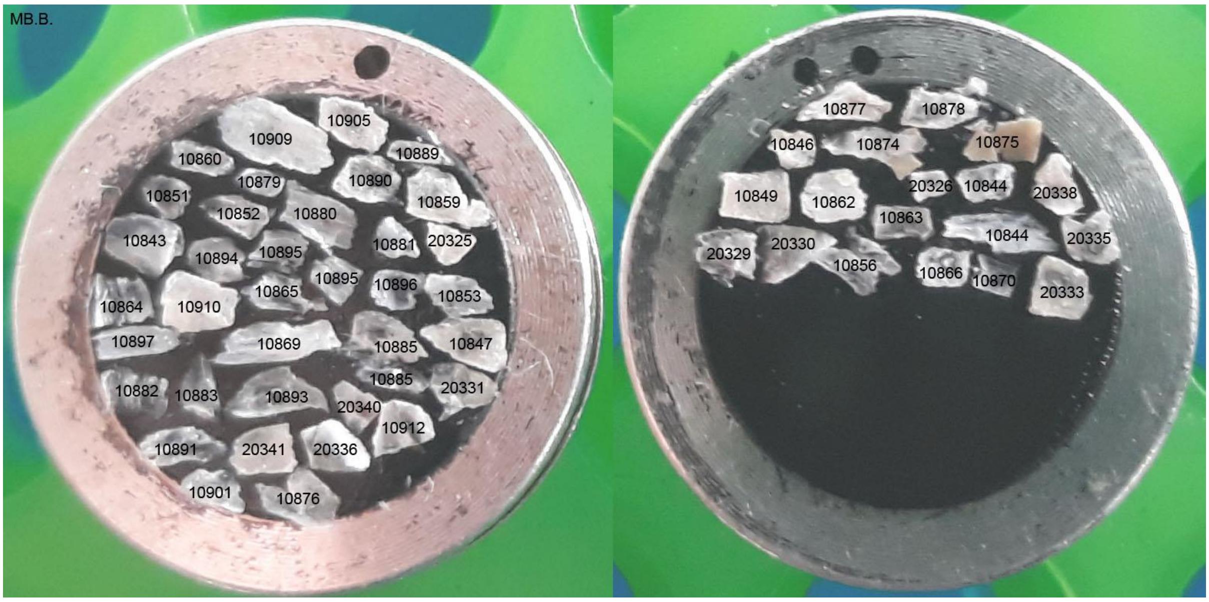

Figure S1: SEM stubs with fossil fragments from Fonte Coberta / Rabaçal. Numbers correspond to collection numbers of the Museum für Naturkunde Berlin (Germany), see below. The diameter of the stubs is 12 mm.

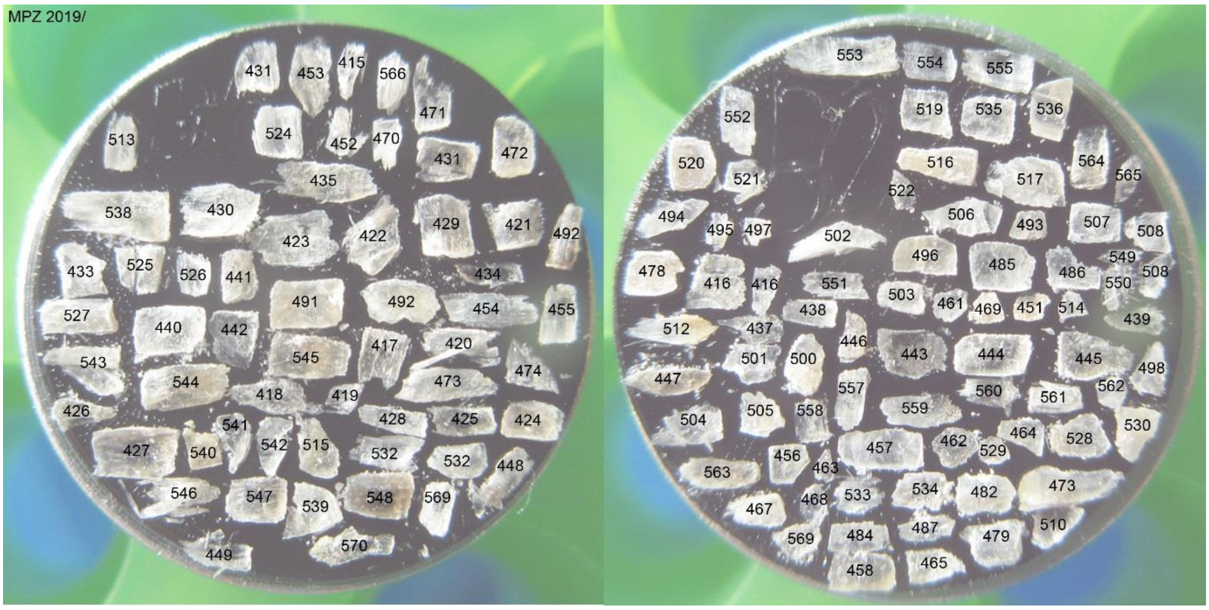

Figure S2: SEM stubs with fossil fragments from the Barranco de la Cañada section. Numbers correspond to collection numbers of the Museu de Ciencias Naturales, Zaragoza (Spain), see below. The diameter of the stubs is 12 mm.

Fossil preparation

Fossil material from Fonte Coberta / Rabaçal is archived at the Museum für Naturkunde, Berlin, Germany (samples MB.B.10843-10912 for brachiopods and MB.B.20325-20346 for bivalves). Shell fragments from Barranco de la Cañada are stored at the Museu de Ciencias Naturales, Zaragoza, Spain (samples MPZ 2019/415-571; Ref. 29).

For all fossil shells the surface was cleaned from sediments and altered rinds, and for brachiopods the primary shell layer was removed if present using a preparation needle, scalpel or hand-held drill with diamond coated drill bit. Visually best-preserved material was targeted and specimens where only clearly altered shell material was present not sampled. For most fossil specimens, shell material was extracted as sheaths of multiple shell layers with a preparation needle. Where this was not possible due to the dense nature of the material (mostly for some specimens of *Gryphaea*), samples were taken with a scalpel or hand-held drill using a diamond coated drill bit of ca 1 mm diameter. Sample sizes ranged from 0.9 to 14 mg (typically 1 – 3 mg) for fossils.

For each stratigraphic interval apart from levels C12 and BC22 for Barranco de la Cañada and 22 horizons for Fonte Coberta / Rabaçal a sample of bulk rock was extracted with a hand-held drill from the bulk rock matrix of a suitable fossil specimen. Sample sizes ranged from 7 to 31 mg (typically 15 to 30 mg). Where present, sparitic calcite cements

filling or partially filling brachiopod fossils were sampled as well. Sample sizes ranged from 3 to 29 mg (typically 5 to 15 mg).

All 550 samples (4 belemnite samples, 130 bivalve samples, 359 brachiopod samples, 41 bulk rock and 16 cement samples) for Barranco de la Cañada and 135 samples for Fonte Coberta / Rabaçal were collected and stored in 2 mL sample vials for later processing and archiving of surplus material for possible replicate measurements or future specialist analysis.

Element/Ca ratio determination

A fraction of each sample amounting to 220 to 820 µg (typically 330 to 550 µg) was weighed into 15 mL centrifuge tubes using a Micro Balance at a precision of 1µg. The centrifuge tubes were then weighed at 10 µg precision using either a model semi-microbalance. 2% v/v nitric acid prepared from 18.2 MΩ water and 67% concentrated nitric acid was added from an FEP wash bottle to dissolve the fossil calcite at a ratio of ca 15 mL per mg resulting in a dilution to ca 25 µg/g Ca. After dilution the tubes were weighed again with the same semi-microbalance to allow for the computation of precise dilution factors.

The samples were analysed for element/Ca ratios using an Agilent 5110 VDV Inductively Coupled Optical Emission Spectrometer (ICP-OES) with Seaspray U-series glass nebulizer and double pass cyclonic spray chamber. Sample and standard transfer was automated by an Agilent SPS4 autosampler.

Analysis was done in batches of 120 samples subdivided into blocks of 30 samples bracketed by calibrations and subdivided into sets of 10 samples by quality control solutions (BCQC & BCQ2) and international reference materials (JLs-1 limestone[30]; UN AK carbonate[31]). This protocol resulted in 5 calibrations, 12 analyses of JLs-1, 8 analyses of UN AK, and 4 analyses of BCQC or BCQ2 per batch of 120 samples.

Three synthetic calibration solutions were prepared from certified 1,000 µg/g single element plasma standards. A uniform Ca concentration of 25 µg/g was opted for to match the matrix of the fossil samples. For all other elements contents were variable to cover the expected range of element/Ca ratios. The calibration solutions were prepared gravimetrically by incremental addition of single element standards into a 1 L bottle and differential weighing using a Semi-microbalance with 10 µg precision up to a weight of 120 g and 100 µg precision up to a weight of 230 g. The final weight of the calibration

solutions after filling up to 1 L with 25 v/v nitric acid was determined using an top loader balance with precision of 0.01 g. Comparability of the weighing results was ensured by weighing the empty bottles on either balance which gave indistinguishable results. For Ca concentrations a two-point calibration with calibration blank and three solutions of near equivalent Ca concentration resulted. For all other analytes, a linear four point calibration with a calibration blank and three calibration solutions with different concentrations was made. Covered calibration ranges are 0 to 33.4 mmol/mol for Mg/Ca, 0 to 3.66 mmol/mol for Sr/Ca, 0 to 5.81 mmol/mol for Fe/Ca, 0 to 2.93 mmol/mol for Mn/Ca, 0 to 13.1 mmol/mol for P/Ca, and 0 to 12.7 mmol/mol for S/Ca ratios.

Analysed wavelengths are 317.933 nm and 422.673 nm for Ca, 279.553 nm, 280.270 nm and 285.213 nm for Mg, 407.771 nm and 421.552 nm for Sr, 238.204 nm and 259.94 nm for Fe, 257.610 nm and 259.372 nm for Mn, 213.618 nm for P and 181.972 nm for S. Signals were quantified synchronously for all wavelengths in 12 blocks of 5s each resulting in signal integration over 60s per measurement. Optimum wavelengths for Mg/Ca and Sr/Ca results were chosen to maximize repeatability of the QC and standard solutions during data reduction. Results for both analysed wavelengths for Fe and Mn were pooled to improve signal to noise ratios.

Typical quantification limits of the method measured as 6 standard deviations of the baseline variability are 2 ng/g for Ca, 0.3 ng/g for Mg, 0.02 ng/g for Sr, 0.5 ng/g for Fe, 0.2 ng/g for Mn, 10 ng/g for P and 20 ng/g for S. Resulting effective quantification limits for element/Ca ratios in the carbonate are 0.02 mmol/mol for Mg/Ca, 0.4 μmol/mol For Sr/Ca, 0.02 mmol/mol for Fe/Ca, 0.006 mmol/mol for Mn/Ca, 0.5 mmol/mol for P/Ca and 1 mmol/mol for S/Ca ratios.

Reproducibility over six analytical sessions for Barranco de la Cañada was controlled by 72 measurements of JLs-1, 48 measurements of UN AK and 24 measurements of BCQC. Over two analytical sessions for materials from Fonte Coberta / Rabaçal 24 aliquots of JLs-1, 16 aliquots of UN AK and 8 aliquots of BCQ2 were measured. Reproducibility depends on absolute analyte concentrations and is controlled by integration time when concentrations are low and random noise when concentrations are high. Consequently, element/Ca ratios which were higher than 100 times the quantification limit reproduced within 1 % (2 sd) for all reference materials. Largest 2 sd uncertainties for element/Ca ratios of reference materials where element/Ca ratios are below 100 times the quantification limit are 0.05 mmol/mol for Mg/Ca, 0.005 mmol/mol for Sr/Ca, 0.002

mmol/mol for Mn/Ca, 0.06 mmol/mol for Fe/Ca, 0.28 mmol/mol for S/Ca, and 0.17 mmol/mol for P/Ca. Analytical results for all measured standard solutions are listed in **Table S2**.

Internal consistency of the measurements was ascertained by the quality control solution (BCQC and BCQ2) which was prepared in the same way as the calibration solutions but was not used for signal quantification. Relative bias of the analytical results for this quality control solution was smaller than 1 % for all analytes (**Table S2**).

Stock solutions for JLs-1 and UN AK were prepared from 0.5 L 1,000 µg/g stock solutions generated by the dissolution of ca 1.25 g of either of these standards using 2% v/v nitric acid. Due to minor non-carbonate fractions in both standards that were not dissolved in the nitric acid the results for some of the elements are therefore slightly biased. For the standards measured alongside samples from Barranco de la Cañada averages (± 2sd) for JLs-1 (n = 72) are Mg/Ca: 15.37 ± 0.07; Sr/Ca: 0.341 ± 0.005; Fe/Ca: 0.14 ± 0.01 mmol/mol; Mn/Ca: 0.032 ± 0.001 mmol/mol; P/Ca: 0.41 ± 0.17 mmol/mol; S/Ca: 0.43 ± 0.22 mmol/mol. Averages (± 2sd) for UN AK (n = 48) are Mg/Ca: 2.47 ± 0.02 mmol/mol; Sr/Ca: 3.01 ± 0.01 mmol/mol; Fe/Ca: 1.11 ± 0.06 mmol/mol; Mn/Ca: 0.043 ± 0.001 mmol/mol; P/Ca: 0.39 ± 0.17 mmol/mol; S/Ca: 1.73 ± 0.18 mmol/mol.

Stock solutions for the samples from Fonte Coberta / Rabaçal were prepared from a differing standard batch and therefore give slightly differing results from the data obtained for Barranco de la Cañada. Averages (± 2sd) for JLs-1 (n = 24) are Mg/Ca: 15.4 ± 0.1; Sr/Ca: 0.342 ± 0.002; Fe/Ca: 0.155 ± 0.004 mmol/mol; Mn/Ca: 0.032 ± 0.002 mmol/mol; P/Ca: 0.43 ± 0.09 mmol/mol; S/Ca: 0.50 ± 0.28 mmol/mol. Averages (± 2sd) for UN AK (n = 16) are Mg/Ca: 2.64 ± 0.05 mmol/mol; Sr/Ca: 3.04 ± 0.02 mmol/mol; Fe/Ca: 1.366 ± 0.007 mmol/mol; Mn/Ca: 0.045 ± 0.002 mmol/mol; P/Ca: 0.40 ± 0.15 mmol/mol; S/Ca: 1.82 ± 0.21 mmol/mol.

Absolute Ca concentrations calculated for fossils reproduced to better than 0.6 wt% (2 sd) for all brachiopod genera from Barranco de la Cañada for which at least 30 samples were taken (2 outliers). This variability is equivalent to an average of 5 to 8 µg sample material and is therefore thought to be mostly controlled by weighing imprecision of the micro balance and signal noise of the ICP-OES.

Possible external errors of element/Ca determinations and Ca concentrations relate to the uncertainty of element concentrations in certified 1,000 µg/g single element solutions taken for preparation of the calibration solutions. 95 % confidence intervals stated on the

certificates of analysis were 0.6 % of the analyte concentration or better for all studied elements.

C and O isotope ratio measurements

685 samples were analysed for $\delta^{13}C$ and $\delta^{18}O$ values as well as carbonate content using a SerCon 20-22 Gas Source Isotope Ratio Mass Spectrometer (GS-IRMS) in continuous flow mode at the University of Exeter Penryn Campus, Environment and Sustainability Institute.

Typically 450 to 600 µg of fossil material, bulk rock powder or calcite cement weighed to 1 µg precision in randomized order using a model Micro Balance and were transferred into 4.5 ml borosilicate Labco Exetainers®. Batches of 80 samples were analysed together with 22 aliquots of the in-house standard CAR (Carrara Marble, $\delta^{13}C$ = +2.10 ‰ V-PDB; $\delta^{18}O$ = -2.03 ‰ V-PDB) and 8 aliquots of the in-house standard NCA (Namibia Carbonatite, $\delta^{13}C$ = -5.63 ‰ V-PDB; $\delta^{18}O$ = -21.90 ‰ V-PDB). These standards were previously calibrated against the international standards NBS-18, CO-8 and LSVEC as well as in-house standards of Freie Universität Berlin (CAM, Carrara Marble; LM, Laaser Marble) and University of Copenhagen (LEO, Carrara Marble). 450 to 550 µg of carbonate powder weighed to 1 µg precision were used for 17 aliquots of CAR and all aliquots of NCA in each batch. The remaining 5 aliquots of CAR ranged from ca. 200 µg to ca. 1,000 µg and were used to define a calibration curve of sample weight versus signal yield, which is used to estimate carbonate concentrations of the bulk powders.

All samples were held at 70°C and flushed with He for 80 s before manual injection of c. 100 µL of nominally anhydrous phosphoric acid. Samples from Barranco de la Cañada were measured alternating with a reference gas which yielded typical peak areas of 201 nAs for mass 44, equivalent to the signal from 551 µg of calcite. Samples from Fonte Coberta / Rabaçal were analysed after replacement of the source filament of the 20-22 GS-IRMS, change of Helium pressure control installations and CO_2 reference gas, resulting in slightly lower reference gas intensities of 192 nAs for mass 44, equivalent to the signal from 501 µg of calcite. For samples from Barranco de la Cañada signal yield of the in-house reference NCA (n = 56) was 97.1 ± 1.7 % (2 sd) versus the in-house reference CAR. For samples from Fonte Coberta / Rabaçal NCA (n = 24) signal intensity was 97.5 ± 0.9 % (2 sd) versus CAR. External errors of carbonate content estimations relate to the uncertainty of the carbonate content in CAR which was assumed to be pure $CaCO_3$ (44.0

wt% CO_2). Minimal non-carbonate impurities, as well as minor adsorbed water on the powdered standard material could have biased the measurements.

Isotopic drift of the instrument reaching up to ca. 0.3 ‰ during an analytical session was controlled with both, the raw isotopic ratios of the reference gas and CAR and subtracted from the data using a polynomial fit to the raw data for CAR. Shift and compression of the isotopic scale were corrected using a two-point calibration of CAR and NCA. For Barranco de la Cañada samples, 2 sd repeatability of CAR (n = 125) was found to be 0.07 ‰ for $\delta^{13}C$ and 0.15 ‰ for $\delta^{18}O$ and 2 sd repeatability of NCA (n = 56) was 0.09 ‰ for $\delta^{13}C$ and 0.35 ‰ for $\delta^{18}O$. For Fonte Coberta / Rabaçal samples, 2 sd repeatability of CAR (n = 54) was found to be 0.06 ‰ for $\delta^{13}C$ and 0.14 ‰ for $\delta^{18}O$ and 2 sd repeatability of NCA (n = 24) was 0.08 ‰ for $\delta^{13}C$ and 0.28 ‰ for $\delta^{18}O$.

All analytical result for isotopic and CaCO3 concentration measurements of standard materials are listed in **Table S3**.

Assessment of fossil preservation

Excellent preservation of fossil material is a prerequisite for high fidelity palaeoenvironmental reconstructions using geochemical proxies from biogenic calcite. Brachiopods as well as oysters are widely studied[32-34] and their shell structure and geochemical patterns well understood so that diagenetic impacts on their calcite can be spotted confidently[35]. Additionally, the low magnesium calcite of rhynchonellid brachiopods and oysters is resistant against post-depositional recrystallization as compared to mineralized tissues of aragonitic or high magnesium calcite secreting organisms.

Reliable identification of diagenetically overprinted material using optical and chemical preservation parameters, however, is complicated. Optical assessment suffers from the fact that visual changes to the shell material are seldom uniform for an entire fossil and one can only qualitatively link optical signs of alteration to changes in chemical and isotopic parameters. Qualitative trends of diagenesis on geochemical parameters in fossil calcite are well-studied, but the local burial and exhumation history of outcropping sedimentary successions can be complicated and will define thresholds of chemical preservation markers.

To overcome these challenges, a comprehensive study of shell preservation employing optical (binocular miscroscope, SEM) and chemical (element/Ca ratios and C & O isotope ratios) techniques was employed. For Barranco de la Cañada, clearly diagenetic phases, in particular geopetal cements encountered in 15 brachiopods, as well as bulk rock samples were analysed for element/Ca and C & O isotope ratios alongside the fossil shells to control the geochemical composition of diagenetic endmembers. Geochemical patterns compatible with diagenesis observed in fossil calcite could then be matched with bulk and cement signals and suitable geochemical thresholds defining good shell preservation defined accordingly. For Fonte Coberta / Rabaçal, for which sparitic calcite was not observed in the studied specimens, only bulk rock data were used as proxy for diagenetic trends.

Optical assessment of shell structure preservation

Rhynchonellid brachiopods

Under the binocular microscope, secondary layer calcite of rhynchonellid brachiopods from Barranco de la Cañada looks exceptionally well preserved. The typical silky

reflection and slightly brownish semi-transparent colour attest to minimal recrystallization. Sample extraction with a preparation needle resulted in well-defined packages of multiples layers of shell fibres detaching along fibre surfaces, rather than disintegrated single fibres or calcite blocks with fracture surfaces oblique to the shell structures. Only on some specimens of *Soaresirhynchia* the coherence of the secondary layer calcite fibres was somewhat compromised and single fibres tended to detach from the shell and break into fragments of sugar-like appearance.

When observed under the SEM, many shell fibres of secondary layer calcite show surficial, pyramid shaped dissolution pits of less than 10 µm depth developed nearly exclusively on exposed surfaces of shell fibres (**Fig. S3**). These dissolution pits, where they are asymmetrical, show uniform orientation on single fibres. Morphologies and orientation of the pits change from fibre to fibre, indicating that the single-crystal character of the fibres is preserved and no pervasive re-crystallisation has taken place. Exposed surfaces of shell fibres are otherwise smooth and show no signs of fusing with adjacent fibres, and imprints of adjacent fibres as well as features of pressure solution and neomorphic calcite are absent. Terebratulid material with large cement-filled punctae as well as clearly recrystallized brachiopod calcite (**Fig. S3**) was not sampled for geochemical analysis.

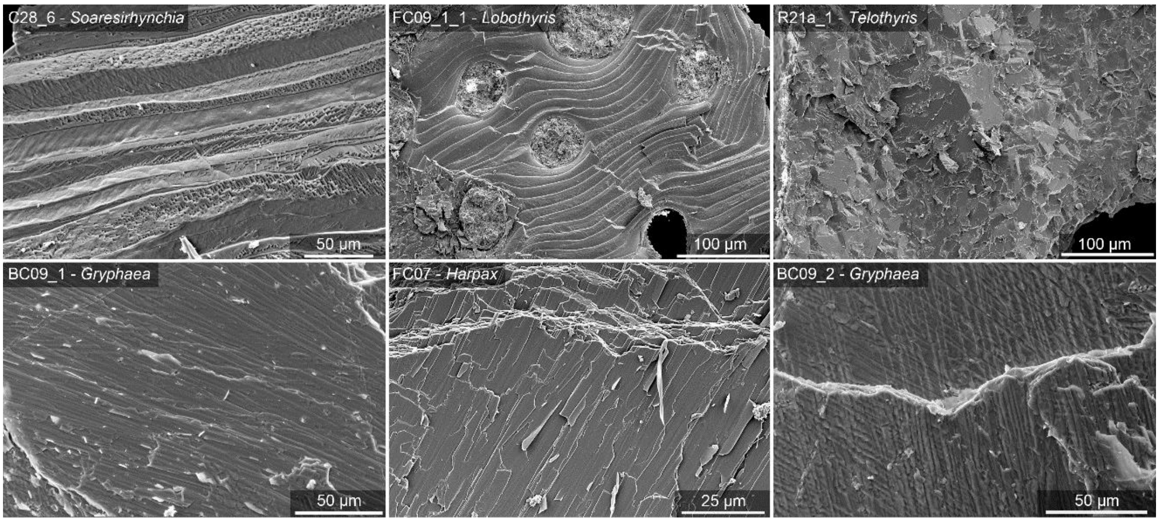

Figure S3: SEM secondary electron images of brachiopods and bivalves from the Barranco de la Cañada and Fonte Coberta / Rabaçal sections. Dissolution pits of a few micrometres depth in brachiopod shells as observed in *Soaresirhynchia* (top left) are common. Terebratulids with large, cement-filled punctae (top middle) and clearly recrystallized shell material (top right) were not processed for geochemical analyses. Shell ultrastructure preservation of bivalves (*Gryphaea*, bottom left; *Harpax*, bottom middle) is often very good. In many instances, however, imprints of surrounding shell fibres are observed (bottom right). Were these effects are pronounced, clear changes in element/Ca, C and O isotope ratios are observed (one sample

of *Gryphaea* specimen BC09_2 had to be excluded from palaeoenvironmental interpretation). For stratigraphic position of imaged material, see **Table S4**.

Brachiopod material from Fonte Coberta / Rabaçal showed the same morphological characteristics as specimens from Barranco de la Cañada. Overall, a larger fraction of specimens which were not further investigated for geochemical parameters due to spurious preservation observed already using a binocular microscope, showed diagenetic overprints, such as fusion of shell fibres and neomorphic calcite.
Overall, optical assessment of the shell calcite of specimens subjected to geochemical analyses did not suggest that the geochemical signatures in the material would be significantly compromised.

Bivalves
Gryphaea shell material from Barranco de la Cañada appears less well preserved than brachiopod shells and a range of features indicative of diagenetic overprint were encountered during visual inspection with the binocular microscope. Fusion of shell layers and irregular surfaces indicative of incipient recrystallization, sometimes even loss of discernible shell structure were observed, but these features were seldom pervasive. Often, partially altered specimens still yielded patches of shell material with the typical stacks of thin, translucent sheaths of calcite layers that could be levered off the shell with a preparation needle. These visually best preserved areas were targeted for sampling for geochemical analysis.
SEM observations confirm that, compared to rhynchonellids, a larger fraction of *Gryphaea* calcite was affected by alteration. Dissolution pitting is almost absent, but neomorphic calcite is sometimes observed and surfaces of shell layers often bear the imprint of the overlying shell fibres, indicating earliest stages of fusing fibre sheaths as well as recrystallization (**Fig. S3**). Nevertheless, primary shell textures even on the micrometre scale are preserved in nearly all studied shell fragments, so that textural evidence suggests that geochemical preservation of the *Gryphaea* material should be good to very good.
Samples of the bivalve genus *Harpax* from Fonte Coberta / Rabaçal show ultrastructural features generally comparable to *Gryphaea* from Barranco de la Cañada (**Fig. S3**). However, as observed also for brachiopods, the preservation in general appears somewhat poorer than samples from Barranco de la Cañada as evidenced by more

pronounced fusion of shell layers. Consequently, data from *Harpax* are not further considered for palaeoenvironmental interpretation in this study.

Geochemical assessment of shell preservation

Bulk rock geochemistry Barranco de la Cañada

The Toarcian sedimentary strata of the Barranco de la Cañada section from which fossils were extracted are typically carbonate rich marls with carbonate contents of 46 to 94 % (median 81 %, n = 42; **Table S4, Figure S4**) according to mass spectrometry results. Carbonate contents below 70 % were only observed for four samples.

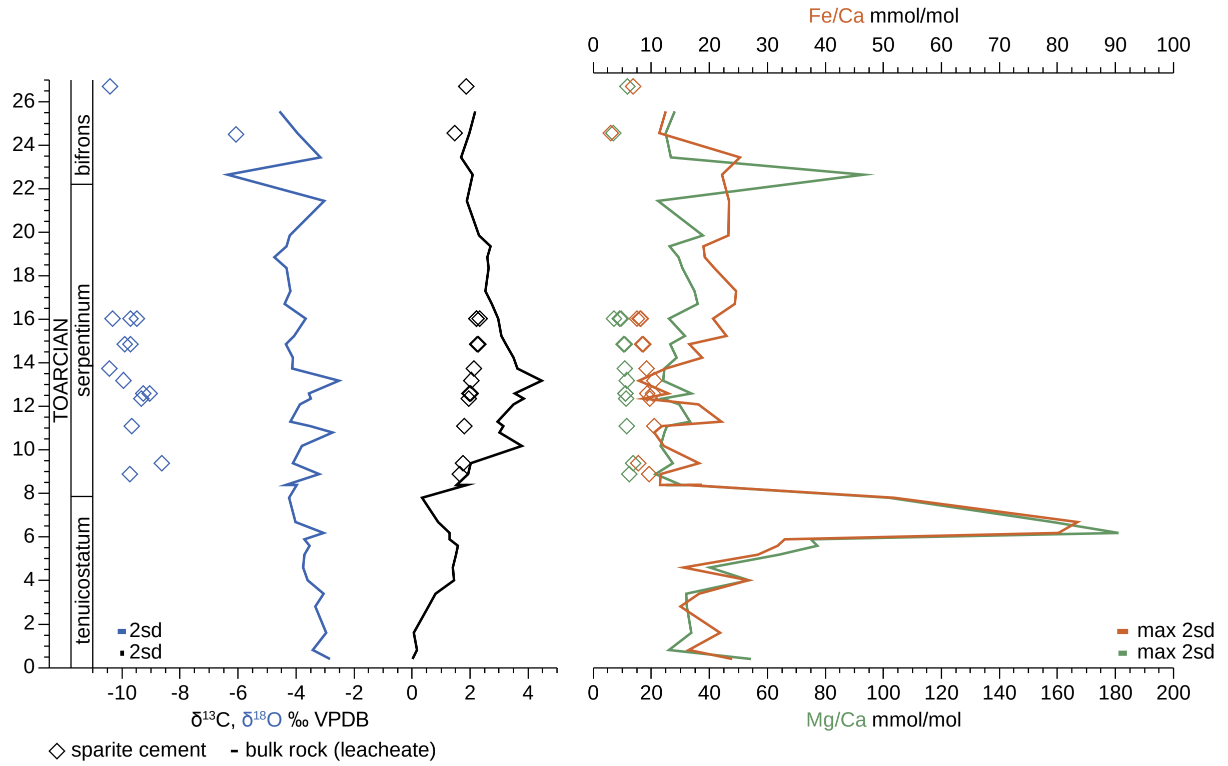

Figure S4: $\delta^{13}C$ and $\delta^{18}O$ values and Fe/Ca and Mg/Ca ratios of bulk rock and sparite cement samples from the Barranco de la Cañada section.

ICP-OES measurements of the 42 bulk rock samples (**Table S4**) show that the majority of the bulk rock matrix dissolved by the 2 v/v HNO_3 is low magnesium calcite with nine measurements yielding Mg/Ca ratios greater than 50 mmol/mol. Besides sample BC18 (24.35m, top Serpentinum Zone), Mg/Ca ratios greater than 50 mmol/mol are restricted to the interval of samples C15 to C21+22/C25+26 (4.0 to 7.8 m, middle Tenuicostatum Zone to basal Serpentinum Zone). The rock leachates are relatively depleted in Sr with Sr/Ca ranging from 0.26 to 0.71 mmol/mol (median 0.42 mmol/mol), but enriched in Mn with Mn/Ca ratios of 0.34 to 1.07 (median 0.53 mmol/mol). Fe/Ca ratios are very high and highly variable ranging from 7.9 to 83.4 mmol/mol (median 18.8 mmol/mol).

With respect to $\delta^{13}C$ values, bulk rock carbonate shows a broadly similar pattern as brachiopod data (**Table S4**), but offset to lower values by 0.6 ‰ (median; range = -1.6 to +0.7 ‰). $\delta^{18}O$ values of bulk carbonate (**Table S4**) do not follow the macrofossil pattern and become progressively more negative upsection, initially fluctuating around -3.0 ‰ V-PDB at the bottom of the section and reaching values around -4.5 ‰ V-PDB at the top. The median offset between fossil and bulk rock $\delta^{18}O$ is -1.2 ‰ (range = -3.6 to +0.6 ‰). Assuming that diagenetic trends in the fossil carbonates led to an endmember similar to the bulk rock signature suggests that fossil calcite should be depleted in ^{13}C, ^{18}O and Sr, and enriched in Mg, Mn and Fe. Due to the highly Fe-enriched nature of the rock matrix, biases on isotope ratios > 0.2 % as well as biases on Sr/Ca ratios > 0.05 mmol/mol would be visible as enrichments in Fe/Ca well above 1 mmol/mol. Because of the comparatively high Mg/Ca ratios of the rock matrix, this threshold could potentially be associated with a bias in fossil Mg/Ca of 0.8 to 4.1 mmol/mol (median 1.7 mmol/mol).

Sparitic cements Barranco de la Cañada

Geopetal cements incompletely filling voids in brachiopods are observed from samples C15+26/C28 to BC8 (8.9 to 18.05 m, basal to middle Serpentinum Zone) and samples BC20 and BC22 (26.25 and 28.4m, lower Bifrons Zone). Both intervals with geopetal cements start immediately above samples with bulk rock Mg/Ca > 50 mmol/mol (**Figure S4**). The cements (**Table S4**) in the Serpentinum zone show stratigraphic trends in Mg/Ca, covering a range from 7.2 to 13.7 mmol/mol and falling upsection as well as a well-defined increase in $\delta^{13}C$ values from +1.6 to +2.3 ‰. Also, a weak downward trend in $\delta^{18}O$ values which range from -8.6 to -10.4 ‰ is observed. Sr/Ca (0.22 to 0.44 mmol/mol), Mn/Ca (0.65 to 0.85 mmol/mol) and Fe/Ca (7.5 to 10.5 mmol/mol) are more stable than the hosting rock matrix and do not show clear stratigraphic trends. The sparite sample at level BC20 is geochemically distinct with particularly low Sr/Ca of 0.05 mmol/mol and low Mg/Ca of 3.0 mmol/mol, high Mn/Ca of 2.50 mmol/mol and less negative $\delta^{18}O$ value of -6.1 ‰. Otherwise, however, the sparite samples of the Bifrons Zone are geochemically comparable to their counterparts in the Serpentinum Zone.
The strong depletion in ^{18}O as compared to the bulk carbonate suggests that the sparitic cements formed much later in the diagenetic sequence, either at elevated temperatures during burial or – more likely – involving meteoric fluids. Fluid migration may have been governed by lithology-controlled permeability as suggested by the high Mg/Ca ratios in

bulk rock leachates directly underlying both intervals of sparite occurrence. No geochemical links between sparite and surrounding rock matrix can otherwise be established suggesting limited effects of this void filling stage on geochemistry of carbonates in the section.

Partial isotopic overprint of fossil shell material approaching sparite-like $\delta^{18}O$ values would be a clear concern. No strong correlations of $\delta^{18}O$ values with Fe/Ca ratios with slopes (approximately 0.7 ‰ per mmol/mol) suggestive of such an alteration trend are observed in fossil material from levels where sparite is present, however. A measureable effect of sparite calcite on fossil geochemistry is therefore excluded.

Bulk rock geochemistry Fonte Coberta / Rabaçal

Toarcian samples from the Fonte Coberta / Rabaçal section from which fossils were extracted are similar to those of the Barranco de la Cañada section (**Table S4, Figure S5**). The samples are carbonate rich marls with carbonate contents of 47 to 89 % (median 71 %, n = 22) according to mass spectrometry results.

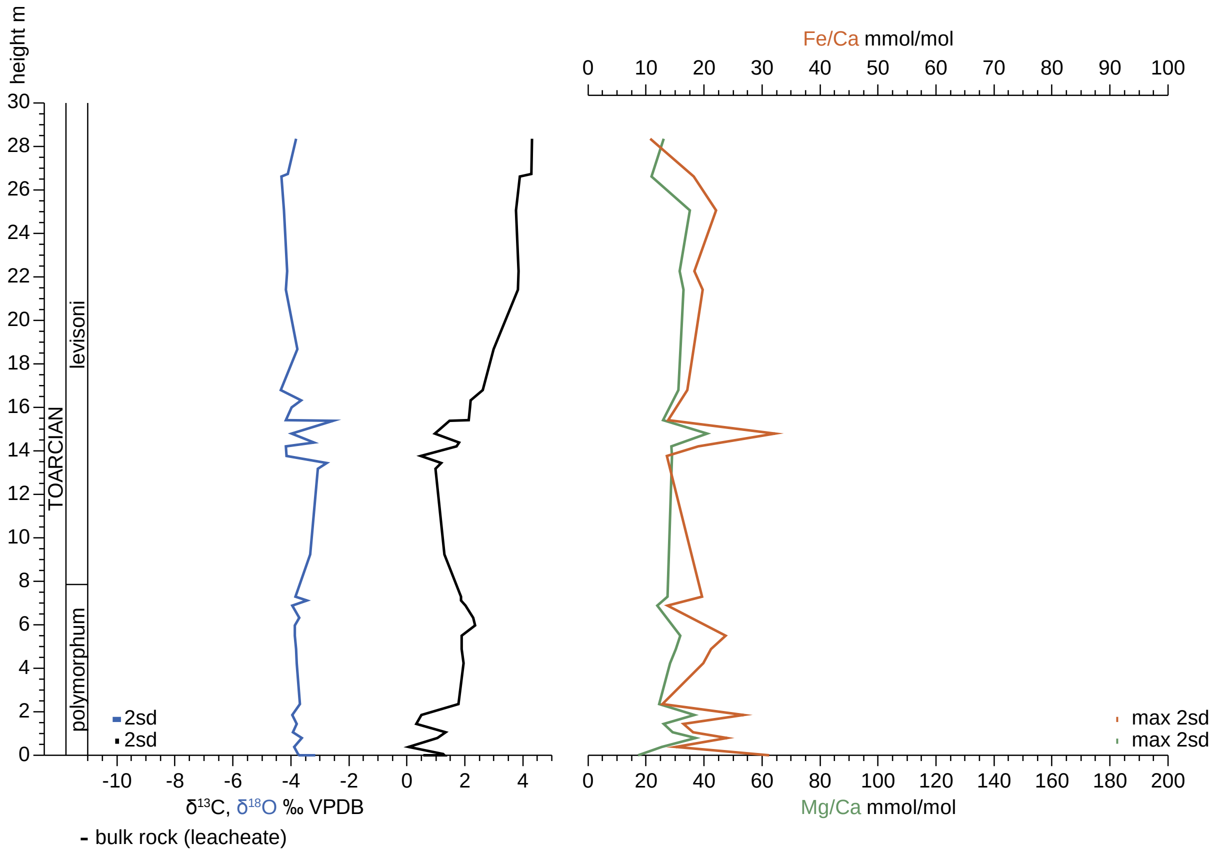

Figure S5: $\delta^{13}C$ and $\delta^{18}O$ values and Fe/Ca and Mg/Ca ratios of bulk rock samples from the Fonte Coberta / Rabaçal section.

ICP-OES measurements of the 22 bulk rock samples (**Table S4, Figure S5**) show that the bulk rock matrix dissolved by the 2 v/v HNO_3 is consistently low magnesium calcite with maximum Mg/Ca ratios reaching 41 mmol/mol in the lower *polymorphum* Zone. The rock leachates are generally slightly less Sr depleted than samples from Barranco de la Cañada with values ranging from 0.37 to 0.91 mmol/mol (median 0.55 mmol/mol). Mn/Ca ratios are comparable to Barranco de la Cañada with an observed range from 0.34 to 0.86 mmol (median 0.47 mmol/mol). As in Barranco de la Cañada Fe/Ca ratios are also high, but spikes coinciding with strong Mg enrichments are missing. Ratios of 10.8 to 32.2 mmol/mol (median 18.7 mmol/mol) are found.

Also C and O isotope trends in the Fonte Coberta / Rabaçal section are similar to those of Barranco de la Cañada (**Table S4, Figs. S4,5**). Bulk rock $\delta^{13}C$ values are typically 1 to 2 ‰ less positive than brachiopod samples from the same horizon and show a pattern comparable to the brachiopod data. Conversely, $\delta^{18}O$ values become progressively depleted in ^{18}O upsection with median values of -3.8 ‰ in the *polymorphum* Zone and -4.2 ‰ in the *levisoni* Zone.

The geochemical signatures in the Fonte Coberta / Rabaçal section suggest that chemical cut offs for fossil preservation similar to material from Barranco de la Cañada can be applied.

Covariation of geochemical proxies in fossil materials indicative of alteration

Barranco de la Cañada

The fact that isotopic trends observed in the rhynchonellid brachiopods and *Gryphaea* are distinct from bulk rock matrix and sparitic cements (**Table S4**) indicates that preservation of palaeoenvironmental information in the fossil calcite may be good. Subtle biases relating to partial recrystallization or addition of cements may nevertheless bias the proxy data and need to be assessed by detailed investigation of co-variations of geochemical indicators of diagenesis.

Co-variation of Fe/Ca ratios as the most sensitive proxy for alteration at the Barranco de la Cañada section in *Gryphaea* ($n = 130$) is only strong with Mn/Ca ($r^2 = 0.62$, $p < 0.001$) and Mg/Ca ($r^2 = 0.88$, $p < 0.001$). For the remaining element/Ca ratios the correlation coefficients are 0.26 or lower and for C & O isotope ratios, they are 0.06 or lower, signifying that the vast majority of signal is not controlled by processes adding iron to the calcite postdepositionally. When excluding all samples with Fe/Ca ratios > 1mmol/mol,

the correlation coefficient of Fe/Ca with Mg/Ca reduces to 0.04 and all samples with Mg/Ca ratios > 6 mmol/mol are excluded from the dataset. An Fe/Ca threshold of 1 mmol/mol for good fossil preservation as deduced also from comparison to bulk rock geochemistry is therefore deemed suitable for *Gryphaea*.

For rhynchonellid brachiopods (n = 359), only the co-variation of Fe/Ca ratios with Mn/Ca ratios is strong with a correlation coefficient of $r^2 = 0.70$. For all other correlations, the correlation coefficient is 0.18 (Sr/Ca) or lower. Considering that the studied dataset encompasses major, global geochemical perturbations, geochemical co-variation in single stratigraphic intervals specific to brachiopod genera was also tested. Only intervals where data density was deemed high enough to produce reliable results (10 specimens of a single genus or more) were tested. As expected, for these subsets correlation coefficients of Fe/Ca with Mn/Ca were partially much higher than the entire dataset. However, in addition to this no clear pattern consistent with diagenetic trends (loss of Sr as depletion in ^{13}C and ^{18}O in parallel with gains in Mg, Mn and Fe) could be identified.

In summary, it was concluded that minor diagenetic overprint of the material must have taken place as is evidenced by the enrichment of the shell calcite in Mn and Fe. These two elements are highly sensitive to partial diagenetic re-equilibration in the Barranco de la Cañada section, so that preservation of other geochemical proxies can robustly be controlled with Mn/Ca ratios and Fe/Ca ratios. In order to exclude altered material effectively, common limits of good preservation of 0.1 mmol/mol for Mn/Ca and 1.0 mmol/mol for Fe/Ca ratios were adopted. These limits led to the exclusion of 34 of 359 (9 %) brachiopod samples and 26 of 130 (20 %) *Gryphaea* samples. The larger fraction of excluded *Gryphaea* samples corroborates the optical assessment of the shell preservation that pointed at better preservation of brachiopod material. The number of excluded samples as compared to other studies on Mesozoic benthic organisms is small, however, further evidencing the overall excellent preservation of the shell material.

Fonte Coberta / Rabaçal

The isotopic ratios of fossil shell materials in the Fonte Coberta / Rabaçal section are distinct from the bulk rock signal, suggesting that primary information is retained in their calcite. Strong fluctuations in particular in $\delta^{13}C$ values relating to known environmental perturbations preclude the use of simple cross-plots of diagenesis proxies with isotope ratios to identify clearly overprinted samples. Comparison of geochemical signals within

single stratigraphic horizons suggests that isotopic proxies are insensitive to diagenetic overprint until Mn/Ca ratios of 0.05 and/or Fe/Ca ratios of 0.5 mmol/mol are reached. For example, samples of one individual of the genus *Cirpa* at 1.45 m height have $\delta^{13}C$ values of +3.0 to +3.3 ‰ and $\delta^{18}O$ values of -1.1 to -1.2 ‰ apart from a single sample with $\delta^{13}C$ value of +2.6 ‰ and $\delta^{18}O$ values of -2.3 ‰ where Mn/Ca is 0.050 and Fe/Ca is 0.57 mmol/mol. Because all but two of the fourteen samples taken from the bivalve *Harpax* cross the above preservation limits we only consider brachiopod data from Fonte Coberta / Rabaçal for palaeoenvironmental reconstruction.

Palaeoenvironmental significance of data

The reconstruction of palaeoenvironmental conditions on the basis of geochemical data requires that robust transfer functions exist for the studied parameters. In biogenic calcite geochemical signatures can be biased even in well-preserved materials by the presence of disequilibrium effects, generally known as "vital effects"[36,37]. Such effects associated with biological factors usually lead to the preferential incorporation of ^{12}C and ^{16}O into shell calcite and also compromise proxies based on element concentrations.

A pragmatic approach to minimise these challenges is to opt for fossil substrates that are thought to be least prone to vital effects. The calcite of the secondary shell layer in rhynchonellid brachiopods has been identified as a highly reliable substrate for geochemical proxies, especially when avoiding sampling from the shell hinge and specialized shell regions[38]. Similarly, the foliate calcite of modern oysters has been found to record environmental parameters with high fidelity[34]. From this point of view the fossil material chosen for study here is of the highest possible quality for investigating Early Jurassic palaeoenvironmental conditions.

In order to further boost confidence in the reliability of the proxy data, potential biases relating to the chosen brachiopod genus and potential inter-specimen and intra-specimen differences, e.g. potential offsets between ventral and dorsal valves were assessed. Furthermore, isotopic data for brachiopods were compared to *Gryphaea* data to assess biases between these fossil groups.

Intraspecimen variability of geochemical proxies

Geochemical compositions of ventral and dorsal valve of brachiopod specimens were only compared for genera for which at least three individuals with four samples that passed preservation criteria were available. This was the case for *Choffatirhynchia*, *Homoeorhynchia*, *Quadratirhynchia* and *Soaresirhynchia* from the Barranco de la Cañada section and *Soaresirhynchia* from the Fonte Coberta / Rabaçal section (**Fig. S6**). *Soaresirhynchia* specimens from the two sections were considered separately in case environmental conditions exerted a measurable control on shell geochemical proxies. For tested genera, differences in average isotope ratios range are generally negligible. Only $\delta^{18}O$ values in *Quadratirhynchia* show a minor offset of 0.2 ‰ in median values. This difference is based on nine measurements per valve only, however, and is not deemed to be large enough to warrant further consideration. The overall excellent correspondence

of isotopic signatures among dorsal and ventral valves of brachiopods instead is taken as evidence that sampling spot on the specimen does not exert a bias on the isotopic data.

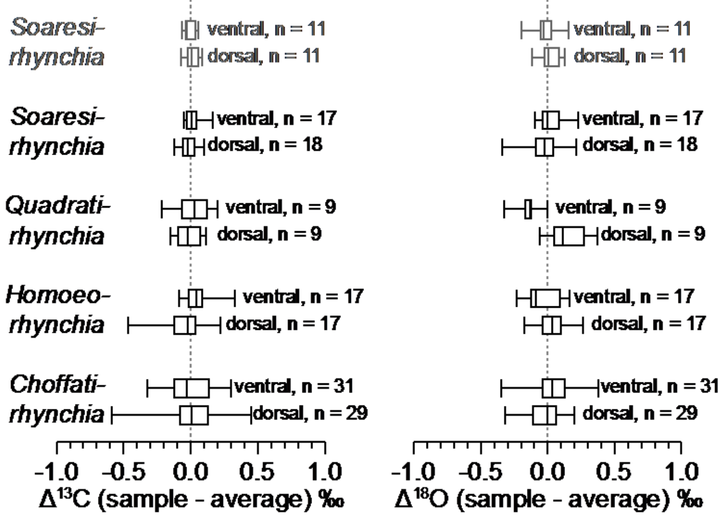

Figure S6: Whisker plot of differences of $\delta^{13}C$ and $\delta^{18}O$ values from specimen average depending on sampled valve. Whiskers indicate the entire observed range and boxes the second and third quartile with median. Only genera for which at least three specimens with four or more well-preserved samples were available are presented. Isotopic differences relating to the sampled valve are generally negligible. The dataset for *Soaresirhynchia* is split into specimens from Portugal (grey) and Spain (black).

Also average differences in Mg/Ca and Sr/Ca are minor with a maximum difference of median Mg/Ca of 0.3 mmol/mol and maximum difference of median Sr/Ca of 0.02 mmol/mol in *Quadratirhynchia* (**Fig. S7**). Consequently, no biases related to the valve chosen for sampling need to be considered.

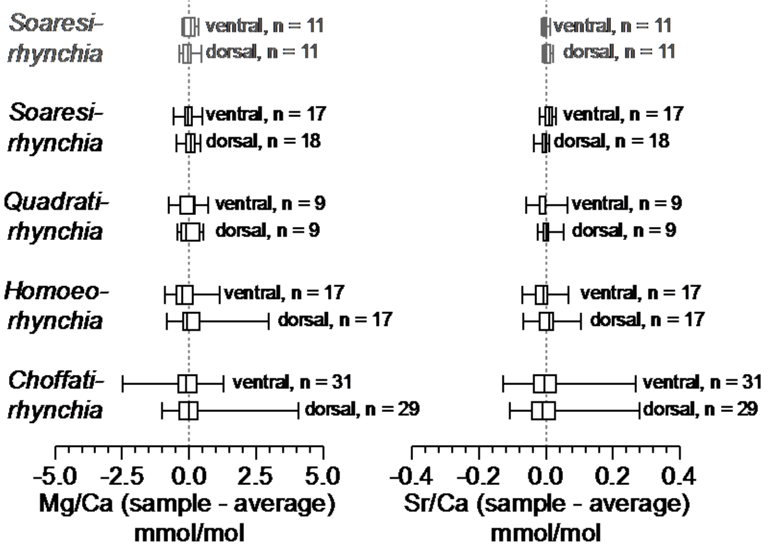

Figure S7: Whisker plot of differences of Mg/Ca and Sr/Ca ratios from specimen average depending on sampled valve. Whiskers indicate the entire observed range and boxes the second and third quartile with median. Only genera for which at least three specimens with four or more well-preserved samples were available are presented. Differences relating to the sampled valve are generally negligible. The dataset for *Soaresirhynchia* is split into specimens from Portugal (grey) and Spain (black).

The median isotopic variability within individuals of the various species has been computed from specimens for which at least four samples which were deemed well-preserved were available (**Table S5**). Median 2 sd isotopic variability is always smaller than 0.5 ‰ for both isotopic systems, but substantially larger in *Choffatirhynchia*, *Gibbirhynchia*, *Homoeorhynchia* and *Quadratirhynchia* than in *Cirpa*, *Nannirhynchia* and *Soaresirhynchia*.

Differences between fossils from the same stratigraphic level

Differences in geochemical proxies between individual brachiopod fossils were assessed for stratigraphic intervals for which at least two fossils yielding four well-preserved samples each were available. This is the case for *Choffatirhynchia*, *Homoeorhynchia*, *Quadratirhynchia* and *Soaresirhynchia* from Barranco de la Cañada and *Cirpa* from Fonte Coberta / Rabaçal. For most of the resulting fossil couples significant (95 % confidence) differences between average $\delta^{13}C$ values (10 of 16) and $\delta^{18}O$ values (12 of 16) were observed (**Fig. S8**).

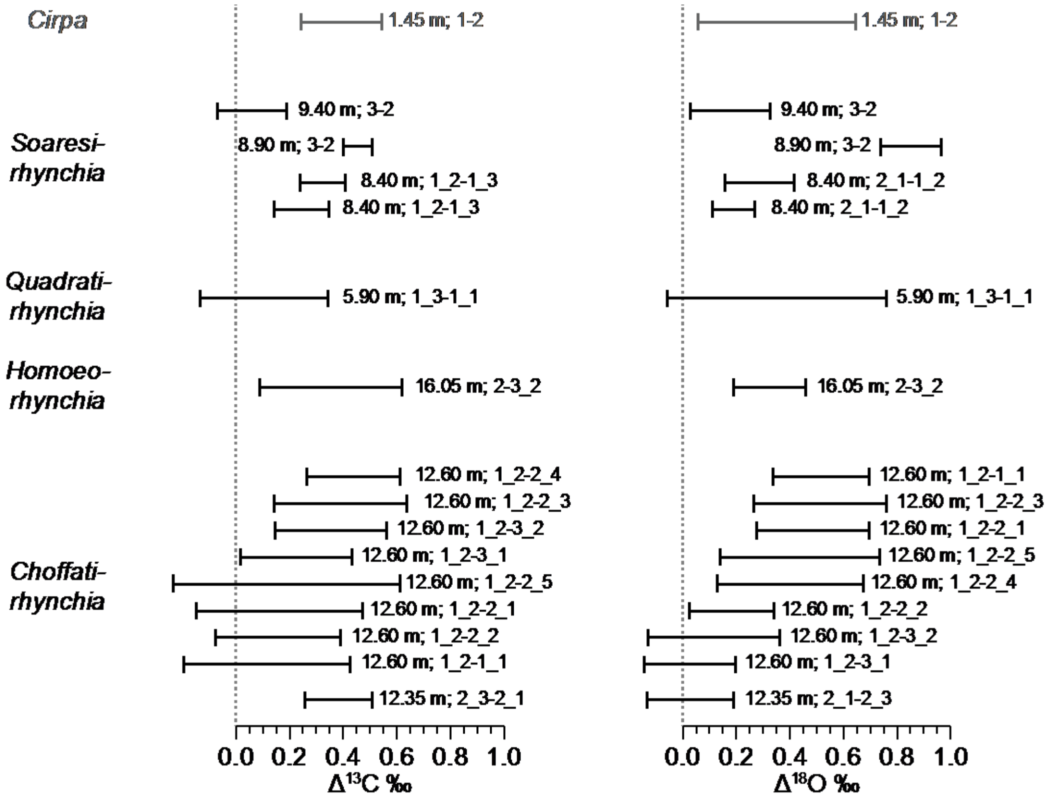

Figure S8: Isotopic differences between fossils from the same stratigraphic horizon. Error bars indicate 95 % confidence interval for the difference in average values. Where more than two specimens from a horizon are available, all are compared to the most ^{13}C and ^{18}O enriched specimen respectively.

Isotopic differences greater than 0.5 ‰ between different specimens, however, were not observed for $\delta^{13}C$ values and only in three instances for $\delta^{18}O$ values (0.51, 0.52, and 0.85 ‰). While stratigraphic horizons for which only one specimen or few specimens are available the computed isotopic averages are therefore less certain than were ample sample material was at hand, uncertainties relating from inter-specimen differences are not major.

Interspecific offsets amongst brachiopods

For most stratigraphic levels only one brachiopod genus was studied, but data for specimens of two brachiopod genera with four samples each are available for sample horizons BC3 (13.2 m; *Choffatirhynchia*, *Homoeorhynchia*) and BC4 (13.75 m; *Gibbirhynchia*; *Homoeorhynchia*) at Barranco de la Cañada. Differences in carbon (0.54 ‰; 0.34 ‰) and oxygen (0.49 ‰; 0.14 ‰) isotope ratios between these genera is comparable to that of individuals of the same genus attesting to the reliability of the measured isotope ratios.

Regarding their chemical composition, the studied rhynchonellid taxa fall into two distinct groups (**Table S6**). The species with comparatively larger isotopic variability (*Choffatirhynchia*, *Gibbirhynchia*, *Homoeorhynchia* and *Quadratirhynchia*) are characterised by comparatively low calcite contents of 97.6 to 99.1 % versus the in-house marble standard CAR and comparatively high Mg/Ca (4.8 to 6.6 mmol/mol) and Sr/Ca (0.95 to 1.14 mmol/mol) ratios. Species with low isotopic variability (*Cirpa*, *Nannirhynchia*, *Soaresirhynchia*) on the other hand have high calcite content of 99.9 to 100.3 % versus the in-house standard CAR and low Mg/Ca (1.9 to 2.6 mmol/mol) and Sr/Ca (0.51 to 0.54 mmol/mol) ratios.

Isotopic difference between rhynchonellids and Gryphaea

When comparing isotope ratios of brachiopods and bivalves from the Barranco de la Cañada section, a clear tendency of $\delta^{13}C$ values from *Gryphaea* to be more positive than brachiopods from the same horizon is observed (**Figure S9**).

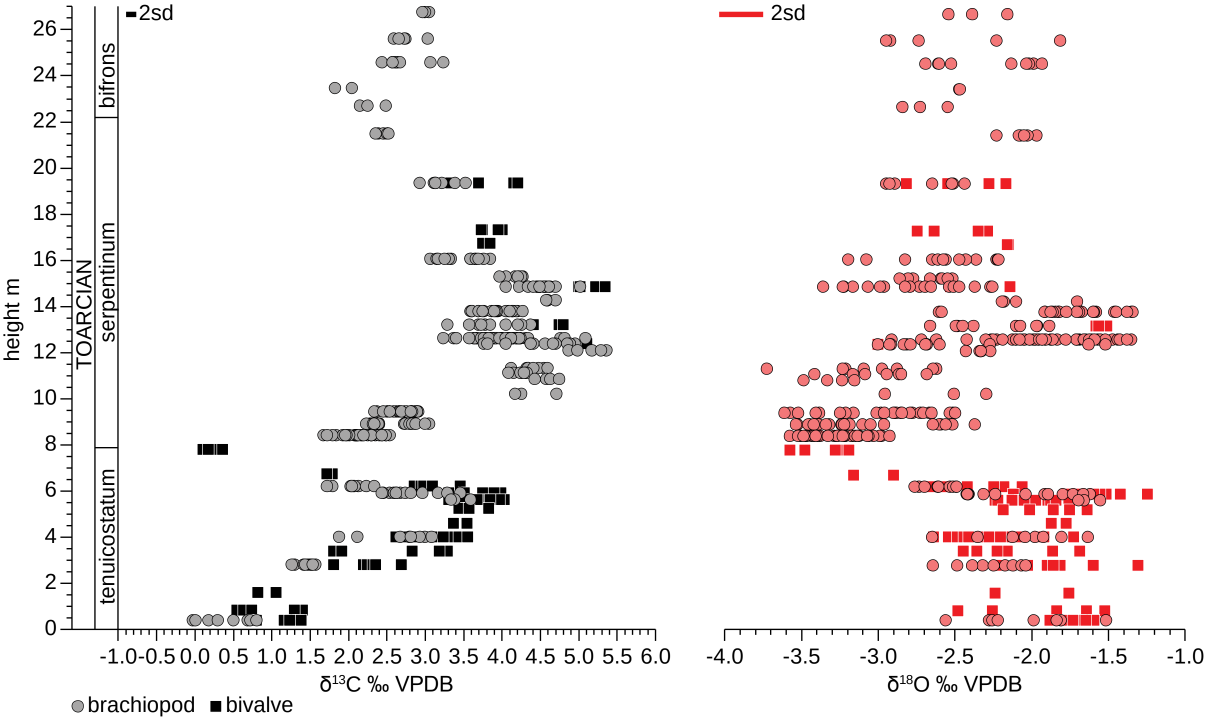

Figure S9: Carbon and oxygen isotope ratios for all individual samples from the Barranco de la Cañada section that passed screening for diagenesis.

For ten stratigraphic levels, samples for both, brachiopods and *Gryphaea*, were analysed. Comparison of the two datasets including overlaps with all rhynchonellids apart from *Soaresirhynchia* revealed a consistent offset of *Gryphaea* data towards more positive $\delta^{13}C$ values (+0.25 to +1.00 ‰), with a weighted average of +0.63 ‰. *Gryphaea* data were also found to be minimally enriched in ^{18}O (-0.43 to +0.70 ‰) with a weighted average of +0.10 ‰.

Independent studies reporting carbon and oxygen isotope ratios for coeval Early Jurassic brachiopods and *Gryphaea* are currently unavailable. The framework for understanding C & O isotope data from rhynchonellid brachiopods is very well established, their average shell preservation is superior to that of *Gryphaea* in the Barranco de la Cañada section, and rhynchonellids contribute more than 75 % of the data of the screened dataset. Futhermore, the dataset from Fonte Coberta / Rabaçal is based solely on brachiopod samples. It was therefore decided to shift all *Gryphaea* $\delta^{13}C$ values by -0.63 ‰ to generate a coherent dataset. The question remains how this recalculated dataset exactly matches to absolute atmospheric carbon isotope ratios during the Toarcian stage, but the correction procedure allows for relative changes in $\delta^{13}C$ values to be studied with confidence. $\delta^{18}O$ values of the *Gryphaea* samples were not adjusted, because the observed difference between the two fossil types of 0.10 ‰ was deemed negligible.

Calculation of palaeo seawater temperatures

A palaeothermometer based on oxygen isotope ratios and supplemented by $MgCO_3$ concentrations has been developed using modern analogues[32]. We adopt this thermometer as the currently most sophisticated thermometer available for brachiopods and use it also for the *Gryphaea* data owing to the good agreement of the brachiopod and bivalve datasets. The equation presented by Brand et al. (2013)[32] requires and input of seawater $\delta^{18}O$ and mol% $MgCO_3$ in the shell calcite. Due to the lack of robust knowledge of seawater $\delta^{18}O$ we use here the value of -1 ‰ vs V-SMOW and also list palaeotemperature data derived from the equation by Anderson and Arthur (1983)[39] to enable maximum comparability to palaeotemperature data of Suan et al., (2008)[33]. The fractionation factor for oxygen isotopes between calcite and water is also to some degree influenced by the magnesium concentration of the secreted calcite[40]. The magnitude of this effect is approximately 0.17 ‰ per mol% $MgCO_3$. The resulting MgCO3 effect on the fractionation factor is very small (0.07 ‰ on average) due to the very low $MgCO_3$ content of the shell materials averaging 0.4 mol% $MgCO_3$. Because of the narrow range of $MgCO_3$ concentrations in the studied shell materials palaeotemperatures are calculated for aggregated data rather than for each sample individually. The resulting potential biases are negligible, e.g., 0.07 ‰ and 0.24 °C or less between the different taxa.

Average palaeotemperatures for the Barranco de la Cañada section derived from Refs. 32 and 39 together with average carbon and oxygen isotope ratios and ages based on the Geologic Timescale 2012[41] are listed in **Table S7**.

Modelling

Simple mass balance calculations

The datapoints are separated by time intervals in the region of 0.05-0.1Myrs, shorter than the ~100 kyr residence time of oceanic carbon[42] and longer than the c. 1,000 year mixing time of the ocean. Over such timescales we can make rough estimates of how much carbon is needed to drive the perturbation using simple mass-balance calculations[43]. We write the initial isotopic composition of exchangeable carbon in the ocean-atmosphere pool as $\delta^{13}C_T$, then equate the mass of this pool M_T to the sum of its organic and inorganic components $M_T = M_{inorganic} + M_{organic}$. We can then write the pre-perturbation isotopic mass balance as:

$$\delta^{13}C_T \cdot M_T = \delta^{13}C_{organic} \cdot M_{organic} + \delta^{13}C_{inorganic} \cdot M_{inorganic} \qquad (1)$$

The values for present day the exchangeable dissolved organic and inorganic carbon pools are $M_{organic}$ = 662 Gt C, and $M_{inorganic}$ = 37,400 Gt C[44,45], which we use as a reference point for the pre-perturbation Jurassic system.

We write the post-perturbation composition as $\delta^{13}C_T'$, based on the idea that this value deviates from $\delta^{13}C_T$ as a function of the mass M_X and isotopic composition $\delta^{13}C_X$ of some external carbon pool (methane, large igneous province derived CO_2 etc) putatively responsible for the perturbation:

$$\delta^{13}C_T{}' = \frac{\delta^{13}C_T \cdot M_T + \delta^{13}C_X \cdot M_X}{M_T + M_X}, \therefore M_X = \frac{M_T(\delta^{13}C_T{}' - \delta^{13}C_T)}{\delta^{13}C_X - \delta^{13}C_T{}'} \qquad (2)$$

The right hand side of (2) illustrates how the more negative $\delta^{13}C_X$ (i.e. the isotopically lighter the carbon input) the lower M_X (i.e. the less carbon is needed to explain an excursion of a given magnitude). Sources of isotopically light carbon plausibly relevant to the Toarcian include CH_4 released by clathrate destabilization $\delta^{13}C_{CH_4\,hyd} = -60$ ‰[45,46] thermogenic CH_4 from contact metamorphism -35 ‰ $\leq \delta^{13}C_{CH_4\,therm} \leq -25$ ‰[47], CO_2 derived directly from magma $-7‰ \leq \delta^{13}C_{CO_2\,magma} = -5‰$ and/or thermogenic CO_2 sources associated with large igneous province emplacement $-27‰ \leq \delta^{13}C_{CO_2\,therm} \leq 2‰$[48].

The carbon isotopic dataset reaches a minimum of $\delta^{13}C$=–0.36 ‰ after a downward trend that begins from an inflection point at $\delta^{13}C$=3.13 ‰. Treating this as a single negative carbon isotopic excursion (NCIE) we calculate a magnitude of $\Delta\delta^{13}C_{data,NCIE} = -(3.13 - -0.36) = -3.49‰$. This happens within an interval of Δt_{NCIE} ~ 181.98-181.70=0.28Myrs[41]. The NCIE is followed by a large amplitude positive excursion

$\Delta\delta^{13}C_{data,PCIE} = 5.13 - -0.36 = 5.49‰$, which occurs over Δt_{NCIE} ~ 181.7 Ma – 181.3 Ma =0.4Myrs[41].

Writing $\delta^{13}C_T = \delta^{13}C_{data,pre} = 3.13$ and $\delta^{13}C_T{}' = \delta^{13}C_{data,pre} + \Delta\delta^{13}C_{data,NCIE} = -0.36$ we can solve (2) for M_X using various different values of $\delta^{13}C_X$ corresponding to the above sources, thus estimate the mass of different types of carbon plausibly responsible. This produces the results depicted in **Fig. S10** (see also **Table S8**).

The numbers for methane are in the same range as the Beerling et al. (2002)[49] upper estimate of a clathrate-derived methane induced excursion of c. 5,000 Gt C, as well as earlier estimates[44], considering a likely background pCO_2 of approximately 2-4 time pre-industrial values[50].

The dynamical modelling results referred to in the main text correspond, unless otherwise stated, to $\delta^{13}C_{CO_2} = \delta^{13}C_{CO_2\,magma} = -7‰$ and $\delta^{13}C_{CH_4} = \delta^{13}C_{CH_4\,hyd} = -60‰$. The above mass balance calculations neglect dynamical carbon sinks and are consequently underestimates. The input fluxes and the time interval over which these fluxes were non-zero were tuned so as to reproduce the data:

$$TCO_{2\,(input)} = \int_{t_{start\,CO_2}}^{t_{end\,CO_2}} F_{Toarcian\,(CO_2)} dt \tag{3}$$

$$TCH_{4\,(input)} = \int_{t_{start\,CH_4}}^{t_{end\,CH_4}} F_{Toarcian\,(CO_2)} dt \tag{4}$$

With $F_{Toarcian\,(CO_2)} = 3.6 \times 10^{13} molyr^{-1}$ and $F_{Toarcian\,(CH_4)} = 1.107 \times 10^{12} molyr^{-1}$ with the time of the start and end of the CO_2 "burn" $t_{start\,CO_2} = 181.87$ Ma,$t_{end\,CO_2} = 181.74$ Ma and the time of methane input $t_{start\,CH_4} = 181.75$ Ma $t_{end\,CH_4} = 181.65$ Ma.

This gives the total time-integrated carbon inputs to the dynamical model described below is an LIP- CO_2 input $F_{Toarcian\,(CO_2)} \cdot (0.13 \times 10^6 yrs) = 5.25 \times 10^{18} mol$, or $F_{Toarcian\,(CO_2)} \cdot (0.13 \times 10^6 yrs) \cdot \frac{12.0107}{1\times10^{15}} = 63146$ GtC as LIP-associated CO_2 , and $F_{Toarcian\,(CH_4)} \cdot (0.01 \times 10^6 yrs) = 1.107 \times 10^{17} mol$ or $F_{Toarcian\,(CH_4)} \cdot (0.01 \times 10^6 yrs) \cdot \frac{12.0107}{1\times10^{15}} = 1329$ GtC as clathrate methane.

A coarse estimate of how much carbon would be required in order to explain the observed PCIE can be derived by modifying (1) to describe the removal of mass M_X, i.e. $\delta^{13}C_T{}'(M_T - M_X) = \delta^{13}C_T \cdot M_T - \delta^{13}C_X \cdot M_X$, thus $M_X = \frac{M_T(\delta^{13}C_T{}' - \delta^{13}C_T)}{(\delta^{13}C_T{}' - \delta^{13}C_X)}$. Writing $\delta^{13}C_X = \delta^{13}C_{organic}$, then equating the pre- and post- perturbation isotopic compositions to $\delta^{13}C_T = -0.36$, and $\delta^{13}C_T{}' = 5.13$, and the duration of the PCIE from the data

$\Delta t_{data,\ PCIE} = 0.4\ Myrs$. A provisional estimate of the necessary organic carbon burial flux can be provisionally derived using an intermediate value for the organic carbon isotopic composition $\delta^{13}C_{organic} = -24.5‰$ (An expanded range $-32.5‰ \leq \delta^{13}C_{organic} \leq -17.5‰$[51] is explored in the figure below (**Fig. S10**)). These numbers (in GtC) can be related to the present day organic carbon burial flux $mocb_0 = 3.75 \times 10^{12} molyr^{-1}$ [52] via $GtC\ (organic, out) = x \cdot \int_0^{\Delta t_{data,\ PCIE}} mocb_0 dt$ where x is the magnitude of the organic carbon burial flux relative to present. Results of these calculations are listed in **Table S8**.

However, these are underestimates because the mass balance calculation in (2) neglects both dynamical carbon sinks and the fact the positive excursion occurs after the carbon input corresponding to the negative excursion, which will increase the baseline reservoir size M_T. Consequently we now turn to a dynamical modelling approach.

Proxy inversion modelling

Our inversion model aims to get the maximum amount of information from the dataset whilst making the minimum possible number of assumptions. We proceed by back-calculating CO_2 and temperature via the $\delta^{18}O$ data, then estimating carbon cycle fluxes compatible with both these calculations and the $\delta^{13}C$ data:

1. The $\delta^{18}O$ dataset is related to global average surface temperature using an existing empirical formulation[32] (see below).
2. This $\delta^{18}O$-inversion temperature $T_{\delta^{18}O}$ is used to estimate a corresponding value for relative atmospheric CO_2, $R_{CO_2\ (\delta^{18}O)}$ using the Geocarbsulf model's temperature function.
3. Estimates of the normalized magnitude of uplift U, degassing D and bulk weathering W are taken from the forcings for the COPSE model[53] across the time interval corresponding to the dataset – allowing us to estimate the various weathering fluxes relevant to the organic and carbonate carbon cycle mass balances.
4. The marine carbonate carbon burial flux $mccb$ is assumed to be calculable from mass balance with the silicate and carbonate weathering fluxes (or, in an alternative scenario, the size and distribution of the atmosphere ocean CO_2 reservoir).
5. The combination of all the above flux estimates, the $\delta^{13}C$ data, and isotopic mass balance, allows us to estimate the marine organic carbon burial $mocb$ flux.
6. The marine phosphate concentration $\bar{P}$ corresponding to this marine organic carbon burial flux, along with a rough estimate of the corresponding global ocean anoxic fraction f_{anox} is then calculated.

Thus, our aim in this section is to describe an approximate mapping from each $\delta^{13}C, \delta^{18}O$ datapoint to temperature, carbon cycle weathering/burial fluxes, marine limiting nutrient concentration, and finally anoxia.

We begin with Berner's global carbon isotopic mass balance[54], which is an isotopically weighted mass balance combining the inputs to and outputs from the organic and carbonate carbon cycles (please see **Table S9** for flux abbreviations):

$$\delta^{13}C_C \cdot (carbw + ccdeg) + \delta^{13}C_G \cdot (oxidw + ocdeg) + \delta^{13}C_{\Delta toarcian} \cdot F_{\Delta toarcian}$$
$$= \delta^{13}C_{mccb} \cdot mccb + (\delta^{13}C_{mccb} - \varepsilon) \cdot mocb \quad (5)$$

The isotopic composition $\delta^{13}C_{mccb}$ of the marine carbonate carbon burial (mccb) flux is assumed to reflect the isotopic composition of the marine DIC pool and is given by:

$$\delta^{13}C_{mccb} = \frac{\delta^{13}C_C \cdot (carbw + ccdeg) + \delta^{13}C_G \cdot (oxidw + ocdeg) + \varepsilon \cdot mocb + \delta^{13}C_{\Delta toarcian} \cdot F_{\Delta toarcian}}{mocb + mccb} \quad (6)$$

Where $\delta^{13}C_{\Delta toarcian} \cdot F_{\Delta toarcian}$ is the isotopically weighted input from the putative Toarcian-specific perturbation, i.e. the sum of large igneous province (LIP) associated CO_2 degassing and CO_2 associated with the oxidation of clathrate-derived CH_4:

$$\delta^{13}C_{\Delta toarcian} \cdot F_{\Delta toarcian} = \delta^{13}C_{LIP} \cdot F_{LIP} + \delta^{13}C_{CH_4} \cdot F_{CH_4} \quad (7)$$

(Note that the above is set to zero by default for the figure discussed in the main text, because in this exercise we treat each $\delta^{13}C$ data point as reflecting a mass balance within which the above fluxes have already gone in to the atmospheric CO_2 pool, and thus drive the temperature increase). The $\delta^{18}O$ data is related to the inversion model temperature estimate using the fit of Brand et al (2013)[32], which incorporates the impact on $\delta^{18}O$ of the proportion of $MgCO_3$ in the shell:

$$T_{\delta^{18}O\,(model)} = 16.192 - 3.468 \cdot (\delta^{18}O_{data} - \delta^{18}O_{SW} - \delta_{Mg}) \quad (8)$$

Where we assume, in line with previous estimates, that the average seawater isotopic composition can be approximated by $\delta^{18}O_{SW} = -1‰$, and the magnesium calcite adjustment factor $\delta_{Mg} = 0.17\, mol\%MgCO_3$ [14], was for this dataset approximately $mol\%\, MgCO_3 = 0.4$. For comparison, we additionally show Andersen & Arthur's[39] fit, which does not include a magnesium calcite correction:

$$T_{\delta^{18}O\,(reference)} = 16.0 - 4.14 \cdot \delta^{18}O_{data} + 0.13 \cdot ({\delta^{18}O_{data}}^2) \quad (9)$$

We equate (9) to the Geocarbsulf's temperature function:

$$T_{\delta^{18}O} - T_0 = ESS \cdot \frac{\ln\left(\frac{CO_{2\,(atmos)}}{CO_{2\,0}}\right)}{\ln(2)} - W_s \left(\frac{t_{bp}}{570}\right) + GEOG \quad (10)$$

Where the baseline modern temperature $T_0 = 15°C$, $ESS = 5$ is the "Earth system sensitivity" (long term climate sensitivity), $GEOG = 3$ (corresponding to the early Jurassic) is Royer's adjustment to Berner's original model to express the sensitivity of temperature to changes in paleogeography[55], $W_s = 7.4$ represents Berner's linearization

of the luminosity component of the temperature function[56], atmospheric CO_2 is normalized to pre-industrial levels of $CO_{2\,0} = 280$ ppm and t_{bp} is the model time in millions of years before present. This allows us to derive an estimate of the relative atmospheric $R_{CO_2} = \frac{CO_{2\,(atmos)}}{CO_{2\,0}}$ corresponding to each inversion temperature.

$$R_{CO_2\,(\delta^{18}O)} = \frac{CO_{2\,(atmos)}}{CO_{2\,0}} = e^{\left(T_{\delta^{18}O} - T_0 - GEOG + W_s\left(\frac{t_{bp}}{570}\right)\right)\cdot\frac{\ln(2)}{ESS}} \quad (11)$$

Several of the fluxes in (5) are plausibly a function of the relative size of the carbonate and organic carbon rock reservoirs. The differential equations describing the mass of carbonate C and organic G carbon are estimated by simple differential equations of the form given by Berner's original Geocarb model:

$$\frac{dC}{dt} = mccb - carbw - ccdeg \quad (12)$$

$$\frac{dG}{dt} = mocb - oxidw - ocdeg \quad (13)$$

Degassing fluxes are constrained by time-dependent forcings taken from the COPSE model[53]:

$$ccdeg = ccdeg_0 \cdot D_{(t_{bp})} \quad (14)$$

$$ocdeg = ocdeg_0 \cdot D_{(t_{bp})} \quad (15)$$

Where $D_{(t_{bp})}$ is a normalized estimate of the time-specific degassing rate. We estimate the carbonate $carbw$ and oxidative $oxidw$ weathering fluxes via:

$$carbw = carbw_0 \cdot U_{(t_{bp})}^{0.9} \cdot (1 + 0.087 \cdot \Delta T) \cdot 2 \cdot \left(\frac{R_{CO_2\,(\delta^{18}O)}}{1 + R_{CO_2\,(\delta^{18}O)}}\right) \cdot \frac{C_{(t)}}{C_0} \quad (16)$$

$$oxidw = oxidw_0 \cdot U_{(t_{bp})} \cdot \frac{G_{(t)}}{G_0} \quad (17)$$

Where $\Delta T = T_{\delta^{18}O} - T_0$ is the deviation between the temperature estimated from the proxy inversion and the baseline planetary average. (We also experimented with an adjustment for the bias in global weathering to tropical latitudes $\Delta T_{tropics} = \frac{2}{3}\Delta T$[5], but this did not qualitatively alter the results).

We explored two options for the marine carbonate carbon burial flux $mccb$. The default carbonate carbon burial flux was assumed to balance carbonate and silicate weathering:

$$mccb_{balance} = carbw + silw \quad (18)$$

The kinetic-limited silicate weathering flux scales with temperature and CO_2 via Caldeira and Kasting (1992)[57]:

$$silw_{kinetic} = silw_0 \cdot U_{(t_{bp})}^{0.33} \cdot e^{0.09\Delta T} \cdot (1 + 0.038 \cdot \Delta T)^{0.65} \cdot \sqrt{R_{CO_2\,(\delta^{18}O)}} \quad (19)$$

Limitation of silicate weathering by the supply, via physical erosion, of silicate cations in weather-able rock is imposed using a formulation from previous deep time work[58]:

$$silw_{supply} = silw_{kinetic} + \frac{silw_{max} - silw_{kinetic}}{1+e^{-k_{supply}(silw_{kinetic}-silw_{max})}} \qquad (20)$$

Where $silw_{max} = 3 \cdot silw_0$ and $k_{supply} = 100$, such that the formulation in (20) dictates the manner in which $silw_{supply}$ plateaus out at $silw_{max}$ at high weathering rates. In the simulations discussed in the main text we imposed supply limitation of silicate weathering at lower thresholds of 1.5 to 2 times the modern value.

An alternative functional form of the marine carbonate carbon burial flux involves a coarse approximation to its (short-term) dependence on carbonate alkalinity:

$$mccb_{variable} = mccb_0 \cdot RCO_{2\,(ocean)} \qquad (21)$$

Where $RCO_{2\,(ocean)}$ is the normalized size of the marine component of the global ocean-atmosphere CO_2 reservoir. In keeping with the revised COPSE model[59] and previous calculations[51], we assume that relative atmospheric CO_2 scales with the second power of the normalized reservoir size:

$$R_{CO_2\,(\delta^{18}O)} = \left(\frac{A_{(t)}}{A_0}\right)^2, \therefore A_{(t)} = A_0 \cdot \sqrt{R_{CO_2\,(\delta^{18}O)}} \qquad (22)$$

This allows us to estimate the atmospheric fraction ϕ of the total ocean-atmosphere CO_2 reservoir (assuming a constant total number of moles of air in the atmosphere of $mol_{atmos} = 1.773 \times 10^{20}$):

$$\phi_{(t)} = \frac{R_{CO_2\,(\delta^{18}O)} \cdot CO_{2\,0} \cdot 1\times10^{-6} \cdot mol_{atmos}}{A_0 \cdot \sqrt{R_{CO_2\,(\delta^{18}O)}}} \qquad (23)$$

Which finally gives the ocean component of the CO_2 reservoir, scaled relative to present day values:

$$RCO_{2\,(ocean)(t)} = \frac{(1-\phi_{(t)})A_{(t)}}{(1-\phi_0)A_0} \qquad (24)$$

This is then plugged into the $mccb_{variable}$ flux (21).

The only remaining undetermined flux, marine organic carbon burial, can now be calculated by assuming $\delta^{13}C_{mccb} = \delta^{13}C_{data}$ and solving (5) for this flux:

$$mocb = \frac{\delta^{13}C_C \cdot (carbw + ccdeg) + \delta^{13}C_G \cdot (oxidw + ocdeg) + \delta^{13}C_{\Delta toarcian} \cdot F_{\Delta toarcian} - \delta^{13}C_{mccb} \cdot mccb}{(\delta^{13}C_{mccb} - \varepsilon)} \qquad (25)$$

We initialize at the first time step using isotopic compositions for carbonate $\delta^{13}C_C = 0‰$ and organic carbon in rock $\delta^{13}C_G = -\varepsilon = -24.5$ and modern day reservoir sizes C_0 and G_0, then proceed to use the dataset to solve the differential equations (24) and (25) by

Euler's method. Berner's original equations for the change over time in the isotopic composition of the organic and carbonate rock reservoirs were also used:

$$\frac{d\delta_C C}{dt} = \delta_{mccb} \cdot mccb - \delta_C \cdot carbw - \delta_C \cdot ccdeg \qquad (26)$$

$$\frac{d\delta_G G}{dt} = (\delta_{mccb} - \varepsilon) \cdot mocb - \delta_G \cdot oxidw - \delta_G \cdot ocdeg \qquad (27)$$

For each estimate of marine organic carbon burial $mocb$ that we arrive at (i.e. at each timestep), we calculate the corresponding relative concentration of limiting nutrient necessary to sustain it:

$$mocb = mocb_0 \cdot \bar{P}^2, \therefore \bar{P} = \frac{PO_4}{PO_{40}} = \sqrt{\frac{mocb}{mocb_0}} \qquad (28)$$

The global phosphorous weathering flux is taken from COPSE, and is a function of temperature, CO_2 and the other weathering fluxes:

$$phosw = phosw_0(f_{silw}\frac{silw}{silw_0} + f_{carbw}\frac{carbw}{carbw_0} + f_{oxidw}\frac{oxidw}{oxidw_0}) \qquad (29)$$

Where we use recently revised apportioning factors are $f_{silw} = 0.58$, $f_{carbw} = 0.21$, $f_{oxidw} = 0.21$. The formulations of the calcium-phosphate burial flux $capb$, iron adsorbed phosphate burial flux $fepb$ and marine organic phosphate $mopb$ are given by:

$$capb = capb_0 \cdot \bar{P}^2 \qquad (30)$$

$$fepb = fepb_0(1 - f_{anox}) \qquad (31)$$

$$mopb = mocb(\frac{f_{anox}}{CP_{anox}} + \frac{1-f_{anox}}{CP_{ox}}) \qquad (32)$$

We make the (highly idealized/approximate) simplifying assumption that the global phosphate reservoir's dynamics are at roughly steady state at each carbon isotope data point:

$$\frac{d\bar{P}}{dt} = phosw - capb - fepb - mopb \approx 0 \qquad (33)$$

This assumption allows us to estimate the global ocean anoxic fraction f_{anox} by substituting (35)-(38) into (39):

$$f_{anox} = \frac{phosw - capb - fepb_0 - \frac{mocb}{CP_{ox}}}{mocb\left(\frac{1}{CP_{anox}} - \frac{1}{CP_{ox}}\right) - fepb_0} \qquad (34)$$

Where we additionally impose the necessary constraint that $0 \leq f_{anox} \leq 1$.

To sum up, we have used the $\delta^{18}O$ data to produce a temperature (thus CO_2 greenhouse) estimate, then plugged this estimate and the $\delta^{13}C_{data}$ into an isotopically weighted carbon cycle mass balance in order to estimate marine organic carbon burial, thus

limiting nutrient concentration, thus anoxia. The results of this proxy inversion exercise are discussed in the main text. Discrete values taken for modelling are listed in **Table S9**.

Forward modelling in the COPSE (Carbon, Oxygen, Phosphorous, Sulphur, Evolution) model

In the forward model we aim to reproduce the data using prescribed greenhouse forcings, as opposed to using this data to drive the model. Full model equations for COPSE can be found in the original descriptions[53,59]; we restrict our description here to those fluxes of key conceptual relevance. The greenhouse forcings dictating the injection of LIP-CO_2 and clathrate CH_4 are given by (also see equations (3) and (4) above):

$$F_{LIP\ CO_2(t_{LIP\ start} \geq |t_{bp}| \geq t_{LIP\ end})} = F_{Toarcian\ (CO_2)0},\ F_{LIP\ CO_2\left(\neg(t_{LIP\ start} \geq |t_{bp}| \geq t_{LIP\ end})\right)} = 0 \quad (35)$$

$$F_{Clathrate\ CH_4(t_{CH_4\ start} \geq |t_{bp}| \geq t_{CH_4\ end})} = F_{Toarcian\ (CH_4)0}$$

$$F_{Clathrate\ CH_4\left(\neg(t_{CH_4\ start} \geq |t_{bp}| \geq t_{CH_4\ end})\right)} = 0 \quad (36)$$

Where t_{bp} is model time in millions of years before present and $t_{LIP\ start}$, $t_{LIP\ end}$ $t_{CH_4\ start}$, $t_{CH_4\ end}$ are tuneable parameters describing, respectively, the time (in years before present) at which LIP-CO_2 and clathrate CH_4 begin and cease. Additional forcings are degassing D, uplift U, weathering W, and relative biotic terrestrial evolution E, from the original model.

As above, the δ_{mccb} value predicted by the model is assumed equilibrated with the isotopic composition of the global ocean-atmosphere CO_2 reservoir A, $\delta_{mccb} = \delta_A$. The mass and isotopic composition of the global ocean-atmosphere CO_2 reservoir changes over time according to:

$$\frac{dA}{dt} = ocdeg + ccdeg + oxidw + carbw - mccb - mocb - tocb - sfw + F_{LIP\ CO_2} + F_{Clathrate\ CH_4} \quad (37)$$

$$\frac{d(\delta_A A)}{dt} = \delta_G(ocdeg + oxidw) + \delta_C(ccdeg + carbw) - \delta_A mccb - (\delta_A - \varepsilon_{ocean})mocb - (\delta_A - \varepsilon_{land})tocb + \delta^{13}C_{CH_4}F_{Clathrate\ CH_4} + \delta^{13}C_{LIP}F_{LIP\ CO_2} \quad (38)$$

Where the isotopic composition of the organic δ_G and carbonate δ_C rock reservoirs change according to (26) and (27). The terrestrial organic carbon burial flux *tocb* is related to phosphorous weathering of the terrestrial rock surface:

$$tocb = 0.02041 \cdot V \cdot CP_{land} \cdot phosw \quad (39)$$

Where $CP_{land} = 1000$. The relative mass of the terrestrial photosynthetic biosphere V exhibits a simple Michaelis-Menten style dependence on atmospheric CO_2, a dependence on temperature relative to a presumed maximum productivity at $T_{P_{max}} = 25°C$, and is inhibited by excessive oxygen levels in a way the represents photorespiration and fire:

$$V = k_{npp} \cdot E_{(t_{bp})} \cdot (1.5 - 0.5 \cdot \bar{O}) \cdot \left(1 - \left(\frac{T - T_{P_{max}}}{T_{P_{max}}}\right)^2\right) \cdot \left(\frac{P_{CO_2} - P_{min}}{P_{\frac{1}{2}} + P_{CO_2} - P_{min}}\right) \cdot \left(\frac{k_{fire}}{k_{fire} - 1 + ignit}\right)$$

(40)

Where $k_{npp} = 2$ is a normalizing constant, $E_{(t_{bp})} = 1$ is the terrestrial evolutionary forcing corresponding to the Jurassic, $\bar{O} = \frac{O_2}{O_{2\,0}}$ is the normalized global oxygen reservoir size, $P_{CO_2} = R_{CO_2} \cdot CO_{2\,0}$ is the atmospheric CO_2 mixing ratio in parts per million, $P_{min} = 10\,ppm$ and $P_{\frac{1}{2}} = 183.6\,ppm$ express the dependence of primary production on P_{CO_2}, $k_{fire} = 3$ expresses a 50% suppression of vegetation today relative to a world with no fire[60], and $ignit$ is a measure of the magnitude of the negative impact of fires on terrestrial production as oxygen's mixing ratio increases, given by Lenton et al (2018)[59]:

$$ignit = MIN\left[MAX\left[48 \cdot \left(\frac{\bar{O}}{\bar{O} + 3.762}\right) - 9.08,\ 0\right],\ 5\right] \quad (41)$$

Removal of CO_2 at the deep ocean crust via seafloor "weathering" sfw is assumed to exhibit a kinetic dependence on temperature comparable to terrestrial basalt weathering:

$$sfw = sfw_0 \cdot k_{sfw} \cdot D_{(t_{bp})} \cdot e^{k_T sfw \Delta T} \quad (42)$$

Where $sfw_0 = 3 \times 10^{12} mol yr^{-1}$, $k_{sfw} = 0.068$, $k_T sfw = 0.1332$. Terrestrial silicate and carbonate weathering fluxes are enhanced by the biosphere via V using the formulation from the original COPSE model[53]:

$$f_{Tsilw} = e^{0.09\Delta T} \cdot (1 + 0.038\Delta T)^{0.65} \quad (43)$$

$$f_{CO_2\,silw} = \sqrt{R_{CO_2}}\left(1 - MIN\left[V \cdot W_{(t_{bp})}, 1\right]\right) + \left(\frac{2 \cdot R_{CO_2}}{R_{CO_2} + 1}\right)^{0.4} \cdot MIN\left[V \cdot W_{(t_{bp})}, 1\right] \quad (44)$$

$$silw = silw_0 \cdot U_{(t_{bp})} \cdot f_{Tsilw} \cdot f_{CO_2\,silw} \cdot \left(k_{plants} + \left(1 - k_{plants}\right) \cdot MIN\left[V \cdot W_{(t_{bp})}, 1\right]\right) \quad (45)$$

Similarly for the carbonate weathering flux:

$$g_{Tcarbw} = 1 + 0.087\Delta T \quad (46)$$

$$g_{CO_2\,carbw} = f_{CO_2\,silw} \quad (47)$$

$$carbw = carbw_0 \cdot U_{(t_{bp})} \cdot g_{Tcarbw} \cdot g_{CO_2\,carbw} \cdot \left(k_{plants} + (1 - k_{plants}) \cdot MIN\left[V \cdot W_{(t_{bp})}, 1\right]\right) \quad (48)$$

The time derivative for the global oxygen reservoir is identical to that of the original model, except for the addition of an oxygen sink for those time steps at which the CH_4 input is non-zero (at which points we assume complete and instantaneous oxidation to CO_2 via stoichiometry $CH_4 + 2O_2 \rightarrow CO_2 + 2H_2O$):

$$\frac{dO}{dt} = mocb + tocb - oxidw + 2 \cdot (mpsb - pyrw - pyrdeg) - 2 \cdot F_{Clathrate\,CH_4} \quad (49)$$

Where the other fluxes are (left to right) marine organic carbon burial, terrestrial organic carbon burial, oxidative weathering, marine pyrite sulphur burial, pyrite weathering, pyrite degassing (see **Table S9** for a full list of abbreviations). The formulation of each of these fluxes is:

$$mocb = mocb_0(\bar{P}^2) \cdot \alpha e^{-\beta O_{2\,conc}} \quad (50)$$

For $\alpha = 2.217$, $\beta = 2277$, and $O_{2\,conc}$ is an estimate of the oxygen concentration in surficial seawater corresponding to the global reservoir size $O_{2\,conc} = \bar{O} \cdot O_{2\,conc\,0}$, where $O_{2\,conc\,0} = 331.5\ \mu molkg^{-1}$. The sulphur cycle is a key component of the Earth system's response to global anoxic events, including the Toarcian[61]. The relationship between burial of marine pyrite, that of marine organic carbon and the oxygen/sulphur marine reservoirs, is written as:

$$mpsb_{revised} = mocb \cdot \left(\frac{f_{anox}}{C{:}S_{anox}} + \frac{1 - f_{anox}}{C{:}S_{ox}}\right) \quad (51)$$

Where $C{:}S_{anox} = 2$, $C{:}S_{ox} = 3.5$[32]. The weathering and degassing of pyrite in rock is described by:

$$pyrw = pyrw_0 \cdot U_{(t_{bp})} \cdot \frac{PYR}{PYR_0} \quad (52)$$

$$pyrdeg = pyrdeg_0 \cdot D_{(t_{bp})} \cdot \frac{PYR}{PYR_0} \quad (53)$$

The pyrite rock reservoir PYR changes according $\frac{dpyr}{dt} = pyrw + pyrdeg - mpsb$ as described in the original model.

Elaboration on modelling results

Mass balance calculations

A simple estimate of the mass of ^{13}C-depleted carbon that must be introduced into the exchangeable ocean-atmosphere carbon pool in order to drive the observed negative CIE can be derived from simple mass balance equations (see supplement, model description section 1[42]). **Figure S10** depicts the requisite quantity of methane carbon derived from clathrate-decomposition ($\delta^{13}C_{CH_4\,hyd} = -60\,‰$)[46] or contact metamorphism ($\delta^{13}C_{CH_4\,therm} \leq -25\,‰$)[47], as well as that of CO_2 carbon derived directly from magma $-7‰ \leq \delta^{13}C_{CO_2\,magma} = -5‰$ [62] or thermogenic sources $-27‰ \leq \delta^{13}C_{CO_2\,therm} \leq 5‰$[47]. Additionally depicted for reference is the quantity of organic carbon (assuming $-32.5‰ \leq \delta^{13}C_{organic} \leq -17.5‰$, e.g. Ref. 38) that would need to be removed from the exchangeable pool (i.e. buried) in order to explain the positive isotopic excursion (i.e. the recovery from the negative CIE). However in this latter case it is more realistic to conceive of the carbon burial increase as a continuous dynamical change than the discrete perturbation represented by these calculations.

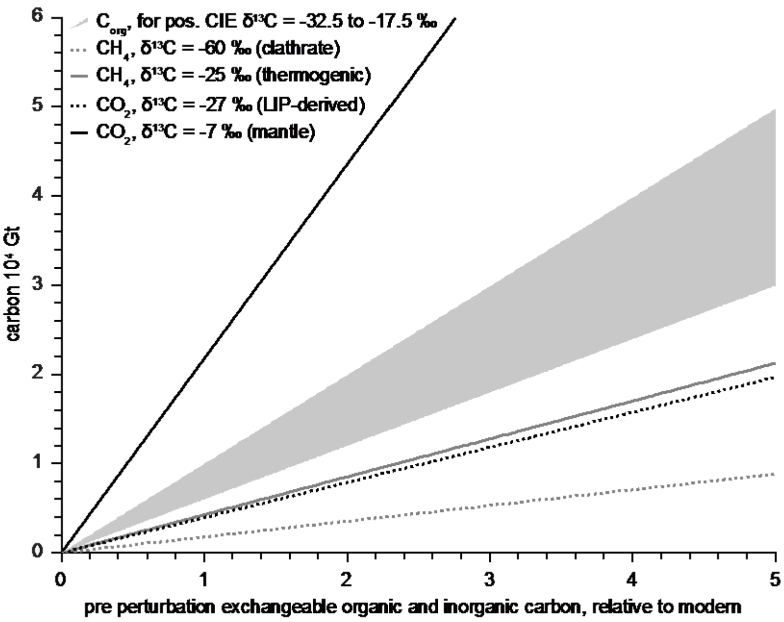

Figure S10: Simple mass balance calculations depicting the quantity of methane (grey) and CO_2 (black) needed to drive the observed negative carbon isotopic excursion, and the amount of additional organic carbon burial (grey field) necessary to drive the observed positive carbon isotopic excursion, assuming different pre-perturbation sizes of the combined organic/inorganic ocean-atmosphere exchangeable carbon pool relative to present.

Proxy Inversion model

The results of proxy inversion modelling are depicted in **Figure S11**. The inversion temperature $T_{\delta^{18}O}$ is compatible with a hyperthermal interval and supports a $\sim 2-4$°C increase precisely in line with the negative CIE, strongly supporting the case for methane induced warming, in the context of a prolonged, baseline hyperthermal state.

The organic carbon burial "*f*" ratio positively covaries with $\delta^{13}C$, reaching a lower limit at the most negative point of the CIE. This is based on the assumption that the organic carbon burial flux is in equilibrium with a carbonate burial flux, which in turn proportionally increases in line with the CO_2/CH_4 input, which is, in turn, tracked by the negative CIE (see model description, equation (10)). However, as $\delta^{13}C$ approaches zero it is highly likely that the system deviates from steady state- with the implication that the "*f*" ratio estimates at this point are loose approximations only. All other factors being equal, high marine organic carbon burial necessitates both high limiting nutrient concentration (to support the production) and translates into high ocean anoxia (due to the increased oxygen consumption as the organic carbon is remineralized).

The marine carbonate carbon burial flux is generally assumed to balance silicate and carbonate weathering over timescales of the order of that represented by the dataset (e.g. Ref. 51); which implicitly assumes continuous carbonate supersaturation in the ocean. We (simplistically) represent a scenario in which this assumption is relaxed (e.g. due to short term changes in marine carbonate alkalinity, and/or insufficient time for carbonate burial to equilibrate with increased weathering), via a formulation in which marine carbonate carbon burial scales with total ocean inorganic carbon (see description). This translates into a lower carbonate burial flux (and correspondingly higher organic flux in order to sustain mass balance).

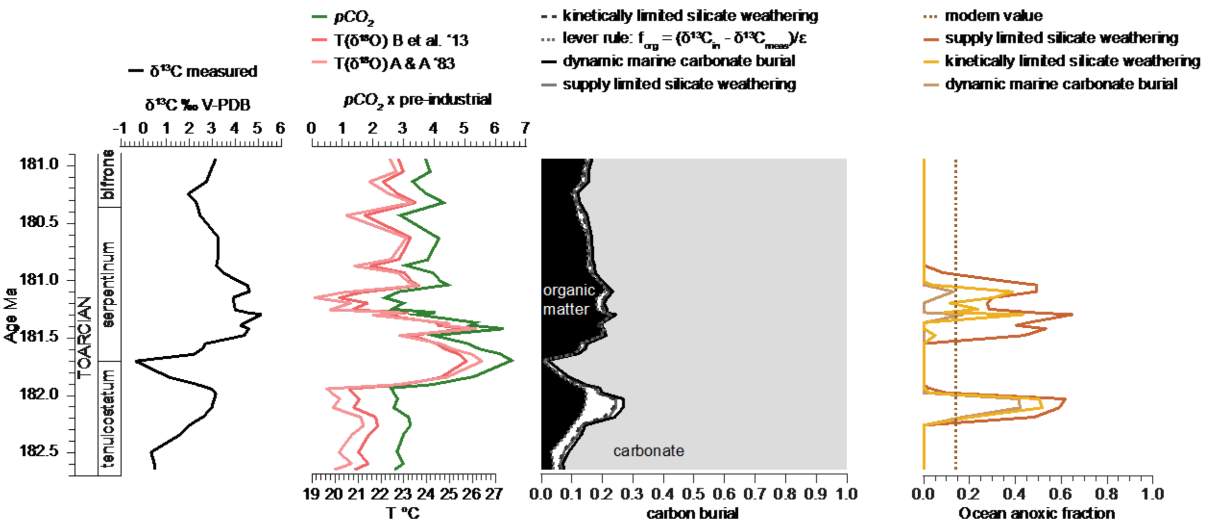

Figure S11: Results of a Geocarb-style inversion model in which the data is used to directly infer temperature, CO_2, carbon cycle fluxes and anoxia. $\delta^{18}O$ (red dotted line) and $\delta^{13}C$ (blue dotted line) data, temperature inversions (yellow and purple lines) corresponding to the former, and the corresponding relative atmospheric CO_2 level (green line) calculated from the selected (yellow) temperature, using Geocarbsulf's temperature function. The organic carbon burial "f" ratios $f_{org} = \frac{mocb}{mocb+mccb}$ calculated from the model temperature and CO_2 and isotopic mass balance (model description, equations (10) and (31)) under different scenarios for the other carbon cycle fluxes with which marine organic carbon burial is in mass balance. Grey solid line shows a supply (i.e. rock substrate) limited silicate weathering flux, dashed black line a kinetically (i.e. CO_2, temperature) flux, black solid line a scenario in which the marine carbonate carbon burial flux scales with the relative size of the marine inorganic carbon pool (rather than balancing weathering as in the other scenarios). Dashed grey line shows a "traditional" steady state interpretation of the $\delta^{13}C$ record in which the organic fraction is calculated from $\delta^{13}C$ assuming constant weathering input $\delta^{13}C_{in} = -0.5‰$ and organic fractionation $\varepsilon = -24.5‰$ (e.g., Ref 51). Global ocean anoxic fraction calculated from the marine organic carbon burial fluxes, combined with the assumption of steady state marine phosphate. Dashed brown line shows modern value.

Forward biogeochemical modelling

Results of COPSE (Carbon, Oxygen, Phosphorous, Sulphur, Evolution)[53,59] forward biogeochemical box modeling using Toarcian-specific tectonic forcings representing large igneous province (LIP)-derived CO_2 and hydrate-derived CH_4 (see model description) are presented in **Figure S12**. The greenhouse perturbation translates to an increase in the input of phosphorous to the ocean via weathering, which boosts production, marine organic carbon burial and respiratory oxygen demand. This increases marine anoxia, reducing marine phosphate burial[63], further boosting organic carbon production, within a net positive feedback. The timing of anoxia lags behind that of the greenhouse input (consistent with the lag between the most negative lower limit of the $\delta^{13}C$ curve and the maximum value of the marine organic carbon burial flux implied by the inversion model). The hyperthermal interval also lasts longer than the negative CIE (due to the time needed for weathering to consume the CO_2 introduced via the perturbation).

The OAE terminates as a result of the “extra” (i.e. perturbation induced) CO_2 input being consumed by the silicate weathering flux, as well as a slight increase in oxygen caused by the elevated marine organic carbon burial flux (not shown, see supplement). As above, the time interval required to bring CO_2 down to pre-perturbation levels is increased if silicate weathering becomes supply limited.

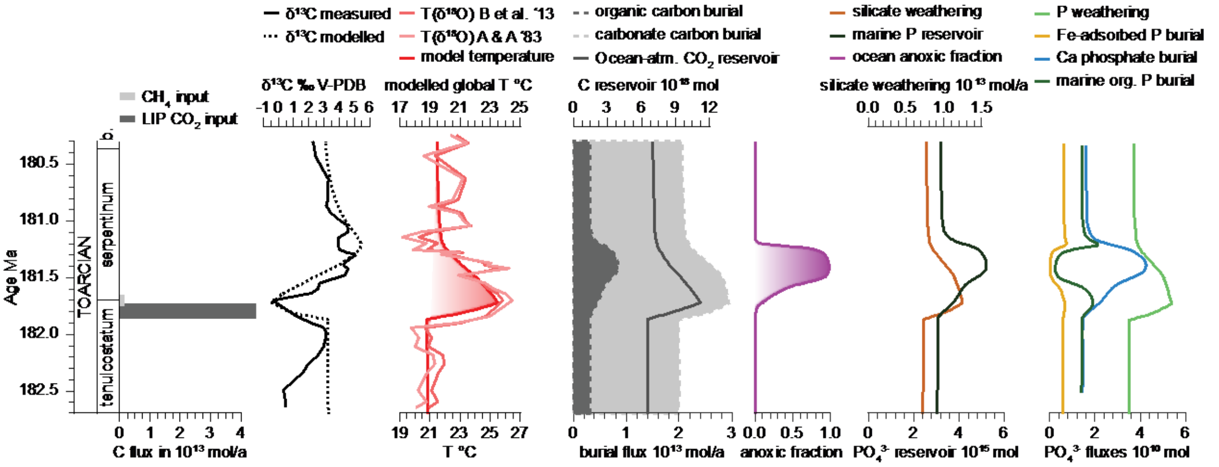

Figure S12: Reproduction of the coarse features of the $\delta^{13}C$ data by transient injection of CO_2 and CH_4 in the COPSE forward biogeochemical model. $\delta^{13}C$ data and greenhouse forcings relative to baseline fluxes (model description, equations (7) and (8)). An initial boundary condition of $\delta^{13}C_C$ = +2 ‰ is imposed on the carbonate carbon rock reservoir. Injected CO_2 is assumed to be thermogenic with $\delta^{13}C$ value of -27 ‰, and assumed $\delta^{13}C$ for injected methane is -60 ‰. Modelled global temperature (red line with shading) is shown together with local temperature reconstructions based on fossil oxygen isotope ratios converted using equations by Brand et al. (2013)[31] and Anderson and Arthur (1983)[38]. Carbon cycle fluxes and relative size of global ocean-atmosphere CO_2 reservoir (dark grey line). Note the increase in marine organic carbon burial (dark grey field) and silicate weathering (brown line), given by the minimum of the kinetically limited and supply limited flux values with W_{max} = 2 * $silw_0$ associated with the anoxic event. Global ocean anoxic fraction and various fluxes relevant to the marine phosphate cycle. Note the decline in iron-adsorbed and organic phosphate burial as anoxia increase.

Formulation of ocean anoxic fraction

The representation of the global ocean anoxic fraction depicted in **Figure S12** is defined as the fraction of the ocean surface area below which the saturation of oxygen in the oxygen minimum zone would be less than 10%[64]. An alternative formulation[59] represents the fraction of the seafloor directly overlain by anoxic waters. This corresponds to a much lower baseline value[65] but is arguably a better encapsulation of the spatial heterogeneity in marine redox state. Nevertheless, the qualitative feedback sequence of “greenhouse gas injection, negative CIE, hyperthermal, elevated organic carbon burial and anoxia, recovery from perturbation via silicate weathering CO_2 sink” is robust to such changes in formulation, as is the sustained temperature increase.

The $\delta^{13}C$ record can either be evaluated in terms of simple mass balance calculations, used as an input to proxy inversion models, or a target at which that forward models aim

via appropriate formulation of the underlying biogeochemical dynamics. We briefly consider each of these approaches in turn.

Qualitative description of feedback sequence corresponding to model results

The modelling illustrates that the basic feedback sequence resulting from the LIP-associated CO_2 input is as follows: an increase in silicate weathering, leading to an increase in the size of the marine phosphate reservoir, thus an increase in marine production (increasing remineralization of organic carbon in the ocean, thus increasing marine anoxia over a short timescale) and marine organic carbon burial (ultimately increasing a long-term source of oxygen). The short-term increase in anoxia results in a decrease (and eventual cessation) of Fe-adsorbed phosphate burial. This leads to a secondary increase in the marine phosphate reservoir, which in the short-term boosts production, therefore remineralization, therefore anoxia (i.e. a positive feedback), and in the long term boosts marine organic carbon burial, therefore oxygen, therefore a decrease in anoxia (a negative feedback).

Because each of these processes operates over different timescales, predicting the system's net response time is complex. Examples of the timescales over which various fluxes and reservoirs respond to the greenhouse perturbation are given in Table S1, which corresponds to a model run based on the same set of parameter choices as those used to produce the results in the main text (see also **Table S10**). The peak of the CO_2 perturbation shown corresponds to an estimated atmospheric CO_2 reservoir of $\sim 12.31 PAL$, which happens at 181.724 million years before present. The fall from this maximum value corresponds to an e-folding time of:

$$12.31 \cdot \left(1 - \frac{1}{e}\right) = 7.7814 PAL \equiv 293 Kyrs.$$

This is broadly in line with the error margin placed on the e-folding time of $170 - 340 Kyrs$, probably $\sim 240 Kyrs$, estimated from more complex climate models[66].

The anoxia function responds within approximately 18kyrs to the increased nutrient input, but the above-mentioned feedback involves a sharp decrease in Fe-adsorbed P burial, which does not become significant until anoxia reaches roughly $f_{anox} > 0.8$. (The asymptotic nature of Fe-P burial's response to anoxia is due to the fact that anoxia is a logistic function that increases with increasingly P, but decreases with increasing O, and is determined by the ratio of these two variables, both of which are increasing in size at different rates). If the OAE is defined as the entire perturbation of f_{anox}, it lasts for

approximately 565kyrs. If it is defined as the continuous interval of time during which $f_{anox} > 0.8$, it lasts~266 kyrs. This is shorter than (although within a reasonable "ballpark" estimate of) the~1.4Myr OAE duration that this model system produces[64] in the absence of a significant greenhouse perturbation – because the elevated nutrient input associated with the temperature/ CO_2 pushes the system through the above-mentioned feedback sequence at a faster rate than would occur at, for example, 1PAL CO_2. It is important to note that the numbers quoted here, and shown in the table, are subject to an error margin dictated by uncertainty in parameter choices, which in this case have been selected so as to most accurately reproduce the carbon isotope data. This is illustrated by the sensitivity analysis depicted in **Figure S13**, which shows different values for the climate sensivity parameter k_c.

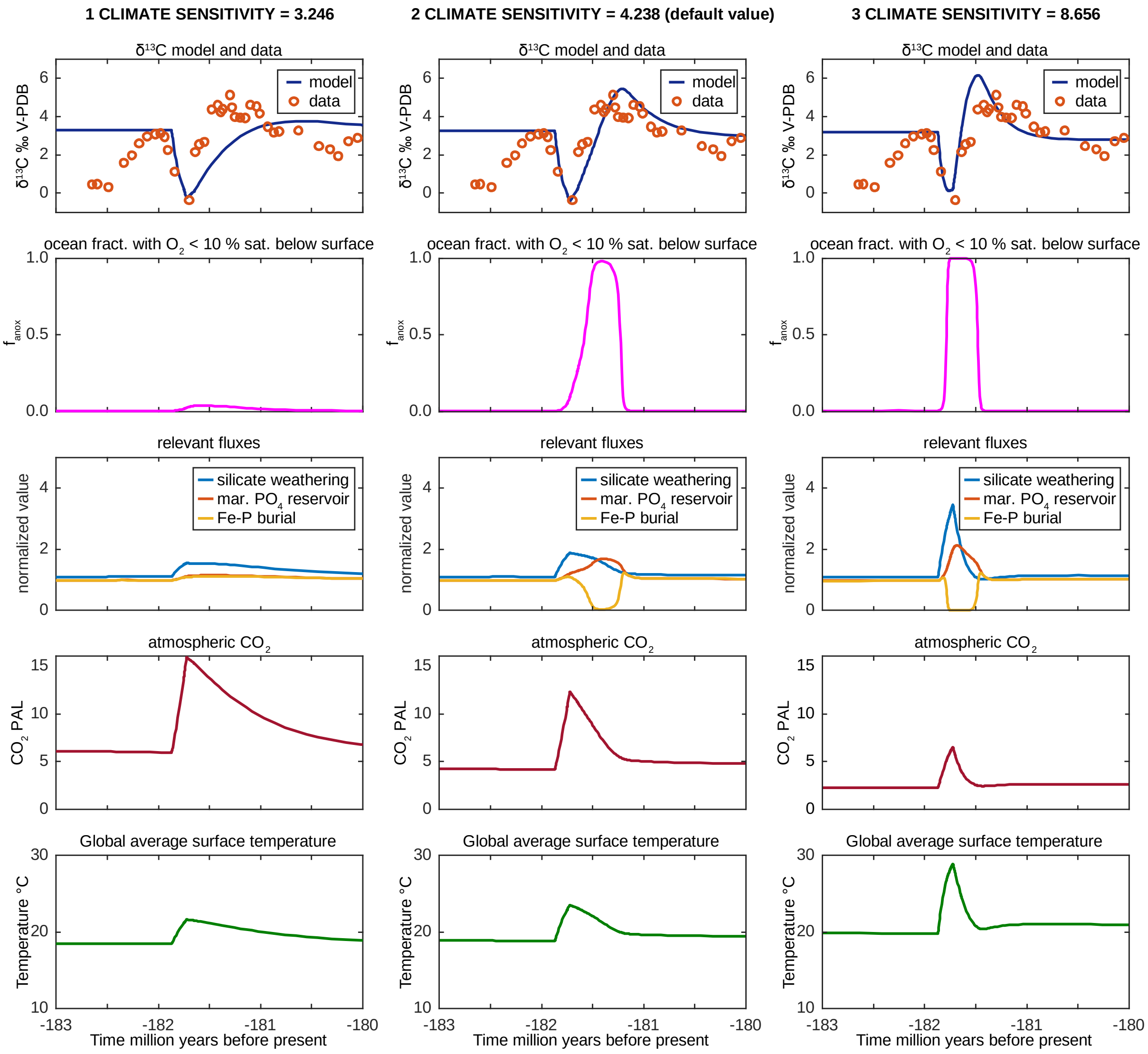

Figure S13: Model response as a function of the climate sensitivity parameter.

Model temperature is of the form $T = T_L + k_c \log(\frac{CO_2}{CO_{2\,0}})$, where T_L describes the increase in temperature associated with the increase in solar luminosity over Earth's history, whereas k_c represents temperature's sensitivity to log-normalized $CO_{2\,0}$. The default value $k_c = 4.328$, corresponds to the widely assumed climate sensitivity of $4.328 \cdot \log(2) \approx 3$ degrees warming per doubling of CO_2. Figure S1 illustrates the sensitivity of the depicted feedback sequence to larger and smaller values of k_c, taken from the range explored in the COPSE-reloaded model[59]. A larger value of k_c means that a smaller unit CO_2 change will cause the same temperature change – meaning that the greenhouse perturbation triggers the feedback sequence described more rapidly, and the overall greenhouse gas change is lower. Similarly a smaller k_c requires a larger CO_2 change to initiate the feedback sequence, which is spread over a correspondingly larger timescale (i.e. relative to the response times in **Table S11**).

Based on previous discussion of the relationship between the silicate weathering and OAE duration[67], we investigated the idea of supply limitation of this flux. We find that the best reproduction of the $\delta^{13}C$ data is produced with a maximum kinetic limited silicate weathering of around twice the modern value (i.e. such that silicate weathering becomes supply limited rather than kinetically limited above this threshold). However, the difference, in terms of reproducing the carbon isotope data, is slight relative to silicate weathering that is solely kinetically limited (**Figure S14**), because the kinetically limited flux does not significantly exceed this magnitude in any case. Furthermore, the threshold at which supply limitation begins is highly uncertain, and a fair case can be made that it is likely higher than twice the present value. We nevertheless retain the $W_{max} = 2$ threshold (see model description), because it accurately reproduces the data. Some limitation of terrestrial silicate weathering and its biotic enhancement may also be a legitimate assumption based on previous arguments[68] for a link between enhanced fire probability and repression of growth of the terrestrial biosphere during the Toarcian event. In other words, our modelling is compatible with the idea that the silicate weathering flux plateaued at about twice its present magnitude, which could have occurred for a range of reasons.

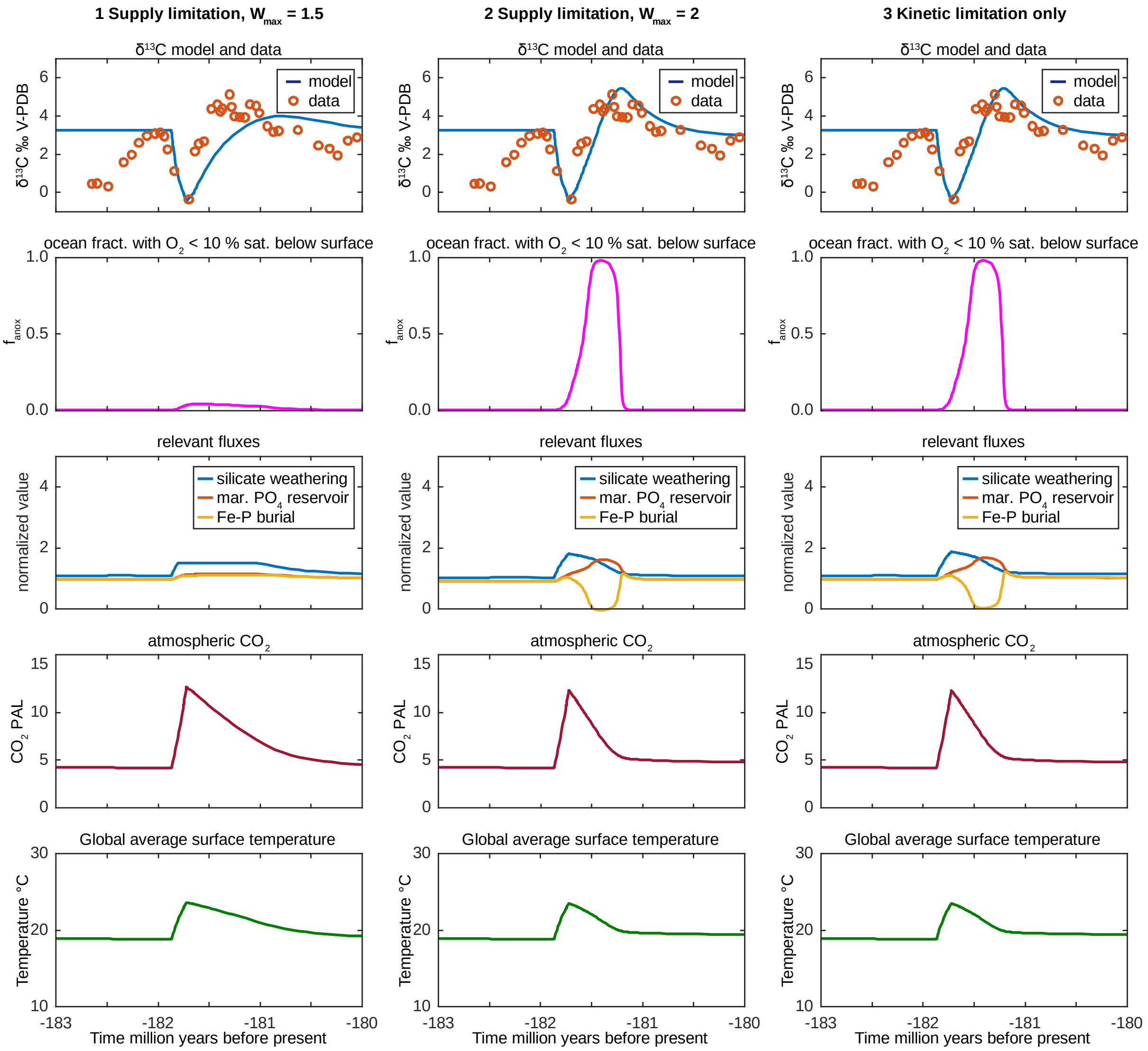

Figure S14: Sensitivity study exploring the effects of the supply limitation threshold.

References

[1]Duarte, L.V., 2007. Lithostratigraphy, sequence stratigraphy and depositional setting of the Pliensbachian and Toarcian series in the Lusitanian Basin, Portugal. Pp. 17–23 in R. B. Rocha, ed. The Peniche section (Portugal). Contributions to the definition of the Toarcian GSSP. International Subcommission on Jurassic Stratigraphy, Lisbon.

[2]Pittet, B., Suan, G., Lenoir, F., Duarte, L.V., Mattioli, E., 2014. Carbon isotope evidence for sedimentary discontinuities in the lower Toarcian of the Lusitanian Basin (Portugal): Sea level change at the onset of the Oceanic Anoxic Event. Sedimentary Geology 303, 1–14. Doi: 10.1016/j.sedgeo.2014.01.001.

[3]Rodríguez-Tovar, F. J., Miguez-Salas, O., Duarte, L.V., 2017. Toarcian Oceanic Anoxic Event induced unusual behaviour and palaeobiological changes in *Thalassinoides* tracemakers. Palaeogeography, Palaeoclimatology, Palaeoecology 485, 46–56.

[4]Piazza, V., Duarte, L.V., Renaudie, J., Aberhan, M., 2019. Reductions in body size of benthic macroinvertebrates as a precursor of the early Toarcian (Early Juarssic) extinction event in the Lusitanian Basin, Portugal. Paleobiology 45 (2), 296–316. doi: 10.1017/pab.2019.11.

[5]Gómez, J. J., Goy, A., 2005. Late Triassic and Early Jurassic palaeogeographic evolution and depositional cycles of the Western Tethys Iberian platform system (Eastern Spain). Palaeogeography, Palaeoclimatology, Palaeoecology 222, 77–94.

[6]Goy, A., Gómez, J.J., Yébenes, A., 1976. El Jurásico de la Rama Castellana de la Cordillera Ibérica (Mitad Norte). I. Unidades litoestratigráficas 32, 391–423.

[7]Gahr, M.E., 2002. Palökologie des Makrobenthos aus dem Unter-Toarc SW-Europas. Beringeria 31, 3–204.

[8]García Joral, F., Goy, A., 2010. Rhynchonellida (Brachiopoda) Biozones of the Toarcian in the Iberian and Cantabrian Cordilleras (Spain). In: Comunicaciones del V Congreso del Jurásico de España. Museo del Jurásico de Asturias (MUJA), Colunga, 8-11 de septiembre de 2010 (J.I. Ruiz-Omeñaca, L. Piñuela & J.C. García-Ramos, Eds.). Museo del Jurásico de Asturias, Colunga, 3–9.

[9]Gahr, M.E., 2005. Response of Lower Toarcian (Lower Jurassic) macrobenthos of the Iberian Peninsula to sea level changes and mass extinction. Journal of Iberian Geology 31, 197–215.

[10]Fürsich, F.T., Berndt, R., Scheuer, T., Gahr, M., 2001. Comparative ecological analysis of Toarcian (Lower Jurassic) benthic faunas from southern France and east-central Spain. Lethaia 34, 169–199.

[11]Aurell, M., Robles, S., Bádenas, B., Rosales, I., Quesada, S., Meléndez, G., García-Ramos, J.C., 2003. Transgressive-regressive cycles and Jurassic palaeogeography of northeast Iberia. Sedimentary Geology 162, 239–271.

[12]Duarte, L.V., Soares, A.F., 2002. Litostratigraphy of the Lower Jurassic marly limestone units from the Lusitanian Basin (Portugal). Comun. Inst. Geol. e Mineiro 89, 135–154.

[13]Mouterde, R., Ruget, C., Moitinho de Almeida, F., 1964-1965. Coupe du Lias au Sud de Condeixa. Comunicações dos Serviços Geológicas de Portugal 48, 61–91.

[14]Comas-Rengifo, M. J., Duarte, L.V., García Joral, F., Goy, A., 2013. The brachiopod record in the Lower Toarcian (Jurassic) of the Rabaçal - Condeixa region (Portugal): stratigraphic distribution and palaeobiogeography. Comunicações Geológicas 100, 37–42.

[15]Perilli, N., Duarte, L.V., 2006. Toarcian nannobiohorizons from Lusitanian Basin (Portugal) and their calibration against ammonite zones. Rivista Italiana di Paleontologia e Stratigrafia 112, 417–434.

[16]Correia, V.D.P.F., 2018. Jurassic dinoflagellate cyst biostratigraphy of the Lusitanian Basin, West-central Portugal, and its relevance to the opening of the North Atlantic and petroleum geology, PhD thesis, Universidade do Algarve, 315pp.

[17]Duarte, L.V., 1997. Facies analysis and sequential evolution of the Toarcian-Lower Aalenian series in the Lusitanian Basin (Portugal). Communicações do Instituto Geológico e Mineiro 83, 65–94.

[18]Duarte, L.V., Perilli, N., Dino, R., Rodrigues, R., Paredes, R., 2004. Lower to Middle Toarcian from the Coimbra region (Lusitanian Basin, Portugal): sequence stratigraphy, calcareous nannofossils and stable-isotope evolution. Rivista Italiana di Paleontologia e Stratigrafia 110, 115–127.

[19]Duarte, L.V., Oliveira, L.C., Rodrigues, R., 2007. Carbon isotopes as a sequence stratigraphic tool: examples from the Lower to Middle Toarcian marly limestones of Portugal. Boletín Geológico y Minero 118, 3–18.

[20]Kullberg, J.C., Oloriz, F., Marques, B., Caetano, P.S., Rocha, R.B., 2001. Sedimentary Geology 139, 49–70.

[21]Röhl, H.-J., Schmidt-Röhl, A., Oschmann, W., Frimmel, A., Schwark, L., 2001. The Posidonia Shale (Lower Toarcian) of SW-Germany: an oxygen-depleted ecosystem controlled by sea level and palaeoclimate. Palaeogeography, Palaeoclimatology, Palaeoecology 165, 27–52.

[22]Wignall, P.B., Bond, D.P.G., 2008. The end-Triassic and Early Jurassic mass extinction records in the British Isles. Proceedings of the Geologists' Association 119, 73–84.

[23]Danise, S., Twitchett, R.J., Little, C.T.S., 2015. Environmental controls on Jurassic marine ecosystems during global warming. Geology 43, 263–266.

[24]Martindale, R.C., Aberhan, M., 2017. Response of microbenthic communities to the Toarcian Oceanic Anoxic Event in northeastern Panthalassa (Ya Ha Tinda, Alberta, Canada). Paleogeography, Palaeoclimatology, Palaeoecology 478, 103–120. doi: 10.1016/j.palaeo.2017.01.009.

[25]Duarte, L.V., Rodrigues, R., Oliveira, L.C., Silva, F., 2005. Avaliação preliminar das variações do carbono orgânico total nos sedimentos margosos do Jurássico inferior da Bacia Lusitânica (Portugal). Pp. 39-43 *in* XIV Semana de Geoquímica and VIII Congresso de Geoquímica dos Países de Lingua Portuguesa, Aveiro, I.

[26]García Joral, F., Gómez, J.J., Goy, A., 2011. Mass extinction and recovery of the Early Toarcian (Early Jurassic) brachiopods linked to climate change in Northern and Central Spain. Palaeogeography, Palaeoclimatology, Palaeoecology 302, 367–380.

[27]Miguez-Salas, O., Rodríguez-Tovar, F.J., Duarte, L.V., 2017. Selective incidence of the Toarcian oceanic anoxic event on macroinvertebrate marine communities: a case from the Lusitanian basin, Portugal. Lethaia 50, 548–560.

[28]Rodríguez-Tovar, F. J., Miguez-Salas, O., Dorador, J., Duarte, L.V., 2019. Opportunistic behaviour after the Toarcian Oceanic Anoxic Event: The trace fossil *Halimedides*. Palaeogeography, Palaeoclimatology, Palaeoecology 520, 240–256. Doi: 10.1016/j.palaeo.2019.01.036.

[29]Canudo, J.I., 2018. The collection of type fossils of the Natural Science Museum of the University of Zaragoza (Spain). Geoheritage 10 (3), 385–392. doi: 10.1007/s12371-017-0228-1.

[30]Imai N., Terashima S., Itoh S, Ando A., 1996. 1996 Compilation of analytical data on nine GSJ geochemical reference samples, "sedimentary rock series". Geostandards Newsletter 20 (2), 165–216.

[31]Govindaraju K., 1994. 1994 Compilation of working values and sample description for 383 geostandards. Geostandards Newsletter 18, 1–158.

[32]Brand, U., Azmy, K., Bitner, M.A., Logan, A., Zuschin, M., Came, R., Ruggiero, E., 2013. Oxygen isotopes and MgCO3 in brachiopod calcite and a new paleotemperature equation. Chemical Geology 359, 23–31. doi: 10.1016/j.chemgeo.2013.09.014.

[33]Suan, G., Mattioli, E., Pittet, B., Mailliot, S., Lécuyer, C., 2008. Evidence for major environmental perturbation prior to and during the Toarcian (Early Jurassic) oceanic anoxic event from the Lusitanian Basin, Portugal. Paleoceanography 23, PA1202, doi: 10.1029/2007PA001459.

[34]Ullmann, C.V., Böhm, F., Rickaby, R.E.M., Wiechert, U., Korte, C., 2013. The Giant Pacific Oyster (Crassostrea gigas) as a modern analog for fossil ostreoids: Isotopic (Ca, O, C) and elemental (Mg/Ca, Sr/Ca, Mn/Ca) proxies. Geochemistry, Geophysics, Geosystems 14 (10), 4109–4120. doi: 10.1002/ggge.20257.

[35]Ullmann, C.V., Korte, C., 2015. Diagenetic alteration in low-Mg calcite from macrofossils: a review. Geological Quarterly 59 (1), 3–20.

[36]McConnaughey, T., 1989. ^{13}C and ^{18}O isotopic disequilibrium in biological carbonates: I. Patterns. Geochimica et Cosmochimica Acta 53, 151–162.

[37]Wefer, G., Berger, W.H., 1991. Isotope paleontology: growth and composition of extant calcareous species. Marine Geology 100, 207–248.

[38]Ullmann, C.V., Frei, R., Korte, C., Lüter, C., 2017. Element/Ca, C and O isotope ratios in modern brachiopods: Species-specific signals of biomineralisation. Chemical Geology 460, 15–24. doi: 10.1016/j.chemgeo.2017.03.034.

[39]Anderson, T.F., Arthur, M.A., 1983. Stable isotopes of oxygen and carbon and their application to sedimentologic and paleoenvironmental problems. In: Arthur, M.A., Anderson, T.F., Kaplan, I.R., Veizer, J., Land, L.S. (Eds.), Stable Isotopes in Sedimentary Geology. SEPM, Dallas, Texas, pp. 1–151.

[40]Jiménez-López, C., Romanek, C.S., Huertas, F.J., Ohmoto, H., Caballero, E., 2004. Oxygen isotope fractionation in synthetic magnesian calcite. Geochimica et Cosmochimica Acta 68 (16), 3367–3377.

[41]Gradstein, F.M., Ogg, J.G., Schmitz, M., Ogg, G. (eds.), 2012. The geologic time scale 2012. elsevier.

[42]Holser, W.T., Schidlowski, M., Mackenzie, F.T., Maynard, J.B., 1988. in Chemical Cycles in the Evolution of the Earth, eds. Gregor, C. B., Garrels, R. M., Mackenzie, F. T. & Maynard, J. B. (Wiley, New York), pp. 105–173.

[43]Dickens, G.R., O'Neill, J.R., Rea, D.K., Owen, R.M., 1995. Dissociation of oceanic methane as the cause of the carbon isotopic excursion at the end of the Paleocene. Paleoceanography 10 (6), 965–971.

[44]Hesselbo, S.P., Gröcke, D.R., Jenkyns, H.C., Bjerrum, C.J., Farrimond, P., Morgans Bell, H.S., Green, O.R., 2000. Massive dissociation of gas hydrate during an oceanic anoxic event. Nature 406, 392–395.

[45]Jiao, N., Herndl, G.J., Hansell, D.A., Benner R., Kattner G., Wilhelm, S.W., Kirchman, D.L., Weinbauer M.G., Luo, T., Chen, F., Azam, F., 2010. Microbial production of recalcitrant dissolved organic matter: long-term carbon storage in the global ocean. Nat. Rev. Microbiol. 8, 593–599.

[46]Kvenvolden, K.A., 2002. Methane Hydrate in the global carbon cycle. Terra Nova 14, 302–306.

[47]Dunkley-Jones, T., Ridgwell, A., Lunt, D.J., Maslin, M.A., Schmidt, D.N., Valdes, P.J., 2010. A Palaeogene perspective on climate sensitivity and methane hydrate instability. Phil. Trans. R. Soc. A 368, 2395–2415.

[48]Jones, M.T., Jerram, D.A. Svensen, H.H., Grove, C., 2016. The effects of large igneous provinces on the global carbon and sulphur cycles. Paleogeography, Palaeoclimatology, Palaeoecology 441 4–21.
[49]Beerling, D.J., Lomas, M.R., Gröcke, D.R., 2002. On the nature of methane gas hydrate dissociation during the Toarcian and Aptian oceanic anoxic events. Am. J. Sci. 302, 28–49.
[50]Dera, G., Donnadieu, Y., 2012. Modeling evidences for global warming, Arctic seawater freshening, and sluggish oceanic circulation during the Early Toarcian anoxic event. Paleoceanography 27, PA2211. doi: 10.1029/2012PA002283.
[51]Kump, L.R., Arthur, M.A., 1999. Interpreting carbon isotope excursions: carbonates and organic matter. Chem. Geol. 161, 181–198.
[52]Betts, R.A., Holland, H.D., 1991. The oxygen content of ocean bottom waters, the burial efficiency of organic carbon, and the regulation of atmospheric oxygen. Palaeogeography, Palaeoclimatology, Palaeoecology 97, 5–8.
[53]Bergman, N.M., Lenton, T.M., Watson, A.J., 2004. COPSE: a new model of biogeochemical cycling over Phanerozoic time. Am. J. Sci. 304, 397–437.
[54]Berner, R.A., 1994. Geocarb II. A revised model of atmospheric CO_2 over geologic time. Am. J. Sci. 294, 55–91.
[55]Royer, D.L., Donnadieu, Y., Park, J., Kowalczyk, J. Godderis, Y., 2014. Error analysis of CO_2 and O_2 estimates from the long-term geochemical model Geocarbsulf. Am. J. Sci. 314, 1259-1284.
[56]Berner, R.A., 2006. Geocarbsulf: A combined model for Phanerozoic atmospheric O_2 and CO_2. Geochimica et Cosmochimica Acta 70 (23), 5653–5664.
[57]Caldeira, K. & Kasting, J. F. 1992, The life span of the biosphere revisited: Nature 360, 721–723.
[58]Mills, B.J.W, Watson, A.J., Goldblatt, C., Boyle, R.A., Lenton, T.M.L., 2011. Timing of Neoproterozoic glaciations linked to transport-limited global weathering. Nature Geoscience 4, 861–864.
[59]Lenton, T.M., Daines, S.J., Mills, B.M., 2018. COPSE reloaded: An improved model of biogeochemical cycling over Phanerozoic time. Earth Sci. Rev 178, 1–28.
[60]Bond, W.J., Woodward, F.I., Midgley, G.F., 2005. The global distribution of ecosystems in a world without fire. New Phytol. 165, 525–538.
[61]Gill, B.C., Lyons, T.W., Jenkyns, H.C., 2011. A global perturbation to the sulphur cycle during the Toarcian ocean. Earth Plan. Sci. Lett. 312, 484–496.
[62]Cervantes, P., Wallace, P.J., 2003. Role of H_2O in subduction-zone magmatism: New insights from melt inclusions in high-Mg basalts from central Mexico. Geology 31, 235–238.
[63]Van Cappellen, P., Ingall, E.D. 1994. Benthic phosphorus Regeneration, primary production, and ocean anoxia: A model of the coupled marine biogeochemical cycles of carbon and phosphorus Paleoceanography 9, (5), 677–692.
[64]Handoh, I.C., Lenton, T.M., 2003. Periodic mid-Cretaceous oceanic anoxic events linked by oscillations of the phosphorus and oxygen biogeochemical cycles. Global. Biogeochem. Cycles. 17 (4), 1092.
[65]Helly, J.J., Levin, L.A., 2004. Global distribution of naturally occurring marine hypoxia on continental margins. Deep-Sea Res. I. Oceanogr. Res. Pap. 51 (9), 1159–1168.
[66]Colbourn, G., Ridgwell, A., Lenton, T.M., 2015. The timescale of the silicate weathering negative feedback on atmospheric CO_2. Global Biogeochemical cycles 29, 583-596.
[67]Kump, L.R., 2013. Prolonged Late Permian–Early Triassic hyperthermal: A failure of climate regulation? Phil. Trans. R. Soc. A 376: 20170078.

[68]Baker, S.J., Hesselbo, S.P., Lenton, T.M., Duarte. L.V., Belcher, C.M. Charcoal evidence that rising atmospheric oxygen terminated Early Jurassic ocean anoxia. Nat. Comm. 8:15018.

Table S1: Occurences of macrofossils in the studied sections

locality		sample name	height m	Belemnites	Bivalvia	Brachiopoda										
						Rhynchonellida								Terebratulida	Spiriferinida	Lingulida
					Gryphaea	*Cirpa*	*Gibbirhynchia*	*Nannirhynchia*	*Quadratirhynchia*	*Tetrarhynchia*	*Choffatirhynchia*	*Homoeorhynchia*	*Soaresirhynchia*			
Barranco de la Cañada		BC23	27.90											7		
Barranco de la Cañada		BC22	26.70		1							10		37		
Barranco de la Cañada		BC21	25.55		1							6		4		
Barranco de la Cañada		BC20	24.55	1								20		12		
Barranco de la Cañada		BC19	23.45									1		5		
Barranco de la Cañada		BC18	22.65									1		3		
Barranco de la Cañada		BC17	21.45								1			12		
Barranco de la Cañada		BC16	20.45											1		
Barranco de la Cañada		BC15	19.85		2						1	2		13		
Barranco de la Cañada		BC14	19.35		3							2		9		
Barranco de la Cañada		BC13	18.85									4		11		
Barranco de la Cañada		BC12	18.35									1		13		
Barranco de la Cañada		BC11	17.95											6		
Barranco de la Cañada		BC10	17.30		3						1	3		10		
Barranco de la Cañada		BC9	16.70		5						2	6		44		
Barranco de la Cañada		BC8	16.05								1	5		9		1
Barranco de la Cañada		BC7	15.25		1							8		10		
Barranco de la Cañada		BC6	14.85		5						13	41		1		
Barranco de la Cañada		BC5	14.25									11		11		
Barranco de la Cañada		BC4	13.75				9					17		11		
Barranco de la Cañada		BC3	13.20	1	1						2	3		32		
Barranco de la Cañada		BC2	12.60	1			12				19			26		1
Barranco de la Cañada		BC1b	12.35	1	2		16				6			27		
Barranco de la Cañada		BC1a	12.10										5			
Barranco de la Cañada		C37b	11.80										1			5
Barranco de la Cañada		C37a	11.30										31			
Barranco de la Cañada		C33b	11.10		1								30			1
Barranco de la Cañada		C33a	10.80										13			3
Barranco de la Cañada		C31	10.40										5			1
Barranco de la Cañada		C30	10.20										4			1
Barranco de la Cañada		C29	10.00										6			1
Barranco de la Cañada		C28	9.40										148			3
Barranco de la Cañada		C26	8.90		20								137			1
Barranco de la Cañada		C25b	8.70		1								12			5
Barranco de la Cañada		C25a	8.40		1				81							1
Barranco de la Cañada		C22	7.80		12										1	2
Barranco de la Cañada		C21	7.20											91		
Barranco de la Cañada		C20	6.70		19									93		
Barranco de la Cañada		C19	6.20		44				11	5				1		
Barranco de la Cañada		C18c	5.90		69		3		15							
Barranco de la Cañada		C18b	5.60		80				5					2	1	
Barranco de la Cañada		C18a	5.20	2	77				6	2				1		
Barranco de la Cañada		C15b	4.60		34									6		
Barranco de la Cañada		C15a	4.00		88		3		1	6				7		
Barranco de la Cañada		C12b	3.40		42									35		
Barranco de la Cañada		C12a	2.80		42		4		17					9		
Barranco de la Cañada		C7b	1.60		63									4		
Barranco de la Cañada		C7a	0.80	8	36		1							3		
Barranco de la Cañada		C5	0.40	4	35		28							2		
Fonte Coberta / Rabaçal	Rabaçal	R24b	28.35											17		
Fonte Coberta / Rabaçal	Rabaçal	R24a	27.65									2		4		
Fonte Coberta / Rabaçal	Rabaçal	R23e	27.30									1				
Fonte Coberta / Rabaçal	Rabaçal	R23d	27.00		1									9		
Fonte Coberta / Rabaçal	Rabaçal	R23c	26.60									1		24		
Fonte Coberta / Rabaçal	Rabaçal	R23b	26.25											1		
Fonte Coberta / Rabaçal	Rabaçal	R23a	25.05											9		
Fonte Coberta / Rabaçal	F.C.	FC22b	24.35											1		
Fonte Coberta / Rabaçal	F.C.	FC22a	23.85											1		
Fonte Coberta / Rabaçal	F.C.	FC21b	23.25											6		1
Fonte Coberta / Rabaçal	F.C.	FC21a	22.25											5		
Fonte Coberta / Rabaçal	F.C.	FC19c	21.40										21	1		
Fonte Coberta / Rabaçal	F.C.	FC19b	20.30													
Fonte Coberta / Rabaçal	F.C. (trench)	FC19a	19.05										1			
Fonte Coberta / Rabaçal	F.C. (trench)	FC17f	17.75													
Fonte Coberta / Rabaçal	F.C. (trench)	FC17e	17.25										5	3		
Fonte Coberta / Rabaçal	F.C. (trench)	FC17d	16.80										7	4		
Fonte Coberta / Rabaçal	F.C. (trench)	FC17c	16.35										2	1		
Fonte Coberta / Rabaçal	F.C. (trench)	FC17b	15.85										2	1		
Fonte Coberta / Rabaçal	F.C. (trench)	FC17a	15.40										9	6		
Fonte Coberta / Rabaçal	F.C. (trench)	FC16e	14.80										12	4		
Fonte Coberta / Rabaçal	F.C. (trench)	FC16d	14.20										4			
Fonte Coberta / Rabaçal	F.C. (trench)	FC16c	13.75													
Fonte Coberta / Rabaçal	F.C. (trench)	FC16b	13.25													
Fonte Coberta / Rabaçal	F.C. (trench)	FC16a	13.00													
Fonte Coberta / Rabaçal	F.C. (trench)	FC14i	12.30													
Fonte Coberta / Rabaçal	F.C. (trench)	FC14h	11.60													

Fonte Coberta / Rabaçal	F.C. (trench)	FC14g	10.60													
Fonte Coberta / Rabaçal	F.C. (trench)	FC14f	9.60													
Fonte Coberta / Rabaçal	F.C. (trench)	FC14e	8.75													
Fonte Coberta / Rabaçal	F.C.	FC14d	7.90											1		
Fonte Coberta / Rabaçal	F.C.	FC14c	7.30	1		1		24						8		
Fonte Coberta / Rabaçal	F.C.	FC14b	6.90					50						4		
Fonte Coberta / Rabaçal	F.C.	FC14a	6.45	4				10						3		
Fonte Coberta / Rabaçal	F.C.	FC13e	6.10	1				66						5		
Fonte Coberta / Rabaçal	F.C.	FC13d	5.50	3				7						14	1	
Fonte Coberta / Rabaçal	F.C.	FC13c	4.90	3				8						17	1	
Fonte Coberta / Rabaçal	F.C.	FC13b	4.25	10		1		16						21	12	
Fonte Coberta / Rabaçal	F.C.	FC13a	3.30	3		1		12						20	7	
Fonte Coberta / Rabaçal	F.C.	FC12	2.35	7		5		10					2	9	21	2
Fonte Coberta / Rabaçal	F.C.	FC11	1.85	2				1						8	17	2
Fonte Coberta / Rabaçal	F.C.	FC10	1.45	4				14						9	11	
Fonte Coberta / Rabaçal	F.C.	FC09	1.05	1				27						10	17	
Fonte Coberta / Rabaçal	F.C.	FC08	0.80				3	31					1	17	14	
Fonte Coberta / Rabaçal	F.C.	FC07	0.40				1	3						12	1	
Fonte Coberta / Rabaçal	F.C.	FC06	0.00		4	4								15	4	

Table S2: Analytical results for element-Ca standards measured alongside studied samples. Note that samples from Portugal were measured using a different batch of AK and BCQC and standard data therefore differ for these two stock solutions.

		Mg/Ca mmol/mol	Sr/Ca mmol/mol	Mn/Ca mmol/mol	Fe/Ca mmol/mol	S/Ca mmol/mol	P/Ca mmol/mol
**should**							
**bias %**							
**avg**		**15.37**	**0.341**	**0.032**	**0.142**	**0.434**	**0.411**
**2ds**		**0.07**	**0.005**	**0.001**	**0.010**	**0.221**	**0.169**
**2rsd**		**0.46**	**1.40**	**2.8**	**7.2**	**51**	**41**
**n**		**72**	**72**	**72**	**72**	**72**	**72**
JLs-1	01/06/2018	15.412	0.343	0.031	0.150	0.337	0.544
JLs-1	01/06/2018	15.311	0.342	0.031	0.152	0.414	0.398
JLs-1	01/06/2018	15.362	0.343	0.031	0.150	0.565	0.549
JLs-1	01/06/2018	15.387	0.343	0.031	0.148	0.364	0.182
JLs-1	01/06/2018	15.407	0.343	0.031	0.152	0.432	0.484
JLs-1	01/06/2018	15.389	0.343	0.032	0.148	0.480	0.347
JLs-1	01/06/2018	15.383	0.343	0.032	0.143	0.520	0.326
JLs-1	01/06/2018	15.386	0.342	0.032	0.142	0.496	0.498
JLs-1	01/06/2018	15.382	0.343	0.032	0.143	0.364	0.431
JLs-1	01/06/2018	15.388	0.343	0.032	0.146	0.484	0.425
JLs-1	01/06/2018	15.437	0.343	0.032	0.142	0.573	0.369
JLs-1	01/06/2018	15.401	0.343	0.031	0.143	0.404	0.294
JLs-1	04/06/2018	15.458	0.344	0.032	0.147	0.414	0.323
JLs-1	04/06/2018	15.311	0.342	0.032	0.147	0.405	0.401
JLs-1	04/06/2018	15.355	0.342	0.032	0.147	0.518	0.393
JLs-1	04/06/2018	15.353	0.342	0.032	0.147	0.508	0.386
JLs-1	04/06/2018	15.407	0.343	0.032	0.143	0.434	0.413
JLs-1	04/06/2018	15.353	0.342	0.032	0.144	0.428	0.372
JLs-1	04/06/2018	15.345	0.341	0.032	0.151	0.396	0.446
JLs-1	04/06/2018	15.362	0.342	0.032	0.150	0.550	0.384
JLs-1	04/06/2018	15.350	0.342	0.031	0.152	0.689	0.465
JLs-1	04/06/2018	15.376	0.342	0.031	0.152	0.336	0.462
JLs-1	04/06/2018	15.343	0.341	0.032	0.149	0.398	0.501
JLs-1	04/06/2018	15.366	0.342	0.032	0.151	0.308	0.342
JLs-1	08/06/2018	15.407	0.342	0.032	0.146	0.328	0.162
JLs-1	08/06/2018	15.359	0.343	0.032	0.142	0.740	0.456
JLs-1	08/06/2018	15.395	0.342	0.032	0.139	0.573	0.527
JLs-1	08/06/2018	15.369	0.342	0.032	0.140	0.514	0.438
JLs-1	08/06/2018	15.393	0.343	0.032	0.143	0.519	0.417
JLs-1	08/06/2018	15.403	0.344	0.032	0.141	0.414	0.277
JLs-1	08/06/2018	15.385	0.342	0.031	0.138	0.575	0.431
JLs-1	08/06/2018	15.401	0.342	0.032	0.136	0.424	0.541
JLs-1	08/06/2018	15.406	0.342	0.032	0.135	0.452	0.683
JLs-1	08/06/2018	15.403	0.341	0.031	0.135	0.509	0.528
JLs-1	08/06/2018	15.390	0.343	0.031	0.134	0.418	0.424
JLs-1	08/06/2018	15.392	0.344	0.031	0.139	0.449	0.396
JLs-1	25/06/2018	15.446	0.344	0.032	0.143	0.319	0.323
JLs-1	25/06/2018	15.290	0.343	0.031	0.138	0.229	0.551
JLs-1	25/06/2018	15.367	0.343	0.031	0.142	0.388	0.481
JLs-1	25/06/2018	15.358	0.342	0.031	0.138	0.335	0.349
JLs-1	25/06/2018	15.307	0.341	0.031	0.137	0.520	0.462
JLs-1	25/06/2018	15.378	0.341	0.032	0.141	0.345	0.435
JLs-1	25/06/2018	15.341	0.341	0.032	0.138	0.206	0.450
JLs-1	25/06/2018	15.372	0.343	0.032	0.139	0.348	0.506
JLs-1	25/06/2018	15.422	0.343	0.032	0.137	0.331	0.432
JLs-1	25/06/2018	15.406	0.342	0.032	0.141	0.240	0.430
JLs-1	25/06/2018	15.401	0.343	0.032	0.140	0.279	0.489
JLs-1	25/06/2018	15.384	0.342	0.032	0.138	0.391	0.471
JLs-1	06/07/2018	15.439	0.338	0.032	0.138	0.397	0.426
JLs-1	06/07/2018	15.312	0.338	0.032	0.138	0.728	0.365
JLs-1	06/07/2018	15.392	0.338	0.032	0.140	0.661	0.378
JLs-1	06/07/2018	15.407	0.337	0.032	0.139	0.512	0.453
JLs-1	06/07/2018	15.348	0.337	0.031	0.140	0.339	0.478
JLs-1	06/07/2018	15.407	0.338	0.032	0.137	0.412	0.415
JLs-1	06/07/2018	15.396	0.338	0.032	0.141	0.572	0.371
JLs-1	06/07/2018	15.391	0.338	0.032	0.140	0.472	0.331
JLs-1	06/07/2018	15.366	0.338	0.031	0.140	0.409	0.275
JLs-1	06/07/2018	15.348	0.337	0.031	0.143	0.471	0.396
JLs-1	06/07/2018	15.367	0.337	0.032	0.139	0.382	0.324
JLs-1	06/07/2018	15.382	0.337	0.032	0.137	0.574	0.492
JLs-1	02/08/2018	15.384	0.339	0.032	0.138	0.467	0.402
JLs-1	02/08/2018	15.307	0.337	0.031	0.140	0.344	0.420
JLs-1	02/08/2018	15.331	0.337	0.031	0.141	0.365	0.384
JLs-1	02/08/2018	15.371	0.338	0.031	0.141	0.363	0.364
JLs-1	02/08/2018	15.311	0.336	0.031	0.140	0.345	0.349
JLs-1	02/08/2018	15.357	0.336	0.032	0.136	0.309	0.345

		Mg/Ca mmol/mol	Sr/Ca mmol/mol	Mn/Ca mmol/mol	Fe/Ca mmol/mol	S/Ca mmol/mol	P/Ca mmol/mol
**should**							
**bias %**							
**avg**		**2.472**	**3.044**	**0.043**	**1.110**	**1.731**	**0.388**
**2ds**		**0.017**	**0.013**	**0.001**	**0.059**	**0.182**	**0.167**
**2rsd**		**0.67**	**0.44**	**2.2**	**5.3**	**11**	**43**
**n**		**48**	**48**	**48**	**48**	**48**	**48**
AK	01/06/2018	2.481	3.052	0.042	1.078	1.645	0.265
AK	01/06/2018	2.475	3.045	0.042	1.070	1.707	0.347
AK	01/06/2018	2.470	3.044	0.042	1.074	1.640	0.468
AK	01/06/2018	2.467	3.029	0.043	1.069	1.709	0.429
AK	01/06/2018	2.473	3.041	0.042	1.069	1.750	0.369
AK	01/06/2018	2.478	3.041	0.043	1.069	1.721	0.376
AK	01/06/2018	2.480	3.050	0.043	1.076	1.615	0.366
AK	01/06/2018	2.472	3.032	0.043	1.066	1.746	0.418
AK	04/06/2018	2.487	3.064	0.042	1.088	1.857	0.240
AK	04/06/2018	2.469	3.046	0.043	1.089	1.806	0.290
AK	04/06/2018	2.471	3.051	0.043	1.089	1.696	0.465
AK	04/06/2018	2.479	3.049	0.042	1.082	1.560	0.413
AK	04/06/2018	2.474	3.042	0.043	1.087	1.786	0.482
AK	04/06/2018	2.469	3.047	0.042	1.095	1.788	0.552
AK	04/06/2018	2.478	3.048	0.042	1.088	1.746	0.452
AK	04/06/2018	2.478	3.056	0.043	1.086	1.688	0.561
AK	08/06/2018	2.473	3.044	0.044	1.094	1.753	0.457
AK	08/06/2018	2.478	3.045	0.043	1.103	1.858	0.237
AK	08/06/2018	2.480	3.050	0.042	1.098	1.854	0.375
AK	08/06/2018	2.464	3.045	0.042	1.091	1.755	0.152
AK	08/06/2018	2.474	3.037	0.043	1.090	1.590	0.470
AK	08/06/2018	2.472	3.049	0.042	1.093	1.702	0.521
AK	08/06/2018	2.468	3.045	0.042	1.090	2.040	0.501
AK	08/06/2018	2.475	3.049	0.042	1.092	1.591	0.365
AK	25/06/2018	2.491	3.044	0.043	1.148	1.779	0.425
AK	25/06/2018	2.477	3.043	0.042	1.137	1.699	0.416
AK	25/06/2018	2.480	3.041	0.042	1.134	1.762	0.367
AK	25/06/2018	2.472	3.039	0.043	1.128	1.657	0.292
AK	25/06/2018	2.471	3.041	0.043	1.135	1.552	0.404
AK	25/06/2018	2.477	3.052	0.043	1.124	1.625	0.397
AK	25/06/2018	2.485	3.049	0.042	1.133	1.665	0.430
AK	25/06/2018	2.478	3.048	0.043	1.128	1.795	0.479
AK	06/07/2018	2.457	3.027	0.043	1.158	1.696	0.389
AK	06/07/2018	2.457	3.045	0.042	1.167	1.794	0.365
AK	06/07/2018	2.464	3.048	0.043	1.174	1.771	0.312
AK	06/07/2018	2.454	3.031	0.043	1.156	1.705	0.391
AK	06/07/2018	2.460	3.042	0.043	1.147	1.781	0.348
AK	06/07/2018	2.457	3.042	0.043	1.143	1.643	0.311
AK	06/07/2018	2.464	3.039	0.043	1.140	1.824	0.379
AK	06/07/2018	2.462	3.039	0.043	1.145	1.697	0.429
AK	02/08/2018	2.467	3.039	0.044	1.120	1.684	0.381
AK	02/08/2018	2.468	3.045	0.042	1.120	1.775	0.248
AK	02/08/2018	2.476	3.043	0.043	1.120	1.801	0.356
AK	02/08/2018	2.471	3.040	0.043	1.117	1.627	0.311
AK	02/08/2018	2.475	3.052	0.043	1.126	1.799	0.450
AK	02/08/2018	2.463	3.045	0.043	1.121	1.823	0.379

		Mg/Ca mmol/mol	Sr/Ca mmol/mol	Mn/Ca mmol/mol	Fe/Ca mmol/mol	S/Ca mmol/mol	P/Ca mmol/mol
**should**		**13.281**	**0.923**	**0.730**	**1.445**	**2.518**	**2.613**
**bias %**		**0.28**	**0.23**	**-0.27**	**-0.79**	**0.74**	**-0.34**
**avg**		**13.318**	**0.926**	**0.728**	**1.434**	**2.536**	**2.604**
**2ds**		**0.079**	**0.009**	**0.003**	**0.013**	**0.220**	**0.110**
**2rsd**		**0.59**	**0.97**	**0.43**	**0.91**	**9**	**4**
**n**		**24**	**24**	**24**	**24**	**24**	**24**
BCQC	01/06/2018	13.270	0.926	0.728	1.428	2.652	2.646
BCQC	01/06/2018	13.349	0.925	0.730	1.440	2.406	2.721
BCQC	01/06/2018	13.323	0.925	0.727	1.429	2.572	2.615
BCQC	01/06/2018	13.336	0.927	0.728	1.433	2.432	2.691
BCQC	04/06/2018	13.347	0.927	0.727	1.432	2.451	2.570
BCQC	04/06/2018	13.394	0.932	0.727	1.433	2.685	2.618
BCQC	04/06/2018	13.350	0.928	0.728	1.436	2.630	2.642
BCQC	04/06/2018	13.344	0.928	0.730	1.445	2.542	2.685
BCQC	08/06/2018	13.295	0.927	0.728	1.428	2.475	2.513
BCQC	08/06/2018	13.316	0.931	0.728	1.428	2.519	2.591
BCQC	08/06/2018	13.317	0.930	0.727	1.426	2.660	2.636
BCQC	08/06/2018	13.292	0.929	0.728	1.425	2.645	2.535
BCQC	25/06/2018	13.279	0.930	0.730	1.432	2.592	2.607
BCQC	25/06/2018	13.331	0.927	0.730	1.433	2.552	2.625
BCQC	25/06/2018	13.368	0.930	0.731	1.421	2.363	2.565
BCQC	25/06/2018	13.397	0.932	0.730	1.433	2.490	2.585
BCQC	06/07/2018	13.230	0.919	0.727	1.436	2.764	2.587
BCQC	06/07/2018	13.285	0.917	0.727	1.439	2.501	2.560
BCQC	06/07/2018	13.311	0.921	0.727	1.445	2.442	2.565
BCQC	06/07/2018	13.283	0.919	0.727	1.443	2.468	2.484
BCQC	02/08/2018	13.290	0.921	0.730	1.435	2.416	2.641
BCQC	02/08/2018	13.283	0.921	0.728	1.435	2.360	2.631
BCQC	02/08/2018	13.335	0.921	0.729	1.441	2.646	2.594
BCQC	02/08/2018	13.315	0.922	0.732	1.443	2.611	2.583

JLs-1	02/08/2018	15.353	0.338	0.031	0.138	0.317	0.400	AK	02/08/2018	2.466	3.041	0.043	1.120	1.708	0.439								
JLs-1	02/08/2018	15.295	0.337	0.033	0.137	0.570	0.389	AK	02/08/2018	2.487	3.041	0.043	1.128	1.823	0.331								
JLs-1	02/08/2018	15.370	0.337	0.033	0.135	0.465	0.321																
JLs-1	02/08/2018	15.379	0.338	0.033	0.136	0.316	0.454																
JLs-1	02/08/2018	15.345	0.338	0.032	0.132	0.387	0.333																
JLs-1	02/08/2018	15.408	0.338	0.032	0.134	0.412	0.338																
		**Mg/Ca mmol/mol**	**Sr/Ca mmol/mol**	**Mn/Ca mmol/mol**	**Fe/Ca mmol/mol**	**S/Ca mmol/mol**	**P/Ca mmol/mol**			**Mg/Ca mmol/mol**	**Sr/Ca mmol/mol**	**Mn/Ca mmol/mol**	**Fe/Ca mmol/mol**	**S/Ca mmol/mol**	**P/Ca mmol/mol**			**Mg/Ca mmol/mol**	**Sr/Ca mmol/mol**	**Mn/Ca mmol/mol**	**Fe/Ca mmol/mol**	**S/Ca mmol/mol**	**P/Ca mmol/mol**
**should**																		**13.26**	**1.831**	**0.726**	**1.433**	**5.004**	**5.179**
**bias %**																		**0.52**	**-0.16**	**0.19**	**-0.36**	**2.58**	**-0.42**
**avg**		**15.354**	**0.343**	**0.032**	**0.155**	**0.498**	**0.432**			**2.643**	**3.042**	**0.045**	**1.366**	**1.825**	**0.397**			**13.331**	**1.829**	**0.728**	**1.428**	**5.133**	**5.157**
**2sd**		**0.097**	**0.002**	**0.002**	**0.004**	**0.284**	**0.093**			**0.053**	**0.018**	**0.002**	**0.007**	**0.206**	**0.150**			**0.043**	**0.005**	**0.002**	**0.008**	**0.259**	**0.037**
**2rsd**		**0.63**	**0.59**	**4.82**	**2.61**	**57.05**	**21.62**			**2.00**	**0.59**	**3.77**	**0.51**	**11.29**	**37.77**			**0.32**	**0.27**	**0.28**	**0.57**	**5.04**	**0.72**
**n**		**24**	**24**	**24**	**24**	**24**	**24**			**16**	**16**	**16**	**16**	**16**	**16**			**8**	**8**	**8**	**8**	**8**	**8**
JLs-1	17/04/2019	15.366	0.3421	0.0321	0.155	0.33	0.42	AK	17/04/2019	2.665	3.0489	0.0453	1.362	1.72	0.45	BCQ2	17/04/2019	13.322	1.8304	0.7270	1.423	5.08	5.17
JLs-1	17/04/2019	15.264	0.3449	0.0322	0.156	0.43	0.36	AK	17/04/2019	2.669	3.0471	0.0452	1.362	1.75	0.36	BCQ2	17/04/2019	13.326	1.8325	0.7273	1.430	4.94	5.16
JLs-1	17/04/2019	15.367	0.3450	0.0319	0.154	0.64	0.41	AK	17/04/2019	2.670	3.0542	0.0462	1.365	1.86	0.42	BCQ2	17/04/2019	13.310	1.8249	0.7263	1.425	5.08	5.14
JLs-1	17/04/2019	15.308	0.3442	0.0332	0.156	0.72	0.57	AK	17/04/2019	2.671	3.0465	0.0463	1.370	1.96	0.48	BCQ2	17/04/2019	13.326	1.8307	0.7275	1.428	5.06	5.19
JLs-1	17/04/2019	15.342	0.3433	0.0328	0.154	0.61	0.47	AK	17/04/2019	2.667	3.0464	0.0453	1.365	1.84	0.43								
JLs-1	17/04/2019	15.312	0.3430	0.0333	0.157	0.43	0.41	AK	17/04/2019	2.661	3.0523	0.0463	1.362	1.73	0.43								
JLs-1	17/04/2019	15.311	0.3441	0.0330	0.154	0.47	0.50	AK	17/04/2019	2.666	3.0507	0.0441	1.369	1.87	0.46								
JLs-1	17/04/2019	15.287	0.3428	0.0328	0.157	0.62	0.45	AK	17/04/2019	2.665	3.0483	0.0459	1.363	1.74	0.40								
JLs-1	17/04/2019	15.339	0.3435	0.0325	0.156	0.31	0.41																
JLs-1	17/04/2019	15.339	0.3439	0.0311	0.156	0.27	0.41																
JLs-1	17/04/2019	15.322	0.3439	0.0317	0.154	0.49	0.45																
JLs-1	17/04/2019	15.362	0.3446	0.0327	0.155	0.30	0.43																
JLs-1	24/04/2019	15.439	0.3417	0.0334	0.159	0.37	0.42	AK	24/04/2019	2.638	3.0443	0.0460	1.370	1.64	0.32	BCQ2	24/04/2019	13.316	1.8261	0.7288	1.436	5.09	5.14
JLs-1	24/04/2019	15.272	0.3414	0.0311	0.157	0.59	0.51	AK	24/04/2019	2.626	3.0233	0.0442	1.366	1.73	0.36	BCQ2	24/04/2019	13.331	1.8279	0.7293	1.432	5.33	5.14
JLs-1	24/04/2019	15.349	0.3422	0.0311	0.158	0.32	0.41	AK	24/04/2019	2.627	3.0421	0.0441	1.369	1.90	0.53	BCQ2	24/04/2019	13.335	1.8277	0.7285	1.428	5.28	5.14
JLs-1	24/04/2019	15.365	0.3424	0.0314	0.151	0.42	0.46	AK	24/04/2019	2.630	3.0312	0.0437	1.371	1.96	0.46	BCQ2	24/04/2019	13.380	1.8281	0.7280	1.426	5.19	5.17
JLs-1	24/04/2019	15.401	0.3427	0.0308	0.154	0.39	0.41	AK	24/04/2019	2.602	3.0314	0.0445	1.364	1.87	0.39								
JLs-1	24/04/2019	15.405	0.3428	0.0311	0.152	0.76	0.43	AK	24/04/2019	2.608	3.0395	0.0447	1.369	1.79	0.26								
JLs-1	24/04/2019	15.363	0.3422	0.0317	0.154	0.54	0.38	AK	24/04/2019	2.604	3.0310	0.0447	1.365	2.01	0.34								
JLs-1	24/04/2019	15.336	0.3417	0.0321	0.159	0.67	0.40	AK	24/04/2019	2.617	3.0384	0.0450	1.360	1.84	0.27								
JLs-1	24/04/2019	15.395	0.3425	0.0319	0.156	0.60	0.37																
JLs-1	24/04/2019	15.422	0.3432	0.0319	0.155	0.60	0.46																
JLs-1	24/04/2019	15.397	0.3424	0.0320	0.152	0.57	0.39																
JLs-1	24/04/2019	15.431	0.3431	0.0317	0.155	0.49	0.42																

Table S3: Analytical results for isotope standards measured alongside the studied samples.

	date	$\delta^{13}C$ ‰ VPDB	$\delta^{18}O$ ‰ VPDB		date	% $CaCO_3$	$\delta^{13}C$ ‰ VPDB	$\delta^{18}O$ ‰ VPDB
**n**	**179**				**80**	**97.1**		
**2sd**		**0.06**	**0.14**			**1.5**	**0.09**	**0.33**
CAR	10/08/2018	2.10	-2.08	NCA	10/08/2018	97.2	-5.56	-21.98
CAR	10/08/2018	2.11	-1.99	NCA	10/08/2018	97.3	-5.59	-21.97
CAR	10/08/2018	2.11	-1.99	NCA	10/08/2018	97.7	-5.67	-22.02
CAR	10/08/2018	2.08	-2.03	NCA	10/08/2018	97.7	-5.58	-21.77
CAR	10/08/2018	2.11	-1.99	NCA	10/08/2018	97.8	-5.65	-22.01
CAR	10/08/2018	2.11	-2.15	NCA	10/08/2018	97.7	-5.64	-21.96
CAR	10/08/2018	2.12	-2.01	NCA	10/08/2018	97.8	-5.68	-21.83
CAR	10/08/2018	2.09	-2.02	NCA	10/08/2018	98.0	-5.68	-21.67
CAR	10/08/2018	2.08	-2.03					
CAR	10/08/2018	2.08	-1.96					
CAR	10/08/2018	2.16	-2.02					
CAR	10/08/2018	2.10	-2.08					
CAR	10/08/2018	2.08	-2.15					
CAR	10/08/2018	2.08	-1.94					
CAR	10/08/2018	2.14	-1.96					
CAR	10/08/2018	2.08	-2.06					
CAR	10/08/2018	2.10	-2.09					
CAR	10/08/2018	2.09	-1.99					
CAR	12/08/2018	2.13	-1.91	NCA	12/08/2018	96.7	-5.67	-22.11
CAR	12/08/2018	2.07	-2.15	NCA	12/08/2018	98.6	-5.62	-22.05
CAR	12/08/2018	2.12	-2.06	NCA	12/08/2018	96.1	-5.67	-21.87
CAR	12/08/2018	2.06	-2.04	NCA	12/08/2018	97.7	-5.55	-21.80
CAR	12/08/2018	2.12	-2.00	NCA	12/08/2018	98.2	-5.67	-22.00
CAR	12/08/2018	2.11	-2.01	NCA	12/08/2018	98.3	-5.65	-21.71
CAR	12/08/2018	2.14	-1.94	NCA	12/08/2018	98.2	-5.62	-21.73
CAR	12/08/2018	2.08	-2.07	NCA	12/08/2018	97.7	-5.59	-21.94
CAR	12/08/2018	2.10	-2.12					
CAR	12/08/2018	2.04	-2.08					
CAR	12/08/2018	2.08	-1.98					
CAR	12/08/2018	2.15	-1.99					
CAR	12/08/2018	2.18	-1.97					
CAR	12/08/2018	2.04	-2.07					
CAR	12/08/2018	2.05	-2.04					
CAR	12/08/2018	2.12	-2.05					
CAR	12/08/2018	2.11	-2.07					
CAR	12/08/2018	2.09	-1.99					
CAR	13/08/2018	2.09	-2.13	NCA	13/08/2018	96.0	-5.70	-22.22
CAR	13/08/2018	2.12	-1.92	NCA	13/08/2018	96.4	-5.62	-22.06
CAR	13/08/2018	2.10	-2.03	NCA	13/08/2018	97.0	-5.68	-21.96
CAR	13/08/2018	2.09	-2.01	NCA	13/08/2018	93.5	-5.62	-21.54
CAR	13/08/2018	2.12	-2.06	NCA	13/08/2018	96.9	-5.68	-22.16
CAR	13/08/2018	2.07	-2.09	NCA	13/08/2018	97.1	-5.56	-21.62
CAR	13/08/2018	2.13	-2.03	NCA	13/08/2018	96.8	-5.64	-22.03
CAR	13/08/2018	2.12	-1.94	NCA	13/08/2018	97.2	-5.53	-21.61
CAR	13/08/2018	2.07	-2.03					
CAR	13/08/2018	2.07	-2.13					
CAR	13/08/2018	2.13	-1.99					
CAR	13/08/2018	2.11	-1.95					
CAR	13/08/2018	2.08	-2.23					
CAR	13/08/2018	2.14	-1.92					
CAR	13/08/2018	2.07	-1.98					
CAR	13/08/2018	2.11	-2.07					
CAR	13/08/2018	2.10	-1.87					
CAR	13/08/2018	2.10	-2.17					
CAR	14/08/2018	2.09	-1.94	NCA	14/08/2018	97.2	-5.62	-22.08
CAR	14/08/2018	2.12	-2.11	NCA	14/08/2018	97.0	-5.67	-22.05
CAR	14/08/2018	2.07	-2.07	NCA	14/08/2018	97.6	-5.58	-21.87
CAR	14/08/2018	2.10	-2.07	NCA	14/08/2018	97.4	-5.69	-21.95
CAR	14/08/2018	2.16	-1.97	NCA	14/08/2018	97.6	-5.64	-21.82
CAR	14/08/2018	2.08	-1.88	NCA	14/08/2018	97.9	-5.63	-21.83
CAR	14/08/2018	2.05	-2.26	NCA	14/08/2018	97.5	-5.62	-21.80
CAR	14/08/2018	2.11	-2.02	NCA	14/08/2018	97.4	-5.59	-21.81
CAR	14/08/2018	2.07	-1.97					
CAR	14/08/2018	2.15	-1.97					
CAR	14/08/2018	2.07	-2.05					

CAR	14/08/2018	2.11	-2.04					
CAR	14/08/2018	2.08	-2.15					
CAR	14/08/2018	2.15	-1.88					
CAR	14/08/2018	2.06	-2.04					
CAR	14/08/2018	2.12	-2.05					
CAR	14/08/2018	2.10	-2.01					
CAR	14/08/2018	2.10	-2.04					
CAR	15/08/2018	2.11	-1.98	NCA	15/08/2018	95.7	-5.67	-22.10
CAR	15/08/2018	2.12	-2.07	NCA	15/08/2018	96.1	-5.58	-21.94
CAR	15/08/2018	2.07	-2.08	NCA	15/08/2018	96.9	-5.67	-22.16
CAR	15/08/2018	2.05	-2.03	NCA	15/08/2018	96.6	-5.63	-21.90
CAR	15/08/2018	2.17	-1.89	NCA	15/08/2018	95.9	-5.68	-21.76
CAR	15/08/2018	2.11	-2.10	NCA	15/08/2018	97.0	-5.49	-21.71
CAR	15/08/2018	2.12	-2.09	NCA	15/08/2018	97.0	-5.68	-21.82
CAR	15/08/2018	2.11	-1.96	NCA	15/08/2018	96.9	-5.65	-21.81
CAR	15/08/2018	2.09	-1.98					
CAR	15/08/2018	2.05	-2.17					
CAR	15/08/2018	2.03	-2.01					
CAR	15/08/2018	2.09	-1.97					
CAR	15/08/2018	2.19	-2.07					
CAR	15/08/2018	2.20	-1.99					
CAR	15/08/2018	2.04	-2.00					
CAR	15/08/2018	2.04	-2.08					
CAR	15/08/2018	2.09	-2.04					
CAR	15/08/2018	2.13	-2.02					
CAR	16/08/2018	2.07	-2.01	NCA	16/08/2018	96.4	-5.68	-22.17
CAR	16/08/2018	2.13	-2.04	NCA	16/08/2018	96.5	-5.65	-22.13
CAR	16/08/2018	2.10	-2.02	NCA	16/08/2018	97.6	-5.60	-21.90
CAR	16/08/2018	2.09	-2.09	NCA	16/08/2018	97.1	-5.59	-21.94
CAR	16/08/2018	2.10	-1.91	NCA	16/08/2018	97.1	-5.64	-21.87
CAR	16/08/2018	2.11	-2.10	NCA	16/08/2018	96.1	-5.61	-21.78
CAR	16/08/2018	2.12	-2.03	NCA	16/08/2018	97.5	-5.64	-21.72
CAR	16/08/2018	2.08	-2.04	NCA	16/08/2018	95.9	-5.62	-21.69
CAR	16/08/2018	2.09	-2.04					
CAR	16/08/2018	2.09	-2.01					
CAR	16/08/2018	2.12	-2.04					
CAR	16/08/2018	2.11	-2.10					
CAR	16/08/2018	2.01	-2.02					
CAR	16/08/2018	2.16	-1.94					
CAR	air leak							
CAR	16/08/2018	2.11	-2.06					
CAR	16/08/2018	2.07	-2.15					
CAR	16/08/2018	2.12	-1.91					
CAR	17/08/2018	2.09	-2.05	NCA	17/08/2018	96.8	-5.70	-22.18
CAR	17/08/2018	2.11	-2.00	NCA	17/08/2018	96.7	-5.69	-22.31
CAR	17/08/2018	2.08	-2.04	NCA	17/08/2018	97.5	-5.63	-21.91
CAR	17/08/2018	2.14	-2.02	NCA	17/08/2018	97.4	-5.60	-21.99
CAR	17/08/2018	2.03	-2.03	NCA	17/08/2018	97.0	-5.63	-21.68
CAR	17/08/2018	2.11	-2.09	NCA	17/08/2018	95.7	-5.61	-21.68
CAR	17/08/2018	2.11	-1.94	NCA	17/08/2018	97.6	-5.59	-21.82
CAR	17/08/2018	2.16	-2.00	NCA	17/08/2018	97.5	-5.59	-21.64
CAR	17/08/2018	2.10	-2.11					
CAR	17/08/2018	2.08	-2.03					
CAR	17/08/2018	2.10	-2.06					
CAR	17/08/2018	2.06	-2.02					
CAR	17/08/2018	2.10	-2.12					
CAR	17/08/2018	2.15	-1.86					
CAR	17/08/2018	2.07	-2.18					
CAR	17/08/2018	2.11	-1.94					
CAR	17/08/2018	2.10	-2.03					
CAR	17/08/2018	2.10	-2.03					
CAR	30/03/2019	2.08	-2.03	NCA	30/03/2019	96.1	-5.62	-22.04
CAR	30/03/2019	2.11	-2.04	NCA	30/03/2019	96.6	-5.60	-22.09
CAR	30/03/2019	2.09	-2.00	NCA	30/03/2019	97.7	-5.65	-21.87
CAR	30/03/2019	2.12	-2.03	NCA	30/03/2019	97.6	-5.68	-21.71
CAR	30/03/2019	2.12	-2.01	NCA	30/03/2019	97.9	-5.61	-21.88
CAR	30/03/2019	2.06	-2.09	NCA	30/03/2019	98.1	-5.60	-21.81
CAR	30/03/2019	2.11	-2.04	NCA	30/03/2019	97.0	-5.60	-21.91
CAR	30/03/2019	2.09	-2.00	NCA	30/03/2019	97.2	-5.68	-21.90
CAR	30/03/2019	2.11	-2.05					
CAR	30/03/2019	2.09	-2.01					

CAR	30/03/2019	2.14	-2.01					
CAR	30/03/2019	2.09	-2.04					
CAR	30/03/2019	2.06	-2.08					
CAR	30/03/2019	2.11	-1.99					
CAR	30/03/2019	2.08	-2.10					
CAR	30/03/2019	2.14	-1.96					
CAR	30/03/2019	2.09	-1.97					
CAR	30/03/2019	2.11	-2.08					
CAR	18/04/2019	2.08	-2.13	NCA	18/04/2019	98.0	-5.67	-22.05
CAR	18/04/2019	2.12	-1.95	NCA	18/04/2019	97.1	-5.63	-21.96
CAR	18/04/2019	2.12	-2.00	NCA	18/04/2019	97.5	-5.61	-22.03
CAR	18/04/2019	2.08	-1.93	NCA	18/04/2019	97.4	-5.63	-21.94
CAR	18/04/2019	2.08	-2.10	NCA	18/04/2019	97.6	-5.63	-21.85
CAR	18/04/2019	2.12	-2.19	NCA	18/04/2019	97.4	-5.64	-21.78
CAR	18/04/2019	2.09	-1.95	NCA	18/04/2019	97.9	-5.64	-21.83
CAR	18/04/2019	2.10	-2.03	NCA	18/04/2019	97.8	-5.60	-21.75
CAR	18/04/2019	2.08	-1.90					
CAR	18/04/2019	2.16	-2.03					
CAR	18/04/2019	2.00	-2.15					
CAR	18/04/2019	2.15	-1.96					
CAR	18/04/2019	2.07	-2.12					
CAR	18/04/2019	2.16	-2.06					
CAR	18/04/2019	2.10	-2.07					
CAR	18/04/2019	2.09	-1.87					
CAR	18/04/2019	2.05	-2.17					
CAR	18/04/2019	2.14	-1.94					
CAR	21/04/2019	2.08	-2.04	NCA	21/04/2019	97.4	-5.71	-22.15
CAR	21/04/2019	2.11	-2.00	NCA	21/04/2019	97.0	-5.71	-22.05
CAR	21/04/2019	2.13	-2.08	NCA	21/04/2019	97.5	-5.60	-22.02
CAR	21/04/2019	2.08	-2.04	NCA	21/04/2019	98.0	-5.64	-21.90
CAR	21/04/2019	2.06	-2.02	NCA	21/04/2019	97.5	-5.65	-22.00
CAR	21/04/2019	2.11	-1.94	NCA	21/04/2019	97.3	-5.62	-21.68
CAR	21/04/2019	2.10	-2.07	NCA	21/04/2019	97.9	-5.57	-21.81
CAR	21/04/2019	2.11	-2.04	NCA	21/04/2019	97.7	-5.55	-21.60
CAR	21/04/2019	2.10	-2.06					
CAR	21/04/2019	2.12	-2.04					
CAR	21/04/2019	2.10	-1.94					
CAR	21/04/2019	2.05	-2.09					
CAR	21/04/2019	2.11	-1.94					
CAR	21/04/2019	2.13	-2.09					
CAR	21/04/2019	2.12	-2.10					
CAR	21/04/2019	2.07	-2.01					
CAR	21/04/2019	2.06	-1.92					
CAR	21/04/2019	2.14	-2.12					

Table S4: Geochemical data acquired for fossils from Lower Toarcian sections in Spain and Portugal.

collection number	lab number	stratigraphy			sample ID	specimen number	species	type	description	prepared date	measured TE date	Ca wt%	Mg/Ca mmol/mol	Sr/Ca mmol/mol	Mn/Ca mmol/mol	Fe/Ca mmol/mol	S/Ca mmol/mol	P/Ca mmol/mol	measured ISO date	CaCO3 %	$\delta^{13}C$ ‰ VPDB	$\delta^{18}O$ ‰ VPDB
		locality	interval	height (m)																		
MPZ 2019/482	CVU_carb_3831	Barranco de la Cañada	BC22	26.70	1	1	*Homoeorhynchia meridionalis*	brachiopod	ventral	28/06/2018	06/07/2018	38.2	2.55	1.172	0.013	0.07	6.93	0.05	16/08/2018	96.9	3.06	-2.39
MPZ 2019/482	CVU_carb_3832	Barranco de la Cañada	BC22	26.70	1	1	*Homoeorhynchia meridionalis*	brachiopod	ventral	28/06/2018	06/07/2018	38.2	2.37	1.168	0.012	0.05	6.62	0.02	16/08/2018	97.8	2.96	-2.16
MPZ 2019/482	CVU_carb_3833	Barranco de la Cañada	BC22	26.70	1	1	*Homoeorhynchia meridionalis*	brachiopod	ventral	28/06/2018	06/07/2018	39.1	2.73	1.184	0.012	0.07	7.31	0.07	16/08/2018	97.5	3.00	-2.54
MPZ 2019/482	CVU_carb_3977	Barranco de la Cañada	BC22	26.70	1	1		calcite cement	calcite cement	19/07/2018	02/08/2018	37.6	11.78	0.298	0.648	6.91	1.07	0.03	17/08/2018	99.6	1.86	-10.41
MPZ 2019/479	CVU_carb_3826	Barranco de la Cañada	BC21	25.55	2	1	*Homoeorhynchia meridionalis*	brachiopod		28/06/2018	06/07/2018	38.3	3.71	1.114	0.033	0.29	6.46	0.04	15/08/2018	98.0	2.73	-2.95
MPZ 2019/479	CVU_carb_3827	Barranco de la Cañada	BC21	25.55	2	1	*Homoeorhynchia meridionalis*	brachiopod		28/06/2018	06/07/2018	38.5	5.23	1.164	0.047	0.38	6.75	0.04	15/08/2018	98.7	2.74	-2.73
MPZ 2019/480	CVU_carb_3828	Barranco de la Cañada	BC21	25.55	2	2	*Homoeorhynchia meridionalis*	brachiopod		28/06/2018	06/07/2018	38.1	4.74	1.194	0.035	0.37	7.23	0.07	16/08/2018	96.2	2.60	-2.92
MPZ 2019/481	CVU_carb_3829	Barranco de la Cañada	BC21	25.55	2	3	*Homoeorhynchia meridionalis*	brachiopod		28/06/2018	06/07/2018	38.2	2.41	1.183	0.010	0.10	6.24	0.08	16/08/2018	96.0	2.66	-2.23
MPZ 2019/481	CVU_carb_3830	Barranco de la Cañada	BC21	25.55	2	3		bulk rock	bulk rock	28/06/2018	06/07/2018	33.0	27.95	0.454	0.549	12.46	1.38	1.38	16/08/2018	85.4	2.06	-4.47
MPZ 2019/478	CVU_carb_3949	Barranco de la Cañada	BC21	25.55	1	1		brachiopod	terebratulid	18/07/2018	02/08/2018	39.3	9.01	0.886	0.040	0.35	3.45	0.10	17/08/2018	100.4	3.04	-1.81
MPZ 2019/476	CVU_carb_3815	Barranco de la Cañada	BC20	24.55	2	1	*Homoeorhynchia meridionalis*	brachiopod		28/06/2018	06/07/2018	38.3	4.26	1.206	0.029	0.28	7.32	0.13	15/08/2018	97.6	2.58	-2.13
MPZ 2019/476	CVU_carb_3816	Barranco de la Cañada	BC20	24.55	2	1	*Homoeorhynchia meridionalis*	brachiopod		28/06/2018	06/07/2018	38.7	3.66	1.095	0.055	0.30	6.86	0.01	15/08/2018	97.4	2.44	-2.02
MPZ 2019/476	CVU_carb_3817	Barranco de la Cañada	BC20	24.55	2	1	*Homoeorhynchia meridionalis*	brachiopod		28/06/2018	06/07/2018	38.4	3.63	1.189	0.026	0.17	7.26	0.00	15/08/2018	97.3	2.68	-1.93
MPZ 2019/476	CVU_carb_3818	Barranco de la Cañada	BC20	24.55	2	1		calcite cement	calcite cement	28/06/2018	06/07/2018	38.6	6.84	0.047	2.494	2.98	0.50	0.08	15/08/2018	99.9	1.46	-6.07
MPZ 2019/476	CVU_carb_3819	Barranco de la Cañada	BC20	24.55	2	1		bulk rock	bulk rock	28/06/2018	06/07/2018	34.9	24.86	0.436	0.471	11.34	1.42	0.25	15/08/2018	90.6	1.99	-3.95
MPZ 2019/477	CVU_carb_3820	Barranco de la Cañada	BC20	24.55	2	2	*Homoeorhynchia meridionalis*	brachiopod		28/06/2018	06/07/2018	38.6	3.78	1.230	0.020	0.17	6.14	0.00	15/08/2018	98.6	2.62	-2.60
MPZ 2019/473	CVU_carb_3821	Barranco de la Cañada	BC20	24.55	1	1	*Homoeorhynchia meridionalis*	brachiopod		28/06/2018	06/07/2018	38.3	3.31	1.145	0.014	0.14	6.37	-0.05	15/08/2018	98.0	3.25	-2.03
MPZ 2019/473	CVU_carb_3822	Barranco de la Cañada	BC20	24.55	1	1	*Homoeorhynchia meridionalis*	brachiopod		28/06/2018	06/07/2018	38.3	7.90	1.239	0.035	0.44	7.69	0.09	15/08/2018	98.3	3.06	-1.99
MPZ 2019/474	CVU_carb_3823	Barranco de la Cañada	BC20	24.55	1	2	*Homoeorhynchia meridionalis*	brachiopod		28/06/2018	06/07/2018	38.2	4.06	1.128	0.034	0.22	9.05	0.12	15/08/2018	98.4	2.60	-2.61
MPZ 2019/474	CVU_carb_3824	Barranco de la Cañada	BC20	24.55	1	2	*Homoeorhynchia meridionalis*	brachiopod		28/06/2018	06/07/2018	38.3	3.59	1.124	0.025	0.13	8.36	0.05	15/08/2018	98.2	2.57	-2.69
MPZ 2019/475	CVU_carb_3825	Barranco de la Cañada	BC20	24.55	1	3	*Homoeorhynchia meridionalis*	brachiopod		28/06/2018	06/07/2018	38.2	3.39	1.200	0.019	0.19	7.54	0.04	15/08/2018	98.0	2.64	-2.52
MPZ 2019/472	CVU_carb_3751	Barranco de la Cañada	BC19	23.45	1	1	*Homoeorhynchia batalleri*	brachiopod	dorsal	17/06/2018	25/06/2018	37.9	8.93	1.157	0.090	1.94	5.58	0.05	15/08/2018	96.9	2.11	-2.58
MPZ 2019/472	CVU_carb_3752	Barranco de la Cañada	BC19	23.45	1	1	*Homoeorhynchia batalleri*	brachiopod	dorsal	17/06/2018	25/06/2018	38.2	9.38	1.205	0.079	0.86	6.40	0.18	15/08/2018	97.9	1.83	-2.47
MPZ 2019/472	CVU_carb_3753	Barranco de la Cañada	BC19	23.45	1	1	*Homoeorhynchia batalleri*	brachiopod	ventral	17/06/2018	25/06/2018	38.4	7.69	1.107	0.079	0.83	5.26	0.18	15/08/2018	98.5	2.06	-2.47
MPZ 2019/472	CVU_carb_3754	Barranco de la Cañada	BC19	23.45	1	1		bulk rock	bulk rock from fossil fill	17/06/2018	25/06/2018	32.7	26.76	0.356	0.614	25.31	1.16	1.79	15/08/2018	85.4	1.68	-3.16
MPZ 2019/471	CVU_carb_3755	Barranco de la Cañada	BC18	22.65	1	1	*Homoeorhynchia batalleri*	brachiopod	dorsal	17/06/2018	25/06/2018	38.2	6.68	1.006	0.140	0.97	4.72	0.06	15/08/2018	98.2	2.29	-3.07
MPZ 2019/471	CVU_carb_3756	Barranco de la Cañada	BC18	22.65	1	1	*Homoeorhynchia batalleri*	brachiopod	dorsal	17/06/2018	25/06/2018	38.3	5.67	1.167	0.090	0.39	4.93	-0.08	15/08/2018	97.6	2.26	-2.84
MPZ 2019/471	CVU_carb_3757	Barranco de la Cañada	BC18	22.65	1	1	*Homoeorhynchia batalleri*	brachiopod	dorsal	17/06/2018	25/06/2018	38.3	6.02	1.222	0.094	0.40	5.26	0.09	15/08/2018	98.8	2.16	-2.73
MPZ 2019/471	CVU_carb_3758	Barranco de la Cañada	BC18	22.65	1	1	*Homoeorhynchia batalleri*	brachiopod	dorsal	17/06/2018	25/06/2018	38.1	5.44	1.217	0.083	0.33	5.01	0.07	15/08/2018	97.9	2.49	-2.55
MPZ 2019/471	CVU_carb_3759	Barranco de la Cañada	BC18	22.65	1	1	*Homoeorhynchia batalleri*	brachiopod	dorsal	17/06/2018	25/06/2018	38.2	5.62	1.113	0.104	0.45	5.05	0.18	15/08/2018	97.5	2.33	-2.57
MPZ 2019/471	CVU_carb_3760	Barranco de la Cañada	BC18	22.65	1	1	*Homoeorhynchia batalleri*	brachiopod	dorsal	17/06/2018	25/06/2018	38.2	5.81	1.165	0.109	0.67	4.83	0.04	15/08/2018	99.8	2.26	-2.95
MPZ 2019/471	CVU_carb_3761	Barranco de la Cañada	BC18	22.65	1	1		bulk rock	bulk rock from fossil fill	17/06/2018	25/06/2018	32.3	93.17	0.357	0.876	22.22	1.28	0.02	15/08/2018	88.1	2.08	-6.35
MPZ 2019/470	CVU_carb_3681	Barranco de la Cañada	BC17	21.45	1	1	*Choffatirhynchia vasconcellosi*	brachiopod	ventral	07/06/2018	25/06/2018	37.9	6.33	1.243	0.027	0.12	4.60	0.10	14/08/2018	98.8	2.53	-2.05
MPZ 2019/470	CVU_carb_3682	Barranco de la Cañada	BC17	21.45	1	1	*Choffatirhynchia vasconcellosi*	brachiopod	ventral	07/06/2018	25/06/2018	38.6	5.57	1.182	0.026	0.14	4.34	0.23	14/08/2018	98.9	2.50	-2.23
MPZ 2019/470	CVU_carb_3683	Barranco de la Cañada	BC17	21.45	1	1	*Choffatirhynchia vasconcellosi*	brachiopod	ventral	07/06/2018	25/06/2018	38.5	5.43	1.134	0.031	0.14	4.67	0.16	14/08/2018	98.6	2.35	-2.03
MPZ 2019/470	CVU_carb_3684	Barranco de la Cañada	BC17	21.45	1	1	*Choffatirhynchia vasconcellosi*	brachiopod	dorsal	07/06/2018	25/06/2018	38.1	5.47	1.138	0.041	0.38	4.47	0.12	14/08/2018	98.4	2.51	-2.07
MPZ 2019/470	CVU_carb_3685	Barranco de la Cañada	BC17	21.45	1	1	*Choffatirhynchia vasconcellosi*	brachiopod	dorsal	08/06/2018	25/06/2018	38.4	5.60	1.123	0.041	0.37	4.65	0.05	14/08/2018	98.1	2.39	-1.96
MPZ 2019/470	CVU_carb_3686	Barranco de la Cañada	BC17	21.45	1	1	*Choffatirhynchia vasconcellosi*	brachiopod	dorsal	08/06/2018	25/06/2018	38.1	5.55	1.152	0.033	0.16	4.49	0.06	14/08/2018	97.5	2.46	-2.08
MPZ 2019/470	CVU_carb_3687	Barranco de la Cañada	BC17	21.45	1	1		bulk rock	bulk rock, fossil interior	08/06/2018	25/06/2018	33.8	22.30	0.338	0.564	23.38	0.83	0.81	14/08/2018	86.7	1.88	-3.03
MPZ 2019/469	CVU_carb_3931	Barranco de la Cañada	BC15	19.85	1	1	*Gryphaea* cf. *dumortieri*	bivalve		09/07/2018	02/08/2018	37.7	10.34	0.725	0.362	4.06	2.16	0.37	17/08/2018	96.5	2.93	-1.85
MPZ 2019/469	CVU_carb_3932	Barranco de la Cañada	BC15	19.85	1	1		bulk rock	bulk rock	09/07/2018	02/08/2018	28.7	37.76	0.534	0.680	23.27	1.41	0.89	17/08/2018	76.0	2.30	-4.23
MPZ 2019/467	CVU_carb_3784	Barranco de la Cañada	BC14	19.35	2	1	*Homoeorhynchia meridionalis*	brachiopod		22/06/2018	25/06/2018	38.0	5.28	1.310	0.017	0.10	4.60	0.13	15/08/2018	98.2	3.12	-2.89
MPZ 2019/467	CVU_carb_3785	Barranco de la Cañada	BC14	19.35	2	1	*Homoeorhynchia meridionalis*	brachiopod		22/06/2018	25/06/2018	38.2	3.83	1.181	0.022	0.08	4.86	0.10	15/08/2018	95.6	3.53	-2.92
MPZ 2019/467	CVU_carb_3786	Barranco de la Cañada	BC14	19.35	2	1	*Homoeorhynchia meridionalis*	brachiopod	other valve than 3784-3785	22/06/2018	25/06/2018	38.3	4.28	1.115	0.044	0.16	4.73	0.08	15/08/2018	98.1	3.39	-2.65
MPZ 2019/467	CVU_carb_3787	Barranco de la Cañada	BC14	19.35	2	1	*Homoeorhynchia meridionalis*	brachiopod	other valve than 3784-3785	22/06/2018	25/06/2018	38.0	4.15	1.097	0.050	0.20	4.84	0.21	15/08/2018	98.1	3.21	-2.95
MPZ 2019/468	CVU_carb_3788	Barranco de la Cañada	BC14	19.35	2	2	*Homoeorhynchia meridionalis*	brachiopod	ventral	22/06/2018	25/06/2018	37.9	7.64	1.282	0.017	0.08	7.18	0.09	15/08/2018	98.0	3.14	-2.52
MPZ 2019/468	CVU_carb_3789	Barranco de la Cañada	BC14	19.35	2	2	*Homoeorhynchia meridionalis*	brachiopod	ventral	22/06/2018	25/06/2018	38.0	6.35	1.235	0.022	0.13	6.65	0.09	15/08/2018	97.6	2.93	-2.51
MPZ 2019/468	CVU_carb_3790	Barranco de la Cañada	BC14	19.35	2	2	*Homoeorhynchia meridionalis*	brachiopod	dorsal	22/06/2018	25/06/2018	38.1	5.28	1.197	0.031	0.21	5.78	0.12	15/08/2018	97.8	3.13	-2.44
MPZ 2019/468	CVU_carb_3791	Barranco de la Cañada	BC14	19.35	2	2		bulk rock	bulk rock	22/06/2018	25/06/2018	31.0	28.78	0.446	0.659	18.95	0.95	0.41	15/08/2018	80.6	2.71	-4.34
MPZ 2019/465	CVU_carb_3889	Barranco de la Cañada	BC14	19.35	1	1	*Gryphaea* cf. *dumortieri*	bivalve		02/07/2018	06/07/2018	38.8	3.51	0.745	0.088	0.79	1.21	0.08	16/08/2018	100.1	3.34	-2.28
MPZ 2019/465	CVU_carb_3890	Barranco de la Cañada	BC14	19.35	1	1	*Gryphaea* cf. *dumortieri*	bivalve		02/07/2018	06/07/2018	39.0	3.23	0.803	0.117	1.06	1.04	0.08	16/08/2018	100.6	3.53	-1.94
MPZ 2019/465	CVU_carb_3891	Barranco de la Cañada	BC14	19.35	1	1	*Gryphaea* cf. *dumortieri*	bivalve		02/07/2018	06/07/2018	39.0	3.06	0.767	0.120	0.93	0.92	0.12	16/08/2018	100.1	3.47	-2.06
MPZ 2019/466	CVU_carb_3892	Barranco de la Cañada	BC14	19.35	1	2	*Gryphaea* cf. *dumortieri*	bivalve		02/07/2018	06/07/2018	38.8	2.30	0.777	0.033	0.26	0.75	0.20	16/08/2018	101.3	3.70	-2.17
MPZ 2019/466	CVU_carb_3893	Barranco de la Cañada	BC14	19.35	1	2	*Gryphaea* cf. *dumortieri*	bivalve		02/07/2018	06/07/2018	39.0	2.36	0.806	0.033	0.29	0.83	0.17	16/08/2018	99.6	4.15	-2.55
MPZ 2019/466	CVU_carb_3894	Barranco de la Cañada	BC14	19.35	1	2	*Gryphaea* cf. *dumortieri*	bivalve		02/07/2018	06/07/2018	39.0	3.25	0.779	0.042	0.26	1.89	0.10	16/08/2018	100.7	4.21	-2.82
MPZ 2019/462	CVU_carb_3742	Barranco de la Cañada	BC13	18.85	1	1	*Lobothyris hispanica*	brachiopod		17/06/2018	25/06/2018	38.3	6.31	0.923	0.119	1.01	2.16	0.09	14/08/2018	99.1	3.24	-2.65
MPZ 2019/462	CVU_carb_3743	Barranco de la Cañada	BC13	18.85	1	1	*Lobothyris hispanica*	brachiopod		17/06/2018	25/06/2018	38.0	8.71	1.204	0.108	0.82	3.59	0.08	14/08/2018	97.1	2.83	-2.61
MPZ 2019/462	CVU_carb_3744	Barranco de la Cañada	BC13	18.85	1	1	*Lobothyris hispanica*	brachiopod		17/06/2018	25/06/2018	38.4	4.88	1.028	0.102	0.55	2.46	0.05	14/08/2018	99.2	2.86	-2.64
MPZ 2019/462	CVU_carb_3745	Barranco de la Cañada	BC13	18.85	1	1		bulk rock	bulk rock	17/06/2018	25/06/2018	30.3	29.39	0.477	0.666	19.19	0.92	0.29	14/08/2018	79.7	2.60	-4.76
MPZ 2019/463	CVU_carb_3746	Barranco de la Cañada	BC13	18.85	1	2	*Homoeorhynchia meridionalis*	brachiopod		17/06/2018	25/06/2018	38.1	6.07	1.098	0.209	1.38	5.96	0.20	14/08/2018	98.4	2.93	-2.40
MPZ 2019/463	CVU_carb_3747	Barranco de la Cañada	BC13	18.85	1	2	*Homoeorhynchia meridionalis*	brachiopod		17/06/2018	25/06/2018	37.9	6.03	1.068	0.216	1.99	5.45	0.17	14/08/2018	98.3	3.07	-2.59
MPZ 2019/464	CVU_carb_3748	Barranco de la Cañada	BC13	18.85	1	3	*Homoeorhynchia meridionalis*	brachiopod		17/06/2018	25/06/2018	38.2	5.77	1.136	0.139	1.18	7.70	0.17	15/08/2018	96.2	3.21	-2.75
MPZ 2019/464	CVU_carb_3749	Barranco de la Cañada	BC13	18.85	1	3	*Homoeorhynchia meridionalis*	brachiopod		17/06/2018	25/06/2018	38.1	5.83	1.126	0.122	1.09	7.23	0.10	15/08/2018	97.8	3.42	-2.51
MPZ 2019/464	CVU_carb_3750	Barranco de la Cañada	BC13	18.85	1	3	*Homoeorhynchia meridionalis*	brachiopod		17/06/2018	25/06/2018	38.4	7.06	1.167	0.128	1.44	8.65	0.10	15/08/2018	96.3	3.29	-2.78
MPZ 2019/461	CVU_carb_3801	Barranco de la Cañada	BC12	18.35	1	1	*Homoeorhynchia meridionalis*	brachiopod		28/06/2018	06/07/2018	37.7	7.59	1.094	0.161	0.94	6.84	0.23	15/08/2018	97.9	3.00	-3.35
MPZ 2019/461	CVU_carb_3802	Barranco de la Cañada	BC12	18.35	1	1	*Homoeorhynchia meridionalis*	brachiopod		28/06/2018	06/07/2018	38.6	6.18	1.160	0.116	0.52	7.10	0.08	15/08/2018	97.8	3.10	-3.23
MPZ 2019/461	CVU_carb_3803	Barranco de la Cañada	BC12	18.35	1	1		bulk rock	bulk rock	28/06/2018	06/07/2018	31.8	30.74	0.480	0.770	20.89	1.86	5.72	15/08/2018	81.3	2.64	-4.33
MPZ 2019/458	CVU_carb_3933	Barranco de la Cañada	BC10	17.30	1	1	*Gryphaea* (*B.*) *sublobata*	bivalve		09/07/2018	02/08/2018	39.0	2.58	0.772	0.034	0.31	0.88	0.13	17/08/2018	100.8	3.75	-2.29
MPZ 2019/458	CVU_carb_3934	Barranco de la Cañada	BC10	17.30	1	1	*Gryphaea* (*B.*) *sublobata*	bivalve		09/07/2018	02/08/2018	38.9	2.37	0.781	0.032	0.25	1.04	0.09	17/08/2018	100.4	3.73	-2.35
MPZ 2019/458	CVU_carb_3935	Barranco de la Cañada	BC10	17.30	1	1	*Gryphaea* (*B.*) *sublobata*	bivalve		09/07/2018	02/08/2018	39.2	1.94	0.764	0.041	0.36	0.97	0.12	17/08/2018	101.0	4.01	-2.75
MPZ 2019/458	CVU_carb_3936	Barranco de la Cañada	BC10	17.30	1	1	*Gryphaea* (*B.*) *sublobata*	bivalve		09/07/2018	02/08/2018	39.1	2.29	0.793	0.038	0.29	1.33	0.15	17/08/2018	101.1	3.95	-2.64
MPZ 2019/458	CVU_carb_3937	Barranco de la Cañada	BC10	17.30	1	1	*Gryphaea* (*B.*) *sublobata*	bivalve		09/07/2018	02/08/2018	38.9	2.64	0.800	0.146	0.66	1.54	0.11	17/08/2018	100.3	4.06	-2.96
MPZ 2019/459	CVU_carb_3938	Barranco de la Cañada	BC10	17.30	1	2		bulk rock	bulk rock	09/07/2018	02/08/2018	29.4	35.00	0.479	0.735	24.66	1.22	5.65	17/08/2018	75.6	2.52	-4.20
MPZ 2019/456	CVU_carb_3970	Barranco de la Cañada	BC09	16.70	1	1	*Gryphaea* (*B.*) *sublobata*	bivalve		19/07/2018	02/08/2018	38.7	4.12	0.776	0.170	0.80	1.19	0.19	17/08/2018	101.3	3.85	-2.53
MPZ 2019/456	CVU_carb_3971	Barranco de la Cañada	BC09	16.70	1	1	*Gryphaea* (*B.*) *sublobata*	bivalve		19/07/2018	02/08/2018	38.5	3.79	0.757	0.212	1.04	1.14	0.03	17/08/2018	100.3	4.01	-2.39
MPZ 2019/456	CVU_carb_3972	Barranco de la Cañada	BC09	16.70	1	1	*Gryphaea* (*B.*) *sublobata*	bivalve		19/07/2018	02/08/2018	38.9	4.03	0.768	0.188	0.86	1.42	0.27	17/08/2018	100.7	3.86	-2.65
MPZ 2019/456	CVU_carb_3973	Barranco de la Cañada	BC09	16.70	1	1		bulk rock	bulk rock	19/07/2018	02/08/2018	27.6	36.06	0.587	0.712	24.39	1.03	1.65	17/08/2018	73.9	2.75	-4.40
MPZ 2019/457	CVU_carb_3974	Barranco de la Cañada	BC09	16.70	1	2	*Gryphaea* (*B.*) *sublobata*	bivalve		19/07/2018	02/08/2018	38.8	4.62	0.726	0.076	0.37	2.09	0.14	17/08/2018	100.6	3.84	-2.15
MPZ 2019/457	CVU_carb_3975	Barranco de la Cañada	BC09	16.70	1	2	*Gryphaea* (*B.*) *sublobata*	bivalve		19/07/2018	02/08/2018	38.7	5.40	0.706	0.129	0.63	3.34	0.25	17/08/2018	98.6	3.80	-2.14
MPZ 2019/457	CVU_carb_3976	Barranco de la Cañada	BC09	16.70	1	2	*Gryphaea* (*B.*) *sublobata*	bivalve		19/07/2018	02/08/2018	38.6	3.98	0.754	0.082	0.54	1.95	0.17	17/08/2018	101.2	3.75	-2.15
MPZ 2019/454	CVU_carb_3436	Barranco de la Cañada	BC08	16.05	3	1		calcite cement	sparitic calcite	10/04/2018	04/06/2018	38.5	7.20	0.305	0.802	8.02	0.15	-0.04	10/08/2018	100.9	2.34	-9.70
MPZ 2019/454	CVU_carb_3491	Barranco de la Cañada	BC08	16.05	3	1	*Homoeorhynchia meridionalis*	brachiopod	ventral	29/04/2018	04/06/2018	38.4	4.78	0.988	0.078	0.40	3.58	0.15	10/08/2018	99.3	3.33	-3.08
MPZ 2019/454	CVU_carb_3492	Barranco de la Cañada	BC08	16.05	3	1	*Homoeorhynchia meridionalis*	brachiopod	ventral	29/04/2018	04/06/2018	38.2	5.86	0.924	0.124	0.56	3.43	0.06	10/08/2018	99.8	3.11	-3.08
MPZ 2019/454	CVU_carb_3493	Barranco de la Cañada	BC08	16.05	3	1	*Homoeorhynchia meridionalis*	brachiopod	ventral	29/04/2018	04/06/2018	38.5	2.93	1.065	0.056	0.17	2.91	0.12	10/08/2018	99.4	3.59	-3.20
MPZ 2019/454	CVU_carb_3494	Barranco de la Cañada	BC08	16.05	3	1	*Homoeorhynchia meridionalis*	brachiopod	ventral	29/04/2018	04/06/2018	38.7	4.11	0.624	0.325	4.52	2.32	0.07	10/08/2018	100.5	2.78	-4.49
MPZ 2019/454	CVU_carb_3495	Barranco de la Cañada	BC08	16.05	3	1		calcite cement	calcite cement	29/04/2018	04/06/2018	38.4	9.05	0.331	0.841	8.14	0.27	0.06	10/08/2018	99.6	2.22	-9.49
MPZ 2019/454	CVU_carb_3496	Barranco de la Cañada	BC08	16.05	3	1		calcite cement	calcite cement	29/04/2018	04/06/2018	38.3	9.62	0.439	0.798	7.55	0.50	-0.04	10/08/2018	100.4	2.21	-10.34
MPZ 2019/455	CVU_carb_3497	Barranco de la Cañada	BC08	16.05	3	2	*Homoeorhynchia meridionalis*	brachiopod	ventral	29/04/2018	04/06/2018	38.3	4.27	1.140	0.037	0.14	3.88	0.01	10/08/2018	98.9	3.60	-2.58
MPZ 2019/455	CVU_carb_3498	Barranco de la Cañada	BC08	16.05	3	2	*Homoeorhynchia meridionalis*	brachiopod	ventral	29/04/2018	04/06/2018	37.9	4.14	1.124	0.030	0.09	4.08	0.04	10/08/2018	99.8	3.25	-2.83
MPZ 2019/455	CVU_carb_3499	Barranco de la Cañada	BC08	16.05	3	2	*Homoeorhynchia meridionalis*	brachiopod	dorsal	29/04/2018	04/06/2018	38.5	4.45	1.149	0.040	0.17	3.64	0.09	10/08/2018	99.4	3.08	-2.65
MPZ 2019/455	CVU_carb_3500	Barranco de la Cañada	BC08	16.05	3	2	*Homoeorhynchia meridionalis*	brachiopod	dorsal	29/04/2018	04/06/2018	38.0	4.76	1.139	0.039	0.15	4.04	0.10	10/08/2018	100.1	3.15	-2.61
MPZ 2019/455	CVU_carb_3501	Barranco de la Cañada	BC08	16.05	3	2	*Homoeorhynchia meridionalis*	brachiopod	dorsal	29/04/2018	04/06/2018	38.6	5.08	1.159	0.050	0.28	3.87	-0.07	10/08/2018	98.1	3.31	-2.56
MPZ 2019/452	CVU_carb_3773	Barranco de la Cañada	BC08	16.05	1	1	*Choffatirhynchia vasconcellosi*	brachiopod	ventral	17/06/2018	25/06/2018	38.2	8.26	1.020	0.385	2.27	4.81	1.39	15/08/2018	96.7	3.55	-2.63
MPZ 2019/452	CVU_carb_3774	Barranco de la Cañada	BC08	16.05	1	1	*Choffatirhynchia vasconcellosi*	brachiopod	ventral	17/06/2018	25/06/2018	38.3	7.75	1.100	0.157	0.76	5.19	0.29	15/08/2018	97.6	3.61	-2.91
MPZ 2019/453	CVU_carb_3775	Barranco de la Cañada	BC08	16.05	2	1	*Homoeorhynchia batalleri*	brachiopod	ventral	17/06/2018	25/06/2018	38.5	3.65	1.082	0.045	0.11	3.09	0.16	15/08/2018	98.4	3.84	-2.43

MPZ 2019/453	CVU_carb_3776	Barranco de la Cañada	BC08	16.05	2	1	*Homoeorhynchia batalleri*	brachiopod	ventral	17/06/2018	25/06/2018	38.3	3.53	1.071	0.044	0.08	2.91	0.17	15/08/2018	98.0	3.67	-2.22
MPZ 2019/453	CVU_carb_3777	Barranco de la Cañada	BC08	16.05	2	1	*Homoeorhynchia batalleri*	brachiopod	ventral	17/06/2018	25/06/2018	38.4	4.14	1.059	0.097	0.28	3.00	0.14	15/08/2018	97.9	3.64	-2.23
MPZ 2019/453	CVU_carb_3778	Barranco de la Cañada	BC08	16.05	2	1	*Homoeorhynchia batalleri*	brachiopod	dorsal	17/06/2018	25/06/2018	38.1	3.70	1.060	0.038	0.07	3.40	0.07	15/08/2018	98.9	3.76	-2.22
MPZ 2019/453	CVU_carb_3779	Barranco de la Cañada	BC08	16.05	2	1	*Homoeorhynchia batalleri*	brachiopod	dorsal	17/06/2018	25/06/2018	38.5	4.16	1.094	0.059	0.12	3.32	0.16	15/08/2018	98.8	3.16	-2.36
MPZ 2019/453	CVU_carb_3780	Barranco de la Cañada	BC08	16.05	2	1	*Homoeorhynchia batalleri*	brachiopod	dorsal	17/06/2018	25/06/2018	38.3	3.66	1.103	0.045	0.06	3.18	0.19	15/08/2018	99.3	3.70	-2.47
MPZ 2019/452	CVU_carb_3781	Barranco de la Cañada	BC08	16.05	1	1		bulk rock	bulk rock from fossil fill	17/06/2018	25/06/2018	34.2	26.15	0.543	0.672	20.59	3.02	26.79	15/08/2018	85.3	2.98	-3.66
MPZ 2019/449	CVU_carb_3430	Barranco de la Cañada	BC07	15.25	1	2	*Homoeorhynchia meridionalis*	brachiopod		10/04/2018	01/06/2018	38.1	3.42	1.019	0.045	0.34	2.42	0.13	10/08/2018	98.5	4.05	-2.59
MPZ 2019/448	CVU_carb_3688	Barranco de la Cañada	BC07	15.25	1	1	*Homoeorhynchia meridionalis*	brachiopod		08/06/2018	25/06/2018	38.6	6.20	0.981	0.121	0.69	3.37	0.16	14/08/2018	98.9	4.34	-2.38
MPZ 2019/448	CVU_carb_3689	Barranco de la Cañada	BC07	15.25	1	1	*Homoeorhynchia meridionalis*	brachiopod		08/06/2018	25/06/2018	38.4	6.35	0.984	0.133	0.71	3.56	0.19	14/08/2018	99.5	4.30	-2.70
MPZ 2019/448	CVU_carb_3690	Barranco de la Cañada	BC07	15.25	1	1	*Homoeorhynchia meridionalis*	brachiopod		08/06/2018	25/06/2018	38.7	4.27	0.932	0.135	0.42	3.19	0.10	14/08/2018	99.4	4.39	-2.73
MPZ 2019/448	CVU_carb_3691	Barranco de la Cañada	BC07	15.25	1	1		bulk rock	bulk rock	08/06/2018	25/06/2018	30.1	31.55	0.460	0.762	22.96	0.97	0.94	14/08/2018	78.3	3.08	-4.06
MPZ 2019/449	CVU_carb_3692	Barranco de la Cañada	BC07	15.25	1	2	*Homoeorhynchia meridionalis*	brachiopod	ventral	08/06/2018	25/06/2018	38.8	4.17	0.982	0.070	0.29	2.70	0.13	14/08/2018	98.1	4.22	-2.81
MPZ 2019/449	CVU_carb_3693	Barranco de la Cañada	BC07	15.25	1	2	*Homoeorhynchia meridionalis*	brachiopod	ventral	08/06/2018	25/06/2018	38.7	4.39	0.965	0.096	0.60	2.80	-0.02	14/08/2018	99.2	4.27	-2.77
MPZ 2019/449	CVU_carb_3694	Barranco de la Cañada	BC07	15.25	1	2	*Homoeorhynchia meridionalis*	brachiopod	ventral	08/06/2018	25/06/2018	38.4	3.69	0.893	0.092	0.74	2.37	0.14	14/08/2018	98.0	4.17	-2.52
MPZ 2019/449	CVU_carb_3695	Barranco de la Cañada	BC07	15.25	1	2	*Homoeorhynchia meridionalis*	brachiopod	dorsal	08/06/2018	25/06/2018	38.3	4.60	0.976	0.069	0.25	2.77	0.02	14/08/2018	99.1	3.97	-2.86
MPZ 2019/449	CVU_carb_3696	Barranco de la Cañada	BC07	15.25	1	2	*Homoeorhynchia meridionalis*	brachiopod	dorsal	08/06/2018	25/06/2018	38.6	4.26	0.990	0.058	0.20	3.16	0.15	14/08/2018	99.6	4.25	-2.58
MPZ 2019/449	CVU_carb_3697	Barranco de la Cañada	BC07	15.25	1	2	*Homoeorhynchia meridionalis*	brachiopod	dorsal	08/06/2018	25/06/2018	38.5	4.10	0.974	0.057	0.17	2.83	0.16	14/08/2018	98.6	4.20	-2.55
MPZ 2019/450	CVU_carb_3698	Barranco de la Cañada	BC07	15.25	1	3	*Homoeorhynchia meridionalis*	brachiopod	dorsal	08/06/2018	25/06/2018	38.2	6.04	1.128	0.214	0.71	3.42	0.16	14/08/2018	98.2	3.87	-2.54
MPZ 2019/450	CVU_carb_3699	Barranco de la Cañada	BC07	15.25	1	3	*Homoeorhynchia meridionalis*	brachiopod	dorsal	08/06/2018	25/06/2018	38.5	3.34	1.053	0.198	0.57	2.23	0.28	14/08/2018	98.9	4.38	-2.59
MPZ 2019/450	CVU_carb_3700	Barranco de la Cañada	BC07	15.25	1	3	*Homoeorhynchia meridionalis*	brachiopod	dorsal	08/06/2018	25/06/2018	38.4	5.96	1.134	0.099	0.33	3.46	0.20	14/08/2018	99.1	4.22	-2.66
MPZ 2019/442	CVU_carb_3435	Barranco de la Cañada	BC06	14.85	1	1	*Gryphaea* cf. *dumortieri*	bivalve		10/04/2018	04/06/2018	39.0	3.14	0.882	0.051	0.37	0.94	0.05	10/08/2018	100.4	5.34	-2.53
MPZ 2019/443	CVU_carb_3512	Barranco de la Cañada	BC06	14.85	2	1	*Homoeorhynchia meridionalis*	brachiopod	ventral	02/05/2018	04/06/2018	38.5	3.82	0.935	0.044	0.33	2.58	0.12	12/08/2018	99.6	4.56	-3.22
MPZ 2019/443	CVU_carb_3513	Barranco de la Cañada	BC06	14.85	2	1	*Homoeorhynchia meridionalis*	brachiopod	ventral	02/05/2018	04/06/2018	38.2	6.02	1.060	0.034	0.15	3.62	0.24	12/08/2018	98.8	4.55	-2.98
MPZ 2019/443	CVU_carb_3514	Barranco de la Cañada	BC06	14.85	2	1	*Homoeorhynchia meridionalis*	brachiopod	ventral	02/05/2018	04/06/2018	38.2	4.18	0.988	0.029	0.12	3.07	0.17	12/08/2018	99.0	4.61	-3.16
MPZ 2019/443	CVU_carb_3515	Barranco de la Cañada	BC06	14.85	2	1		bulk rock	bulk rock	02/05/2018	04/06/2018	32.2	26.51	0.452	0.691	16.60	1.21	1.05	12/08/2018	85.2	3.24	-4.35
MPZ 2019/444	CVU_carb_3516	Barranco de la Cañada	BC06	14.85	2	2	*Homoeorhynchia meridionalis*	brachiopod	dorsal	02/05/2018	04/06/2018	37.8	3.97	0.989	0.037	0.17	2.84	0.06	12/08/2018	99.2	4.46	-3.07
MPZ 2019/444	CVU_carb_3517	Barranco de la Cañada	BC06	14.85	2	2	*Homoeorhynchia meridionalis*	brachiopod	dorsal	02/05/2018	04/06/2018	38.3	3.90	1.025	0.023	0.10	3.10	0.22	12/08/2018	99.3	4.41	-3.23
MPZ 2019/444	CVU_carb_3518	Barranco de la Cañada	BC06	14.85	2	2	*Homoeorhynchia meridionalis*	brachiopod	dorsal	02/05/2018	04/06/2018	38.2	4.04	0.990	0.034	0.16	3.07	0.16	12/08/2018	100.0	4.34	-3.36
MPZ 2019/444	CVU_carb_3519	Barranco de la Cañada	BC06	14.85	2	2		calcite cement	calcite cement	02/05/2018	04/06/2018	38.0	10.33	0.291	0.827	8.36	0.56	0.00	12/08/2018	97.6	2.24	-9.70
MPZ 2019/444	CVU_carb_3520	Barranco de la Cañada	BC06	14.85	2	2		calcite cement	calcite cement	02/05/2018	04/06/2018	38.3	10.75	0.268	0.846	8.65	0.50	0.09	12/08/2018	99.6	2.28	-9.89
MPZ 2019/445	CVU_carb_3521	Barranco de la Cañada	BC06	14.85	2	3	*Homoeorhynchia meridionalis*	brachiopod	ventral	10/05/2018	04/06/2018	38.5	3.93	0.981	0.039	0.23	2.80	-0.05	12/08/2018	99.6	4.49	-2.73
MPZ 2019/445	CVU_carb_3522	Barranco de la Cañada	BC06	14.85	2	3	*Homoeorhynchia meridionalis*	brachiopod	ventral	10/05/2018	04/06/2018	38.6	4.60	1.024	0.030	0.16	2.95	0.03	12/08/2018	99.4	4.50	-2.82
MPZ 2019/445	CVU_carb_3523	Barranco de la Cañada	BC06	14.85	2	3	*Homoeorhynchia meridionalis*	brachiopod	ventral	10/05/2018	04/06/2018	38.4	4.52	1.018	0.027	0.17	2.88	0.02	12/08/2018	98.4	4.58	-2.96
MPZ 2019/445	CVU_carb_3524	Barranco de la Cañada	BC06	14.85	2	3	*Homoeorhynchia meridionalis*	brachiopod	dorsal	10/05/2018	04/06/2018	38.3	4.94	1.129	0.037	0.14	3.16	0.02	12/08/2018	99.5	4.55	-2.65
MPZ 2019/445	CVU_carb_3525	Barranco de la Cañada	BC06	14.85	2	3	*Homoeorhynchia meridionalis*	brachiopod	dorsal	10/05/2018	04/06/2018	38.1	5.14	1.024	0.054	0.18	3.09	0.14	12/08/2018	98.5	4.46	-2.79
MPZ 2019/445	CVU_carb_3526	Barranco de la Cañada	BC06	14.85	2	3	*Homoeorhynchia meridionalis*	brachiopod	dorsal	10/05/2018	04/06/2018	38.6	3.76	0.979	0.022	0.08	2.92	0.02	12/08/2018	99.9	4.49	-2.47
MPZ 2019/446	CVU_carb_3527	Barranco de la Cañada	BC06	14.85	2	4	*Homoeorhynchia meridionalis*	brachiopod	dorsal	10/05/2018	04/06/2018	38.1	7.89	1.134	0.076	0.42	4.17	0.26	12/08/2018	98.8	4.05	-2.54
MPZ 2019/446	CVU_carb_3528	Barranco de la Cañada	BC06	14.85	2	4	*Homoeorhynchia meridionalis*	brachiopod	dorsal	10/05/2018	04/06/2018	38.2	6.81	1.136	0.087	0.38	3.76	0.21	12/08/2018	99.3	4.62	-2.51
MPZ 2019/446	CVU_carb_3529	Barranco de la Cañada	BC06	14.85	2	4	*Homoeorhynchia meridionalis*	brachiopod	dorsal	10/05/2018	04/06/2018	38.3	6.18	1.079	0.097	0.45	3.63	-0.03	12/08/2018	98.6	4.22	-2.37
MPZ 2019/447	CVU_carb_3530	Barranco de la Cañada	BC06	14.85	2	5	*Homoeorhynchia meridionalis*	brachiopod		10/05/2018	04/06/2018	38.2	6.74	1.041	0.079	0.58	3.93	0.24	12/08/2018	98.9	4.48	-2.27
MPZ 2019/447	CVU_carb_3531	Barranco de la Cañada	BC06	14.85	2	5	*Homoeorhynchia meridionalis*	brachiopod		10/05/2018	04/06/2018	38.3	4.91	1.092	0.050	0.21	3.28	0.17	12/08/2018	99.1	4.69	-2.69
MPZ 2019/447	CVU_carb_3532	Barranco de la Cañada	BC06	14.85	2	5	*Homoeorhynchia meridionalis*	brachiopod		10/05/2018	04/06/2018	38.7	4.68	0.949	0.073	0.40	3.19	0.23	12/08/2018	98.7	5.02	-2.26
MPZ 2019/442	CVU_carb_3870	Barranco de la Cañada	BC06	14.85	1	1	*Gryphaea* cf. *dumortieri*	bivalve		29/06/2018	06/07/2018	39.2	2.54	0.749	0.056	0.41	0.88	0.11	16/08/2018	100.6	5.02	-2.14
MPZ 2019/442	CVU_carb_3871	Barranco de la Cañada	BC06	14.85	1	1	*Gryphaea* cf. *dumortieri*	bivalve		29/06/2018	06/07/2018	38.8	3.40	0.920	0.057	0.43	1.78	0.00	16/08/2018	100.5	4.99	-2.80
MPZ 2019/442	CVU_carb_3872	Barranco de la Cañada	BC06	14.85	1	1	*Gryphaea* cf. *dumortieri*	bivalve		29/06/2018	06/07/2018	39.0	3.17	0.892	0.065	0.47	1.67	0.00	16/08/2018	99.5	5.22	-2.68
MPZ 2019/440	CVU_carb_3429	Barranco de la Cañada	BC05	14.25	1	1	*Homoeorhynchia batalleri*	brachiopod		10/04/2018	01/06/2018	38.2	4.85	1.117	0.060	0.18	2.49	0.12	10/08/2018	97.7	4.57	-1.70
MPZ 2019/440	CVU_carb_3484	Barranco de la Cañada	BC05	14.25	1	1	*Homoeorhynchia batalleri*	brachiopod		29/04/2018	04/06/2018	38.4	4.53	1.063	0.088	0.27	2.27	0.12	10/08/2018	99.1	4.70	-2.19
MPZ 2019/440	CVU_carb_3485	Barranco de la Cañada	BC05	14.25	1	1	*Homoeorhynchia batalleri*	brachiopod		29/04/2018	04/06/2018	38.8	4.39	1.008	0.098	0.28	2.44	-0.02	10/08/2018	99.5	4.59	-2.10
MPZ 2019/440	CVU_carb_3486	Barranco de la Cañada	BC05	14.25	1	1	*Homoeorhynchia batalleri*	brachiopod		29/04/2018	04/06/2018	38.8	5.05	1.075	0.093	0.37	2.57	0.13	10/08/2018	99.7	4.59	-2.18
MPZ 2019/441	CVU_carb_3487	Barranco de la Cañada	BC05	14.25	1	2	*Homoeorhynchia batalleri*	brachiopod		29/04/2018	04/06/2018	38.2	6.46	0.967	0.212	1.39	2.40	0.04	10/08/2018	99.8	4.64	-2.11
MPZ 2019/441	CVU_carb_3488	Barranco de la Cañada	BC05	14.25	1	2	*Homoeorhynchia batalleri*	brachiopod		29/04/2018	04/06/2018	37.6	8.23	0.883	0.333	2.74	2.41	0.35	10/08/2018	98.8	4.67	-2.12
MPZ 2019/441	CVU_carb_3489	Barranco de la Cañada	BC05	14.25	1	2	*Homoeorhynchia batalleri*	brachiopod		29/04/2018	04/06/2018	38.2	6.54	1.042	0.171	0.98	3.03	0.16	10/08/2018	99.2	4.45	-2.52
MPZ 2019/441	CVU_carb_3490	Barranco de la Cañada	BC05	14.25	1	2		bulk rock		29/04/2018	04/06/2018	31.1	28.80	0.427	0.574	18.73	1.10	0.88	10/08/2018	80.7	3.50	-4.10
MPZ 2019/435	CVU_carb_3575	Barranco de la Cañada	BC04	13.75	2	1	*Gibbirhynchia muirwoodae*	brachiopod	ventral	28/05/2018	08/06/2018	37.9	4.76	1.128	0.039	0.08	3.06	0.20	12/08/2018	99.0	3.90	-1.60
MPZ 2019/435	CVU_carb_3576	Barranco de la Cañada	BC04	13.75	2	1	*Gibbirhynchia muirwoodae*	brachiopod	ventral	28/05/2018	08/06/2018	38.4	5.51	1.085	0.050	0.10	3.17	0.13	12/08/2018	99.2	3.87	-1.77
MPZ 2019/435	CVU_carb_3577	Barranco de la Cañada	BC04	13.75	2	1	*Gibbirhynchia muirwoodae*	brachiopod	ventral	28/05/2018	08/06/2018	38.1	4.83	1.116	0.050	0.11	2.72	0.21	12/08/2018	99.2	3.92	-1.45
MPZ 2019/435	CVU_carb_3578	Barranco de la Cañada	BC04	13.75	2	1	*Gibbirhynchia muirwoodae*	brachiopod	dorsal	28/05/2018	08/06/2018	38.4	5.58	1.156	0.064	0.21	3.18	0.15	12/08/2018	99.5	3.69	-1.82
MPZ 2019/435	CVU_carb_3579	Barranco de la Cañada	BC04	13.75	2	1	*Gibbirhynchia muirwoodae*	brachiopod	dorsal	28/05/2018	08/06/2018	38.3	5.24	1.134	0.071	0.14	3.06	0.06	12/08/2018	99.2	3.70	-1.84
MPZ 2019/435	CVU_carb_3580	Barranco de la Cañada	BC04	13.75	2	1	*Gibbirhynchia muirwoodae*	brachiopod	dorsal	28/05/2018	08/06/2018	38.5	4.35	1.068	0.055	0.18	2.61	0.18	12/08/2018	97.7	3.61	-1.91
MPZ 2019/435	CVU_carb_3581	Barranco de la Cañada	BC04	13.75	2	1		bulk rock	bulk rock	28/05/2018	08/06/2018	33.2	24.57	0.432	0.496	12.53	0.92	0.72	12/08/2018	87.9	3.64	-4.14
MPZ 2019/437	CVU_carb_3622	Barranco de la Cañada	BC04	13.75	3	1	*Homoeorhynchia* ? aff. *batalleri*	brachiopod	ventral	04/06/2018	08/06/2018	38.0	8.77	1.241	0.022	0.31	4.00	0.24	13/08/2018	98.2	3.95	-1.91
MPZ 2019/437	CVU_carb_3623	Barranco de la Cañada	BC04	13.75	3	1	*Homoeorhynchia* ? aff. *batalleri*	brachiopod	dorsal	04/06/2018	08/06/2018	38.3	6.97	0.954	0.057	0.35	3.57	0.03	13/08/2018	100.1	3.59	-2.60
MPZ 2019/437	CVU_carb_3624	Barranco de la Cañada	BC04	13.75	3	1	*Homoeorhynchia* ? aff. *batalleri*	brachiopod	dorsal	04/06/2018	08/06/2018	38.1	7.84	1.045	0.047	0.30	4.22	0.05	13/08/2018	99.7	3.60	-2.59
MPZ 2019/438	CVU_carb_3625	Barranco de la Cañada	BC04	13.75	3	2	*Homoeorhynchia* ? aff. *batalleri*	brachiopod	ventral	04/06/2018	08/06/2018	38.0	5.56	1.124	0.048	0.44	3.01	0.23	13/08/2018	98.9	4.09	-1.82
MPZ 2019/438	CVU_carb_3626	Barranco de la Cañada	BC04	13.75	3	2	*Homoeorhynchia* ? aff. *batalleri*	brachiopod	ventral	04/06/2018	08/06/2018	38.1	4.80	1.111	0.029	0.14	2.87	0.32	13/08/2018	100.1	4.20	-1.69
MPZ 2019/438	CVU_carb_3627	Barranco de la Cañada	BC04	13.75	3	2	*Homoeorhynchia* ? aff. *batalleri*	brachiopod	ventral	04/06/2018	08/06/2018	38.3	4.18	1.083	0.026	0.13	2.57	0.23	13/08/2018	98.5	4.13	-1.46
MPZ 2019/438	CVU_carb_3628	Barranco de la Cañada	BC04	13.75	3	2	*Homoeorhynchia* ? aff. *batalleri*	brachiopod	dorsal	04/06/2018	08/06/2018	38.2	4.22	1.034	0.038	0.12	2.65	0.26	13/08/2018	98.9	4.10	-1.34
MPZ 2019/438	CVU_carb_3629	Barranco de la Cañada	BC04	13.75	3	2	*Homoeorhynchia* ? aff. *batalleri*	brachiopod	dorsal	04/06/2018	08/06/2018	38.3	3.90	0.989	0.053	0.25	2.29	0.13	13/08/2018	98.5	4.06	-1.58
MPZ 2019/438	CVU_carb_3630	Barranco de la Cañada	BC04	13.75	3	2	*Homoeorhynchia* ? aff. *batalleri*	brachiopod	dorsal	04/06/2018	08/06/2018	38.4	3.89	0.997	0.053	0.23	2.50	0.21	13/08/2018	99.2	4.14	-1.67
MPZ 2019/438	CVU_carb_3631	Barranco de la Cañada	BC04	13.75	3	2		calcite cement	geopetal cement	04/06/2018	08/06/2018	38.0	10.80	0.398	0.814	9.15	0.40	0.24	13/08/2018	99.4	2.13	-10.44
MPZ 2019/439	CVU_carb_3632	Barranco de la Cañada	BC04	13.75	3	3	*Homoeorhynchia* ? aff. *batalleri*	brachiopod		04/06/2018	08/06/2018	38.3	5.08	1.030	0.060	0.16	2.88	0.25	13/08/2018	99.7	4.15	-1.59
MPZ 2019/439	CVU_carb_3633	Barranco de la Cañada	BC04	13.75	3	3	*Homoeorhynchia* ? aff. *batalleri*	brachiopod		04/06/2018	08/06/2018	38.3	4.77	1.023	0.044	0.07	2.79	0.11	13/08/2018	98.7	4.27	-1.38
MPZ 2019/439	CVU_carb_3634	Barranco de la Cañada	BC04	13.75	3	3	*Homoeorhynchia* ? aff. *batalleri*	brachiopod		04/06/2018	08/06/2018	38.2	5.35	1.067	0.049	0.14	2.88	0.20	13/08/2018	99.7	4.19	-1.35
MPZ 2019/434	CVU_carb_3703	Barranco de la Cañada	BC04	13.75	1	1	*Homoeorhynchia meridionalis*	brachiopod		08/06/2018	25/06/2018	38.5	5.00	1.137	0.043	0.07	2.60	0.02	14/08/2018	98.6	3.74	-1.88
MPZ 2019/434	CVU_carb_3704	Barranco de la Cañada	BC04	13.75	1	1	*Homoeorhynchia meridionalis*	brachiopod		08/06/2018	25/06/2018	38.3	4.38	1.028	0.056	0.12	2.73	0.13	14/08/2018	99.6	3.94	-1.71
MPZ 2019/434	CVU_carb_3705	Barranco de la Cañada	BC04	13.75	1	1	*Homoeorhynchia meridionalis*	brachiopod		08/06/2018	25/06/2018	38.1	4.67	1.056	0.057	0.11	2.90	0.17	14/08/2018	100.1	3.76	-1.86
MPZ 2019/432	CVU_carb_3641	Barranco de la Cañada	BC03	13.20	2	1	*Homoeorhynchia batalleri*	brachiopod	ventral	04/06/2018	08/06/2018	38.3	7.00	0.953	0.128	0.54	3.86	0.37	13/08/2018	100.2	4.51	-2.65
MPZ 2019/432	CVU_carb_3642	Barranco de la Cañada	BC03	13.20	2	1	*Homoeorhynchia batalleri*	brachiopod	ventral	04/06/2018	08/06/2018	38.5	5.36	0.882	0.086	0.38	3.10	0.09	13/08/2018	99.1	4.26	-2.38
MPZ 2019/432	CVU_carb_3643	Barranco de la Cañada	BC03	13.20	2	1	*Homoeorhynchia batalleri*	brachiopod	ventral	04/06/2018	08/06/2018	38.0	5.59	0.902	0.075	0.51	3.53	0.27	13/08/2018	99.3	4.06	-2.48
MPZ 2019/432	CVU_carb_3644	Barranco de la Cañada	BC03	13.20	2	1	*Homoeorhynchia batalleri*	brachiopod	dorsal	04/06/2018	08/06/2018	37.9	9.03	1.035	0.065	0.41	5.51	0.33	13/08/2018	98.4	4.37	-2.49
MPZ 2019/432	CVU_carb_3645	Barranco de la Cañada	BC03	13.20	2	1	*Homoeorhynchia batalleri*	brachiopod	ventral	04/06/2018	08/06/2018	38.8	5.19	0.890	0.072	0.29	3.03	-0.02	13/08/2018	99.5	4.21	-2.66
MPZ 2019/432	CVU_carb_3646	Barranco de la Cañada	BC03	13.20	2	1	*Homoeorhynchia batalleri*	brachiopod	dorsal	04/06/2018	08/06/2018	38.6	5.24	0.953	0.048	0.20	3.38	0.26	13/08/2018	99.0	3.85	-2.45
MPZ 2019/432	CVU_carb_3647	Barranco de la Cañada	BC03	13.20	2	1		calcite cement	cement	04/06/2018	08/06/2018	38.1	11.53	0.256	0.806	10.52	0.21	0.18	13/08/2018	100.0	2.05	-9.94
MPZ 2019/431	CVU_carb_3676	Barranco de la Cañada	BC03	13.20	1	1	*Choffatirhynchia* aff. *paucicostata*	brachiopod	ventral	07/06/2018	25/06/2018	37.6	7.37	1.015	0.066	0.23	3.42	-0.13	14/08/2018	98.5	3.58	-1.96
MPZ 2019/431	CVU_carb_3677	Barranco de la Cañada	BC03	13.20	1	1	*Choffatirhynchia* aff. *paucicostata*	brachiopod	ventral	07/06/2018	25/06/2018	38.8	7.22	1.055	0.054	0.19	3.57	0.17	14/08/2018	99.3	3.73	-1.96
MPZ 2019/431	CVU_carb_3678	Barranco de la Cañada	BC03	13.20	1	1	*Choffatirhynchia* aff. *paucicostata*	brachiopod	ventral	07/06/2018	25/06/2018	37.9	7.62	0.999	0.094	0.43	3.50	0.16	14/08/2018	98.7	3.78	-1.88
MPZ 2019/431	CVU_carb_3679	Barranco de la Cañada	BC03	13.20	1	1	*Choffatirhynchia* aff. *paucicostata*	brachiopod	dorsal	07/06/2018	25/06/2018	38.0	7.36	1.054	0.060	0.25	3.43	0.03	14/08/2018	99.3	3.69	-2.07
MPZ 2019/431	CVU_carb_3680	Barranco de la Cañada	BC03	13.20	1	1	*Choffatirhynchia* aff. *paucicostata*	brachiopod	dorsal	07/06/2018	25/06/2018	38.0	6.82	0.934	0.078	0.33	3.51	0.12	14/08/2018	99.8	3.28	-2.10
MPZ 2019/433	CVU_carb_3834	Barranco de la Cañada	BC03	13.20	3	1	*Gryphaea cf. dumortieri*	bivalve		29/06/2018	06/07/2018	39.0	3.02	0.689	0.023	0.19	1.19	0.54	16/08/2018	100.0	4.42	-1.56
MPZ 2019/433	CVU_carb_3835	Barranco de la Cañada	BC03	13.20	3	1	*Gryphaea cf. dumortieri*	bivalve		29/06/2018	06/07/2018	38.8	3.13	0.669	0.027	0.28	1.27	0.47	16/08/2018	100.5	4.75	-1.58
MPZ 2019/433	CVU_carb_3836	Barranco de la Cañada	BC03	13.20	3	1	*Gryphaea cf. dumortieri*	bivalve		29/06/2018	06/07/2018	38.9	3.50	0.784	0.017	0.24	3.69	0.37	16/08/2018	99.6	4.81	-1.51
MPZ 2019/433	CVU_carb_3837	Barranco de la Cañada	BC03	13.20	3	1		bulk rock	bulk rock	29/06/2018	06/07/2018	35.9	24.03	0.373	0.340	7.86	0.93	0.50	16/08/2018	91.5	4.32	-2.40
MPZ 2019/424	CVU_carb_3440	Barranco de la Cañada	BC02	12.60	2	1	*Choffatirhynchia vasconcellosi*	brachiopod	ventral	28/04/2018	04/06/2018	38.5	6.67	0.981	0.050	0.40	3.52	0.05	10/08/2018	98.6	3.63	-1.70
MPZ 2019/424	CVU_carb_3441	Barranco de la Cañada	BC02	12.60	2	1	*Choffatirhynchia vasconcellosi*	brachiopod	ventral	28/04/2018	04/06/2018	38.3	6.24	0.998	0.042	0.32	3.42	0.10	10/08/2018	98.6	3.65	-1.52
MPZ 2019/424	CVU_carb_3442	Barranco de la Cañada	BC02	12.60	2	1	*Choffatirhynchia vasconcellosi*	brachiopod	ventral	28/04/2018	04/06/2018	38.9	6.10	0.986	0.041	0.32	3.30	0.05	10/08/2018	98.7	3.58	-1.42
MPZ 2019/424	CVU_carb_3443	Barranco de la Cañada	BC02	12.60	2	1	*Choffatirhynchia vasconcellosi*	brachiopod	dorsal	28/04/2018	04/06/2018	38.6	6.28	1.026	0.036	0.20	3.47	0.14	10/08/2018	98.7	4.19	-1.62
MPZ 2019/424	CVU_carb_3444	Barranco de la Cañada	BC02	12.60	2	1	*Choffatirhynchia vasconcellosi*	brachiopod	dorsal	28/04/2018	04/06/2018	38.3	6.74	1.034	0.046	0.31	3.25	0.06	10/08/2018	99.3	3.92	-1.78
MPZ 2019/424	CVU_carb_3445	Barranco de la Cañada	BC02	12.60	2	1	*Choffatirhynchia vasconcellosi*	brachiopod	dorsal	28/04/2018	04/06/2018	38.8	6.05	1.023	0.042	0.26	3.14	0.08	10/08/2018	99.2	4.34	-1.95
MPZ 2019/424	CVU_carb_3446	Barranco de la Cañada	BC02	12.60	2	1		bulk rock	bulk rock	28/04/2018	04/06/2018	29.7	33.87	0.442	0.475	12.97	0.99	0.91	10/08/2018	78.2	3.54	-3.56

MPZ 2019/425	CVU_carb_3447	Barranco de la Cañada	BC02	12.60	2	2	*Choffatirhynchia vasconcellosi*	brachiopod	ventral	28/04/2018	04/06/2018	38.4	5.69	0.973	0.085	0.38	2.73	0.11	10/08/2018	99.1	4.15	-1.91
MPZ 2019/425	CVU_carb_3448	Barranco de la Cañada	BC02	12.60	2	2	*Choffatirhynchia vasconcellosi*	brachiopod	ventral	28/04/2018	04/06/2018	38.4	4.48	0.890	0.068	0.29	2.52	0.04	10/08/2018	100.0	3.74	-1.98
MPZ 2019/425	CVU_carb_3449	Barranco de la Cañada	BC02	12.60	2	2	*Choffatirhynchia vasconcellosi*	brachiopod	ventral	28/04/2018	04/06/2018	38.6	4.27	0.828	0.094	0.47	2.01	0.10	10/08/2018	100.5	3.67	-2.10
MPZ 2019/425	CVU_carb_3450	Barranco de la Cañada	BC02	12.60	2	2	*Choffatirhynchia vasconcellosi*	brachiopod	dorsal	28/04/2018	04/06/2018	38.2	4.95	0.925	0.069	0.28	2.67	0.03	10/08/2018	98.7	4.10	-1.97
MPZ 2019/425	CVU_carb_3451	Barranco de la Cañada	BC02	12.60	2	2	*Choffatirhynchia vasconcellosi*	brachiopod	dorsal	28/04/2018	04/06/2018	39.1	3.79	0.792	0.094	0.47	1.98	0.07	10/08/2018	99.1	3.76	-1.88
MPZ 2019/425	CVU_carb_3452	Barranco de la Cañada	BC02	12.60	2	2	*Choffatirhynchia vasconcellosi*	brachiopod	dorsal	28/04/2018	04/06/2018	38.3	4.73	0.839	0.089	0.44	2.14	0.14	10/08/2018	99.7	3.85	-1.98
MPZ 2019/426	CVU_carb_3453	Barranco de la Cañada	BC02	12.60	2	3	*Choffatirhynchia vasconcellosi*	brachiopod	ventral	28/04/2018	04/06/2018	37.9	7.76	1.031	0.094	0.60	3.16	-0.10	10/08/2018	98.5	3.93	-1.47
MPZ 2019/426	CVU_carb_3454	Barranco de la Cañada	BC02	12.60	2	3	*Choffatirhynchia vasconcellosi*	brachiopod	ventral	28/04/2018	04/06/2018	38.2	6.86	1.030	0.065	0.33	3.09	0.05	10/08/2018	98.3	4.00	-1.55
MPZ 2019/426	CVU_carb_3455	Barranco de la Cañada	BC02	12.60	2	3	*Choffatirhynchia vasconcellosi*	brachiopod	ventral	28/04/2018	04/06/2018	38.2	8.44	0.984	0.108	0.84	3.53	0.01	10/08/2018	98.8	3.35	-2.09
MPZ 2019/426	CVU_carb_3456	Barranco de la Cañada	BC02	12.60	2	3	*Choffatirhynchia vasconcellosi*	brachiopod	dorsal	29/04/2018	04/06/2018	38.0	9.32	1.106	0.085	0.53	3.99	-0.01	10/08/2018	98.4	4.31	-1.60
MPZ 2019/426	CVU_carb_3457	Barranco de la Cañada	BC02	12.60	2	3	*Choffatirhynchia vasconcellosi*	brachiopod	dorsal	29/04/2018	04/06/2018	37.8	13.40	1.428	0.044	0.19	5.77	0.03	10/08/2018	98.1	4.22	-1.93
MPZ 2019/426	CVU_carb_3458	Barranco de la Cañada	BC02	12.60	2	3	*Choffatirhynchia vasconcellosi*	brachiopod	dorsal	29/04/2018	04/06/2018	38.5	11.63	1.098	0.117	0.69	4.13	-0.02	10/08/2018	99.8	4.09	-1.89
MPZ 2019/427	CVU_carb_3459	Barranco de la Cañada	BC02	12.60	2	4	*Choffatirhynchia vasconcellosi*	brachiopod	dorsal	29/04/2018	04/06/2018	38.0	6.84	0.926	0.076	0.38	3.86	0.10	10/08/2018	99.7	4.20	-2.07
MPZ 2019/427	CVU_carb_3460	Barranco de la Cañada	BC02	12.60	2	4	*Choffatirhynchia vasconcellosi*	brachiopod	dorsal	29/04/2018	04/06/2018	38.0	7.43	0.980	0.072	0.37	3.89	-0.02	10/08/2018	99.2	4.24	-1.71
MPZ 2019/427	CVU_carb_3461	Barranco de la Cañada	BC02	12.60	2	4	*Choffatirhynchia vasconcellosi*	brachiopod	dorsal	29/04/2018	04/06/2018	38.2	6.81	0.912	0.080	0.41	3.56	0.09	10/08/2018	99.1	4.13	-1.69
MPZ 2019/427	CVU_carb_3462	Barranco de la Cañada	BC02	12.60	2	4	*Choffatirhynchia vasconcellosi*	brachiopod	ventral	29/04/2018	04/06/2018	38.2	7.02	0.986	0.072	0.32	3.56	-0.03	10/08/2018	99.4	4.13	-2.10
MPZ 2019/427	CVU_carb_3463	Barranco de la Cañada	BC02	12.60	2	4	*Choffatirhynchia vasconcellosi*	brachiopod	ventral	29/04/2018	04/06/2018	38.0	8.46	1.257	0.035	0.14	3.80	0.04	10/08/2018	98.5	4.13	-1.38
MPZ 2019/427	CVU_carb_3464	Barranco de la Cañada	BC02	12.60	2	4	*Choffatirhynchia vasconcellosi*	brachiopod	ventral	29/04/2018	04/06/2018	38.2	6.79	0.881	0.083	0.39	3.30	0.06	10/08/2018	100.3	4.14	-1.57
MPZ 2019/428	CVU_carb_3465	Barranco de la Cañada	BC02	12.60	2	5	*Choffatirhynchia vasconcellosi*	brachiopod	ventral	29/04/2018	04/06/2018	38.3	5.91	0.944	0.105	1.01	2.98	0.15	10/08/2018	100.5	4.16	-1.78
MPZ 2019/428	CVU_carb_3466	Barranco de la Cañada	BC02	12.60	2	5	*Choffatirhynchia vasconcellosi*	brachiopod	ventral	29/04/2018	04/06/2018	38.2	6.17	0.944	0.089	0.98	3.14	0.10	10/08/2018	100.0	4.18	-1.71
MPZ 2019/428	CVU_carb_3467	Barranco de la Cañada	BC02	12.60	2	5	*Choffatirhynchia vasconcellosi*	brachiopod	ventral	29/04/2018	04/06/2018	38.1	6.37	0.989	0.063	0.55	3.12	-0.02	10/08/2018	99.1	4.22	-1.35
MPZ 2019/428	CVU_carb_3468	Barranco de la Cañada	BC02	12.60	2	5	*Choffatirhynchia vasconcellosi*	brachiopod	dorsal	29/04/2018	04/06/2018	38.5	6.03	0.935	0.087	0.71	3.13	0.22	10/08/2018	98.2	3.87	-1.87
MPZ 2019/428	CVU_carb_3469	Barranco de la Cañada	BC02	12.60	2	5	*Choffatirhynchia vasconcellosi*	brachiopod	dorsal	29/04/2018	04/06/2018	38.7	6.92	0.986	0.100	0.83	3.15	0.16	10/08/2018	99.6	3.69	-1.82
MPZ 2019/428	CVU_carb_3470	Barranco de la Cañada	BC02	12.60	2	5	*Choffatirhynchia vasconcellosi*	brachiopod	dorsal	29/04/2018	04/06/2018	38.3	6.55	0.930	0.085	0.65	2.65	0.18	10/08/2018	99.2	3.37	-1.93
MPZ 2019/429	CVU_carb_3560	Barranco de la Cañada	BC02	12.60	3	1	*Choffatirhynchia* aff. *paucicostata*	brachiopod	ventral	28/05/2018	08/06/2018	38.7	6.96	0.956	0.060	0.29	3.77	0.22	12/08/2018	99.1	3.87	-2.19
MPZ 2019/429	CVU_carb_3561	Barranco de la Cañada	BC02	12.60	3	1	*Choffatirhynchia* aff. *paucicostata*	brachiopod	ventral	28/05/2018	08/06/2018	38.3	6.89	0.959	0.060	0.30	3.96	0.19	12/08/2018	99.3	3.79	-2.01
MPZ 2019/429	CVU_carb_3562	Barranco de la Cañada	BC02	12.60	3	1	*Choffatirhynchia* aff. *paucicostata*	brachiopod	ventral	28/05/2018	08/06/2018	38.5	5.96	0.822	0.074	0.44	3.37	0.01	12/08/2018	100.6	3.90	-2.26
MPZ 2019/429	CVU_carb_3563	Barranco de la Cañada	BC02	12.60	3	1	*Choffatirhynchia* aff. *paucicostata*	brachiopod	dorsal	28/05/2018	08/06/2018	38.1	7.91	1.024	0.075	0.54	4.13	0.02	12/08/2018	99.1	4.11	-2.12
MPZ 2019/429	CVU_carb_3564	Barranco de la Cañada	BC02	12.60	3	1	*Choffatirhynchia* aff. *paucicostata*	brachiopod	dorsal	28/05/2018	08/06/2018	38.4	7.66	0.996	0.069	0.33	4.32	0.21	12/08/2018	99.8	4.07	-2.05
MPZ 2019/429	CVU_carb_3565	Barranco de la Cañada	BC02	12.60	3	1		calcite cement	geopetal cement	28/05/2018	08/06/2018	37.8	11.02	0.215	0.774	10.29	0.83	0.02	12/08/2018	100.2	1.97	-9.27
MPZ 2019/430	CVU_carb_3566	Barranco de la Cañada	BC02	12.60	3	2	*Choffatirhynchia* aff. *paucicostata*	brachiopod	ventral	28/05/2018	08/06/2018	37.9	7.15	1.074	0.046	0.24	4.04	0.13	12/08/2018	99.0	4.24	-1.91
MPZ 2019/430	CVU_carb_3567	Barranco de la Cañada	BC02	12.60	3	2	*Choffatirhynchia* aff. *paucicostata*	brachiopod	dorsal	28/05/2018	08/06/2018	38.0	6.53	1.018	0.063	0.33	4.15	0.10	12/08/2018	97.9	4.09	-2.30
MPZ 2019/430	CVU_carb_3568	Barranco de la Cañada	BC02	12.60	3	2	*Choffatirhynchia* aff. *paucicostata*	brachiopod	dorsal	28/05/2018	08/06/2018	38.1	6.16	0.979	0.054	0.25	3.77	0.13	12/08/2018	99.4	3.99	-2.08
MPZ 2019/430	CVU_carb_3569	Barranco de la Cañada	BC02	12.60	3	2	*Choffatirhynchia* aff. *paucicostata*	brachiopod	dorsal	28/05/2018	08/06/2018	38.4	5.94	0.922	0.059	0.26	3.39	0.13	12/08/2018	99.3	3.99	-1.86
MPZ 2019/430	CVU_carb_3570	Barranco de la Cañada	BC02	12.60	3	2		calcite cement	sparry calcite cement	28/05/2018	08/06/2018	37.9	10.99	0.245	0.745	9.29	0.73	-0.09	12/08/2018	99.9	2.02	-9.05
MPZ 2019/421	CVU_carb_3582	Barranco de la Cañada	BC02	12.60	1	1	*Choffatirhynchia vasconcellosi*	brachiopod	ventral	03/06/2018	08/06/2018	38.6	6.58	1.011	0.048	0.20	4.25	0.08	12/08/2018	99.6	4.06	-1.59
MPZ 2019/421	CVU_carb_3583	Barranco de la Cañada	BC02	12.60	1	1	*Choffatirhynchia vasconcellosi*	brachiopod	ventral	03/06/2018	08/06/2018	38.6	6.33	0.911	0.064	0.28	3.50	0.18	12/08/2018	99.8	3.78	-1.66
MPZ 2019/421	CVU_carb_3584	Barranco de la Cañada	BC02	12.60	1	1	*Choffatirhynchia vasconcellosi*	brachiopod	ventral	03/06/2018	08/06/2018	38.1	7.86	0.989	0.046	0.22	3.99	0.10	12/08/2018	99.4	3.97	-1.66
MPZ 2019/421	CVU_carb_3585	Barranco de la Cañada	BC02	12.60	1	1	*Choffatirhynchia vasconcellosi*	brachiopod	dorsal	03/06/2018	08/06/2018	38.5	7.66	1.035	0.058	0.34	4.58	0.10	12/08/2018	99.3	4.14	-1.44
MPZ 2019/421	CVU_carb_3586	Barranco de la Cañada	BC02	12.60	1	1	*Choffatirhynchia vasconcellosi*	brachiopod	dorsal	03/06/2018	08/06/2018	38.1	7.41	1.009	0.052	0.27	4.10	0.06	12/08/2018	99.9	3.83	-1.65
MPZ 2019/421	CVU_carb_3587	Barranco de la Cañada	BC02	12.60	1	1	*Choffatirhynchia vasconcellosi*	brachiopod	dorsal	03/06/2018	08/06/2018	38.1	7.47	0.984	0.062	0.32	3.90	0.05	12/08/2018	100.6	3.25	-1.83
MPZ 2019/422	CVU_carb_3588	Barranco de la Cañada	BC02	12.60	1	2	*Choffatirhynchia vasconcellosi*	brachiopod	ventral	03/06/2018	08/06/2018	38.3	7.73	1.020	0.064	0.29	4.50	0.10	13/08/2018	97.9	4.00	-1.96
MPZ 2019/422	CVU_carb_3589	Barranco de la Cañada	BC02	12.60	1	2	*Choffatirhynchia vasconcellosi*	brachiopod	ventral	03/06/2018	08/06/2018	38.2	6.75	0.967	0.071	0.28	4.17	0.11	13/08/2018	97.2	3.76	-2.01
MPZ 2019/422	CVU_carb_3590	Barranco de la Cañada	BC02	12.60	1	2	*Choffatirhynchia vasconcellosi*	brachiopod	ventral	03/06/2018	08/06/2018	38.2	7.54	0.929	0.097	0.63	4.05	0.10	13/08/2018	97.3	3.40	-2.42
MPZ 2019/422	CVU_carb_3591	Barranco de la Cañada	BC02	12.60	1	2	*Choffatirhynchia vasconcellosi*	brachiopod	dorsal	03/06/2018	08/06/2018	38.1	8.62	1.028	0.071	0.48	4.63	0.04	13/08/2018	98.1	3.86	-2.04
MPZ 2019/422	CVU_carb_3592	Barranco de la Cañada	BC02	12.60	1	2	*Choffatirhynchia vasconcellosi*	brachiopod	dorsal	03/06/2018	08/06/2018	38.0	7.92	0.996	0.068	0.43	4.15	0.21	13/08/2018	98.1	3.67	-2.23
MPZ 2019/422	CVU_carb_3593	Barranco de la Cañada	BC02	12.60	1	2	*Choffatirhynchia vasconcellosi*	brachiopod	dorsal	03/06/2018	08/06/2018	38.3	6.50	0.931	0.075	0.38	3.89	0.15	13/08/2018	98.5	3.64	-2.25
MPZ 2019/423	CVU_carb_3594	Barranco de la Cañada	BC02	12.60	1	3	*Choffatirhynchia vasconcellosi*	brachiopod	ventral	03/06/2018	08/06/2018	38.1	7.66	1.008	0.055	0.22	4.40	0.07	13/08/2018	97.8	5.08	-2.72
MPZ 2019/423	CVU_carb_3595	Barranco de la Cañada	BC02	12.60	1	3	*Choffatirhynchia vasconcellosi*	brachiopod	ventral	03/06/2018	08/06/2018	38.0	7.67	0.976	0.052	0.19	4.20	0.15	13/08/2018	98.8	4.81	-2.62
MPZ 2019/423	CVU_carb_3596	Barranco de la Cañada	BC02	12.60	1	3	*Choffatirhynchia vasconcellosi*	brachiopod	ventral	03/06/2018	08/06/2018	38.7	6.66	0.953	0.052	0.24	3.80	0.00	13/08/2018	97.8	4.77	-2.91
MPZ 2019/417	CVU_carb_3437	Barranco de la Cañada	BC01b	12.35	2	1		calcite cement	sparitic calcite	10/04/2018	04/06/2018	38.2	11.26	0.243	0.737	9.70	0.25	0.05	10/08/2018	100.1	1.96	-9.33
MPZ 2019/416	CVU_carb_3438	Barranco de la Cañada	BC01b	12.35	1	1		bulk rock	bulk rock	10/04/2018	04/06/2018	33.2	23.77	0.410	0.427	8.61	0.97	0.40	10/08/2018	86.8	3.85	-3.49
MPZ 2019/417	CVU_carb_3648	Barranco de la Cañada	BC01b	12.35	2	1	*Choffatirhynchia vasconcellosi*	brachiopod	ventral	05/06/2018	08/06/2018	38.1	6.52	1.133	0.052	0.19	3.20	0.16	13/08/2018	98.6	4.66	-2.92
MPZ 2019/417	CVU_carb_3649	Barranco de la Cañada	BC01b	12.35	2	1	*Choffatirhynchia vasconcellosi*	brachiopod	ventral	05/06/2018	08/06/2018	38.3	6.12	0.957	0.082	0.34	3.03	0.19	13/08/2018	99.1	4.69	-2.81
MPZ 2019/417	CVU_carb_3650	Barranco de la Cañada	BC01b	12.35	2	1	*Choffatirhynchia vasconcellosi*	brachiopod	ventral	05/06/2018	08/06/2018	38.8	4.78	0.839	0.078	0.36	3.02	0.11	13/08/2018	100.5	4.55	-2.68
MPZ 2019/417	CVU_carb_3651	Barranco de la Cañada	BC01b	12.35	2	1	*Choffatirhynchia vasconcellosi*	brachiopod	dorsal	05/06/2018	08/06/2018	38.5	4.87	0.885	0.088	0.40	2.76	0.21	13/08/2018	100.0	4.41	-3.00
MPZ 2019/417	CVU_carb_3652	Barranco de la Cañada	BC01b	12.35	2	1	*Choffatirhynchia vasconcellosi*	brachiopod	dorsal	05/06/2018	08/06/2018	38.8	4.59	0.871	0.089	0.40	2.47	0.04	13/08/2018	100.6	4.38	-2.68
MPZ 2019/417	CVU_carb_3653	Barranco de la Cañada	BC01b	12.35	2	1	*Choffatirhynchia vasconcellosi*	brachiopod	dorsal	05/06/2018	08/06/2018	38.4	4.60	0.807	0.098	0.42	2.92	-0.05	13/08/2018	100.2	4.38	-2.60
MPZ 2019/418	CVU_carb_3654	Barranco de la Cañada	BC01b	12.35	2	2	*Choffatirhynchia vasconcellosi*	brachiopod		05/06/2018	08/06/2018	38.9	5.35	0.985	0.053	0.28	3.02	0.11	13/08/2018	99.4	4.06	-1.52
MPZ 2019/418	CVU_carb_3655	Barranco de la Cañada	BC01b	12.35	2	2	*Choffatirhynchia vasconcellosi*	brachiopod		05/06/2018	08/06/2018	38.8	6.27	1.001	0.095	0.49	3.33	0.15	13/08/2018	99.7	3.76	-1.62
MPZ 2019/418	CVU_carb_3656	Barranco de la Cañada	BC01b	12.35	2	2	*Choffatirhynchia vasconcellosi*	brachiopod		05/06/2018	08/06/2018	38.3	6.47	0.945	0.105	0.55	3.22	0.12	13/08/2018	100.2	3.63	-1.75
MPZ 2019/419	CVU_carb_3657	Barranco de la Cañada	BC01b	12.35	2	3	*Choffatirhynchia vasconcellosi*	brachiopod	ventral	05/06/2018	08/06/2018	38.7	6.03	0.970	0.042	0.25	3.57	0.03	13/08/2018	99.9	4.89	-2.83
MPZ 2019/419	CVU_carb_3658	Barranco de la Cañada	BC01b	12.35	2	3	*Choffatirhynchia vasconcellosi*	brachiopod	ventral	05/06/2018	08/06/2018	38.5	6.45	1.022	0.046	0.27	3.43	0.18	13/08/2018	100.4	4.88	-2.69
MPZ 2019/419	CVU_carb_3659	Barranco de la Cañada	BC01b	12.35	2	3	*Choffatirhynchia vasconcellosi*	brachiopod	ventral	05/06/2018	08/06/2018	38.6	6.58	1.034	0.035	0.13	3.62	0.01	13/08/2018	100.2	4.86	-2.79
MPZ 2019/419	CVU_carb_3660	Barranco de la Cañada	BC01b	12.35	2	3	*Choffatirhynchia vasconcellosi*	brachiopod	dorsal	05/06/2018	08/06/2018	38.3	6.65	1.027	0.049	0.22	3.60	0.01	13/08/2018	100.7	4.95	-2.92
MPZ 2019/420	CVU_carb_3661	Barranco de la Cañada	BC01b	12.35	2	4	*Choffatirhynchia vasconcellosi*	brachiopod		05/06/2018	08/06/2018	38.4	5.04	0.739	0.119	0.58	2.84	0.11	13/08/2018	99.8	3.54	-2.31
MPZ 2019/420	CVU_carb_3662	Barranco de la Cañada	BC01b	12.35	2	4	*Choffatirhynchia vasconcellosi*	brachiopod		05/06/2018	08/06/2018	38.7	6.24	0.832	0.102	0.58	3.14	0.10	13/08/2018	99.5	3.34	-2.29
MPZ 2019/420	CVU_carb_3663	Barranco de la Cañada	BC01b	12.35	2	4	*Choffatirhynchia vasconcellosi*	brachiopod		05/06/2018	08/06/2018	38.3	8.17	0.895	0.095	0.43	3.86	0.15	13/08/2018	99.8	3.81	-2.27
MPZ 2019/416	CVU_carb_3838	Barranco de la Cañada	BC01b	12.35	1	1	*Gryphaea* cf. *dumortieri*	bivalve		29/06/2018	06/07/2018	38.9	4.42	0.713	0.106	0.82	1.85	0.06	16/08/2018	100.4	4.93	-2.99
MPZ 2019/416	CVU_carb_3839	Barranco de la Cañada	BC01b	12.35	1	1	*Gryphaea* cf. *dumortieri*	bivalve		29/06/2018	06/07/2018	38.0	7.32	0.655	0.184	2.25	1.67	0.19	16/08/2018	92.3	4.71	-3.00
MPZ 2019/416	CVU_carb_3840	Barranco de la Cañada	BC01b	12.35	1	1	*Gryphaea* cf. *dumortieri*	bivalve		29/06/2018	06/07/2018	38.7	4.35	0.695	0.088	0.73	1.79	0.12	16/08/2018	99.5	5.11	-3.00
MPZ 2019/415	CVU_carb_3597	Barranco de la Cañada	BC01a	12.10	1	1	*Soaresirhynchia bouchardi*	brachiopod	ventral	03/06/2018	08/06/2018	38.9	2.84	0.563	0.035	0.15	1.55	0.11	13/08/2018	100.0	5.17	-2.27
MPZ 2019/415	CVU_carb_3598	Barranco de la Cañada	BC01a	12.10	1	1	*Soaresirhynchia bouchardi*	brachiopod	ventral	03/06/2018	08/06/2018	38.9	2.66	0.551	0.031	0.12	1.51	0.09	13/08/2018	99.3	5.29	-2.33
MPZ 2019/415	CVU_carb_3599	Barranco de la Cañada	BC01a	12.10	1	1	*Soaresirhynchia bouchardi*	brachiopod	ventral	03/06/2018	08/06/2018	39.0	2.60	0.541	0.032	0.12	1.48	0.04	13/08/2018	98.0	5.36	-2.43
MPZ 2019/415	CVU_carb_3600	Barranco de la Cañada	BC01a	12.10	1	1	*Soaresirhynchia bouchardi*	brachiopod	dorsal	03/06/2018	08/06/2018	38.8	3.18	0.675	0.056	0.22	1.63	-0.05	13/08/2018	99.6	4.99	-2.32
MPZ 2019/415	CVU_carb_3601	Barranco de la Cañada	BC01a	12.10	1	1	*Soaresirhynchia bouchardi*	brachiopod	ventral	03/06/2018	08/06/2018	38.9	3.06	0.640	0.073	0.29	1.60	0.10	13/08/2018	99.7	4.86	-2.34
MPZ 2019/415	CVU_carb_3602	Barranco de la Cañada	BC01a	12.10	1	1		bulk rock	bulk rock	03/06/2018	08/06/2018	29.6	29.53	0.480	0.458	18.11	0.95	0.71	13/08/2018	76.4	3.50	-3.86
MPZ 2019/569	CVU_carb_3782	Barranco de la Cañada	C37a	11.30	1	1	*Soaresirhynchia bouchardi*	brachiopod	dorsal	17/06/2018	25/06/2018	38.8	3.62	0.524	0.083	0.85	1.44	0.10	15/08/2018	99.7	4.33	-3.23
MPZ 2019/569	CVU_carb_3783	Barranco de la Cañada	C37a	11.30	1	1	*Soaresirhynchia bouchardi*	brachiopod	ventral	17/06/2018	25/06/2018	38.9	3.87	0.527	0.031	0.22	1.65	0.13	15/08/2018	99.4	4.41	-3.10
MPZ 2019/569	CVU_carb_3808	Barranco de la Cañada	C37a	11.30	1	1	*Soaresirhynchia bouchardi*	brachiopod	ventral	28/06/2018	06/07/2018	39.2	2.60	0.525	0.025	0.23	1.87	0.08	15/08/2018	100.2	4.34	-3.21
MPZ 2019/569	CVU_carb_3809	Barranco de la Cañada	C37a	11.30	1	1	*Soaresirhynchia bouchardi*	brachiopod	dorsal	28/06/2018	06/07/2018	39.1	2.26	0.526	0.038	0.41	1.57	0.03	15/08/2018	101.3	4.54	-2.88
MPZ 2019/569	CVU_carb_3810	Barranco de la Cañada	C37a	11.30	1	1	*Soaresirhynchia bouchardi*	brachiopod	dorsal	28/06/2018	06/07/2018	39.0	3.06	0.531	0.036	0.34	1.99	0.02	15/08/2018	99.5	4.11	-2.98
MPZ 2019/570	CVU_carb_3811	Barranco de la Cañada	C37a	11.30	1	2b	*Soaresirhynchia bouchardi*	brachiopod	dorsal	28/06/2018	06/07/2018	39.3	2.24	0.577	0.027	0.37	1.52	0.09	15/08/2018	99.2	4.47	-2.62
MPZ 2019/570	CVU_carb_3812	Barranco de la Cañada	C37a	11.30	1	2b	*Soaresirhynchia bouchardi*	brachiopod	dorsal	28/06/2018	06/07/2018	39.2	1.79	0.541	0.014	0.12	1.34	-0.07	15/08/2018	99.0	4.59	-2.64
MPZ 2019/571	CVU_carb_3813	Barranco de la Cañada	C37a	11.30	1	3	*Soaresirhynchia bouchardi*	brachiopod	dorsal	28/06/2018	06/07/2018	39.3	3.65	0.504	0.038	0.41	1.86	0.00	15/08/2018	100.8	4.32	-3.73
MPZ 2019/571	CVU_carb_3814	Barranco de la Cañada	C37a	11.30	1	3		bulk rock	bulk rock	28/06/2018	06/07/2018	29.9	33.35	0.459	0.480	22.07	1.21	1.10	15/08/2018	77.3	2.96	-4.21
MPZ 2019/567	CVU_carb_3733	Barranco de la Cañada	C33b	11.10	1	1	*Soaresirhynchia bouchardi*	brachiopod	ventral	09/06/2018	25/06/2018	39.0	2.07	0.555	0.009	0.04	1.03	0.04	14/08/2018	101.4	4.25	-2.85
MPZ 2019/567	CVU_carb_3734	Barranco de la Cañada	C33b	11.10	1	1	*Soaresirhynchia bouchardi*	brachiopod	ventral	09/06/2018	25/06/2018	39.0	2.51	0.563	0.021	0.15	1.26	-0.04	14/08/2018	100.6	4.26	-3.09
MPZ 2019/567	CVU_carb_3735	Barranco de la Cañada	C33b	11.10	1	1	*Soaresirhynchia bouchardi*	brachiopod	dorsal	09/06/2018	25/06/2018	39.2	1.73	0.527	0.010	0.06	1.11	-0.04	14/08/2018	100.9	4.31	-2.87
MPZ 2019/567	CVU_carb_3736	Barranco de la Cañada	C33b	11.10	1	1	*Soaresirhynchia bouchardi*	brachiopod	dorsal	09/06/2018	25/06/2018	39.1	2.23	0.498	0.029	0.21	1.07	-0.07	14/08/2018	99.5	4.15	-3.16
MPZ 2019/567	CVU_carb_3737	Barranco de la Cañada	C33b	11.10	1	1	*Soaresirhynchia bouchardi*	brachiopod	dorsal	09/06/2018	25/06/2018	38.9	2.61	0.536	0.021	0.16	1.18	-0.13	14/08/2018	99.9	4.09	-3.41
MPZ 2019/568	CVU_carb_3738	Barranco de la Cañada	C33b	11.10	1	2	*Soaresirhynchia bouchardi*	brachiopod		09/06/2018	25/06/2018	38.9	2.93	0.547	0.025	0.19	1.52	0.04	14/08/2018	100.9	4.31	-2.68
MPZ 2019/568	CVU_carb_3739	Barranco de la Cañada	C33b	11.10	1	2	*Soaresirhynchia bouchardi*	brachiopod		09/06/2018	25/06/2018	38.8	2.79	0.577	0.007	0.03	1.52	0.03	14/08/2018	100.0	4.28	-2.94
MPZ 2019/568	CVU_carb_3740	Barranco de la Cañada	C33b	11.10	1	2		calcite cement	cement	09/06/2018	25/06/2018	37.9	11.47	0.322	0.799	10.47	0.38	-0.01	14/08/2018	99.9	1.80	-9.66
MPZ 2019/568	CVU_carb_3741	Barranco de la Cañada	C33b	11.10	1	2		bulk rock	bulk rock	09/06/2018	25/06/2018	33.0	25.43	0.416	0.435	11.77	0.89	0.28	14/08/2018	86.3	3.15	-3.52
MPZ 2019/566	CVU_carb_3762	Barranco de la Cañada	C33a	10.80	1	1	*Soaresirhynchia bouchardi*	brachiopod	ventral	17/06/2018	25/06/2018	38.8	2.74	0.529	0.049	0.67	1.24	0.01	15/08/2018	100.6	4.42	-3.49
MPZ 2019/566	CVU_carb_3763	Barranco de la Cañada	C33a	10.80	1	1	*Soaresirhynchia bouchardi*	brachiopod	ventral	17/06/2018	25/06/2018	38.6	2.84	0.551	0.015	0.08	1.36	-0.10	15/08/2018	101.1	4.57	-3.33
MPZ 2019/566	CVU_carb_3764	Barranco de la Cañada	C33a	10.80	1	1	*Soaresirhynchia bouchardi*	brachiopod	ventral	17/06/2018	25/06/2018	38.4	2.85	0.548	0.006	0.03	1.49	0.03	15/08/2018	101.1	4.75	-3.24
MPZ 2019/566	CVU_carb_3765	Barranco de la Cañada	C33a	10.80	1	1	*Soaresirhynchia bouchardi*	brachiopod	ventral	17/06/2018	25/06/2018	38.6	2.80	0.544	0.007	0.03	1.40	0.05	15/08/2018	99.7	4.63	-3.15

MPZ 2019/566	CVU_carb_3766	Barranco de la Cañada	C33a	10.80	1	1		bulk rock	bulk rock from fossil fill	17/06/2018	25/06/2018	34.5	24.59	0.381	0.358	10.55	0.71	0.50	15/08/2018	90.7	3.01	-2.74
MPZ 2019/564	CVU_carb_3706	Barranco de la Cañada	C30	10.20	1	1	*Soaresirhynchia bouchardi*	brachiopod		08/06/2018	25/06/2018	38.2	7.88	1.012	0.055	0.26	4.50	0.07	14/08/2018	98.8	4.72	-2.96
MPZ 2019/565	CVU_carb_3707	Barranco de la Cañada	C30	10.20	1	2	*Soaresirhynchia bouchardi*	brachiopod		08/06/2018	25/06/2018	38.3	6.07	0.917	0.060	0.33	3.62	-0.10	14/08/2018	99.3	4.18	-2.51
MPZ 2019/565	CVU_carb_3708	Barranco de la Cañada	C30	10.20	1	2	*Soaresirhynchia bouchardi*	brachiopod		08/06/2018	25/06/2018	37.9	7.10	0.898	0.066	0.60	3.70	-0.08	14/08/2018	89.8	4.25	-2.29
MPZ 2019/565	CVU_carb_3709	Barranco de la Cañada	C30	10.20	1	2		bulk rock	bulk rock	08/06/2018	25/06/2018	33.9	23.16	0.413	0.417	12.16	0.93	0.42	14/08/2018	88.5	3.78	-3.79
MPZ 2019/557	CVU_carb_3533	Barranco de la Cañada	C28	9.40	1	1	*Soaresirhynchia bouchardi*	brachiopod	dorsal	24/05/2018	04/06/2018	39.4	3.60	0.524	0.026	0.20	1.58	0.02	12/08/2018	100.4	2.88	-2.98
MPZ 2019/557	CVU_carb_3534	Barranco de la Cañada	C28	9.40	1	1	*Soaresirhynchia bouchardi*	brachiopod	dorsal	24/05/2018	04/06/2018	39.1	3.71	0.534	0.027	0.25	1.68	0.03	12/08/2018	100.6	2.92	-2.78
MPZ 2019/557	CVU_carb_3535	Barranco de la Cañada	C28	9.40	1	1	*Soaresirhynchia bouchardi*	brachiopod	dorsal	24/05/2018	04/06/2018	39.2	3.28	0.524	0.016	0.17	1.65	0.25	12/08/2018	101.1	2.90	-2.85
MPZ 2019/557	CVU_carb_3536	Barranco de la Cañada	C28	9.40	1	1		bulk rock	bulk rock	24/05/2018	04/06/2018	31.6	27.33	0.408	0.476	18.21	2.28	0.55	12/08/2018	80.4	2.01	-4.11
MPZ 2019/558	CVU_carb_3537	Barranco de la Cañada	C28	9.40	1	2	*Soaresirhynchia bouchardi*	brachiopod	ventral	24/05/2018	04/06/2018	39.4	2.95	0.614	0.037	0.29	1.45	-0.03	12/08/2018	100.3	2.63	-2.73
MPZ 2019/558	CVU_carb_3538	Barranco de la Cañada	C28	9.40	1	2	*Soaresirhynchia bouchardi*	brachiopod	ventral	24/05/2018	04/06/2018	39.2	2.21	0.525	0.026	0.20	1.21	0.12	12/08/2018	100.6	2.84	-2.67
MPZ 2019/558	CVU_carb_3539	Barranco de la Cañada	C28	9.40	1	2	*Soaresirhynchia bouchardi*	brachiopod	ventral	24/05/2018	04/06/2018	39.3	2.57	0.511	0.030	0.23	1.22	0.13	12/08/2018	100.9	2.87	-2.88
MPZ 2019/558	CVU_carb_3540	Barranco de la Cañada	C28	9.40	1	2	*Soaresirhynchia bouchardi*	brachiopod	dorsal	24/05/2018	04/06/2018	39.1	2.85	0.547	0.045	0.31	1.35	0.22	12/08/2018	101.1	2.66	-2.89
MPZ 2019/558	CVU_carb_3541	Barranco de la Cañada	C28	9.40	1	2		calcite cement	calcite cement	24/05/2018	04/06/2018	38.5	13.70	0.344	0.651	7.76	0.78	0.14	12/08/2018	98.6	1.76	-8.63
MPZ 2019/559	CVU_carb_3542	Barranco de la Cañada	C28	9.40	1	3	*Soaresirhynchia bouchardi*	brachiopod	ventral	24/05/2018	04/06/2018	39.2	2.60	0.533	0.009	0.08	1.42	0.09	12/08/2018	100.3	2.76	-2.65
MPZ 2019/559	CVU_carb_3543	Barranco de la Cañada	C28	9.40	1	3	*Soaresirhynchia bouchardi*	brachiopod	ventral	24/05/2018	04/06/2018	39.4	2.47	0.544	0.010	0.08	1.24	0.09	12/08/2018	101.8	2.89	-2.53
MPZ 2019/559	CVU_carb_3544	Barranco de la Cañada	C28	9.40	1	3	*Soaresirhynchia bouchardi*	brachiopod	ventral	24/05/2018	04/06/2018	39.3	2.41	0.544	0.005	0.06	1.25	0.07	12/08/2018	101.6	2.82	-2.49
MPZ 2019/559	CVU_carb_3545	Barranco de la Cañada	C28	9.40	1	3	*Soaresirhynchia bouchardi*	brachiopod	dorsal	24/05/2018	04/06/2018	39.5	2.86	0.520	0.017	0.14	1.25	-0.08	12/08/2018	100.7	2.84	-2.70
MPZ 2019/559	CVU_carb_3546	Barranco de la Cañada	C28	9.40	1	3	*Soaresirhynchia bouchardi*	brachiopod	dorsal	24/05/2018	04/06/2018	39.1	2.87	0.536	0.010	0.06	1.39	0.02	12/08/2018	101.9	2.81	-2.51
MPZ 2019/559	CVU_carb_3547	Barranco de la Cañada	C28	9.40	1	3	*Soaresirhynchia bouchardi*	brachiopod	dorsal	24/05/2018	04/06/2018	39.3	2.85	0.533	0.013	0.10	1.04	0.06	12/08/2018	101.3	2.73	-2.79
MPZ 2019/560	CVU_carb_3548	Barranco de la Cañada	C28	9.40	1	4	*Soaresirhynchia bouchardi*	brachiopod	ventral	24/05/2018	04/06/2018	39.0	2.38	0.548	0.016	0.12	1.27	0.02	12/08/2018	97.4	2.59	-3.57
MPZ 2019/560	CVU_carb_3549	Barranco de la Cañada	C28	9.40	1	4	*Soaresirhynchia bouchardi*	brachiopod	ventral	24/05/2018	04/06/2018	39.0	2.83	0.534	0.008	0.04	1.45	0.03	12/08/2018	100.6	2.67	-3.52
MPZ 2019/560	CVU_carb_3550	Barranco de la Cañada	C28	9.40	1	4	*Soaresirhynchia bouchardi*	brachiopod	ventral	24/05/2018	04/06/2018	39.5	2.71	0.566	0.003	0.01	1.17	0.10	12/08/2018	100.3	2.68	-3.38
MPZ 2019/561	CVU_carb_3551	Barranco de la Cañada	C28	9.40	1	5	*Soaresirhynchia bouchardi*	brachiopod	ventral	28/05/2018	04/06/2018	38.9	2.34	0.542	0.056	0.45	1.19	-0.02	12/08/2018	100.9	2.36	-3.40
MPZ 2019/561	CVU_carb_3552	Barranco de la Cañada	C28	9.40	1	5	*Soaresirhynchia bouchardi*	brachiopod	ventral	28/05/2018	08/06/2018	39.6	2.37	0.511	0.027	0.21	1.19	0.18	12/08/2018	101.3	2.51	-3.16
MPZ 2019/561	CVU_carb_3553	Barranco de la Cañada	C28	9.40	1	5	*Soaresirhynchia bouchardi*	brachiopod	ventral	28/05/2018	08/06/2018	39.5	2.37	0.513	0.019	0.12	1.19	0.14	12/08/2018	100.5	2.50	-3.21
MPZ 2019/562	CVU_carb_3554	Barranco de la Cañada	C28	9.40	1	6	*Soaresirhynchia bouchardi*	brachiopod	ventral	28/05/2018	08/06/2018	39.4	2.12	0.524	0.009	0.06	1.18	0.10	12/08/2018	101.3	2.46	-2.84
MPZ 2019/562	CVU_carb_3555	Barranco de la Cañada	C28	9.40	1	6	*Soaresirhynchia bouchardi*	brachiopod	ventral	28/05/2018	08/06/2018	39.6	2.10	0.528	0.009	0.06	1.25	0.18	12/08/2018	101.6	2.54	-2.90
MPZ 2019/562	CVU_carb_3556	Barranco de la Cañada	C28	9.40	1	6	*Soaresirhynchia bouchardi*	brachiopod	dorsal	28/05/2018	08/06/2018	38.7	3.09	0.526	0.071	0.79	1.22	0.21	12/08/2018	101.4	2.33	-3.61
MPZ 2019/563	CVU_carb_3557	Barranco de la Cañada	C28	9.40	1	7	*Soaresirhynchia bouchardi*	brachiopod	dorsal	28/05/2018	08/06/2018	39.7	1.84	0.527	0.037	0.27	1.25	0.11	12/08/2018	100.0	2.55	-3.25
MPZ 2019/563	CVU_carb_3558	Barranco de la Cañada	C28	9.40	1	7	*Soaresirhynchia bouchardi*	brachiopod	dorsal	28/05/2018	08/06/2018	39.1	2.29	0.542	0.032	0.22	1.24	0.19	12/08/2018	99.8	2.69	-3.01
MPZ 2019/563	CVU_carb_3559	Barranco de la Cañada	C28	9.40	1	7	*Soaresirhynchia bouchardi*	brachiopod	dorsal	28/05/2018	08/06/2018	39.1	1.99	0.545	0.015	0.08	1.24	0.09	12/08/2018	101.0	2.81	-2.96
MPZ 2019/552	CVU_carb_3710	Barranco de la Cañada	C26	8.90	1	1	*Soaresirhynchia bouchardi*	brachiopod	ventral	08/06/2018	25/06/2018	38.4	2.75	0.517	0.029	0.25	1.25	0.03	14/08/2018	99.9	2.40	-3.52
MPZ 2019/552	CVU_carb_3711	Barranco de la Cañada	C26	8.90	1	1	*Soaresirhynchia bouchardi*	brachiopod	ventral	08/06/2018	25/06/2018	38.5	2.45	0.559	0.012	0.09	1.28	0.05	14/08/2018	98.1	2.31	-3.32
MPZ 2019/552	CVU_carb_3712	Barranco de la Cañada	C26	8.90	1	1	*Soaresirhynchia bouchardi*	brachiopod	ventral	08/06/2018	25/06/2018	38.8	2.33	0.536	0.011	0.08	1.22	0.06	14/08/2018	100.5	2.30	-3.45
MPZ 2019/553	CVU_carb_3713	Barranco de la Cañada	C26	8.90	1	2	*Soaresirhynchia bouchardi*	brachiopod	ventral	08/06/2018	25/06/2018	39.1	2.43	0.557	0.011	0.08	1.34	0.14	14/08/2018	100.2	2.34	-3.42
MPZ 2019/553	CVU_carb_3714	Barranco de la Cañada	C26	8.90	1	2	*Soaresirhynchia bouchardi*	brachiopod	dorsal	09/06/2018	25/06/2018	38.6	2.51	0.504	0.034	0.39	1.18	0.02	14/08/2018	97.1	2.29	-3.46
MPZ 2019/553	CVU_carb_3715	Barranco de la Cañada	C26	8.90	1	2	*Soaresirhynchia bouchardi*	brachiopod	dorsal	09/06/2018	25/06/2018	38.8	2.49	0.527	0.013	0.10	1.41	0.09	14/08/2018	101.2	2.40	-3.24
MPZ 2019/553	CVU_carb_3716	Barranco de la Cañada	C26	8.90	1	2	*Soaresirhynchia bouchardi*	brachiopod	dorsal	09/06/2018	25/06/2018	38.8	2.70	0.538	0.013	0.09	1.65	-0.15	14/08/2018	101.6	2.33	-3.54
MPZ 2019/553	CVU_carb_3717	Barranco de la Cañada	C26	8.90	1	2	*Soaresirhynchia bouchardi*	brachiopod	ventral	09/06/2018	25/06/2018	38.8	3.18	0.556	0.013	0.14	1.49	0.09	14/08/2018	99.8	2.38	-3.39
MPZ 2019/553	CVU_carb_3718	Barranco de la Cañada	C26	8.90	1	2	*Soaresirhynchia bouchardi*	brachiopod	ventral	09/06/2018	25/06/2018	38.7	2.92	0.539	0.008	0.05	1.63	-0.07	14/08/2018	100.9	2.31	-3.34
MPZ 2019/553	CVU_carb_3719	Barranco de la Cañada	C26	8.90	1	2		calcite cement	geopetal cement	09/06/2018	25/06/2018	37.8	12.32	0.346	0.786	9.57	0.57	-0.02	14/08/2018	99.1	1.65	-9.73
MPZ 2019/553	CVU_carb_3720	Barranco de la Cañada	C26	8.90	1	2		bulk rock	bulk rock, fossil interior	09/06/2018	25/06/2018	31.2	21.37	0.313	0.423	11.62	0.79	0.38	14/08/2018	94.3	1.94	-3.20
MPZ 2019/553	CVU_carb_3720	Barranco de la Cañada	C26	8.90	1	2		bulk rock	bulk rock, fossil interior	09/06/2018									17/08/2018	94.6	1.96	-3.03
MPZ 2019/554	CVU_carb_3721	Barranco de la Cañada	C26	8.90	1	3	*Soaresirhynchia bouchardi*	brachiopod	ventral	09/06/2018	25/06/2018	38.8	3.40	0.550	0.013	0.09	1.61	0.05	14/08/2018	100.5	2.75	-2.60
MPZ 2019/554	CVU_carb_3722	Barranco de la Cañada	C26	8.90	1	3	*Soaresirhynchia bouchardi*	brachiopod	ventral	09/06/2018	25/06/2018	39.0	3.26	0.556	0.013	0.09	1.36	-0.03	14/08/2018	100.5	2.80	-2.56
MPZ 2019/554	CVU_carb_3723	Barranco de la Cañada	C26	8.90	1	3	*Soaresirhynchia bouchardi*	brachiopod	ventral	09/06/2018	25/06/2018	39.1	2.83	0.554	0.014	0.08	1.33	-0.02	14/08/2018	99.6	2.83	-2.64
MPZ 2019/554	CVU_carb_3724	Barranco de la Cañada	C26	8.90	1	3	*Soaresirhynchia bouchardi*	brachiopod	dorsal	09/06/2018	25/06/2018	39.0	3.53	0.555	0.020	0.12	1.54	0.03	14/08/2018	101.2	2.72	-2.59
MPZ 2019/554	CVU_carb_3725	Barranco de la Cañada	C26	8.90	1	3	*Soaresirhynchia bouchardi*	brachiopod	dorsal	09/06/2018	25/06/2018	39.3	3.83	0.559	0.013	0.07	1.72	-0.04	14/08/2018	100.6	2.86	-2.52
MPZ 2019/554	CVU_carb_3726	Barranco de la Cañada	C26	8.90	1	3	*Soaresirhynchia bouchardi*	brachiopod	dorsal	09/06/2018	25/06/2018	39.2	3.50	0.558	0.011	0.06	1.55	-0.01	14/08/2018	101.0	2.81	-2.37
MPZ 2019/555	CVU_carb_3727	Barranco de la Cañada	C26	8.90	1	4	*Soaresirhynchia bouchardi*	brachiopod	ventral	09/06/2018	25/06/2018	39.0	1.92	0.516	0.008	0.03	1.00	0.00	14/08/2018	100.7	3.05	-3.10
MPZ 2019/555	CVU_carb_3728	Barranco de la Cañada	C26	8.90	1	4	*Soaresirhynchia bouchardi*	brachiopod	ventral	09/06/2018	25/06/2018	39.2	2.06	0.526	0.007	0.03	0.96	0.01	14/08/2018	101.5	3.01	-3.05
MPZ 2019/555	CVU_carb_3729	Barranco de la Cañada	C26	8.90	1	4	*Soaresirhynchia bouchardi*	brachiopod	ventral	09/06/2018	25/06/2018	39.0	2.36	0.530	0.009	0.04	1.26	0.00	14/08/2018	100.9	2.88	-2.96
MPZ 2019/556	CVU_carb_3730	Barranco de la Cañada	C26	8.90	1	5	*Soaresirhynchia bouchardi*	brachiopod	ventral	09/06/2018	25/06/2018	39.1	2.09	0.543	0.007	0.03	1.21	0.01	14/08/2018	100.2	2.33	-3.22
MPZ 2019/556	CVU_carb_3731	Barranco de la Cañada	C26	8.90	1	5	*Soaresirhynchia bouchardi*	brachiopod	ventral	09/06/2018	25/06/2018	39.5	2.14	0.528	0.007	0.04	1.06	0.02	14/08/2018	101.6	2.33	-3.24
MPZ 2019/556	CVU_carb_3732	Barranco de la Cañada	C26	8.90	1	5	*Soaresirhynchia bouchardi*	brachiopod	ventral	09/06/2018	25/06/2018	38.8	1.94	0.553	0.008	0.07	1.14	-0.02	14/08/2018	99.9	2.24	-3.18
MPZ 2019/549	CVU_carb_3767	Barranco de la Cañada	C25b	8.70	1	1	*Soaresirhynchia bouchardi*	brachiopod		17/06/2018	25/06/2018	38.7	2.65	0.509	0.064	0.47	1.09	0.13	15/08/2018	97.5	2.45	-3.48
MPZ 2019/549	CVU_carb_3768	Barranco de la Cañada	C25b	8.70	1	1		bulk rock	bulk rock	17/06/2018	25/06/2018	31.3	32.14	0.387	0.494	16.11	1.53	0.67	15/08/2018	82.8	1.90	-3.97
MPZ 2019/550	CVU_carb_3769	Barranco de la Cañada	C25b	8.70	1	2	*Soaresirhynchia bouchardi*	brachiopod	ventral	17/06/2018	25/06/2018	38.7	2.36	0.537	0.035	0.30	1.37	0.09	15/08/2018	100.5	1.67	-3.57
MPZ 2019/550	CVU_carb_3770	Barranco de la Cañada	C25b	8.70	1	2	*Soaresirhynchia bouchardi*	brachiopod	ventral	17/06/2018	25/06/2018	38.8	2.38	0.532	0.032	0.25	0.99	-0.04	15/08/2018	98.3	1.79	-3.41
MPZ 2019/550	CVU_carb_3771	Barranco de la Cañada	C25b	8.70	1	2	*Soaresirhynchia bouchardi*	brachiopod	ventral	17/06/2018	25/06/2018	38.9	2.19	0.520	0.026	0.15	1.03	0.09	15/08/2018	100.0	1.72	-3.52
MPZ 2019/551	CVU_carb_3772	Barranco de la Cañada	C25b	8.70	1	3	*Soaresirhynchia bouchardi*	brachiopod		17/06/2018	25/06/2018	38.9	2.73	0.515	0.065	0.34	1.28	0.14	15/08/2018	100.1	2.21	-3.48
MPZ 2019/545	CVU_carb_3428	Barranco de la Cañada	C25a	8.40	1	3	*Soaresirhynchia bouchardi*	brachiopod		10/04/2018	01/06/2018	39.2	2.30	0.453	0.026	0.12	1.05	0.02	10/08/2018	97.4	2.30	-3.17
MPZ 2019/546	CVU_carb_3471	Barranco de la Cañada	C25a	8.40	2	1	*Soaresirhynchia bouchardi*	brachiopod	ventral	29/04/2018	04/06/2018	38.9	2.44	0.523	0.026	0.14	1.39	0.10	10/08/2018	100.1	2.12	-3.06
MPZ 2019/546	CVU_carb_3472	Barranco de la Cañada	C25a	8.40	2	1	*Soaresirhynchia bouchardi*	brachiopod	ventral	29/04/2018	04/06/2018	39.3	2.42	0.491	0.007	0.04	1.34	0.05	10/08/2018	100.7	2.30	-2.92
MPZ 2019/546	CVU_carb_3473	Barranco de la Cañada	C25a	8.40	2	1	*Soaresirhynchia bouchardi*	brachiopod	ventral	29/04/2018	04/06/2018	39.3	2.34	0.495	0.024	0.18	1.34	-0.01	10/08/2018	99.9	2.09	-3.03
MPZ 2019/546	CVU_carb_3474	Barranco de la Cañada	C25a	8.40	2	1	*Soaresirhynchia bouchardi*	brachiopod	dorsal	29/04/2018	04/06/2018	39.5	2.39	0.506	0.024	0.13	1.33	-0.01	10/08/2018	100.7	2.09	-2.98
MPZ 2019/546	CVU_carb_3475	Barranco de la Cañada	C25a	8.40	2	1	*Soaresirhynchia bouchardi*	brachiopod	dorsal	29/04/2018	04/06/2018	39.1	2.41	0.510	0.021	0.13	1.33	-0.02	10/08/2018	100.3	2.10	-2.99
MPZ 2019/546	CVU_carb_3476	Barranco de la Cañada	C25a	8.40	2	1	*Soaresirhynchia bouchardi*	brachiopod	dorsal	29/04/2018	04/06/2018	39.2	2.74	0.516	0.023	0.15	1.35	0.03	10/08/2018	100.4	2.11	-3.15
MPZ 2019/547	CVU_carb_3477	Barranco de la Cañada	C25a	8.40	2	2b	*Soaresirhynchia bouchardi*	brachiopod		29/04/2018	04/06/2018	39.2	3.42	0.496	0.061	0.37	1.43	0.08	17/08/2018	99.2	2.14	-3.07
MPZ 2019/547	CVU_carb_3478	Barranco de la Cañada	C25a	8.40	2	2b	*Soaresirhynchia bouchardi*	brachiopod		29/04/2018	04/06/2018	39.1	3.20	0.457	0.058	0.39	1.15	0.01	10/08/2018	100.8	2.30	-3.22
MPZ 2019/547	CVU_carb_3479	Barranco de la Cañada	C25a	8.40	2	2b	*Soaresirhynchia bouchardi*	brachiopod		29/04/2018	04/06/2018	38.6	3.97	0.482	0.028	0.39	1.74	-0.09	10/08/2018	100.9	2.17	-3.13
MPZ 2019/548	CVU_carb_3480	Barranco de la Cañada	C25a	8.40	2	3b	*Soaresirhynchia bouchardi*	brachiopod	ventral(?)	29/04/2018	04/06/2018	39.3	2.62	0.541	0.036	0.21	1.32	0.02	10/08/2018	101.7	1.97	-3.23
MPZ 2019/548	CVU_carb_3481	Barranco de la Cañada	C25a	8.40	2	3b	*Soaresirhynchia bouchardi*	brachiopod	ventral(?)	29/04/2018	04/06/2018	35.0	2.78	0.530	0.042	0.24	1.54	0.00	10/08/2018	100.7	1.99	-3.33
MPZ 2019/548	CVU_carb_3482	Barranco de la Cañada	C25a	8.40	2	3b	*Soaresirhynchia bouchardi*	brachiopod	ventral(?)	29/04/2018	04/06/2018	39.1	2.79	0.513	0.046	0.29	1.49	0.10	10/08/2018	100.1	2.01	-3.39
MPZ 2019/548	CVU_carb_3483	Barranco de la Cañada	C25a	8.40	2	3b		bulk rock	bulk rock	29/04/2018	04/06/2018	30.8	30.21	0.347	0.519	18.74	1.11	0.48	10/08/2018	80.5	1.53	-4.34
MPZ 2019/543	CVU_carb_3603	Barranco de la Cañada	C25a	8.40	1	1	*Soaresirhynchia bouchardi*	brachiopod		03/06/2018	08/06/2018	38.7	1.87	0.552	0.049	0.34	0.95	0.04	13/08/2018	99.5	1.85	-3.46
MPZ 2019/543	CVU_carb_3604	Barranco de la Cañada	C25a	8.40	1	1	*Soaresirhynchia bouchardi*	brachiopod		03/06/2018	08/06/2018	39.0	1.93	0.538	0.032	0.20	1.42	0.09	13/08/2018	99.9	1.93	-3.51
MPZ 2019/543	CVU_carb_3605	Barranco de la Cañada	C25a	8.40	1	1	*Soaresirhynchia bouchardi*	brachiopod		03/06/2018	08/06/2018	39.4	3.39	0.624	0.087	0.98	1.31	0.12	13/08/2018	100.0	2.12	-3.43
MPZ 2019/544	CVU_carb_3606	Barranco de la Cañada	C25a	8.40	1	2	*Soaresirhynchia bouchardi*	brachiopod	ventral	03/06/2018	08/06/2018	38.8	2.48	0.483	0.044	0.19	1.28	0.13	13/08/2018	100.5	2.44	-3.17
MPZ 2019/544	CVU_carb_3607	Barranco de la Cañada	C25a	8.40	1	2	*Soaresirhynchia bouchardi*	brachiopod	ventral	03/06/2018	08/06/2018	38.9	2.44	0.514	0.013	0.06	1.39	-0.09	13/08/2018	100.3	2.41	-3.12
MPZ 2019/544	CVU_carb_3608	Barranco de la Cañada	C25a	8.40	1	2	*Soaresirhynchia bouchardi*	brachiopod	ventral	03/06/2018	08/06/2018	39.1	2.29	0.532	0.015	0.07	1.38	0.08	13/08/2018	100.1	2.50	-3.27
MPZ 2019/544	CVU_carb_3609	Barranco de la Cañada	C25a	8.40	1	2	*Soaresirhynchia bouchardi*	brachiopod	dorsal	03/06/2018	08/06/2018	39.1	2.21	0.486	0.052	0.31	1.18	0.02	13/08/2018	99.9	2.36	-3.22
MPZ 2019/544	CVU_carb_3610	Barranco de la Cañada	C25a	8.40	1	2	*Soaresirhynchia bouchardi*	brachiopod	dorsal	03/06/2018	08/06/2018	39.0	2.43	0.495	0.011	0.06	1.42	0.00	13/08/2018	99.7	2.49	-3.22
MPZ 2019/544	CVU_carb_3611	Barranco de la Cañada	C25a	8.40	1	2	*Soaresirhynchia bouchardi*	brachiopod	dorsal	03/06/2018	08/06/2018	39.3	2.17	0.494	0.011	0.06	1.37	-0.07	13/08/2018	100.0	2.55	-3.29
MPZ 2019/545	CVU_carb_3612	Barranco de la Cañada	C25a	8.40	1	3	*Soaresirhynchia bouchardi*	brachiopod		03/06/2018	08/06/2018	39.2	2.11	0.502	0.057	0.40	1.13	0.19	13/08/2018	100.1	2.19	-3.42
MPZ 2019/545	CVU_carb_3613	Barranco de la Cañada	C25a	8.40	1	3	*Soaresirhynchia bouchardi*	brachiopod		03/06/2018	08/06/2018	39.5	2.26	0.508	0.029	0.21	1.25	0.18	13/08/2018	100.0	2.11	-3.26
MPZ 2019/545	CVU_carb_3614	Barranco de la Cañada	C25a	8.40	1	3	*Soaresirhynchia bouchardi*	brachiopod		03/06/2018	08/06/2018	39.2	1.91	0.492	0.029	0.20	1.26	0.19	13/08/2018	100.1	2.26	-3.38
MPZ 2019/545	CVU_carb_3615	Barranco de la Cañada	C25a	8.40	1	3		bulk rock	bulk rock	03/06/2018	08/06/2018	33.6	24.93	0.335	0.487	11.48	1.12	0.95	13/08/2018	87.7	1.69	-4.04
MPZ 2019/541	CVU_carb_3925	Barranco de la Cañada	C22	7.80	1	2	*Gryphaea* cf. *dumortieri*	bivalve		06/07/2018	02/08/2018	39.2	3.39	0.734	0.048	0.68	1.33	0.19	17/08/2018	100.6	0.31	-3.26
MPZ 2019/541	CVU_carb_3926	Barranco de la Cañada	C22	7.80	1	2	*Gryphaea* cf. *dumortieri*	bivalve		06/07/2018	02/08/2018	39.2	3.13	0.769	0.037	0.56	1.10	0.30	17/08/2018	100.7	0.37	-3.28
MPZ 2019/541	CVU_carb_3927	Barranco de la Cañada	C22	7.80	1	2	*Gryphaea* cf. *dumortieri*	bivalve		06/07/2018	02/08/2018	39.1	3.40	0.762	0.059	0.78	1.21	0.23	17/08/2018	100.1	0.37	-3.48
MPZ 2019/541	CVU_carb_3928	Barranco de la Cañada	C22	7.80	1	2		bulk rock	bulk rock	06/07/2018	02/08/2018	21.5	101.91	0.483	1.046	51.88	2.25	1.00	17/08/2018	58.5	0.34	-4.25
MPZ 2019/542	CVU_carb_3929	Barranco de la Cañada	C22	7.80	1	3	*Gryphaea* cf. *dumortieri*	bivalve		06/07/2018	02/08/2018	39.1	3.10	0.765	0.040	0.49	1.88	0.24	17/08/2018	100.6	0.18	-3.19
MPZ 2019/542	CVU_carb_3930	Barranco de la Cañada	C22	7.80	1	3	*Gryphaea* cf. *dumortieri*	bivalve		06/07/2018	02/08/2018	39.0	3.16	0.762	0.052	0.59	1.93	0.18	17/08/2018	100.6	0.11	-3.57
MPZ 2019/539	CVU_carb_3921	Barranco de la Cañada	C20	6.70	1	1	*Gryphaea* (*B.*) *sublobata*	bivalve		06/07/2018	02/08/2018	38.3	5.11	0.741	0.027	0.33	2.89	0.20	17/08/2018	100.2	1.72	-2.90
MPZ 2019/539	CVU_carb_3922	Barranco de la Cañada	C20	6.70	1	1	*Gryphaea* (*B.*) *sublobata*	bivalve		06/07/2018	02/08/2018	39.0	3.64	0.792	0.047	0.51	3.49	0.10	17/08/2018	100.5	1.79	-3.16
MPZ 2019/539	CVU_carb_3923	Barranco de la Cañada	C20	6.70	1	1	*Gryphaea* (*B.*) *sublobata*	bivalve		06/07/2018	02/08/2018	39.2	3.94	0.757	0.086	1.01	2.94	-0.03	17/08/2018	100.0	1.73	-3.07
MPZ 2019/539	CVU_carb_3924	Barranco de la Cañada	C20	6.70	1	1		bulk rock	bulk rock	06/07/2018	02/08/2018	16.5	158.31	0.706	1.070	83.43	3.32	0.98	17/08/2018	45.8	0.91	-4.02

MPZ 2019/535	CVU_carb_3433	Barranco de la Cañada	C19	6.20	3	1	*Gryphaea (B.) sublobata*	bivalve		10/04/2018	04/06/2018	38.8	3.71	0.710	0.046	0.51	4.95	-0.02	10/08/2018	99.4	2.98	-2.18
MPZ 2019/538	CVU_carb_3616	Barranco de la Cañada	C19	6.20	4	1	*Quadratirhynchia attenuata*	brachiopod	ventral	04/06/2018	08/06/2018	38.2	4.60	0.951	0.025	0.21	5.08	0.20	13/08/2018	98.1	2.24	-2.70
MPZ 2019/538	CVU_carb_3617	Barranco de la Cañada	C19	6.20	4	1	*Quadratirhynchia attenuata*	brachiopod	ventral	04/06/2018	08/06/2018	38.5	4.55	1.001	0.016	0.16	4.79	0.10	13/08/2018	97.8	2.34	-2.49
MPZ 2019/538	CVU_carb_3618	Barranco de la Cañada	C19	6.20	4	1	*Quadratirhynchia attenuata*	brachiopod	ventral	04/06/2018	08/06/2018	38.5	5.20	1.033	0.016	0.19	6.12	0.15	13/08/2018	99.1	2.06	-2.51
MPZ 2019/538	CVU_carb_3619	Barranco de la Cañada	C19	6.20	4	1	*Quadratirhynchia attenuata*	brachiopod	dorsal	04/06/2018	08/06/2018	38.3	6.06	0.983	0.043	0.56	5.54	0.13	13/08/2018	98.6	2.14	-2.61
MPZ 2019/538	CVU_carb_3620	Barranco de la Cañada	C19	6.20	4	1	*Quadratirhynchia attenuata*	brachiopod	dorsal	04/06/2018	08/06/2018	36.0	5.46	0.960	0.038	0.40	5.53	0.18	13/08/2018	98.0	2.06	-2.76
MPZ 2019/538	CVU_carb_3621	Barranco de la Cañada	C19	6.20	4	1	*Quadratirhynchia attenuata*	brachiopod	dorsal	04/06/2018	08/06/2018	38.4	5.22	0.989	0.031	0.32	5.21	0.17	13/08/2018	98.3	2.08	-2.49
MPZ 2019/533	CVU_carb_3804	Barranco de la Cañada	C19	6.20	2	1	*Tetrarhynchia subconcinna*	brachiopod	ventral	28/06/2018	06/07/2018	38.3	6.44	0.996	0.055	0.54	5.38	-0.02	15/08/2018	97.1	2.04	-2.61
MPZ 2019/533	CVU_carb_3805	Barranco de la Cañada	C19	6.20	2	1	*Tetrarhynchia subconcinna*	brachiopod	ventral	28/06/2018	06/07/2018	38.6	6.16	0.972	0.067	0.91	5.38	-0.02	15/08/2018	96.7	1.79	-2.73
MPZ 2019/533	CVU_carb_3806	Barranco de la Cañada	C19	6.20	2	1	*Tetrarhynchia subconcinna*	brachiopod	ventral	28/06/2018	06/07/2018	38.4	6.83	1.066	0.043	0.56	6.26	-0.01	15/08/2018	99.1	1.72	-2.53
MPZ 2019/533	CVU_carb_3807	Barranco de la Cañada	C19	6.20	2	1		bulk rock	bulk rock	28/06/2018	06/07/2018	18.4	181.04	0.593	0.999	80.16	3.93	2.47	15/08/2018	52.8	1.30	-3.05
MPZ 2019/535	CVU_carb_3876	Barranco de la Cañada	C19	6.20	3	1	*Gryphaea (B.) sublobata*	bivalve		29/06/2018	06/07/2018	38.9	3.47	0.715	0.044	0.61	5.62	-0.01	16/08/2018	99.3	2.86	-2.61
MPZ 2019/535	CVU_carb_3877	Barranco de la Cañada	C19	6.20	3	1	*Gryphaea (B.) sublobata*	bivalve		02/07/2018	06/07/2018	39.1	3.13	0.670	0.043	0.50	3.49	0.02	16/08/2018	100.3	3.02	-2.42
MPZ 2019/535	CVU_carb_3878	Barranco de la Cañada	C19	6.20	3	1	*Gryphaea (B.) sublobata*	bivalve		02/07/2018	06/07/2018	38.8	3.68	0.752	0.031	0.40	3.27	-0.07	16/08/2018	101.0	3.46	-2.06
MPZ 2019/536	CVU_carb_3879	Barranco de la Cañada	C19	6.20	3	2	*Gryphaea (B.) sublobata*	bivalve		02/07/2018	06/07/2018	38.9	2.86	0.707	0.022	0.27	5.93	0.11	16/08/2018	98.4	2.98	-2.57
MPZ 2019/536	CVU_carb_3880	Barranco de la Cañada	C19	6.20	3	2	*Gryphaea (B.) sublobata*	bivalve		02/07/2018	06/07/2018	39.0	2.96	0.719	0.024	0.28	6.04	0.05	16/08/2018	99.5	2.95	-2.25
MPZ 2019/536	CVU_carb_3881	Barranco de la Cañada	C19	6.20	3	2	*Gryphaea (B.) sublobata*	bivalve		02/07/2018	06/07/2018	38.8	3.82	0.746	0.017	0.21	6.20	-0.01	16/08/2018	100.3	3.10	-2.65
MPZ 2019/527	CVU_carb_3431	Barranco de la Cañada	C18c	5.90	1	3	*Quadratirhynchia* aff. *attenuata*	brachiopod		10/04/2018	01/06/2018	38.4	3.85	1.015	0.016	0.18	4.77	0.00	10/08/2018	98.7	2.44	-1.66
MPZ 2019/525	CVU_carb_3664	Barranco de la Cañada	C18c	5.90	1	1	*Quadratirhynchia* aff. *attenuata*	brachiopod	ventral	05/06/2018	08/06/2018	38.5	6.61	1.165	0.009	0.08	5.23	0.02	13/08/2018	98.6	2.46	-2.41
MPZ 2019/525	CVU_carb_3665	Barranco de la Cañada	C18c	5.90	1	1	*Quadratirhynchia* aff. *attenuata*	brachiopod	ventral	05/06/2018	08/06/2018	38.3	6.26	1.082	0.014	0.16	5.59	0.07	13/08/2018	98.9	2.63	-2.41
MPZ 2019/525	CVU_carb_3666	Barranco de la Cañada	C18c	5.90	1	1	*Quadratirhynchia* aff. *attenuata*	brachiopod	ventral	05/06/2018	08/06/2018	38.3	6.08	1.084	0.011	0.10	5.63	0.16	13/08/2018	98.2	2.74	-2.42
MPZ 2019/525	CVU_carb_3667	Barranco de la Cañada	C18c	5.90	1	1	*Quadratirhynchia* aff. *attenuata*	brachiopod	dorsal	05/06/2018	08/06/2018	38.5	6.62	1.078	0.014	0.17	5.61	0.04	13/08/2018	99.4	2.54	-2.31
MPZ 2019/525	CVU_carb_3668	Barranco de la Cañada	C18c	5.90	1	1	*Quadratirhynchia* aff. *attenuata*	brachiopod	dorsal	05/06/2018	08/06/2018	38.2	6.98	1.110	0.012	0.15	5.65	0.05	14/08/2018	96.6	2.68	-1.92
MPZ 2019/525	CVU_carb_3669	Barranco de la Cañada	C18c	5.90	1	1	*Quadratirhynchia* aff. *attenuata*	brachiopod	dorsal	05/06/2018	08/06/2018	38.4	6.01	1.095	0.013	0.15	4.91	0.06	14/08/2018	97.9	2.59	-2.23
MPZ 2019/526	CVU_carb_3670	Barranco de la Cañada	C18c	5.90	1	2	*Quadratirhynchia* aff. *attenuata*	brachiopod	ventral	05/06/2018	08/06/2018	38.1	2.99	0.932	0.017	0.24	3.52	0.12	14/08/2018	98.6	3.30	-1.73
MPZ 2019/526	CVU_carb_3671	Barranco de la Cañada	C18c	5.90	1	2	*Quadratirhynchia* aff. *attenuata*	brachiopod	ventral	05/06/2018	08/06/2018	38.5	3.59	0.951	0.018	0.26	3.88	0.03	14/08/2018	99.4	3.46	-1.79
MPZ 2019/526	CVU_carb_3672	Barranco de la Cañada	C18c	5.90	1	2	*Quadratirhynchia* aff. *attenuata*	brachiopod	ventral	05/06/2018	25/06/2018	38.0	3.56	0.950	0.024	0.31	3.51	-0.03	14/08/2018	98.4	3.18	-1.89
MPZ 2019/527	CVU_carb_3673	Barranco de la Cañada	C18c	5.90	1	3	*Quadratirhynchia* aff. *attenuata*	brachiopod	ventral	05/06/2018	25/06/2018	37.9	4.39	1.038	0.016	0.16	4.72	0.02	14/08/2018	99.1	2.82	-1.62
MPZ 2019/527	CVU_carb_3674	Barranco de la Cañada	C18c	5.90	1	3	*Quadratirhynchia* aff. *attenuata*	brachiopod	dorsal	05/06/2018	25/06/2018	38.1	4.85	1.033	0.017	0.16	4.97	0.00	14/08/2018	98.0	2.62	-2.42
MPZ 2019/527	CVU_carb_3675	Barranco de la Cañada	C18c	5.90	1	3	*Quadratirhynchia* aff. *attenuata*	brachiopod	dorsal	05/06/2018	25/06/2018	38.1	4.64	1.038	0.012	0.11	5.10	-0.01	14/08/2018	99.1	2.96	-2.04
MPZ 2019/528	CVU_carb_3960	Barranco de la Cañada	C18c	5.90	2	1	*Gryphaea (B.) sublobata*	bivalve		19/07/2018	02/08/2018	38.5	3.57	0.701	0.050	0.75	4.23	0.14	17/08/2018	100.6	3.51	-1.76
MPZ 2019/528	CVU_carb_3961	Barranco de la Cañada	C18c	5.90	2	1	*Gryphaea (B.) sublobata*	bivalve		19/07/2018	02/08/2018	38.8	2.51	0.750	0.040	0.63	3.41	0.12	17/08/2018	100.0	3.31	-2.12
MPZ 2019/528	CVU_carb_3962	Barranco de la Cañada	C18c	5.90	2	1	*Gryphaea (B.) sublobata*	bivalve		19/07/2018	02/08/2018	38.6	2.70	0.780	0.035	0.54	2.65	-0.05	17/08/2018	100.4	3.78	-1.70
MPZ 2019/529	CVU_carb_3963	Barranco de la Cañada	C18c	5.90	2	2	*Gryphaea (B.) sublobata*	bivalve		19/07/2018	02/08/2018	38.7	2.67	0.793	0.015	0.34	0.91	0.04	17/08/2018	101.4	3.84	-1.51
MPZ 2019/529	CVU_carb_3964	Barranco de la Cañada	C18c	5.90	2	2	*Gryphaea (B.) sublobata*	bivalve		19/07/2018	02/08/2018	38.6	2.89	0.759	0.014	0.24	1.63	0.07	17/08/2018	100.7	3.75	-1.55
MPZ 2019/529	CVU_carb_3965	Barranco de la Cañada	C18c	5.90	2	2	*Gryphaea (B.) sublobata*	bivalve		19/07/2018	02/08/2018	38.8	3.16	0.756	0.016	0.31	1.85	0.02	17/08/2018	101.0	3.90	-1.59
MPZ 2019/530	CVU_carb_3966	Barranco de la Cañada	C18c	5.90	2	3	*Gryphaea (B.) sublobata*	bivalve		19/07/2018	02/08/2018	38.7	2.19	0.784	0.008	0.16	0.94	-0.04	17/08/2018	101.1	3.90	-1.42
MPZ 2019/530	CVU_carb_3967	Barranco de la Cañada	C18c	5.90	2	3	*Gryphaea (B.) sublobata*	bivalve		19/07/2018	02/08/2018	38.9	2.25	0.793	0.009	0.16	0.99	0.08	17/08/2018	101.2	3.98	-1.24
MPZ 2019/530	CVU_carb_3968	Barranco de la Cañada	C18c	5.90	2	3	*Gryphaea (B.) sublobata*	bivalve		19/07/2018	02/08/2018	38.9	2.05	0.811	0.009	0.19	0.98	0.07	17/08/2018	100.7	3.97	-1.59
MPZ 2019/530	CVU_carb_3969	Barranco de la Cañada	C18c	5.90	2	3		bulk rock	bulk rock	19/07/2018	02/08/2018	25.4	74.96	0.472	0.633	32.97	1.94	0.60	17/08/2018	69.2	1.30	-3.71
MPZ 2019/524	CVU_carb_3571	Barranco de la Cañada	C18b	5.60	3	1	*Quadratirhynchia attenuata*	brachiopod	ventral	28/05/2018	08/06/2018	38.5	2.52	1.027	0.013	0.10	3.51	0.05	12/08/2018	100.4	3.34	-1.69
MPZ 2019/524	CVU_carb_3572	Barranco de la Cañada	C18b	5.60	3	1	*Quadratirhynchia attenuata*	brachiopod	ventral	28/05/2018	08/06/2018	38.4	2.58	1.027	0.014	0.11	3.51	0.05	12/08/2018	100.4	3.39	-1.66
MPZ 2019/524	CVU_carb_3573	Barranco de la Cañada	C18b	5.60	3	1	*Quadratirhynchia attenuata*	brachiopod	ventral	28/05/2018	08/06/2018	38.5	2.48	1.030	0.017	0.14	3.61	0.09	12/08/2018	98.2	3.60	-1.55
MPZ 2019/524	CVU_carb_3574	Barranco de la Cañada	C18b	5.60	3	1		bulk rock	bulk rock	28/05/2018	08/06/2018	26.7	77.25	0.457	0.576	31.81	2.78	0.49	12/08/2018	71.1	1.58	-3.53
MPZ 2019/520	CVU_carb_3939	Barranco de la Cañada	C18b	5.60	2	1	*Gryphaea (B.) sublobata*	bivalve		09/07/2018	02/08/2018	38.9	2.04	0.758	0.037	0.59	3.12	-0.02	17/08/2018	99.7	3.30	-2.05
MPZ 2019/520	CVU_carb_3940	Barranco de la Cañada	C18b	5.60	2	1	*Gryphaea (B.) sublobata*	bivalve		09/07/2018	02/08/2018	38.4	2.40	0.811	0.048	0.92	3.65	0.07	17/08/2018	99.9	3.41	-2.24
MPZ 2019/520	CVU_carb_3941	Barranco de la Cañada	C18b	5.60	2	1	*Gryphaea (B.) sublobata*	bivalve		09/07/2018	02/08/2018	38.8	2.08	0.815	0.030	0.50	1.89	-0.01	17/08/2018	91.8	3.64	-1.98
MPZ 2019/521	CVU_carb_3942	Barranco de la Cañada	C18b	5.60	2	2	*Gryphaea (B.) sublobata*	bivalve		09/07/2018	02/08/2018	39.1	2.41	0.604	0.071	0.70	3.70	0.03	17/08/2018	100.8	3.34	-2.07
MPZ 2019/521	CVU_carb_3943	Barranco de la Cañada	C18b	5.60	2	2	*Gryphaea (B.) sublobata*	bivalve		09/07/2018	02/08/2018	38.7	3.01	0.791	0.034	0.61	2.37	-0.03	17/08/2018	101.9	3.89	-2.13
MPZ 2019/521	CVU_carb_3944	Barranco de la Cañada	C18b	5.60	2	2	*Gryphaea (B.) sublobata*	bivalve		09/07/2018	02/08/2018	39.1	2.09	0.653	0.037	0.45	1.81	0.07	17/08/2018	99.3	3.50	-2.05
MPZ 2019/521	CVU_carb_3945	Barranco de la Cañada	C18b	5.60	2	2	*Gryphaea (B.) sublobata*	bivalve		09/07/2018	02/08/2018	38.8	2.37	0.644	0.044	0.52	2.60	0.09	17/08/2018	101.0	3.60	-2.22
MPZ 2019/522	CVU_carb_3946	Barranco de la Cañada	C18b	5.60	2	3	*Gryphaea (B.) sublobata*	bivalve		09/07/2018	02/08/2018	38.8	3.29	0.745	0.016	0.33	2.29	-0.10	17/08/2018	100.9	3.85	-1.88
MPZ 2019/522	CVU_carb_3947	Barranco de la Cañada	C18b	5.60	2	3	*Gryphaea (B.) sublobata*	bivalve		09/07/2018	02/08/2018	38.4	5.52	0.867	0.031	0.82	1.95	0.10	17/08/2018	101.6	4.04	-1.98
MPZ 2019/523	CVU_carb_3948	Barranco de la Cañada	C18b	5.60	2	4	*Gryphaea (B.) sublobata*	bivalve		09/07/2018	02/08/2018	38.8	3.14	0.811	0.010	0.16	2.61	-0.05	17/08/2018	101.6	3.67	-1.84
MPZ 2019/519	CVU_carb_3950	Barranco de la Cañada	C18b	5.60	1	1	*Gryphaea (B.) sublobata*	bivalve		18/07/2018	02/08/2018	38.5	3.77	0.935	0.020	0.54	1.34	0.06	17/08/2018	100.2	3.83	-1.89
MPZ 2019/519	CVU_carb_3951	Barranco de la Cañada	C18b	5.60	1	1	*Gryphaea (B.) sublobata*	bivalve		18/07/2018	02/08/2018	38.5	3.17	0.886	0.024	0.60	1.50	0.09	17/08/2018	99.1	3.82	-1.76
MPZ 2019/519	CVU_carb_3952	Barranco de la Cañada	C18b	5.60	1	1	*Gryphaea (B.) sublobata*	bivalve		18/07/2018	02/08/2018	38.7	2.79	0.982	0.018	0.44	2.45	0.05	17/08/2018	100.2	3.97	-1.85
MPZ 2019/519	CVU_carb_3953	Barranco de la Cañada	C18b	5.60	1	1	*Gryphaea (B.) sublobata*	bivalve		18/07/2018	02/08/2018	38.5	2.87	1.058	0.015	0.36	2.26	-0.04	17/08/2018	100.6	3.84	-1.97
MPZ 2019/516	CVU_carb_3852	Barranco de la Cañada	C18a	5.20	1	1	*Gryphaea (B.) sublobata*	bivalve		29/06/2018	06/07/2018	38.2	3.37	0.736	0.029	0.70	1.90	0.04	16/08/2018	99.9	3.43	-2.01
MPZ 2019/516	CVU_carb_3853	Barranco de la Cañada	C18a	5.20	1	1	*Gryphaea (B.) sublobata*	bivalve		29/06/2018	06/07/2018	38.3	4.09	0.833	0.040	0.84	1.92	0.09	16/08/2018	99.7	3.44	-2.18
MPZ 2019/516	CVU_carb_3854	Barranco de la Cañada	C18a	5.20	1	1		bulk rock	bulk rock	29/06/2018	06/07/2018	27.1	64.06	0.400	0.534	28.36	2.16	0.42	16/08/2018	74.8	1.40	-3.61
MPZ 2019/517	CVU_carb_3855	Barranco de la Cañada	C18a	5.20	1	2	*Gryphaea (B.) sublobata*	bivalve		29/06/2018	06/07/2018	37.0	6.52	0.727	0.065	1.92	4.61	0.12	16/08/2018	96.1	2.21	-1.91
MPZ 2019/517	CVU_carb_3856	Barranco de la Cañada	C18a	5.20	1	2	*Gryphaea (B.) sublobata*	bivalve		29/06/2018	06/07/2018	37.5	7.13	0.765	0.067	2.08	3.11	0.03	16/08/2018	99.0	2.47	-1.83
MPZ 2019/517	CVU_carb_3857	Barranco de la Cañada	C18a	5.20	1	2	*Gryphaea (B.) sublobata*	bivalve		29/06/2018	06/07/2018	37.4	10.40	0.685	0.117	4.21	3.33	0.30	16/08/2018	94.0	2.06	-1.77
MPZ 2019/518	CVU_carb_3858	Barranco de la Cañada	C18a	5.20	1	3	*Gryphaea (B.) sublobata*	bivalve		29/06/2018	06/07/2018	38.1	2.38	0.749	0.031	0.56	3.12	0.07	16/08/2018	99.3	3.58	-1.75
MPZ 2019/518	CVU_carb_3859	Barranco de la Cañada	C18a	5.20	1	3	*Gryphaea (B.) sublobata*	bivalve		29/06/2018	06/07/2018	38.5	2.42	0.801	0.020	0.38	2.84	0.09	16/08/2018	98.6	3.58	-1.86
MPZ 2019/518	CVU_carb_3860	Barranco de la Cañada	C18a	5.20	1	3	*Gryphaea (B.) sublobata*	bivalve		29/06/2018	06/07/2018	38.6	2.54	0.757	0.023	0.37	4.44	0.04	16/08/2018	97.9	3.83	-1.64
MPZ 2019/515	CVU_carb_3439	Barranco de la Cañada	C15b	4.60	2	1		bulk rock	bulk rock	10/04/2018	04/06/2018	28.1	40.08	0.350	0.459	15.59	1.70	0.23	10/08/2018	74.7	1.41	-3.76
MPZ 2019/514	CVU_carb_3873	Barranco de la Cañada	C15b	4.60	1	1	*Gryphaea (B.) sublobata*	bivalve		29/06/2018	06/07/2018	38.9	3.17	0.743	0.086	1.43	4.35	0.05	16/08/2018	100.1	3.04	-2.32
MPZ 2019/514	CVU_carb_3874	Barranco de la Cañada	C15b	4.60	1	1	*Gryphaea (B.) sublobata*	bivalve		29/06/2018	06/07/2018	38.7	4.35	0.791	0.020	0.32	4.03	0.04	16/08/2018	99.7	3.54	-1.77
MPZ 2019/514	CVU_carb_3875	Barranco de la Cañada	C15b	4.60	1	1	*Gryphaea (B.) sublobata*	bivalve		29/06/2018	06/07/2018	38.9	3.39	0.790	0.042	0.85	1.79	-0.05	16/08/2018	99.4	3.37	-1.87
MPZ 2019/515	CVU_carb_3954	Barranco de la Cañada	C15b	4.60	2	1	*Gryphaea (B.) sublobata*	bivalve		18/07/2018	02/08/2018	34.5	23.21	0.359	0.350	11.23	2.02	0.36	17/08/2018	91.0	1.85	-3.43
MPZ 2019/515	CVU_carb_3955	Barranco de la Cañada	C15b	4.60	2	1	*Gryphaea (B.) sublobata*	bivalve		18/07/2018	02/08/2018	35.0	17.67	0.453	0.263	8.23	4.00	0.32	17/08/2018	93.3	2.16	-2.99
MPZ 2019/515	CVU_carb_3956	Barranco de la Cañada	C15b	4.60	2	1	*Gryphaea (B.) sublobata*	bivalve		18/07/2018	02/08/2018	36.9	13.18	0.628	0.217	4.15	6.90	0.17	17/08/2018	95.0	2.85	-2.55
MPZ 2019/513	CVU_carb_3635	Barranco de la Cañada	C15a	4.00	4	1	*Quadratirhynchia attenuata*	brachiopod	ventral	04/06/2018	08/06/2018	38.3	5.51	1.004	0.045	0.63	4.23	0.18	13/08/2018	98.0	2.67	-2.04
MPZ 2019/513	CVU_carb_3636	Barranco de la Cañada	C15a	4.00	4	1	*Quadratirhynchia attenuata*	brachiopod	ventral	04/06/2018	08/06/2018	38.3	5.06	1.012	0.050	0.50	4.05	0.11	13/08/2018	98.1	2.82	-2.13
MPZ 2019/513	CVU_carb_3637	Barranco de la Cañada	C15a	4.00	4	1	*Quadratirhynchia attenuata*	brachiopod	ventral	04/06/2018	08/06/2018	38.3	4.12	0.995	0.028	0.32	4.00	0.15	13/08/2018	99.0	3.09	-1.92
MPZ 2019/513	CVU_carb_3638	Barranco de la Cañada	C15a	4.00	4	1	*Quadratirhynchia attenuata*	brachiopod	dorsal	04/06/2018	08/06/2018	38.2	5.39	1.065	0.027	0.26	4.50	0.16	13/08/2018	99.0	2.79	-1.98
MPZ 2019/513	CVU_carb_3639	Barranco de la Cañada	C15a	4.00	4	1	*Quadratirhynchia attenuata*	brachiopod	dorsal	04/06/2018	08/06/2018	38.5	4.79	1.011	0.030	0.31	4.36	0.14	13/08/2018	98.5	3.00	-1.80
MPZ 2019/513	CVU_carb_3640	Barranco de la Cañada	C15a	4.00	4	1	*Quadratirhynchia attenuata*	brachiopod	dorsal	04/06/2018	08/06/2018	38.4	4.59	0.998	0.027	0.29	4.24	0.21	13/08/2018	98.9	2.94	-1.63
MPZ 2019/512	CVU_carb_3701	Barranco de la Cañada	C15a	4.00	3	1	*Tetrarhynchia subconcinna*	brachiopod	dorsal, sediment cont.	08/06/2018	25/06/2018	38.4	4.70	0.970	0.015	0.27	4.42	0.00	14/08/2018	97.8	2.13	-2.35
MPZ 2019/512	CVU_carb_3702	Barranco de la Cañada	C15a	4.00	3	1	*Tetrarhynchia subconcinna*	brachiopod	dorsal, sediment cont.	08/06/2018	25/06/2018	38.1	4.82	0.995	0.022	0.20	4.32	0.08	14/08/2018	98.7	1.88	-2.65
MPZ 2019/510	CVU_carb_3901	Barranco de la Cañada	C15a	4.00	2	1	*Gryphaea (B.) sublobata*	bivalve		02/07/2018	06/07/2018	39.0	3.50	0.701	0.038	0.42	3.37	-0.13	16/08/2018	100.2	3.38	-2.16
MPZ 2019/510	CVU_carb_3902	Barranco de la Cañada	C15a	4.00	2	1	*Gryphaea (B.) sublobata*	bivalve		02/07/2018	06/07/2018	38.8	3.44	0.690	0.038	0.42	3.94	0.05	16/08/2018	99.9	3.24	-2.17
MPZ 2019/510	CVU_carb_3903	Barranco de la Cañada	C15a	4.00	2	1	*Gryphaea (B.) sublobata*	bivalve		02/07/2018	06/07/2018	38.9	3.00	0.711	0.012	0.10	2.07	0.03	16/08/2018	99.3	3.56	-2.41
MPZ 2019/506	CVU_carb_3904	Barranco de la Cañada	C15a	4.00	1	1	*Gryphaea (B.) sublobata*	bivalve		02/07/2018	06/07/2018	39.3	3.16	0.625	0.060	0.75	4.26	0.06	16/08/2018	100.5	2.62	-2.34
MPZ 2019/506	CVU_carb_3905	Barranco de la Cañada	C15a	4.00	1	1	*Gryphaea (B.) sublobata*	bivalve		03/07/2018	06/07/2018	39.2	3.64	0.749	0.030	0.48	6.10	-0.03	16/08/2018	99.2	2.85	-2.08
MPZ 2019/506	CVU_carb_3906	Barranco de la Cañada	C15a	4.00	1	1	*Gryphaea (B.) sublobata*	bivalve		03/07/2018	06/07/2018	38.7	4.04	0.737	0.019	0.27	7.34	0.10	16/08/2018	100.4	2.73	-2.20
MPZ 2019/507	CVU_carb_3907	Barranco de la Cañada	C15a	4.00	1	2	*Gryphaea (B.) sublobata*	bivalve		03/07/2018	06/07/2018	38.7	2.67	0.704	0.040	0.66	3.18	0.06	16/08/2018	100.9	2.99	-1.92
MPZ 2019/507	CVU_carb_3908	Barranco de la Cañada	C15a	4.00	1	2	*Gryphaea (B.) sublobata*	bivalve		03/07/2018	06/07/2018	38.9	2.06	0.654	0.063	0.98	2.08	0.01	17/08/2018	93.2	3.36	-1.95
MPZ 2019/507	CVU_carb_3909	Barranco de la Cañada	C15a	4.00	1	2	*Gryphaea (B.) sublobata*	bivalve		03/07/2018	06/07/2018	38.9	1.90	0.723	0.014	0.18	1.77	0.07	17/08/2018	99.5	3.44	-1.73
MPZ 2019/508	CVU_carb_3910	Barranco de la Cañada	C15a	4.00	1	3	*Gryphaea (B.) sublobata*	bivalve		03/07/2018	06/07/2018	39.2	3.01	0.659	0.050	0.63	4.60	-0.04	17/08/2018	100.0	3.18	-2.27
MPZ 2019/508	CVU_carb_3911	Barranco de la Cañada	C15a	4.00	1	3	*Gryphaea (B.) sublobata*	bivalve		03/07/2018	06/07/2018	39.2	2.59	0.648	0.043	0.51	4.39	-0.09	17/08/2018	99.8	2.97	-2.44
MPZ 2019/508	CVU_carb_3912	Barranco de la Cañada	C15a	4.00	1	3	*Gryphaea (B.) sublobata*	bivalve		03/07/2018	02/08/2018	38.6	2.57	0.633	0.057	0.84	4.99	0.07	17/08/2018	100.2	3.16	-2.64
MPZ 2019/509	CVU_carb_3913	Barranco de la Cañada	C15a	4.00	1	4	*Gryphaea (B.) sublobata*	bivalve		03/07/2018	02/08/2018	38.5	2.34	0.671	0.054	0.80	6.28	0.09	17/08/2018	100.2	3.18	-2.54
MPZ 2019/509	CVU_carb_3914	Barranco de la Cañada	C15a	4.00	1	4	*Gryphaea (B.) sublobata*	bivalve		03/07/2018	02/08/2018	38.6	2.51	0.665	0.061	0.80	4.67	0.14	17/08/2018	100.2	3.14	-2.49
MPZ 2019/509	CVU_carb_3915	Barranco de la Cañada	C15a	4.00	1	4	*Gryphaea (B.) sublobata*	bivalve		03/07/2018	02/08/2018	38.1	3.74	0.657	0.099	1.47	4.59	0.06	17/08/2018	100.0	2.51	-3.30
MPZ 2019/509	CVU_carb_3916	Barranco de la Cañada	C15a	4.00	1	4		bulk rock	bulk rock	03/07/2018	02/08/2018	28.2	53.66	0.372	0.533	26.68	2.52	0.71	17/08/2018	75.9	1.46	-3.60
MPZ 2019/504	CVU_carb_3882	Barranco de la Cañada	C12b	3.40	1	1	*Gryphaea (B.) sublobata*	bivalve		02/07/2018	06/07/2018	39.1	2.85	0.776	0.029	0.40	5.17	0.12	16/08/2018	100.0	2.82	-2.16

MPZ 2019/504	CVU_carb_3883	Barranco de la Cañada	C12b	3.40	1	1	*Gryphaea* (*B.*) *sublobata*	bivalve		02/07/2018	06/07/2018	39.2	2.83	0.792	0.015	0.26	3.51	-0.07	16/08/2018	101.0	3.28	-1.86
MPZ 2019/504	CVU_carb_3884	Barranco de la Cañada	C12b	3.40	1	1	*Gryphaea* (*B.*) *sublobata*	bivalve		02/07/2018	06/07/2018	38.7	2.15	0.772	0.020	0.22	4.89	-0.07	16/08/2018	97.8	3.19	-1.69
MPZ 2019/505	CVU_carb_3885	Barranco de la Cañada	C12b	3.40	1	2	*Gryphaea* (*B.*) *sublobata*	bivalve		02/07/2018	06/07/2018	38.8	3.14	0.650	0.025	0.39	4.55	0.02	16/08/2018	99.6	1.91	-2.22
MPZ 2019/505	CVU_carb_3886	Barranco de la Cañada	C12b	3.40	1	2	*Gryphaea* (*B.*) *sublobata*	bivalve		02/07/2018	06/07/2018	38.6	3.56	0.656	0.039	0.51	5.82	-0.01	16/08/2018	100.4	1.82	-2.45
MPZ 2019/505	CVU_carb_3887	Barranco de la Cañada	C12b	3.40	1	2	*Gryphaea* (*B.*) *sublobata*	bivalve		02/07/2018	06/07/2018	38.6	3.09	0.645	0.031	0.36	4.57	0.06	16/08/2018	98.6	1.91	-2.36
MPZ 2019/505	CVU_carb_3888	Barranco de la Cañada	C12b	3.40	1	2		bulk rock	bulk rock	02/07/2018	06/07/2018	30.4	32.00	0.263	0.429	18.22	1.56	1.51	16/08/2018	79.8	0.72	-2.94
MPZ 2019/498	CVU_carb_3432	Barranco de la Cañada	C12a	2.80	2	1	*Gryphaea* (*B.*) *sublobata*	bivalve		10/04/2018	04/06/2018	38.7	2.49	0.673	0.086	1.02	4.34	-0.04	10/08/2018	100.4	1.74	-2.23
MPZ 2019/502	CVU_carb_3502	Barranco de la Cañada	C12a	2.80	3	1	*Quadratirhynchia attenuata*	brachiopod	ventral	02/05/2018	04/06/2018	38.3	5.21	1.016	0.034	0.31	4.85	0.02	10/08/2018	98.6	1.48	-2.32
MPZ 2019/502	CVU_carb_3503	Barranco de la Cañada	C12a	2.80	3	1	*Quadratirhynchia attenuata*	brachiopod	ventral	02/05/2018	04/06/2018	38.2	5.53	1.048	0.025	0.22	4.72	0.03	10/08/2018	99.7	1.58	-2.17
MPZ 2019/502	CVU_carb_3504	Barranco de la Cañada	C12a	2.80	3	1	*Quadratirhynchia attenuata*	brachiopod	ventral	02/05/2018	04/06/2018	38.4	5.25	1.077	0.014	0.13	4.50	0.03	10/08/2018	98.6	1.51	-2.12
MPZ 2019/502	CVU_carb_3505	Barranco de la Cañada	C12a	2.80	3	1		calcite cement	calcite cement	02/05/2018	04/06/2018	31.3	32.17	0.324	0.390	15.05	1.66	0.83	10/08/2018	82.3	0.57	-3.33
MPZ 2019/503	CVU_carb_3506	Barranco de la Cañada	C12a	2.80	3	2	*Quadratirhynchia attenuata*	brachiopod	ventral	02/05/2018	04/06/2018	38.4	4.96	0.917	0.044	0.29	3.67	0.07	10/08/2018	98.9	1.54	-2.39
MPZ 2019/503	CVU_carb_3507	Barranco de la Cañada	C12a	2.80	3	2	*Quadratirhynchia attenuata*	brachiopod	ventral	02/05/2018	04/06/2018	38.9	4.15	0.822	0.045	0.39	3.86	0.04	10/08/2018	100.4	1.45	-2.48
MPZ 2019/503	CVU_carb_3508	Barranco de la Cañada	C12a	2.80	3	2	*Quadratirhynchia attenuata*	brachiopod	ventral	02/05/2018	04/06/2018	38.4	5.49	0.862	0.058	0.42	3.45	0.09	12/08/2018	98.2	1.43	-2.64
MPZ 2019/503	CVU_carb_3509	Barranco de la Cañada	C12a	2.80	3	2	*Quadratirhynchia attenuata*	brachiopod	dorsal	02/05/2018	04/06/2018	38.5	4.45	0.913	0.018	0.13	3.99	0.03	12/08/2018	97.5	1.53	-2.04
MPZ 2019/503	CVU_carb_3510	Barranco de la Cañada	C12a	2.80	3	2	*Quadratirhynchia attenuata*	brachiopod	dorsal	02/05/2018	04/06/2018	38.8	4.53	0.889	0.023	0.17	3.70	0.06	12/08/2018	99.2	1.27	-2.25
MPZ 2019/503	CVU_carb_3511	Barranco de la Cañada	C12a	2.80	3	2	*Quadratirhynchia attenuata*	brachiopod	dorsal	02/05/2018	04/06/2018	38.3	5.17	0.883	0.024	0.22	4.32	0.03	12/08/2018	98.4	1.30	-2.06
MPZ 2019/498	CVU_carb_3861	Barranco de la Cañada	C12a	2.80	2	1a	*Gryphaea* (*B.*) *sublobata*	bivalve		29/06/2018	06/07/2018	38.4	3.49	0.698	0.085	1.17	3.32	0.09	16/08/2018	99.6	2.08	-2.24
MPZ 2019/499	CVU_carb_3862	Barranco de la Cañada	C12a	2.80	2	1b	*Gryphaea* (*B.*) *sublobata*	bivalve		29/06/2018	06/07/2018	38.7	3.22	0.787	0.026	0.36	6.43	-0.04	16/08/2018	99.2	1.82	-2.23
MPZ 2019/499	CVU_carb_3863	Barranco de la Cañada	C12a	2.80	2	1b	*Gryphaea* (*B.*) *sublobata*	bivalve		29/06/2018	06/07/2018	38.5	2.47	0.808	0.035	0.56	3.11	0.05	16/08/2018	99.1	2.29	-2.02
MPZ 2019/500	CVU_carb_3864	Barranco de la Cañada	C12a	2.80	2	2	*Gryphaea* (*B.*) *sublobata*	bivalve		29/06/2018	06/07/2018	38.9	2.87	0.648	0.010	0.18	2.22	-0.06	16/08/2018	100.3	2.36	-1.60
MPZ 2019/500	CVU_carb_3865	Barranco de la Cañada	C12a	2.80	2	2	*Gryphaea* (*B.*) *sublobata*	bivalve		29/06/2018	06/07/2018	38.2	3.52	0.689	0.019	0.58	1.50	-0.05	16/08/2018	99.6	2.69	-1.30
MPZ 2019/500	CVU_carb_3866	Barranco de la Cañada	C12a	2.80	2	2	*Gryphaea* (*B.*) *sublobata*	bivalve		29/06/2018	06/07/2018	38.6	2.75	0.639	0.027	1.10	1.50	0.00	16/08/2018	97.8	2.58	-1.46
MPZ 2019/501	CVU_carb_3867	Barranco de la Cañada	C12a	2.80	2	3	*Gryphaea* (*B.*) *sublobata*	bivalve		29/06/2018	06/07/2018	38.8	1.54	0.815	0.023	0.34	2.92	0.01	16/08/2018	100.1	2.32	-1.85
MPZ 2019/501	CVU_carb_3868	Barranco de la Cañada	C12a	2.80	2	3	*Gryphaea* (*B.*) *sublobata*	bivalve		29/06/2018	06/07/2018	39.2	1.86	0.647	0.030	0.35	2.33	0.01	16/08/2018	99.9	2.25	-1.90
MPZ 2019/501	CVU_carb_3869	Barranco de la Cañada	C12a	2.80	2	3	*Gryphaea* (*B.*) *sublobata*	bivalve		29/06/2018	06/07/2018	38.6	1.79	0.860	0.026	0.39	2.90	0.04	16/08/2018	96.9	2.20	-1.81
MPZ 2019/493	CVU_carb_3917	Barranco de la Cañada	C07b	1.60	1	1		bulk rock	bulk rock	06/07/2018	02/08/2018	29.1	33.83	0.272	0.454	21.80	1.36	0.68	17/08/2018	77.4	0.05	-2.96
MPZ 2019/494	CVU_carb_3918	Barranco de la Cañada	C07b	1.60	1	2	*Gryphaea* (*B.*) *sublobata*	bivalve		06/07/2018	02/08/2018	38.6	2.79	0.604	0.082	1.06	4.77	0.09	17/08/2018	100.0	0.72	-2.30
MPZ 2019/494	CVU_carb_3919	Barranco de la Cañada	C07b	1.60	1	2	*Gryphaea* (*B.*) *sublobata*	bivalve		06/07/2018	02/08/2018	38.3	2.44	0.735	0.024	0.39	2.46	0.12	17/08/2018	100.7	1.07	-1.76
MPZ 2019/494	CVU_carb_3920	Barranco de la Cañada	C07b	1.60	1	2	*Gryphaea* (*B.*) *sublobata*	bivalve		06/07/2018	02/08/2018	38.6	2.28	0.680	0.022	0.37	3.20	0.12	17/08/2018	100.7	0.83	-2.24
MPZ 2019/491	CVU_carb_3841	Barranco de la Cañada	C07a	0.80	1	1	*Gryphaea* (*B.*) *sublobata*	bivalve		29/06/2018	06/07/2018	37.9	6.55	0.637	0.031	0.86	3.17	0.12	16/08/2018	97.0	0.55	-1.84
MPZ 2019/491	CVU_carb_3842	Barranco de la Cañada	C07a	0.80	1	1	*Gryphaea* (*B.*) *sublobata*	bivalve		29/06/2018	06/07/2018	38.2	4.77	0.640	0.019	0.65	3.18	0.01	16/08/2018	98.8	0.64	-1.64
MPZ 2019/491	CVU_carb_3843	Barranco de la Cañada	C07a	0.80	1	1	*Gryphaea* (*B.*) *sublobata*	bivalve		29/06/2018	06/07/2018	38.5	4.14	0.668	0.013	0.30	3.51	0.18	16/08/2018	98.3	0.74	-1.52
MPZ 2019/492	CVU_carb_3844	Barranco de la Cañada	C07a	0.80	1	2	*Gryphaea* (*B.*) *sublobata*	bivalve		29/06/2018	06/07/2018	39.1	3.07	0.638	0.049	0.61	10.61	0.04	16/08/2018	98.1	1.34	-2.48
MPZ 2019/492	CVU_carb_3845	Barranco de la Cañada	C07a	0.80	1	2	*Gryphaea* (*B.*) *sublobata*	bivalve		29/06/2018	06/07/2018	38.7	2.56	0.665	0.022	0.29	4.99	0.14	16/08/2018	99.3	1.40	-2.25
MPZ 2019/492	CVU_carb_3846	Barranco de la Cañada	C07a	0.80	1	2	*Gryphaea* (*B.*) *sublobata*	bivalve		29/06/2018	06/07/2018	39.1	2.49	0.681	0.018	0.21	5.01	0.17	16/08/2018	99.8	1.30	-2.26
MPZ 2019/492	CVU_carb_3847	Barranco de la Cañada	C07a	0.80	1	2		belemnite		29/06/2018	06/07/2018	36.5	9.41	1.493	0.007	0.10	1.76	0.62	16/08/2018	98.1	0.04	-0.59
MPZ 2019/492	CVU_carb_3848	Barranco de la Cañada	C07a	0.80	1	2		belemnite		29/06/2018	06/07/2018	38.7	8.78	1.526	0.007	0.23	3.22	0.65	16/08/2018	98.8	0.10	-0.30
MPZ 2019/492	CVU_carb_3849	Barranco de la Cañada	C07a	0.80	1	2		belemnite		29/06/2018	06/07/2018	38.1	11.52	1.817	0.008	0.07	5.21	0.89	16/08/2018	96.6	0.41	-0.49
MPZ 2019/492	CVU_carb_3850	Barranco de la Cañada	C07a	0.80	1	2		belemnite	apical line	29/06/2018	06/07/2018	37.9	13.21	1.759	0.069	1.15	7.08	1.08	16/08/2018	97.9	-0.03	-1.53
MPZ 2019/492	CVU_carb_3851	Barranco de la Cañada	C07a	0.80	1	2		bulk rock	bulk rock	29/06/2018	06/07/2018	32.7	26.16	0.343	0.345	16.45	5.33	0.42	16/08/2018	83.9	0.08	-3.32
MPZ 2019/485	CVU_carb_3434	Barranco de la Cañada	C05	0.40	2	1	*Gryphaea* (*B.*) *sublobata*	bivalve		10/04/2018	04/06/2018	38.7	1.83	0.622	0.025	0.40	7.31	0.21	10/08/2018	99.4	1.18	-1.59
MPZ 2019/487	CVU_carb_3792	Barranco de la Cañada	C05	0.40	3	1	*Gibbirhynchia* cf. *cantabrica*	brachiopod	ventral	28/06/2018	06/07/2018	37.7	5.81	1.116	0.020	0.15	8.28	0.06	15/08/2018	97.3	0.19	-2.28
MPZ 2019/487	CVU_carb_3793	Barranco de la Cañada	C05	0.40	3	1	*Gibbirhynchia* cf. *cantabrica*	brachiopod	ventral	28/06/2018	06/07/2018	37.8	4.86	1.139	0.014	0.07	9.10	0.07	15/08/2018	96.8	0.72	-1.81
MPZ 2019/487	CVU_carb_3794	Barranco de la Cañada	C05	0.40	3	1	*Gibbirhynchia* cf. *cantabrica*	brachiopod	ventral	28/06/2018	06/07/2018	38.0	4.70	1.152	0.011	0.07	9.03	0.16	15/08/2018	96.9	0.81	-1.84
MPZ 2019/487	CVU_carb_3795	Barranco de la Cañada	C05	0.40	3	1	*Gibbirhynchia* cf. *cantabrica*	brachiopod	dorsal	28/06/2018	06/07/2018	37.7	4.98	1.156	0.011	0.03	8.92	-0.02	15/08/2018	96.4	0.70	-1.51
MPZ 2019/488	CVU_carb_3796	Barranco de la Cañada	C05	0.40	3	2	*Gibbirhynchia* cf. *cantabrica*	brachiopod	ventral	28/06/2018	06/07/2018	37.5	7.35	1.219	0.020	0.12	9.21	0.10	15/08/2018	96.9	-0.02	-2.25
MPZ 2019/488	CVU_carb_3797	Barranco de la Cañada	C05	0.40	3	2	*Gibbirhynchia* cf. *cantabrica*	brachiopod	ventral	28/06/2018	06/07/2018	37.8	6.50	1.174	0.020	0.09	8.85	0.08	15/08/2018	95.2	0.01	-2.22
MPZ 2019/488	CVU_carb_3798	Barranco de la Cañada	C05	0.40	3	2	*Gibbirhynchia* cf. *cantabrica*	brachiopod	dorsal	28/06/2018	06/07/2018	37.6	5.99	1.240	0.016	0.07	8.86	0.00	15/08/2018	95.0	0.30	-1.99
MPZ 2019/489	CVU_carb_3799	Barranco de la Cañada	C05	0.40	3	3	*Gibbirhynchia* cf. *cantabrica*	brachiopod	ventral	28/06/2018	06/07/2018	37.9	3.96	1.078	0.022	0.10	7.80	0.11	15/08/2018	97.4	0.52	-2.56
MPZ 2019/490	CVU_carb_3800	Barranco de la Cañada	C05	0.40	3	4		bulk rock	bulk rock	28/06/2018	06/07/2018	26.3	54.31	0.417	0.374	23.98	1.91	0.58	15/08/2018	69.9	0.01	-2.84
MPZ 2019/485	CVU_carb_3895	Barranco de la Cañada	C05	0.40	2	1	*Gryphaea* (*B.*) *sublobata*	bivalve		02/07/2018	06/07/2018	38.9	2.63	0.674	0.026	0.38	3.54	-0.10	16/08/2018	99.6	1.24	-1.68
MPZ 2019/485	CVU_carb_3896	Barranco de la Cañada	C05	0.40	2	1	*Gryphaea* (*B.*) *sublobata*	bivalve		02/07/2018	06/07/2018	38.8	2.72	0.761	0.015	0.29	1.13	0.16	16/08/2018	100.3	1.39	-1.73
MPZ 2019/485	CVU_carb_3897	Barranco de la Cañada	C05	0.40	2	1	*Gryphaea* (*B.*) *sublobata*	bivalve		02/07/2018	06/07/2018	38.9	3.27	0.704	0.038	0.63	1.96	0.06	16/08/2018	100.5	1.35	-1.75
MPZ 2019/486	CVU_carb_3898	Barranco de la Cañada	C05	0.40	2	2	*Gryphaea* (*B.*) *sublobata*	bivalve		02/07/2018	06/07/2018	39.1	2.11	0.640	0.025	0.35	4.11	0.01	16/08/2018	100.0	0.76	-1.88
MPZ 2019/486	CVU_carb_3899	Barranco de la Cañada	C05	0.40	2	2	*Gryphaea* (*B.*) *sublobata*	bivalve		02/07/2018	06/07/2018	38.8	2.16	0.643	0.027	0.36	2.33	-0.04	16/08/2018	100.2	0.77	-1.79
MPZ 2019/486	CVU_carb_3900	Barranco de la Cañada	C05	0.40	2	2	*Gryphaea* (*B.*) *sublobata*	bivalve		02/07/2018	06/07/2018	39.1	2.29	0.651	0.025	0.27	3.87	-0.02	16/08/2018	100.2	0.81	-1.64
MPZ 2019/484	CVU_carb_3957	Barranco de la Cañada	C05	0.40	1	1	*Gryphaea* (*B.*) *sublobata*	bivalve		18/07/2018	02/08/2018	37.4	10.67	0.601	0.216	3.28	5.29	0.53	17/08/2018	97.8	-0.15	-1.93
MPZ 2019/484	CVU_carb_3958	Barranco de la Cañada	C05	0.40	1	1	*Gryphaea* (*B.*) *sublobata*	bivalve		18/07/2018	02/08/2018	38.4	8.08	0.635	0.146	1.11	4.39	0.33	17/08/2018	99.6	0.15	-1.65
MPZ 2019/484	CVU_carb_3959	Barranco de la Cañada	C05	0.40	1	1	*Gryphaea* (*B.*) *sublobata*	bivalve		18/07/2018	02/08/2018	37.1	8.98	0.629	0.122	3.77	4.42	0.40	17/08/2018	95.7	0.34	-1.60
MB.B.10910	CVU_carb_4870	Fonte Coberta / Rabaçal	R24b	28.35	1	1	*Telothyris jauberti*	brachiopod	dorsal	07/04/2019	24/04/2019	96.9	6.97	0.612	0.020	0.101	1.50	0.11	18/04/2019	99.8	5.49	-1.70
MB.B.10910	CVU_carb_4871	Fonte Coberta / Rabaçal	R24b	28.35	1	1	*Telothyris jauberti*	brachiopod	dorsal	07/04/2019	24/04/2019	96.3	8.22	0.731	0.043	0.131	3.20	0.24	18/04/2019	100.0	5.27	-1.84
MB.B.10910	CVU_carb_4872	Fonte Coberta / Rabaçal	R24b	28.35	1	1	*Telothyris jauberti*	brachiopod	dorsal	07/04/2019	24/04/2019	96.8	7.06	0.538	0.010	0.049	1.96	0.16	18/04/2019	100.4	5.40	-1.80
MB.B.10912	CVU_carb_4903	Fonte Coberta / Rabaçal	R24b	28.35	2	1	*Lobothyris hispanica*	brachiopod	dorsal	09/04/2019	24/04/2019	96.4	7.39	0.562	0.029	0.067	1.76	0.10	21/04/2019	101.0	5.75	-2.18
MB.B.10912	CVU_carb_4904	Fonte Coberta / Rabaçal	R24b	28.35	2	1	*Lobothyris hispanica*	brachiopod	dorsal	09/04/2019	24/04/2019	96.5	5.84	0.595	0.031	0.052	1.78	0.10	21/04/2019	100.5	5.59	-1.92
MB.B.10912	CVU_carb_4905	Fonte Coberta / Rabaçal	R24b	28.35	2	1	*Lobothyris hispanica*	brachiopod	dorsal	09/04/2019	24/04/2019	96.8	5.46	0.491	0.013	0.038	1.49	0.15	21/04/2019	100.3	5.53	-2.05
MB.B.10910	CVU_carb_4944	Fonte Coberta / Rabaçal	R24b	28.35	1	1		bulk rock		14/04/2019	24/04/2019	84.3	25.98	0.440	0.470	10.760	1.46	0.33	21/04/2019	88.5	4.32	-3.81
MB.B.10909	CVU_carb_4815	Fonte Coberta / Rabaçal	R23c	26.60	3	1	*Homoeorhynchia meridionalis*	brachiopod	dorsal	25/03/2019	17/04/2019	96.6	4.72	0.945	0.029	0.209	2.28	0.18	30/03/2019	99.6	4.37	-1.97
MB.B.10909	CVU_carb_4816	Fonte Coberta / Rabaçal	R23c	26.60	3	1	*Homoeorhynchia meridionalis*	brachiopod	dorsal	25/03/2019	17/04/2019	96.1	6.34	0.932	0.034	0.307	2.53	0.19	30/03/2019	99.1	4.46	-1.89
MB.B.10909	CVU_carb_4817	Fonte Coberta / Rabaçal	R23c	26.60	3	1	*Homoeorhynchia meridionalis*	brachiopod	ventral	25/03/2019	17/04/2019	96.4	5.47	0.978	0.021	0.139	2.20	0.14	30/03/2019	98.3	4.51	-1.90
MB.B.10909	CVU_carb_4818	Fonte Coberta / Rabaçal	R23c	26.60	3	1	*Homoeorhynchia meridionalis*	brachiopod	ventral	25/03/2019	17/04/2019	96.1	5.45	0.937	0.033	0.320	2.41	0.27	30/03/2019	99.3	4.36	-1.76
MB.B.10905	CVU_carb_4947	Fonte Coberta / Rabaçal	R23c	26.60	1	1		bulk rock		14/04/2019	24/04/2019	81.5	21.83	0.366	0.402	18.161	6.40	0.34	21/04/2019	86.1	3.89	-4.32
MB.B.20346	CVU_carb_4931	Fonte Coberta / Rabaçal	R23a	25.05	2	1		bulk rock		14/04/2019	24/04/2019	68.4	35.17	0.468	0.492	22.040	2.82	0.84	21/04/2019	71.6	3.77	-4.24
MB.B.10902	CVU_carb_4935	Fonte Coberta / Rabaçal	R21a	22.25	2	1		bulk rock		14/04/2019	24/04/2019	71.3	31.61	0.444	0.464	18.320	2.31	0.84	21/04/2019	75.0	3.85	-4.14
MB.B.10898	CVU_carb_4876	Fonte Coberta / Rabaçal	FC19c	21.40	1	1	*Soaresirhynchia bouchardi*	brachiopod	ventral	07/04/2019	24/04/2019	96.4	3.10	0.516	0.020	0.209	1.79	0.16	18/04/2019	99.3	5.71	-1.68
MB.B.10898	CVU_carb_4932	Fonte Coberta / Rabaçal	FC19c	21.40	1	1		bulk rock		14/04/2019	24/04/2019	68.5	32.95	0.506	0.526	19.785	2.59	1.00	21/04/2019	71.7	3.82	-4.18
MB.B.10897	CVU_carb_4873	Fonte Coberta / Rabaçal	FC17d	16.80	1	1	*Soaresirhynchia bouchardi*	brachiopod		07/04/2019	24/04/2019	97.2	1.83	0.517	0.006	0.045	1.77	0.18	18/04/2019	99.5	3.77	-2.39
MB.B.10897	CVU_carb_4874	Fonte Coberta / Rabaçal	FC17d	16.80	1	1	*Soaresirhynchia bouchardi*	brachiopod		07/04/2019	24/04/2019	96.6	2.52	0.502	0.007	0.056	1.92	0.18	18/04/2019	100.6	3.93	-2.16
MB.B.10897	CVU_carb_4875	Fonte Coberta / Rabaçal	FC17d	16.80	1	1	*Soaresirhynchia bouchardi*	brachiopod		07/04/2019	24/04/2019	97.1	2.50	0.537	0.007	0.044	1.96	0.11	18/04/2019	99.7	3.84	-2.22
MB.B.10897	CVU_carb_4942	Fonte Coberta / Rabaçal	FC17d	16.80	1	1		bulk rock		14/04/2019	24/04/2019	73.5	31.10	0.611	0.465	17.080	2.92	0.67	21/04/2019	76.4	2.62	-4.36
MB.B.10894	CVU_carb_4856	Fonte Coberta / Rabaçal	FC17a	15.40	2	1	*Soaresirhynchia bouchardi*	brachiopod	ventral	06/04/2019	17/04/2019	97.3	3.35	0.502	0.028	0.185	1.75	0.11	18/04/2019	100.4	3.06	-2.22
MB.B.10894	CVU_carb_4857	Fonte Coberta / Rabaçal	FC17a	15.40	2	1	*Soaresirhynchia bouchardi*	brachiopod	ventral	06/04/2019	17/04/2019	97.6	3.64	0.498	0.019	0.153	1.45	0.21	18/04/2019	100.0	3.10	-2.28
MB.B.10894	CVU_carb_4858	Fonte Coberta / Rabaçal	FC17a	15.40	2	1	*Soaresirhynchia bouchardi*	brachiopod	ventral	06/04/2019	17/04/2019	97.4	3.58	0.489	0.024	0.203	1.56	0.21	18/04/2019	100.3	3.12	-2.14
MB.B.10895	CVU_carb_4859	Fonte Coberta / Rabaçal	FC17a	15.40	2	2	*Soaresirhynchia bouchardi*	brachiopod	ventral	06/04/2019	17/04/2019	97.8	2.50	0.496	0.007	0.028	1.16	0.15	18/04/2019	99.5	3.23	-2.16
MB.B.10895	CVU_carb_4860	Fonte Coberta / Rabaçal	FC17a	15.40	2	2	*Soaresirhynchia bouchardi*	brachiopod	ventral	06/04/2019	17/04/2019	97.1	2.24	0.492	0.007	0.033	1.02	0.12	18/04/2019	100.2	3.21	-2.25
MB.B.10895	CVU_carb_4861	Fonte Coberta / Rabaçal	FC17a	15.40	2	2	*Soaresirhynchia bouchardi*	brachiopod	ventral	06/04/2019	24/04/2019	93.6	2.76	0.495	0.024	0.297	1.86	0.14	18/04/2019	100.3	3.15	-2.25
MB.B.10895	CVU_carb_4862	Fonte Coberta / Rabaçal	FC17a	15.40	2	2	*Soaresirhynchia bouchardi*	brachiopod	dorsal	06/04/2019	24/04/2019	93.3	2.29	0.500	0.014	0.115	2.31	0.04	18/04/2019	100.7	3.13	-2.11
MB.B.10895	CVU_carb_4863	Fonte Coberta / Rabaçal	FC17a	15.40	2	2	*Soaresirhynchia bouchardi*	brachiopod	dorsal	06/04/2019	24/04/2019	96.5	2.38	0.499	0.011	0.075	1.73	0.14	18/04/2019	100.7	3.20	-2.22
MB.B.10895	CVU_carb_4864	Fonte Coberta / Rabaçal	FC17a	15.40	2	2	*Soaresirhynchia bouchardi*	brachiopod	dorsal	06/04/2019	24/04/2019	96.9	2.61	0.514	0.013	0.097	1.67	0.08	18/04/2019	100.1	3.15	-2.29
MB.B.10896	CVU_carb_4865	Fonte Coberta / Rabaçal	FC17a	15.40	2	3	*Soaresirhynchia bouchardi*	brachiopod	ventral	06/04/2019	24/04/2019	96.1	2.70	0.541	0.014	0.128	1.66	0.22	18/04/2019	100.5	3.22	-2.32
MB.B.10893	CVU_carb_4888	Fonte Coberta / Rabaçal	FC17a	15.40	1	1	*Soaresirhynchia* cf. *flamandi*	brachiopod	ventral	08/04/2019	24/04/2019	95.4	2.44	0.495	0.007	0.023	1.46	0.12	18/04/2019	100.8	3.07	-2.42
MB.B.10893	CVU_carb_4889	Fonte Coberta / Rabaçal	FC17a	15.40	1	1	*Soaresirhynchia* cf. *flamandi*	brachiopod	ventral	08/04/2019	24/04/2019	97.2	2.70	0.508	0.007	0.025	1.54	0.20	18/04/2019	100.1	3.04	-2.39
MB.B.10893	CVU_carb_4890	Fonte Coberta / Rabaçal	FC17a	15.40	1	1	*Soaresirhynchia* cf. *flamandi*	brachiopod	ventral	08/04/2019	24/04/2019	96.8	2.53	0.497	0.010	0.048	1.54	0.21	21/04/2019	96.0	3.14	-2.17
MB.B.10893	CVU_carb_4891	Fonte Coberta / Rabaçal	FC17a	15.40	1	1	*Soaresirhynchia* cf. *flamandi*	brachiopod	dorsal	08/04/2019	24/04/2019	97.2	2.12	0.526	0.015	0.073	1.46	0.18	21/04/2019	98.6	3.09	-2.45
MB.B.10893	CVU_carb_4892	Fonte Coberta / Rabaçal	FC17a	15.40	1	1	*Soaresirhynchia* cf. *flamandi*	brachiopod	dorsal	08/04/2019	24/04/2019	97.1	2.38	0.513	0.013	0.083	1.74	0.16	21/04/2019	99.3	3.18	-2.32
MB.B.10893	CVU_carb_4893	Fonte Coberta / Rabaçal	FC17a	15.40	1	1	*Soaresirhynchia* cf. *flamandi*	brachiopod	dorsal	08/04/2019	24/04/2019	96.7	2.39	0.494	0.010	0.061	1.46	0.19	21/04/2019	99.4	3.11	-2.22
MB.B.10894	CVU_carb_4936	Fonte Coberta / Rabaçal	FC17a	15.40	2	1		bulk rock		14/04/2019	24/04/2019	82.0	25.82	0.456	0.456	13.805	1.74	0.63	21/04/2019	86.6	2.13	-4.17
MB.B.10891	CVU_carb_4894	Fonte Coberta / Rabaçal	FC16e	14.80	1	1	*Soaresirhynchia bouchardi*	brachiopod	dorsal	08/04/2019	24/04/2019	95.0	3.36	0.511	0.019	0.324	1.82	0.11	21/04/2019	100.5	3.39	-2.39

MB.B.10891	CVU_carb_4895	Fonte Coberta / Rabaçal	FC16e	14.80	1	1	*Soaresirhynchia bouchardi*	brachiopod	dorsal	08/04/2019	24/04/2019	97.0	2.68	0.485	0.013	0.068	1.58	0.21	21/04/2019	100.1	3.48	-2.46
MB.B.10891	CVU_carb_4896	Fonte Coberta / Rabaçal	FC16e	14.80	1	1	*Soaresirhynchia bouchardi*	brachiopod	dorsal	08/04/2019	24/04/2019	97.1	2.61	0.485	0.014	0.079	1.67	0.05	21/04/2019	100.1	3.44	-2.56
MB.B.10891	CVU_carb_4897	Fonte Coberta / Rabaçal	FC16e	14.80	1	1	*Soaresirhynchia bouchardi*	brachiopod	ventral	08/04/2019	24/04/2019	97.2	2.79	0.482	0.017	0.158	1.50	0.15	21/04/2019	101.0	3.33	-2.51
MB.B.10891	CVU_carb_4898	Fonte Coberta / Rabaçal	FC16e	14.80	1	1	*Soaresirhynchia bouchardi*	brachiopod	ventral	08/04/2019	24/04/2019	96.5	3.07	0.503	0.022	0.327	1.83	0.12	21/04/2019	100.4	3.35	-2.47
MB.B.10891	CVU_carb_4899	Fonte Coberta / Rabaçal	FC16e	14.80	1	1	*Soaresirhynchia bouchardi*	brachiopod	ventral	08/04/2019	24/04/2019	97.1	3.29	0.507	0.020	0.183	2.01	0.10	21/04/2019	100.3	3.38	-2.71
MB.B.10892	CVU_carb_4939	Fonte Coberta / Rabaçal	FC16e	14.80	1	2		bulk rock		14/04/2019	24/04/2019	46.2	41.14	0.524	0.861	32.191	2.76	2.84	21/04/2019	47.0	0.96	-3.97
MB.B.10889	CVU_carb_4819	Fonte Coberta / Rabaçal	FC16d	14.20	1	1	*Soaresirhynchia bouchardi*	brachiopod	ventral	25/03/2019	17/04/2019	97.8	3.47	0.529	0.009	0.074	1.90	0.22	30/03/2019	100.5	2.49	-2.49
MB.B.10889	CVU_carb_4820	Fonte Coberta / Rabaçal	FC16d	14.20	1	1	*Soaresirhynchia bouchardi*	brachiopod	ventral	25/03/2019	17/04/2019	96.9	3.01	0.529	0.014	0.190	1.27	0.22	30/03/2019	99.8	2.42	-2.58
MB.B.10889	CVU_carb_4821	Fonte Coberta / Rabaçal	FC16d	14.20	1	1	*Soaresirhynchia bouchardi*	brachiopod	ventral	25/03/2019	17/04/2019	97.0	4.68	0.593	0.011	0.092	2.22	0.21	30/03/2019	100.6	2.24	-2.59
MB.B.10890	CVU_carb_4822	Fonte Coberta / Rabaçal	FC16d	14.20	1	2	*Soaresirhynchia bouchardi*	brachiopod	dorsal	25/03/2019	17/04/2019	97.4	3.30	0.499	0.030	0.366	1.62	0.07	30/03/2019	100.9	2.35	-2.58
MB.B.10890	CVU_carb_4823	Fonte Coberta / Rabaçal	FC16d	14.20	1	2	*Soaresirhynchia bouchardi*	brachiopod	dorsal	25/03/2019	17/04/2019	97.4	2.86	0.491	0.014	0.146	1.27	0.22	30/03/2019	100.5	2.49	-2.55
MB.B.10890	CVU_carb_4824	Fonte Coberta / Rabaçal	FC16d	14.20	1	2	*Soaresirhynchia bouchardi*	brachiopod	ventral	25/03/2019	17/04/2019	97.0	2.60	0.492	0.017	0.184	1.14	0.20	30/03/2019	100.1	2.46	-2.58
MB.B.10890	CVU_carb_4825	Fonte Coberta / Rabaçal	FC16d	14.20	1	2	*Soaresirhynchia bouchardi*	brachiopod	ventral	25/03/2019	17/04/2019	97.0	2.59	0.485	0.014	0.120	1.46	0.06	30/03/2019	100.1	2.39	-2.61
MB.B.10889	CVU_carb_4948	Fonte Coberta / Rabaçal	FC16d	14.20	1	1		bulk rock		14/04/2019	24/04/2019	70.7	28.73	0.465	0.493	19.028	1.95	1.03	21/04/2019	73.7	1.72	-4.18
MB.B.20343	CVU_carb_4928	Fonte Coberta / Rabaçal	FC16c	13.75	1	1		bulk rock		14/04/2019	24/04/2019	57.0	28.98	0.913	0.425	13.630	2.09	1.77	21/04/2019	59.6	0.48	-4.15
MB.B.10885	CVU_carb_4878	Fonte Coberta / Rabaçal	FC14c	7.30	1	1	*Nannirhynchia pygmaea*	brachiopod	ventral	07/04/2019	24/04/2019	92.5	1.92	0.499	0.031	0.237	1.43	0.22	18/04/2019	100.1	3.63	-1.60
MB.B.20341	CVU_carb_4906	Fonte Coberta / Rabaçal	FC14c	7.30	2	2	*Harpax spinosa*	bivalve		12/04/2019	24/04/2019	95.7	11.43	0.894	0.153	0.792	3.57	0.26	21/04/2019	100.7	2.66	-1.94
MB.B.10885	CVU_carb_4930	Fonte Coberta / Rabaçal	FC14c	7.30	1	1		bulk rock		14/04/2019	24/04/2019	68.1	27.30	0.727	0.508	19.642	1.79	1.01	21/04/2019	71.4	1.86	-3.85
MB.B.10882	CVU_carb_4885	Fonte Coberta / Rabaçal	FC14b	6.90	1	1	*Nannirhynchia pygmaea*	brachiopod	ventral	08/04/2019	24/04/2019	97.6	2.09	0.568	0.018	0.053	1.77	0.18	18/04/2019	100.1	3.85	-1.55
MB.B.10882	CVU_carb_4886	Fonte Coberta / Rabaçal	FC14b	6.90	1	1	*Nannirhynchia pygmaea*	brachiopod	dorsal	08/04/2019	24/04/2019	97.5	1.68	0.529	0.020	0.122	1.32	0.09	18/04/2019	100.6	3.91	-1.67
MB.B.10883	CVU_carb_4887	Fonte Coberta / Rabaçal	FC14b	6.90	1	2	*Nannirhynchia pygmaea*	brachiopod		08/04/2019	24/04/2019	97.3	1.62	0.543	0.016	0.061	1.10	0.21	18/04/2019	100.3	3.79	-1.61
MB.B.10883	CVU_carb_4940	Fonte Coberta / Rabaçal	FC14b	6.90	1	2		bulk rock		14/04/2019	24/04/2019	76.5	23.86	0.515	0.441	13.650	1.71	1.60	21/04/2019	78.9	2.03	-3.96
MB.B.10879	CVU_carb_4837	Fonte Coberta / Rabaçal	FC13e	6.10	1	1	*Nannirhynchia pygmaea*	brachiopod		05/04/2019	17/04/2019	97.8	1.65	0.557	0.013	0.087	1.08	0.08	18/04/2019	97.2	4.21	-1.71
MB.B.10879	CVU_carb_4838	Fonte Coberta / Rabaçal	FC13e	6.10	1	1	*Nannirhynchia pygmaea*	brachiopod	other	05/04/2019	17/04/2019	96.2	1.86	0.543	0.008	0.027	1.03	0.21	18/04/2019	100.3	4.30	-1.61
MB.B.10879	CVU_carb_4839	Fonte Coberta / Rabaçal	FC13e	6.10	1	1	*Nannirhynchia pygmaea*	brachiopod	other	05/04/2019	17/04/2019	96.8	1.97	0.538	0.009	0.049	1.31	0.13	18/04/2019	98.6	4.22	-1.64
MB.B.10879	CVU_carb_4840	Fonte Coberta / Rabaçal	FC13e	6.10	1	1	*Nannirhynchia pygmaea*	brachiopod	other	05/04/2019	17/04/2019	96.5	2.29	0.562	0.015	0.166	1.55	0.10	18/04/2019	100.2	4.35	-1.46
MB.B.10880	CVU_carb_4841	Fonte Coberta / Rabaçal	FC13e	6.10	1	2	*Nannirhynchia pygmaea*	brachiopod	ventral	05/04/2019	17/04/2019	97.0	1.14	0.574	0.015	0.059	0.89	0.10	18/04/2019	100.4	4.30	-1.47
MB.B.10880	CVU_carb_4842	Fonte Coberta / Rabaçal	FC13e	6.10	1	2	*Nannirhynchia pygmaea*	brachiopod	ventral	05/04/2019	17/04/2019	97.2	1.36	0.512	0.007	0.023	0.80	0.11	18/04/2019	100.2	4.39	-1.41
MB.B.10880	CVU_carb_4843	Fonte Coberta / Rabaçal	FC13e	6.10	1	2	*Nannirhynchia pygmaea*	brachiopod	ventral	05/04/2019	17/04/2019	97.1	1.23	0.542	0.014	0.046	0.86	0.14	18/04/2019	99.5	4.36	-1.63
MB.B.10880	CVU_carb_4844	Fonte Coberta / Rabaçal	FC13e	6.10	1	2	*Nannirhynchia pygmaea*	brachiopod	dorsal	05/04/2019	17/04/2019	97.8	1.68	0.538	0.024	0.237	0.97	0.19	18/04/2019	101.3	3.90	-2.11
MB.B.10881	CVU_carb_4845	Fonte Coberta / Rabaçal	FC13e	6.10	1	3	*Nannirhynchia pygmaea*	brachiopod	ventral	05/04/2019	17/04/2019	97.2	1.62	0.499	0.008	0.030	0.92	0.08	18/04/2019	99.6	3.98	-1.69
MB.B.10881	CVU_carb_4846	Fonte Coberta / Rabaçal	FC13e	6.10	1	3	*Nannirhynchia pygmaea*	brachiopod	ventral	05/04/2019	17/04/2019	97.0	1.61	0.511	0.017	0.033	0.81	0.08	18/04/2019	99.7	4.20	-1.43
MB.B.10876	CVU_carb_4909	Fonte Coberta / Rabaçal	FC13d	5.50	1	1	*Nannirhynchia pygmaea*	brachiopod		12/04/2019	24/04/2019	96.7	1.91	0.554	0.010	0.035	1.46	0.11	21/04/2019	99.9	4.31	-1.49
MB.B.10876	CVU_carb_4910	Fonte Coberta / Rabaçal	FC13d	5.50	1	1	*Nannirhynchia pygmaea*	brachiopod		12/04/2019	24/04/2019	97.3	1.97	0.519	0.008	0.023	1.33	0.12	21/04/2019	100.6	4.21	-1.44
MB.B.10877	CVU_carb_4911	Fonte Coberta / Rabaçal	FC13d	5.50	1	2	*Nannirhynchia pygmaea*	brachiopod		12/04/2019	24/04/2019	96.7	2.27	0.541	0.017	0.188	1.59	0.13	21/04/2019	100.0	4.13	-1.55
MB.B.10878	CVU_carb_4912	Fonte Coberta / Rabaçal	FC13d	5.50	1	3	*Nannirhynchia pygmaea*	brachiopod		12/04/2019	24/04/2019	96.8	1.61	0.582	0.014	0.076	1.20	0.17	21/04/2019	100.5	3.75	-1.58
MB.B.20338	CVU_carb_4924	Fonte Coberta / Rabaçal	FC13d	5.50	2	1	*Harpax spinosa*	bivalve		12/04/2019	24/04/2019	95.8	7.90	0.963	0.046	0.550	1.58	0.13	21/04/2019	101.4	3.49	-1.50
MB.B.20338	CVU_carb_4938	Fonte Coberta / Rabaçal	FC13d	5.50	2	1		bulk rock		14/04/2019	24/04/2019	55.1	31.82	0.815	0.486	23.769	2.43	1.73	21/04/2019	57.1	1.88	-3.86
MB.B.20336	CVU_carb_4907	Fonte Coberta / Rabaçal	FC13c	4.90	2	1	*Harpax spinosa*	bivalve		12/04/2019	24/04/2019	95.6	9.88	1.262	0.067	0.331	1.53	0.15	21/04/2019	100.8	3.10	-1.74
MB.B.20336	CVU_carb_4908	Fonte Coberta / Rabaçal	FC13c	4.90	2	1	*Harpax spinosa*	bivalve		12/04/2019	24/04/2019	95.3	10.24	1.033	0.060	0.348	1.52	0.26	21/04/2019	100.5	3.53	-1.54
MB.B.10874	CVU_carb_4913	Fonte Coberta / Rabaçal	FC13c	4.90	1	1	*Nannirhynchia pygmaea*	brachiopod	ventral	12/04/2019	24/04/2019	96.8	1.28	0.555	0.009	0.012	1.07	0.08	21/04/2019	121.4	3.79	-1.54
MB.B.20337	CVU_carb_4946	Fonte Coberta / Rabaçal	FC13c	4.90	2	2		bulk rock		14/04/2019	24/04/2019	60.5	30.33	0.910	0.566	21.210	2.55	2.23	21/04/2019	62.0	1.90	-3.82
MB.B.10873	CVU_carb_4941	Fonte Coberta / Rabaçal	FC13b	4.25	1	1		bulk rock		14/04/2019	24/04/2019	68.0	28.29	0.609	0.428	19.906	2.54	1.26	21/04/2019	71.1	1.95	-3.80
MB.B.10869	CVU_carb_4879	Fonte Coberta / Rabaçal	FC13a	3.30	1	1	*Cirpa fallax*	brachiopod	ventral	07/04/2019	24/04/2019	97.7	1.90	0.496	0.081	0.051	1.20	0.04	18/04/2019	100.4	4.03	-1.43
MB.B.10869	CVU_carb_4880	Fonte Coberta / Rabaçal	FC13a	3.30	1	1	*Cirpa fallax*	brachiopod	ventral	07/04/2019	24/04/2019	96.7	1.52	0.471	0.015	0.094	1.24	0.14	18/04/2019	100.3	4.03	-1.31
MB.B.10869	CVU_carb_4881	Fonte Coberta / Rabaçal	FC13a	3.30	1	1	*Cirpa fallax*	brachiopod	ventral	07/04/2019	24/04/2019	97.3	1.76	0.486	0.013	0.047	1.23	0.11	18/04/2019	100.5	4.05	-1.37
MB.B.10869	CVU_carb_4882	Fonte Coberta / Rabaçal	FC13a	3.30	1	1	*Cirpa fallax*	brachiopod	dorsal	07/04/2019	24/04/2019	97.2	1.50	0.499	0.011	0.037	1.04	0.11	18/04/2019	101.7	4.12	-1.34
MB.B.10869	CVU_carb_4883	Fonte Coberta / Rabaçal	FC13a	3.30	1	1	*Cirpa fallax*	brachiopod	dorsal	07/04/2019	24/04/2019	97.7	1.56	0.479	0.012	0.061	1.46	0.18	18/04/2019	81.2	4.14	-1.22
MB.B.10869	CVU_carb_4884	Fonte Coberta / Rabaçal	FC13a	3.30	1	1	*Cirpa fallax*	brachiopod	dorsal	07/04/2019	24/04/2019	97.4	1.76	0.479	0.013	0.088	1.32	0.13	18/04/2019	101.0	4.07	-1.41
MB.B.10870	CVU_carb_4927	Fonte Coberta / Rabaçal	FC13a	3.30	2	1	*Cirpa fallax*	brachiopod	ventral	13/04/2019	24/04/2019	97.0	0.93	0.553	0.068	0.145	1.94	0.01	21/04/2019	100.4	3.71	-1.41
MB.B.10864	CVU_carb_4866	Fonte Coberta / Rabaçal	FC12	2.35	2	1	*Cirpa fallax*	brachiopod	ventral	07/04/2019	24/04/2019	97.3	2.14	0.492	0.020	0.053	1.85	0.13	18/04/2019	100.4	3.56	-1.27
MB.B.10864	CVU_carb_4867	Fonte Coberta / Rabaçal	FC12	2.35	2	1	*Cirpa fallax*	brachiopod	dorsal	07/04/2019	24/04/2019	96.0	2.39	0.505	0.035	0.188	1.51	0.14	18/04/2019	99.8	3.56	-1.45
MB.B.10865	CVU_carb_4868	Fonte Coberta / Rabaçal	FC12	2.35	2	2	*Cirpa fallax*	brachiopod	ventral	07/04/2019	24/04/2019	96.9	1.30	0.543	0.016	0.078	1.27	0.09	18/04/2019	99.8	4.08	-1.40
MB.B.10865	CVU_carb_4869	Fonte Coberta / Rabaçal	FC12	2.35	2	2	*Cirpa fallax*	brachiopod	dorsal	07/04/2019	24/04/2019	96.5	1.13	0.515	0.016	0.078	0.78	0.07	18/04/2019	99.8	4.20	-1.32
MB.B.10862	CVU_carb_4914	Fonte Coberta / Rabaçal	FC12	2.35	1	1	*Soaresirhynchia bouchardi*	brachiopod	ventral	12/04/2019	24/04/2019	96.9	3.49	0.561	0.017	0.121	1.57	0.20	21/04/2019	100.6	5.58	-1.69
MB.B.10863	CVU_carb_4915	Fonte Coberta / Rabaçal	FC12	2.35	1	2	*Soaresirhynchia bouchardi*	brachiopod	ventral	12/04/2019	24/04/2019	97.0	5.18	0.654	0.032	0.375	2.39	0.24	21/04/2019	100.7	4.32	-2.36
MB.B.10863	CVU_carb_4916	Fonte Coberta / Rabaçal	FC12	2.35	1	2	*Soaresirhynchia bouchardi*	brachiopod	ventral	12/04/2019	24/04/2019	96.6	4.37	0.576	0.014	0.108	2.26	0.12	21/04/2019	101.3	4.36	-2.33
MB.B.10864	CVU_carb_4949	Fonte Coberta / Rabaçal	FC12	2.35	2	1		bulk rock		14/04/2019	24/04/2019	79.7	24.51	0.547	0.407	12.830	2.01	0.39	21/04/2019	82.8	1.78	-3.69
MB.B.20331	CVU_carb_4900	Fonte Coberta / Rabaçal	FC11	1.85	1	1	*Harpax spinosa*	bivalve		08/04/2019	24/04/2019	95.9	9.41	0.843	0.169	0.894	2.67	0.26	21/04/2019	99.9	2.03	-1.93
MB.B.20331	CVU_carb_4901	Fonte Coberta / Rabaçal	FC11	1.85	1	1	*Harpax spinosa*	bivalve		08/04/2019	24/04/2019	96.4	7.30	0.935	0.101	0.363	1.44	0.29	21/04/2019	100.1	1.91	-1.48
MB.B.20331	CVU_carb_4902	Fonte Coberta / Rabaçal	FC11	1.85	1	1	*Harpax spinosa*	bivalve		08/04/2019	24/04/2019	96.3	8.70	0.819	0.184	0.789	1.55	0.71	21/04/2019	100.4	1.96	-1.55
MB.B.20332	CVU_carb_4933	Fonte Coberta / Rabaçal	FC11	1.85	1	2		bulk rock		14/04/2019	24/04/2019	55.8	36.54	0.585	0.524	26.772	2.38	1.93	21/04/2019	58.1	0.51	-3.95
MB.B.10859	CVU_carb_4826	Fonte Coberta / Rabaçal	FC10	1.45	4	1	*Cirpa fallax*	brachiopod	ventral	25/03/2019	17/04/2019	96.8	2.54	0.474	0.050	0.568	1.36	0.31	30/03/2019	99.0	2.62	-2.30
MB.B.10859	CVU_carb_4827	Fonte Coberta / Rabaçal	FC10	1.45	4	1	*Cirpa fallax*	brachiopod	ventral	25/03/2019	17/04/2019	97.4	1.97	0.488	0.013	0.037	1.16	0.27	30/03/2019	100.4	3.27	-1.13
MB.B.10859	CVU_carb_4828	Fonte Coberta / Rabaçal	FC10	1.45	4	1	*Cirpa fallax*	brachiopod	ventral	25/03/2019	17/04/2019	97.3	2.06	0.512	0.020	0.086	1.30	0.25	30/03/2019	99.8	3.03	-1.20
MB.B.10859	CVU_carb_4829	Fonte Coberta / Rabaçal	FC10	1.45	4	1	*Cirpa fallax*	brachiopod	dorsal	25/03/2019	17/04/2019	97.4	2.42	0.548	0.016	0.053	1.57	0.26	30/03/2019	99.9	3.26	-1.06
MB.B.10859	CVU_carb_4830	Fonte Coberta / Rabaçal	FC10	1.45	4	1	*Cirpa fallax*	brachiopod	dorsal	25/03/2019	17/04/2019	97.1	2.31	0.551	0.021	0.058	1.57	0.12	30/03/2019	100.2	3.18	-1.18
MB.B.10859	CVU_carb_4831	Fonte Coberta / Rabaçal	FC10	1.45	4	1	*Cirpa fallax*	brachiopod	dorsal	25/03/2019	17/04/2019	97.3	2.21	0.510	0.019	0.091	1.38	0.10	18/04/2019	91.1	3.12	-1.15
MB.B.10860	CVU_carb_4832	Fonte Coberta / Rabaçal	FC10	1.45	4	2	*Cirpa fallax*	brachiopod	ventral	05/04/2019	17/04/2019	97.3	2.30	0.529	0.014	0.110	1.36	0.14	18/04/2019	96.2	2.61	-1.76
MB.B.10860	CVU_carb_4833	Fonte Coberta / Rabaçal	FC10	1.45	4	2	*Cirpa fallax*	brachiopod	ventral	05/04/2019	17/04/2019	97.2	3.32	0.539	0.010	0.056	1.71	0.08	18/04/2019	99.8	2.78	-1.14
MB.B.10860	CVU_carb_4834	Fonte Coberta / Rabaçal	FC10	1.45	4	2	*Cirpa fallax*	brachiopod	dorsal	05/04/2019	17/04/2019	97.5	2.44	0.503	0.007	0.035	1.38	0.22	18/04/2019	100.1	2.98	-1.22
MB.B.10860	CVU_carb_4835	Fonte Coberta / Rabaçal	FC10	1.45	4	2	*Cirpa fallax*	brachiopod	dorsal	05/04/2019	17/04/2019	97.8	2.29	0.509	0.008	0.053	1.46	0.11	18/04/2019	99.4	2.82	-1.49
MB.B.10860	CVU_carb_4836	Fonte Coberta / Rabaçal	FC10	1.45	4	2	*Cirpa fallax*	brachiopod	dorsal	05/04/2019	17/04/2019	97.4	2.45	0.518	0.030	0.272	1.36	0.08	18/04/2019	99.3	2.71	-1.87
MB.B.20329	CVU_carb_4925	Fonte Coberta / Rabaçal	FC10	1.45	3	1	*Harpax spinosa*	bivalve		12/04/2019	24/04/2019	95.2	10.17	1.036	0.102	0.602	2.57	0.13	21/04/2019	100.3	1.80	-2.12
MB.B.10856	CVU_carb_4926	Fonte Coberta / Rabaçal	FC10	1.45	2	1	*Nannirhynchia pygmaea*	brachiopod	ventral	13/04/2019	24/04/2019	96.8	2.30	0.535	0.011	0.050	1.36	0.05	21/04/2019	100.6	2.83	-1.44
MB.B.20329	CVU_carb_4934	Fonte Coberta / Rabaçal	FC10	1.45	3	1		bulk rock		14/04/2019	24/04/2019	55.1	26.10	0.628	0.463	16.469	2.31	1.39	21/04/2019	57.4	0.33	-3.80
MB.B.10851	CVU_carb_4850	Fonte Coberta / Rabaçal	FC09	1.05	2	1	*Nannirhynchia pygmaea*	brachiopod	dorsal	05/04/2019	17/04/2019	97.6	2.98	0.489	0.019	0.065	1.52	0.13	18/04/2019	100.5	2.39	-1.65
MB.B.10852	CVU_carb_4851	Fonte Coberta / Rabaçal	FC09	1.05	2	2	*Nannirhynchia pygmaea*	brachiopod	dorsal	05/04/2019	17/04/2019	97.4	2.56	0.526	0.008	0.027	1.17	0.23	18/04/2019	99.8	2.50	-1.41
MB.B.10853	CVU_carb_4852	Fonte Coberta / Rabaçal	FC09	1.05	2	3	*Nannirhynchia pygmaea*	brachiopod		05/04/2019	17/04/2019	97.6	1.87	0.541	0.013	0.086	0.91	0.12	18/04/2019	100.3	2.32	-1.29
MB.B.10851	CVU_carb_4943	Fonte Coberta / Rabaçal	FC09	1.05	2	1		bulk rock		14/04/2019	24/04/2019	68.8	29.04	0.619	0.403	18.074	2.86	1.18	21/04/2019	71.4	1.34	-3.92
MB.B.10847	CVU_carb_4877	Fonte Coberta / Rabaçal	FC08	0.80	3	1	*Nannirhynchia pygmaea*	brachiopod		07/04/2019	24/04/2019	95.7	3.29	0.555	0.047	0.527	1.54	0.23	18/04/2019	98.1	1.87	-1.43
MB.B.20328	CVU_carb_4929	Fonte Coberta / Rabaçal	FC08	0.80	5	1		bulk rock		14/04/2019	24/04/2019	56.2	37.12	0.893	0.534	23.942	2.83	1.24	21/04/2019	59.0	1.05	-3.63
MB.B.20326	CVU_carb_4917	Fonte Coberta / Rabaçal	FC07	0.40	1	1	*Harpax spinosa*	bivalve		12/04/2019	24/04/2019	96.1	8.15	0.958	0.078	0.263	1.43	0.20	21/04/2019	100.6	1.57	-0.93
MB.B.20326	CVU_carb_4918	Fonte Coberta / Rabaçal	FC07	0.40	1	1	*Harpax spinosa*	bivalve		12/04/2019	24/04/2019	95.7	8.76	1.018	0.097	0.362	2.24	0.18	21/04/2019	100.5	1.25	-1.23
MB.B.20326	CVU_carb_4919	Fonte Coberta / Rabaçal	FC07	0.40	1	1	*Harpax spinosa*	bivalve		12/04/2019	24/04/2019	95.9	9.64	1.046	0.081	0.291	2.32	0.21	21/04/2019	101.3	1.50	-1.31
MB.B.20326	CVU_carb_4937	Fonte Coberta / Rabaçal	FC07	0.40	1	1		bulk rock		14/04/2019	24/04/2019	61.9	25.34	0.548	0.498	15.097	2.07	1.21	21/04/2019	64.1	0.06	-3.89
MB.B.20325	CVU_carb_4847	Fonte Coberta / Rabaçal	FC06	0.00	3	1	*Harpax spinosa*	bivalve		05/04/2019	17/04/2019	95.8	11.96	0.968	0.049	0.395	1.67	0.11	18/04/2019	100.1	1.99	-0.64
MB.B.20325	CVU_carb_4848	Fonte Coberta / Rabaçal	FC06	0.00	3	1	*Harpax spinosa*	bivalve		05/04/2019	17/04/2019	96.2	9.04	1.048	0.058	0.438	2.14	0.20	18/04/2019	100.9	1.99	-0.71
MB.B.20325	CVU_carb_4849	Fonte Coberta / Rabaçal	FC06	0.00	3	1	*Harpax spinosa*	bivalve		05/04/2019	17/04/2019	96.1	9.50	1.024	0.048	0.371	1.53	0.24	18/04/2019	100.5	1.77	-0.76
MB.B.10843	CVU_carb_4853	Fonte Coberta / Rabaçal	FC06	0.00	1	1	*Lobothyris arcta*	brachiopod	ventral	05/04/2019	17/04/2019	93.6	7.56	1.274	0.039	0.575	15.93	0.23	18/04/2019	97.3	1.17	-0.51
MB.B.10843	CVU_carb_4854	Fonte Coberta / Rabaçal	FC06	0.00	1	1	*Lobothyris arcta*	brachiopod	ventral	05/04/2019	17/04/2019	94.6	7.36	1.181	0.045	0.777	14.02	0.40	18/04/2019	96.1	1.13	-0.67
MB.B.10843	CVU_carb_4855	Fonte Coberta / Rabaçal	FC06	0.00	1	1	*Lobothyris arcta*	brachiopod	ventral	05/04/2019	17/04/2019	94.1	8.24	1.232	0.047	0.726	14.40	0.18	18/04/2019	97.0	1.23	-0.44
MB.B.10846	CVU_carb_4920	Fonte Coberta / Rabaçal	FC06	0.00	2	1	*Cirpa fallax*	brachiopod	ventral	12/04/2019	24/04/2019	97.2	2.45	0.465	0.021	0.036	1.81	0.26	21/04/2019	100.5	1.72	-1.05
MB.B.10846	CVU_carb_4921	Fonte Coberta / Rabaçal	FC06	0.00	2	1	*Cirpa fallax*	brachiopod	dorsal	12/04/2019	24/04/2019	97.1	2.42	0.484	0.128	25.719	1.45	0.02	21/04/2019	100.6	1.63	-1.24
MB.B.10846	CVU_carb_4922	Fonte Coberta / Rabaçal	FC06	0.00	2	1	*Cirpa fallax*	brachiopod	dorsal	12/04/2019	24/04/2019	97.6	2.27	0.487	0.013	0.047	1.44	0.06	21/04/2019	101.1	1.70	-0.96
MB.B.10846	CVU_carb_4923	Fonte Coberta / Rabaçal	FC06	0.00	2	1	*Cirpa fallax*	brachiopod	dorsal	12/04/2019	24/04/2019	97.4	2.29	0.494	0.017	0.068	1.55	0.04	21/04/2019	101.0	1.67	-1.12
MB.B.10846	CVU_carb_4945	Fonte Coberta / Rabaçal	FC06	0.00	2	1		bulk rock		14/04/2019	24/04/2019	78.5	17.33	0.368	0.336	31.252	37.79	0.82	21/04/2019	81.4	0.57	-3.15

Table S5: Median isotopic variability in specimens of brachiopods from the two studied sections computed from specimens for which at least four measurements of well preserved shell calcite are available.

**genus**	**locality**	**specimens number**	**median 2sd $\delta^{13}C$**	**median 2sd $\delta^{18}O$**
Choffatirhynchia	Barranco de la Cañada	11	0.40	0.31
Gibbirhynchia	Barranco de la Cañada	2	0.41	0.49
Homoeorhynchia	Barranco de la Cañada	6	0.31	0.26
Quadratirhynchia	Barranco de la Cañada	3	0.23	0.39
Soaresirhynchia	Barranco de la Cañada	6	0.12	0.20
Cirpa	Fonte Coberta / Rabaçal	3	0.20	0.14
Homoeorhynchia	Fonte Coberta / Rabaçal	1	0.14	0.18
Nannirhynchia †	Fonte Coberta / Rabaçal	2	0.11	0.22
Soaresirhynchia	Fonte Coberta / Rabaçal	4	0.11	0.18

†: one doubtful datum (CVU_carb_4844) excluded

Table S6: Average $CaCO_3$ concentrations, Mg/Ca and Sr/Ca ratios computed from well-preserved samples for individual rhynchonellid genera from the two studied sections.

genus	locality	samples	average $CaCO_3$		average Mg/Ca		average Sr/Ca	
		number	% vs CAR	2 se	mmol/mol	2 se	mmol/mol	2 se
Choffatirhynchia	Barranco de la Cañada	74	99.1	0.2	6.63	0.31	0.994	0.024
Gibbirhynchia	Barranco de la Cañada	14	97.6	0.8	5.32	0.48	1.140	0.026
Homoeorhynchia	Barranco de la Cañada	91	98.7	0.2	4.82	0.30	1.084	0.020
Quadratirhynchia	Barranco de la Cañada	37 (36)	98.7	0.3	4.85	0.37	1.002	0.024
Soaresirhynchia	Barranco de la Cañada	100 (99)	100.3	0.2	2.63	0.10	0.533	0.006
Cirpa	Fonte Coberta / Rabaçal	22 (20)	99.9	0.5	2.08	0.21	0.506	0.011
Homoeorhynchia	Fonte Coberta / Rabaçal	4	99.1	0.6	5.49	0.66	0.948	0.021
Nannirhynchia	Fonte Coberta / Rabaçal	23 (22)	100.0	0.4	1.85	0.18	0.537	0.010
Soaresirhynchia	Fonte Coberta / Rabaçal	36	100.1	0.3	2.94	0.24	0.514	0.012

Samples numbers in brackets refer to numbers of samples from which average $CaCO_3$ concentration was computed. Excluded samples deviated from averages by > 5 % and deviations are ascribed to erroneous weights.

Table S7: Aggregated isotope data and calculated palaeotemperatures using the equations of Anderson and Arthur (1983) assuming seawater $\delta^{18}O$ of -1 ‰ vs V-SMOW and Brand et al., (2013) additonally using the average magnesium content of 0.42 mol% in the macrofossil calcite. Ages are calculcated from the Geologic Timescale 2012, assuming constant sedimentaiton rates within each ammonite zone.

sample name	height m	n	$\delta^{13}C$ ‰ VPDB	2sd	2se	$\delta^{18}O$ ‰ VPDB	2sd	2se	T (B'13) °C	T (A'83) °C	Age (GT'12) Ma
BC22	26.70	3	3.14	0.10	0.06	-2.47	0.38	0.22	22.8	22.4	179.94
BC21	25.55	5	2.89	0.37	0.17	-2.54	0.86	0.38	23.0	22.7	180.05
BC20	24.55	9	2.72	0.52	0.17	-2.28	0.63	0.21	22.1	21.5	180.14
BC19	23.45	2	1.94	0.32	0.23	-2.47	0.01	0.01	22.7	22.4	180.24
BC18	22.65	3	2.30	0.34	0.20	-2.71	0.29	0.17	23.6	23.4	180.32
BC17	21.45	6	2.46	0.14	0.06	-2.07	0.17	0.07	21.4	20.6	180.43
BC14	19.35	11	3.27	0.57	0.17	-2.65	0.48	0.14	23.4	23.2	180.63
BC10	17.30	4	3.23	0.28	0.14	-2.50	0.44	0.22	22.9	22.5	180.82
BC09	16.70	2	3.17	0.13	0.09	-2.15	0.01	0.01	21.6	20.9	180.87
BC08	16.05	13	3.47	0.52	0.15	-2.57	0.62	0.17	23.1	22.8	180.93
BC07	15.25	8	4.17	0.21	0.07	-2.67	0.26	0.09	23.4	23.3	181.01
BC06	14.85	22	4.53	0.38	0.08	-2.75	0.67	0.14	23.7	23.7	181.04
BC05	14.25	4	4.61	0.12	0.06	-2.04	0.46	0.23	21.3	20.5	181.10
BC04	13.75	21	3.93	0.44	0.10	-1.75	0.67	0.15	20.3	19.2	181.15
BC03	13.20	13	3.95	0.67	0.19	-2.11	0.70	0.19	21.5	20.8	181.20
BC02	12.60	50	3.99	0.68	0.10	-1.91	0.67	0.10	20.8	19.9	181.25
BC01b	12.35	14	4.49	0.78	0.21	-2.60	0.96	0.26	23.2	23.0	181.28
BC01a	12.10	5	5.13	0.42	0.19	-2.34	0.11	0.05	22.3	21.8	181.30
C37a	11.30	8	4.39	0.30	0.11	-3.05	0.72	0.25	24.7	25.0	181.37
C33b	11.10	7	4.24	0.17	0.06	-3.00	0.48	0.18	24.6	24.8	181.39
C33a	10.80	4	4.60	0.28	0.14	-3.30	0.29	0.14	25.6	26.2	181.42
C30	10.20	3	4.38	0.59	0.34	-2.58	0.68	0.39	23.1	22.9	181.48
C28	9.40	25	2.69	0.35	0.07	-2.97	0.67	0.13	24.5	24.7	181.55
C26	8.90	21	2.56	0.55	0.12	-3.08	0.76	0.17	24.9	25.2	181.60
C25a	8.40	30	2.16	0.46	0.08	-3.26	0.36	0.07	25.5	26.0	181.64
C22	7.80	5	-0.36	0.23	0.10	-3.36	0.32	0.14	25.8	26.5	181.70
C20	6.70	2	1.13	0.10	0.07	-3.03	0.37	0.26	24.7	24.9	181.84
C19	6.20	16	2.26	0.63	0.16	-2.55	0.37	0.09	23.0	22.7	181.91
C18c	5.90	22	2.94	0.66	0.14	-1.88	0.73	0.15	20.7	19.7	181.94
C18b	5.60	17	3.13	0.53	0.13	-1.93	0.39	0.09	20.9	20.0	181.98
C18a	5.20	5	3.09	0.33	0.15	-2.01	0.43	0.19	21.1	20.3	182.03
C15b	4.60	2	2.97	0.24	0.17	-1.94	0.14	0.10	20.9	20.0	182.11
C15a	4.00	22	2.60	0.65	0.14	-2.21	0.59	0.13	21.8	21.2	182.19
C12b	3.40	6	1.98	1.39	0.57	-2.24	0.58	0.24	21.9	21.3	182.26
C12a	2.80	16	1.59	0.49	0.12	-2.13	0.59	0.15	21.6	20.8	182.34
C07b	1.60	2	0.32	0.34	0.24	-2.00	0.68	0.48	21.1	20.3	182.49
C07a	0.80	6	0.47	0.80	0.33	-2.11	0.77	0.31	21.5	20.8	182.60
C05	0.40	15	0.46	0.61	0.16	-1.95	0.55	0.14	20.9	20.0	182.65

Table S8: Quantities of Carbon required to explain T-OAE carbon isotope excursions.

		Amount of carbon required to generate negative isotopic excursion, GtC		
Relative mass of ocean-atmosphere exchangeable carbon MT (pre-perterbation mass of total, combined organic and inorganic exchangeable carbon, relative to present $M_{T0} = M_{inorganic} + M_{organic} \cong 38{,}062$ GtC)		$1 * M_{T0}$	$2 * M_{T0}$	$4 * M_{T0}$
M_{CH4} (clathrate)	$\delta^{13}C_{CH4\ hyd} = -60$ ‰	1765	3531	7062
M_{CO2} (thermogenic)	$\delta^{13}C_{CO2\ therm} = -27$ ‰	3943	7868	15737
M_{CH4} (thermogenic)	$\delta^{13}C_{CH4\ therm} = -25$ ‰	4251	8501	17003
	$\delta^{13}C_{CH4\ therm} = -35$ ‰	3031	6063	12126
M_{CO2} (magma)	$\delta^{13}C_{CO2\ magma} = -5$ ‰	21729	43457	86916
	$\delta^{13}C_{CO2\ magma} = -7$ ‰	15937	30795	61590
Total mass of organic carbon necessary to explain PCIE GtC		7476	14953	29907
Relative organic carbon burial flux x, where burial flux = $x * \int_0^{\Delta t_{data},PCIE} mocb_0 dt$		0.415	0.83	1.66

Table S9: Proxy Inversion model parameters

**Abbreviation**	**Meaning**	**Baseline value/source** see COPSE (Bergman et al, 2004, Lenton et al, 2018) Geocarb (Berner, 1994, Royer et al, 2014), Geocarbsulf (Berner, 2006), Redfield revisited (Lenton & Watson, 2000)
C	Carbonate carbon rock reservoir	$5.0 * 10^{21}$ mol
G	Organic carbon rock reservoir	$1.5 * 10^{21}$ mol
carbw	Carbonate weathering flux from land surface	$carbw_0 = 13.25 * 10^{12}$ $molyr^{-1}$
ccdeg	CO_2 degassing flux from the subduction of carbonate	$ccdeg_0 = 6.6 * 10^{12}$ $molyr^{-1}$ (COPSE)
ocdeg	CO_2 degassing flux from the subduction of organic carbon in rock	$ocdeg_0 = 1.25 * 10^{12}$ $molyr^{-1}$ (COPSE)
mccb	Marine carbonate carbon burial flux	$2.0 * 10^{13}$ mol
mocb	Marine organic carbon burial flux	$mocb_0 = 3.75 * 10^{12}$ mol
oxidw	Oxidative weathering flux of organic carbon in rock on the land surface	$oxidw_0 = 3.75 * 10^{12}$ $molyr^{-1}$
$\delta^{13}C_C$	Isotopic composition of carbonate carbon rock reservoir	$\delta_{C(0)} = 0$
$\delta^{13}C_G$	Isotopic composition of carbonate carbon rock reservoir	$\delta_{G(0)} = -\varepsilon = -24.5$ ‰
$\delta^{13}C_{mccb}$	Isotopic composition of carbonates precipitating in shallow shelf ocean waters (assumed equilibrated with ocean-atmosphere CO_2 reservoir)	Model output, equated with data
ε	Difference between the isotopic composition of the marine organic carbon burial flux mocb and the marine carbonate carbon burial flux mccb. Expressed as a positive number ε > 0, such the isotopic composition of organic matter being buried in shallow ocean sediments is $\delta^{13}C_{org} = \delta^{13}C_{mccb} - \varepsilon < 0$	ε = 24.5 ‰
F_{LIP}	CO_2 release flux associated with large igneous province eruption.	$\int_{t_{LIP\,start}}^{t_{LIP\,end}} F_{LIP}\,dt \le T_{CO_2\,LIP}$, where T_{CO2LIP} is the total emission potential for the whole lifetime of the LIP, and $t_{LIP\ start}$ / $t_{LIP\ end}$ are model parameters describing the time of onset/cessation of LIP associated CO_2 degassing (for most model runs shown, $t_{LIP\,start} - t_{LIP\,end} = 0.45$ Myrs, tuned to match the NCIE).<br>Constrained to be in the within the bounds $6.48 * 10^{17}$ mol C ≤ $T_{CO2\ LIP} \le 1.46 * 10^{18}$ mol
$\delta^{13}C_{LIP}$	Isotopic composition of large igneous province derived CO_2	$(\delta^{13}C_{LIP})_{syn-volcanic} = -5$ ‰ (Cervantes & Wallace 2003)<br>-27 ‰ ≤ $(\delta^{13}C_{LIP})_{thermogenic}$ ≤ +2 ‰ (Jones et al, 2016)
F_{CH4}	CO_2 release flux associated with the oxidation of CH_4 produced during clathrate decomposition.	Parameterized, see text
$\delta^{13}C_{CH4}$	Isotopic composition of CH_4-clathrate	$\delta^{13}C_{CH4} = -60$ ‰(Kvenvolden, 2002)
ESS	Long term Earth system sensitivity (temperature change per of atmospheric CO_2)	ESS = 5 (Geocarbsulf)
W_S	Solar luminosity temperature dependence parameter	$W_S = 7.4$ (Geocarbsulf)
GEOG	Continental mean surface temperature (expressing change over time in exposure of fresh minerals to weathering)	GEOG(183 MA) = 3 (Geocarbsulf)
$ccdeg_0$	Baseline carbonate carbon degassing flux	$ccdeg_0 = 6.65 * 10^{12}$ mol (COPSE)
A	Total ocean-atmosphere CO_2 reservoir size	$A_0 = 3.193 * 10^{18}$ mol (COPSE)
φ	Atmospheric CO_2 fraction	Modern value $\phi_0 = 2.8 * 10^8 /(mol_{atmos} * A_0) = 0.0155$ (COPSE)
δ_C	Average isotopic composition of global carbonate carbon rock reservoir	$\delta_{C(0)} = 0$ ‰
δ_G	Average isotopic composition of global organic carbon rock reservoir	$\delta_{G(0)} = -\varepsilon = -24.5$ ‰
f_{anox}	Global ocean anoxic fraction	$f_{anox\,0} = 0.14$
$\bar{P} = \frac{PO_4}{PO_{4\,0}}$	Normalized, globally averaged marine phosphate concentration	$PO_{4\,0} = 2.2$ μmolkg-1 (Redfield)
phosw	Phosphorous weathering flux from land surface	$phosw_0 = 3.675 * 10^{10}$ $molyr^{-1}$;<br>$phosw = phosw_0\left(f_{silw}\frac{silw}{silw_0} + f_{carbw}\frac{carbw}{carbw_0} + f_{oxidw}\frac{oxidw}{oxidw_0}\right)$
capb	Calcium bound marine phosphate burial	$capb_0 = 1.5 * 10^{10}$ $molyr^{-1}$
fepb	Iron absorbed phosphate burial	$fepb_0 = 0.6 * 10^{10} molyr^{-1}$
mopb	Marine organic phosphorous burial	Scales with mocb as a function of anoxia via constants $CP_{ox} = 217 * CP_{anox} = 4320$ (Van Capellen & Ingall, 1994)

Table S10: Example output for model run.

time<br>Ma	atmospheric CO_2<br>* pre-industrial	silicate weathering<br>mol/a	ocean anoxic<br>fraction	LIP CO_2 flux<br>mol/a	marine PO_4<br>normalized	Fe-ads PO_4 burial<br>mol/a	marine C_{org} burial<br>mol/a	global temperature<br>°C
-250.00	1.00	2.71E+12	0.1400	0	1.00	5.17E+09	3.75E+12	11.8
-250.00	1.00	2.71E+12	0.1400	0	1.00	5.17E+09	3.75E+12	11.8
-250.00	1.00	2.71E+12	0.1400	0	1.00	5.17E+09	3.75E+12	11.8
-250.00	1.00	2.71E+12	0.1400	0	1.00	5.17E+09	3.75E+12	11.8
-250.00	1.00	2.71E+12	0.1400	0	1.00	5.17E+09	3.75E+12	11.8
-250.00	1.00	2.71E+12	0.1400	0	1.00	5.17E+09	3.75E+12	11.8
-250.00	1.00	2.71E+12	0.1400	0	1.00	5.17E+09	3.75E+12	11.8
-250.00	1.00	2.71E+12	0.1400	0	1.00	5.17E+09	3.75E+12	11.8
-250.00	1.00	2.71E+12	0.1400	0	1.00	5.17E+09	3.75E+12	11.8
-250.00	1.00	2.71E+12	0.1400	0	1.00	5.17E+09	3.75E+12	11.8
-250.00	1.00	2.71E+12	0.1400	0	1.00	5.17E+09	3.75E+12	11.8
-250.00	1.00	2.71E+12	0.1400	0	1.00	5.17E+09	3.75E+12	11.8
-250.00	1.00	2.71E+12	0.1400	0	1.00	5.17E+09	3.75E+12	11.8
-250.00	1.00	2.71E+12	0.1400	0	1.00	5.17E+09	3.75E+12	11.8
-250.00	1.00	2.71E+12	0.1400	0	1.00	5.17E+09	3.75E+12	11.8
-250.00	1.00	2.71E+12	0.1400	0	1.00	5.17E+09	3.75E+12	11.8
-250.00	1.00	2.71E+12	0.1400	0	1.00	5.17E+09	3.75E+12	11.8
-250.00	1.00	2.71E+12	0.1400	0	1.00	5.17E+09	3.75E+12	11.8
-250.00	1.00	2.71E+12	0.1400	0	1.00	5.17E+09	3.75E+12	11.8
-250.00	1.00	2.71E+12	0.1400	0	1.00	5.17E+09	3.75E+12	11.8
-250.00	1.00	2.71E+12	0.1400	0	1.00	5.17E+09	3.75E+12	11.8
-250.00	1.00	2.71E+12	0.1400	0	1.00	5.17E+09	3.75E+12	11.8
-250.00	1.00	2.71E+12	0.1400	0	1.00	5.17E+09	3.75E+12	11.8
-250.00	1.00	2.71E+12	0.1400	0	1.00	5.17E+09	3.75E+12	11.8
-250.00	1.00	2.71E+12	0.1400	0	1.00	5.17E+09	3.75E+12	11.8
-250.00	1.00	2.71E+12	0.1400	0	1.00	5.17E+09	3.75E+12	11.8
-250.00	1.00	2.71E+12	0.1399	0	1.00	5.17E+09	3.75E+12	11.8
-250.00	1.00	2.71E+12	0.1399	0	1.00	5.17E+09	3.75E+12	11.8
-250.00	1.00	2.71E+12	0.1395	0	1.00	5.18E+09	3.75E+12	11.8
-250.00	1.00	2.71E+12	0.1391	0	1.00	5.18E+09	3.75E+12	11.8
-250.00	1.00	2.71E+12	0.1387	0	1.00	5.18E+09	3.74E+12	11.8
-250.00	1.00	2.71E+12	0.1383	0	1.00	5.18E+09	3.74E+12	11.8
-250.00	1.00	2.72E+12	0.1345	0	1.00	5.19E+09	3.73E+12	11.8
-250.00	1.00	2.72E+12	0.1307	0	0.99	5.20E+09	3.71E+12	11.8
-250.00	1.00	2.73E+12	0.1271	0	0.99	5.21E+09	3.69E+12	11.8
-250.00	1.00	2.73E+12	0.1235	0	0.99	5.22E+09	3.68E+12	11.8
-250.00	1.01	2.76E+12	0.1007	0	0.98	5.28E+09	3.57E+12	11.8
-249.99	1.02	2.78E+12	0.0823	0	0.96	5.30E+09	3.46E+12	11.8
-249.99	1.03	2.81E+12	0.0677	0	0.95	5.31E+09	3.36E+12	11.9
-249.99	1.04	2.84E+12	0.0559	0	0.93	5.30E+09	3.26E+12	11.9
-249.99	1.04	2.87E+12	0.0466	0	0.92	5.28E+09	3.17E+12	11.9
-249.98	1.06	2.92E+12	0.0340	0	0.90	5.22E+09	3.02E+12	12.0
-249.98	1.08	2.98E+12	0.0255	0	0.88	5.15E+09	2.89E+12	12.1
-249.97	1.09	3.03E+12	0.0196	0	0.86	5.08E+09	2.78E+12	12.1
-249.97	1.11	3.09E+12	0.0155	0	0.84	5.00E+09	2.67E+12	12.2
-249.96	1.12	3.15E+12	0.0126	0	0.83	4.93E+09	2.58E+12	12.3
-249.95	1.15	3.25E+12	0.0093	0	0.81	4.82E+09	2.46E+12	12.4
-249.94	1.18	3.35E+12	0.0072	0	0.79	4.73E+09	2.35E+12	12.5
-249.94	1.21	3.45E+12	0.0059	0	0.78	4.66E+09	2.27E+12	12.6
-249.93	1.24	3.54E+12	0.0050	0	0.77	4.60E+09	2.21E+12	12.7
-249.92	1.27	3.64E+12	0.0045	0	0.76	4.55E+09	2.17E+12	12.8
-249.90	1.32	3.81E+12	0.0039	0	0.75	4.49E+09	2.11E+12	13.0
-249.89	1.37	3.97E+12	0.0037	0	0.75	4.47E+09	2.09E+12	13.1
-249.88	1.42	4.13E+12	0.0036	0	0.74	4.46E+09	2.08E+12	13.3
-249.86	1.47	4.28E+12	0.0038	0	0.75	4.47E+09	2.09E+12	13.4
-249.85	1.51	4.42E+12	0.0040	0	0.75	4.49E+09	2.11E+12	13.5
-249.82	1.59	4.63E+12	0.0046	0	0.76	4.54E+09	2.16E+12	13.8
-249.80	1.66	4.83E+12	0.0055	0	0.77	4.60E+09	2.22E+12	13.9
-249.77	1.72	5.00E+12	0.0066	0	0.78	4.66E+09	2.29E+12	14.1
-249.75	1.78	5.16E+12	0.0079	0	0.79	4.73E+09	2.37E+12	14.3
-249.73	1.84	5.30E+12	0.0094	0	0.80	4.79E+09	2.43E+12	14.4
-249.68	1.94	5.52E+12	0.0126	0	0.82	4.89E+09	2.55E+12	14.6
-249.64	2.02	5.70E+12	0.0160	0	0.84	4.96E+09	2.65E+12	14.8
-249.59	2.10	5.84E+12	0.0194	0	0.85	5.02E+09	2.73E+12	15.0
-249.55	2.17	5.96E+12	0.0227	0	0.86	5.07E+09	2.80E+12	15.1
-249.50	2.23	6.06E+12	0.0257	0	0.87	5.10E+09	2.86E+12	15.2
-249.45	2.28	6.45E+12	0.0331	0	0.89	5.17E+09	2.98E+12	15.3
-249.43	2.30	6.47E+12	0.0363	0	0.90	5.19E+09	3.02E+12	15.4
-249.42	2.31	6.49E+12	0.0393	0	0.90	5.21E+09	3.06E+12	15.4
-249.40	2.32	6.51E+12	0.0418	0	0.91	5.22E+09	3.09E+12	15.4
-249.38	2.33	6.52E+12	0.0435	0	0.91	5.23E+09	3.11E+12	15.4
-249.37	2.34	6.53E+12	0.0454	0	0.91	5.24E+09	3.13E+12	15.4
-249.34	2.35	6.55E+12	0.0478	0	0.92	5.25E+09	3.16E+12	15.5
-249.31	2.37	6.57E+12	0.0495	0	0.92	5.25E+09	3.18E+12	15.5
-249.28	2.38	6.59E+12	0.0508	0	0.92	5.26E+09	3.19E+12	15.5
-249.25	2.39	6.60E+12	0.0518	0	0.92	5.27E+09	3.21E+12	15.5
-249.22	2.41	6.61E+12	0.0525	0	0.92	5.27E+09	3.22E+12	15.6
-249.16	2.43	6.64E+12	0.0533	0	0.93	5.28E+09	3.23E+12	15.6
-249.11	2.45	6.66E+12	0.0538	0	0.93	5.29E+09	3.24E+12	15.6
-249.05	2.47	6.67E+12	0.0540	0	0.93	5.30E+09	3.25E+12	15.7
-249.00	2.48	6.69E+12	0.0540	0	0.93	5.30E+09	3.25E+12	15.7
-248.94	2.50	6.70E+12	0.0539	0	0.93	5.31E+09	3.26E+12	15.7
-248.88	2.51	6.71E+12	0.0537	0	0.93	5.32E+09	3.26E+12	15.8
-248.82	2.53	6.73E+12	0.0534	0	0.94	5.33E+09	3.27E+12	15.8
-248.76	2.54	6.74E+12	0.0531	0	0.94	5.33E+09	3.27E+12	15.8
-248.70	2.56	6.75E+12	0.0526	0	0.94	5.34E+09	3.27E+12	15.8
-248.64	2.57	6.76E+12	0.0522	0	0.94	5.35E+09	3.27E+12	15.9
-248.54	2.59	6.77E+12	0.0514	0	0.94	5.36E+09	3.28E+12	15.9
-248.51	2.60	6.78E+12	0.0512	0	0.94	5.36E+09	3.28E+12	15.9
-248.48	2.60	7.21E+12	0.0509	0	0.94	5.37E+09	3.28E+12	15.9
-248.46	2.59	7.19E+12	0.0642	0	0.96	5.39E+09	3.39E+12	15.9
-248.44	2.59	7.18E+12	0.0708	0	0.96	5.39E+09	3.45E+12	15.9
-248.42	2.58	7.17E+12	0.0755	0	0.97	5.39E+09	3.48E+12	15.9
-248.40	2.58	7.16E+12	0.0783	0	0.97	5.39E+09	3.50E+12	15.9
-248.38	2.57	7.15E+12	0.0799	0	0.97	5.40E+09	3.52E+12	15.9
-248.36	2.57	7.13E+12	0.0808	0	0.98	5.40E+09	3.53E+12	15.9
-248.34	2.56	7.12E+12	0.0809	0	0.98	5.40E+09	3.53E+12	15.8

-248.32	2.55	7.11E+12	0.0805	0	0.98	5.41E+09	3.53E+12	15.8
-248.29	2.55	7.10E+12	0.0796	0	0.98	5.41E+09	3.53E+12	15.8
-248.27	2.55	7.09E+12	0.0785	0	0.98	5.42E+09	3.52E+12	15.8
-248.23	2.54	7.07E+12	0.0761	0	0.98	5.42E+09	3.51E+12	15.8
-248.19	2.53	7.05E+12	0.0736	0	0.97	5.43E+09	3.50E+12	15.8
-248.15	2.53	7.04E+12	0.0712	0	0.97	5.44E+09	3.49E+12	15.8
-248.12	2.53	7.03E+12	0.0690	0	0.97	5.45E+09	3.48E+12	15.8
-248.08	2.52	7.02E+12	0.0670	0	0.97	5.45E+09	3.47E+12	15.8
-248.04	2.52	7.01E+12	0.0652	0	0.97	5.46E+09	3.46E+12	15.8
-247.98	2.52	7.00E+12	0.0627	0	0.97	5.46E+09	3.45E+12	15.8
-247.91	2.53	6.99E+12	0.0608	0	0.97	5.47E+09	3.44E+12	15.8
-247.85	2.53	6.99E+12	0.0591	0	0.97	5.48E+09	3.43E+12	15.8
-247.79	2.53	6.98E+12	0.0576	0	0.97	5.48E+09	3.42E+12	15.8
-247.73	2.54	6.98E+12	0.0563	0	0.97	5.49E+09	3.42E+12	15.8
-247.63	2.55	6.98E+12	0.0547	0	0.97	5.50E+09	3.41E+12	15.8
-247.54	2.55	6.99E+12	0.0534	0	0.97	5.51E+09	3.41E+12	15.8
-247.51	2.56	6.99E+12	0.0530	0	0.97	5.51E+09	3.41E+12	15.9
-247.48	2.56	7.28E+12	0.0526	0	0.97	5.51E+09	3.40E+12	15.9
-247.46	2.55	7.27E+12	0.0625	0	0.98	5.52E+09	3.49E+12	15.8
-247.43	2.55	7.26E+12	0.0670	0	0.98	5.53E+09	3.53E+12	15.8
-247.41	2.54	7.25E+12	0.0697	0	0.99	5.53E+09	3.55E+12	15.8
-247.39	2.54	7.23E+12	0.0707	0	0.99	5.53E+09	3.56E+12	15.8
-247.36	2.54	7.22E+12	0.0710	0	0.99	5.54E+09	3.57E+12	15.8
-247.34	2.53	7.21E+12	0.0707	0	0.99	5.54E+09	3.57E+12	15.8
-247.31	2.53	7.20E+12	0.0701	0	0.99	5.54E+09	3.57E+12	15.8
-247.29	2.52	7.19E+12	0.0693	0	0.99	5.55E+09	3.56E+12	15.8
-247.26	2.52	7.18E+12	0.0682	0	0.99	5.55E+09	3.56E+12	15.8
-247.24	2.52	7.17E+12	0.0670	0	0.99	5.55E+09	3.55E+12	15.8
-247.19	2.51	7.15E+12	0.0652	0	0.99	5.56E+09	3.54E+12	15.8
-247.15	2.51	7.14E+12	0.0636	0	0.99	5.56E+09	3.53E+12	15.8
-247.11	2.51	7.14E+12	0.0620	0	0.99	5.57E+09	3.52E+12	15.8
-247.07	2.51	7.13E+12	0.0606	0	0.99	5.57E+09	3.52E+12	15.8
-247.03	2.51	7.12E+12	0.0594	0	0.99	5.58E+09	3.51E+12	15.8
-246.96	2.51	7.12E+12	0.0575	0	0.98	5.58E+09	3.50E+12	15.8
-246.88	2.52	7.11E+12	0.0561	0	0.98	5.59E+09	3.49E+12	15.8
-246.81	2.52	7.11E+12	0.0549	0	0.98	5.59E+09	3.49E+12	15.8
-246.74	2.52	7.11E+12	0.0538	0	0.98	5.60E+09	3.48E+12	15.8
-246.66	2.53	7.11E+12	0.0529	0	0.98	5.60E+09	3.48E+12	15.8
-246.55	2.54	7.12E+12	0.0518	0	0.98	5.61E+09	3.48E+12	15.8
-246.52	2.54	7.12E+12	0.0514	0	0.98	5.61E+09	3.48E+12	15.8
-246.48	2.54	7.24E+12	0.0511	0	0.98	5.62E+09	3.47E+12	15.8
-246.45	2.54	7.23E+12	0.0574	0	0.99	5.63E+09	3.53E+12	15.8
-246.41	2.54	7.22E+12	0.0575	0	0.99	5.63E+09	3.54E+12	15.8
-246.38	2.54	7.22E+12	0.0569	0	0.99	5.63E+09	3.54E+12	15.8
-246.34	2.54	7.21E+12	0.0567	0	0.99	5.64E+09	3.54E+12	15.8
-246.31	2.54	7.20E+12	0.0560	0	0.99	5.64E+09	3.53E+12	15.8
-246.27	2.54	7.20E+12	0.0557	0	0.99	5.64E+09	3.53E+12	15.8
-246.23	2.54	7.19E+12	0.0548	0	0.99	5.65E+09	3.53E+12	15.8
-246.18	2.54	7.19E+12	0.0539	0	0.99	5.65E+09	3.52E+12	15.8
-246.14	2.54	7.19E+12	0.0532	0	0.99	5.65E+09	3.52E+12	15.8
-246.09	2.54	7.19E+12	0.0526	0	0.99	5.65E+09	3.52E+12	15.8
-246.02	2.54	7.18E+12	0.0516	0	0.99	5.66E+09	3.51E+12	15.8
-245.95	2.55	7.18E+12	0.0508	0	0.99	5.66E+09	3.51E+12	15.9
-245.87	2.55	7.18E+12	0.0501	0	0.99	5.67E+09	3.51E+12	15.9
-245.80	2.56	7.19E+12	0.0494	0	0.99	5.67E+09	3.50E+12	15.9
-245.69	2.56	7.19E+12	0.0485	0	0.99	5.68E+09	3.50E+12	15.9
-245.57	2.57	7.19E+12	0.0477	0	0.99	5.68E+09	3.50E+12	15.9
-245.45	2.58	7.18E+12	0.0470	0	0.99	5.69E+09	3.50E+12	15.9
-245.34	2.58	7.16E+12	0.0436	0	0.99	5.69E+09	3.47E+12	15.9
-245.22	2.58	7.17E+12	0.0436	0	0.99	5.70E+09	3.47E+12	15.9
-245.10	2.59	7.17E+12	0.0430	0	0.99	5.70E+09	3.47E+12	15.9
-244.99	2.60	7.18E+12	0.0424	0	0.99	5.71E+09	3.47E+12	15.9
-244.87	2.60	7.18E+12	0.0419	0	0.99	5.71E+09	3.47E+12	16.0
-244.75	2.61	7.18E+12	0.0413	0	0.99	5.72E+09	3.47E+12	16.0
-244.63	2.62	7.19E+12	0.0408	0	0.99	5.72E+09	3.47E+12	16.0
-244.51	2.62	7.19E+12	0.0403	0	0.99	5.73E+09	3.47E+12	16.0
-244.39	2.63	7.10E+12	0.0372	0	0.99	5.72E+09	3.44E+12	16.0
-244.29	2.65	7.12E+12	0.0353	0	0.99	5.72E+09	3.41E+12	16.0
-244.21	2.66	7.13E+12	0.0351	0	0.99	5.72E+09	3.41E+12	16.1
-244.13	2.67	7.15E+12	0.0354	0	0.99	5.73E+09	3.42E+12	16.1
-244.05	2.68	7.16E+12	0.0356	0	0.99	5.73E+09	3.43E+12	16.1
-243.97	2.68	7.17E+12	0.0356	0	0.99	5.73E+09	3.43E+12	16.1
-243.89	2.69	7.17E+12	0.0355	0	0.99	5.74E+09	3.43E+12	16.1
-243.81	2.69	7.18E+12	0.0354	0	0.99	5.74E+09	3.43E+12	16.1
-243.72	2.70	7.18E+12	0.0354	0	0.99	5.75E+09	3.44E+12	16.1
-243.64	2.70	7.19E+12	0.0351	0	0.99	5.75E+09	3.44E+12	16.1
-243.56	2.71	7.19E+12	0.0349	0	0.99	5.75E+09	3.44E+12	16.2
-243.47	2.72	7.04E+12	0.0318	0	0.98	5.74E+09	3.39E+12	16.2
-243.41	2.73	7.06E+12	0.0295	0	0.98	5.72E+09	3.36E+12	16.2
-243.34	2.75	7.08E+12	0.0288	0	0.98	5.72E+09	3.35E+12	16.2
-243.28	2.76	7.10E+12	0.0292	0	0.98	5.73E+09	3.36E+12	16.2
-243.21	2.77	7.12E+12	0.0297	0	0.98	5.73E+09	3.37E+12	16.2
-243.14	2.78	7.13E+12	0.0299	0	0.98	5.74E+09	3.37E+12	16.3
-243.08	2.78	7.14E+12	0.0300	0	0.98	5.74E+09	3.38E+12	16.3
-242.99	2.79	7.15E+12	0.0300	0	0.98	5.74E+09	3.38E+12	16.3
-242.91	2.80	7.16E+12	0.0300	0	0.99	5.75E+09	3.39E+12	16.3
-242.82	2.81	7.17E+12	0.0300	0	0.99	5.75E+09	3.39E+12	16.3
-242.73	2.81	7.17E+12	0.0300	0	0.99	5.76E+09	3.39E+12	16.3
-242.65	2.82	7.18E+12	0.0298	0	0.99	5.76E+09	3.39E+12	16.3
-242.55	2.82	7.18E+12	0.0297	0	0.99	5.76E+09	3.39E+12	16.3
-242.52	2.82	7.18E+12	0.0296	0	0.99	5.76E+09	3.39E+12	16.3
-242.48	2.83	7.01E+12	0.0279	0	0.98	5.75E+09	3.36E+12	16.4
-242.45	2.83	7.02E+12	0.0262	0	0.98	5.74E+09	3.34E+12	16.4
-242.42	2.84	7.03E+12	0.0251	0	0.98	5.73E+09	3.32E+12	16.4
-242.38	2.85	7.05E+12	0.0245	0	0.98	5.72E+09	3.31E+12	16.4
-242.33	2.86	7.06E+12	0.0245	0	0.98	5.73E+09	3.31E+12	16.4
-242.29	2.87	7.07E+12	0.0246	0	0.98	5.73E+09	3.31E+12	16.4
-242.25	2.88	7.08E+12	0.0247	0	0.98	5.73E+09	3.32E+12	16.4
-242.18	2.89	7.10E+12	0.0250	0	0.98	5.74E+09	3.32E+12	16.4
-242.12	2.89	7.11E+12	0.0252	0	0.98	5.74E+09	3.33E+12	16.5

-242.05	2.90	7.12E+12	0.0253	0	0.98	5.75E+09	3.33E+12	16.5
-241.98	2.91	7.13E+12	0.0254	0	0.98	5.75E+09	3.34E+12	16.5
-241.88	2.92	7.14E+12	0.0255	0	0.98	5.75E+09	3.34E+12	16.5
-241.78	2.93	7.15E+12	0.0255	0	0.98	5.76E+09	3.34E+12	16.5
-241.68	2.93	7.16E+12	0.0254	0	0.98	5.76E+09	3.35E+12	16.5
-241.58	2.94	7.16E+12	0.0253	0	0.98	5.77E+09	3.35E+12	16.5
-241.45	2.95	7.02E+12	0.0228	0	0.98	5.74E+09	3.30E+12	16.5
-241.36	2.96	7.04E+12	0.0212	0	0.97	5.73E+09	3.27E+12	16.6
-241.26	2.97	7.05E+12	0.0210	0	0.97	5.73E+09	3.27E+12	16.6
-241.17	2.98	7.07E+12	0.0213	0	0.97	5.74E+09	3.28E+12	16.6
-241.07	2.99	7.08E+12	0.0215	0	0.98	5.74E+09	3.29E+12	16.6
-240.97	3.00	7.09E+12	0.0215	0	0.98	5.75E+09	3.29E+12	16.6
-240.88	3.00	7.09E+12	0.0214	0	0.98	5.75E+09	3.29E+12	16.6
-240.77	3.01	7.10E+12	0.0213	0	0.98	5.75E+09	3.29E+12	16.6
-240.67	3.01	7.10E+12	0.0214	0	0.98	5.76E+09	3.29E+12	16.7
-240.57	3.02	7.11E+12	0.0212	0	0.98	5.76E+09	3.29E+12	16.7
-240.47	3.03	7.04E+12	0.0202	0	0.97	5.75E+09	3.27E+12	16.7
-240.37	3.04	7.05E+12	0.0194	0	0.97	5.74E+09	3.26E+12	16.7
-240.27	3.05	7.07E+12	0.0193	0	0.97	5.74E+09	3.26E+12	16.7
-240.17	3.06	7.08E+12	0.0195	0	0.97	5.75E+09	3.26E+12	16.7
-240.06	3.06	7.09E+12	0.0196	0	0.98	5.75E+09	3.27E+12	16.7
-239.95	3.07	7.09E+12	0.0195	0	0.98	5.76E+09	3.27E+12	16.7
-239.84	3.08	7.10E+12	0.0194	0	0.98	5.76E+09	3.27E+12	16.7
-239.72	3.08	7.10E+12	0.0193	0	0.98	5.76E+09	3.27E+12	16.8
-239.61	3.09	7.11E+12	0.0193	0	0.98	5.77E+09	3.27E+12	16.8
-239.44	3.09	7.16E+12	0.0199	0	0.98	5.78E+09	3.29E+12	16.8
-239.27	3.08	7.15E+12	0.0201	0	0.98	5.79E+09	3.30E+12	16.8
-239.10	3.08	7.15E+12	0.0197	0	0.98	5.79E+09	3.30E+12	16.8
-238.92	3.08	7.14E+12	0.0192	0	0.98	5.79E+09	3.29E+12	16.8
-238.75	3.08	7.14E+12	0.0190	0	0.98	5.79E+09	3.29E+12	16.8
-238.58	3.09	7.15E+12	0.0188	0	0.98	5.79E+09	3.29E+12	16.8
-238.43	3.08	7.34E+12	0.0215	0	0.99	5.84E+09	3.35E+12	16.8
-238.39	3.07	7.32E+12	0.0225	0	0.99	5.85E+09	3.38E+12	16.8
-238.35	3.06	7.31E+12	0.0229	0	1.00	5.86E+09	3.39E+12	16.7
-238.30	3.05	7.30E+12	0.0227	0	1.00	5.86E+09	3.38E+12	16.7
-238.26	3.04	7.28E+12	0.0224	0	1.00	5.85E+09	3.38E+12	16.7
-238.21	3.04	7.27E+12	0.0221	0	0.99	5.85E+09	3.37E+12	16.7
-238.15	3.03	7.26E+12	0.0218	0	0.99	5.85E+09	3.37E+12	16.7
-238.09	3.03	7.25E+12	0.0215	0	0.99	5.85E+09	3.36E+12	16.7
-238.03	3.02	7.24E+12	0.0212	0	0.99	5.85E+09	3.36E+12	16.7
-237.97	3.02	7.24E+12	0.0210	0	0.99	5.85E+09	3.35E+12	16.7
-237.86	3.02	7.23E+12	0.0206	0	0.99	5.84E+09	3.35E+12	16.7
-237.76	3.01	7.22E+12	0.0204	0	0.99	5.84E+09	3.34E+12	16.7
-237.65	3.01	7.22E+12	0.0202	0	0.99	5.84E+09	3.34E+12	16.7
-237.54	3.01	7.22E+12	0.0200	0	0.99	5.84E+09	3.34E+12	16.7
-237.48	3.00	7.49E+12	0.0225	0	1.00	5.88E+09	3.40E+12	16.7
-237.44	2.99	7.47E+12	0.0252	0	1.01	5.91E+09	3.45E+12	16.7
-237.39	2.98	7.44E+12	0.0264	0	1.01	5.92E+09	3.47E+12	16.6
-237.35	2.97	7.42E+12	0.0263	0	1.01	5.92E+09	3.47E+12	16.6
-237.30	2.96	7.40E+12	0.0258	0	1.01	5.92E+09	3.46E+12	16.6
-237.26	2.95	7.38E+12	0.0252	0	1.01	5.91E+09	3.45E+12	16.6
-237.20	2.94	7.37E+12	0.0247	0	1.01	5.91E+09	3.45E+12	16.6
-237.15	2.93	7.35E+12	0.0243	0	1.01	5.91E+09	3.44E+12	16.6
-237.10	2.92	7.34E+12	0.0239	0	1.01	5.91E+09	3.43E+12	16.6
-237.04	2.92	7.33E+12	0.0236	0	1.00	5.90E+09	3.43E+12	16.6
-236.96	2.91	7.32E+12	0.0232	0	1.00	5.90E+09	3.42E+12	16.6
-236.87	2.91	7.31E+12	0.0229	0	1.00	5.90E+09	3.41E+12	16.5
-236.78	2.91	7.30E+12	0.0226	0	1.00	5.90E+09	3.41E+12	16.5
-236.70	2.90	7.30E+12	0.0224	0	1.00	5.90E+09	3.41E+12	16.5
-236.56	2.90	7.29E+12	0.0222	0	1.00	5.90E+09	3.40E+12	16.5
-236.45	2.89	7.46E+12	0.0246	0	1.01	5.92E+09	3.45E+12	16.5
-236.41	2.88	7.45E+12	0.0257	0	1.01	5.94E+09	3.47E+12	16.5
-236.38	2.88	7.44E+12	0.0263	0	1.01	5.94E+09	3.49E+12	16.5
-236.35	2.87	7.42E+12	0.0263	0	1.01	5.94E+09	3.49E+12	16.5
-236.31	2.87	7.41E+12	0.0260	0	1.01	5.94E+09	3.48E+12	16.5
-236.28	2.86	7.40E+12	0.0260	0	1.01	5.94E+09	3.48E+12	16.5
-236.22	2.86	7.39E+12	0.0256	0	1.01	5.94E+09	3.48E+12	16.5
-236.16	2.85	7.38E+12	0.0252	0	1.01	5.93E+09	3.47E+12	16.5
-236.11	2.85	7.37E+12	0.0249	0	1.01	5.93E+09	3.46E+12	16.5
-236.05	2.84	7.36E+12	0.0247	0	1.01	5.93E+09	3.46E+12	16.5
-235.96	2.84	7.35E+12	0.0244	0	1.01	5.93E+09	3.46E+12	16.5
-235.88	2.83	7.35E+12	0.0242	0	1.01	5.93E+09	3.45E+12	16.4
-235.79	2.83	7.34E+12	0.0240	0	1.01	5.93E+09	3.45E+12	16.4
-235.70	2.83	7.34E+12	0.0239	0	1.01	5.93E+09	3.45E+12	16.4
-235.56	2.83	7.34E+12	0.0237	0	1.01	5.93E+09	3.45E+12	16.4
-235.51	2.83	7.34E+12	0.0237	0	1.01	5.93E+09	3.44E+12	16.4
-235.46	2.83	7.45E+12	0.0236	0	1.01	5.93E+09	3.44E+12	16.4
-235.41	2.83	7.45E+12	0.0277	0	1.02	5.96E+09	3.52E+12	16.4
-235.36	2.82	7.44E+12	0.0273	0	1.02	5.96E+09	3.51E+12	16.4
-235.30	2.82	7.44E+12	0.0269	0	1.02	5.96E+09	3.51E+12	16.4
-235.25	2.82	7.43E+12	0.0268	0	1.02	5.96E+09	3.50E+12	16.4
-235.20	2.81	7.43E+12	0.0265	0	1.02	5.96E+09	3.50E+12	16.4
-235.14	2.81	7.42E+12	0.0264	0	1.02	5.96E+09	3.50E+12	16.4
-235.07	2.81	7.42E+12	0.0263	0	1.02	5.96E+09	3.50E+12	16.4
-235.00	2.81	7.42E+12	0.0262	0	1.02	5.96E+09	3.50E+12	16.4
-234.93	2.81	7.42E+12	0.0261	0	1.02	5.96E+09	3.49E+12	16.4
-234.85	2.81	7.41E+12	0.0260	0	1.02	5.96E+09	3.49E+12	16.4
-234.71	2.81	7.41E+12	0.0259	0	1.02	5.96E+09	3.49E+12	16.4
-234.57	2.81	7.41E+12	0.0258	0	1.02	5.96E+09	3.49E+12	16.4
-234.42	2.81	7.44E+12	0.0258	0	1.02	5.96E+09	3.49E+12	16.4
-234.28	2.80	7.43E+12	0.0272	0	1.02	5.97E+09	3.52E+12	16.4
-234.13	2.80	7.42E+12	0.0262	0	1.02	5.96E+09	3.50E+12	16.4
-233.98	2.80	7.42E+12	0.0260	0	1.02	5.96E+09	3.50E+12	16.4
-233.83	2.80	7.42E+12	0.0259	0	1.02	5.96E+09	3.50E+12	16.4
-233.68	2.80	7.42E+12	0.0259	0	1.02	5.96E+09	3.50E+12	16.4
-233.51	2.79	7.42E+12	0.0258	0	1.02	5.96E+09	3.50E+12	16.4
-233.38	2.80	7.37E+12	0.0247	0	1.01	5.95E+09	3.48E+12	16.4
-233.24	2.80	7.38E+12	0.0242	0	1.01	5.95E+09	3.47E+12	16.4
-233.11	2.81	7.39E+12	0.0243	0	1.01	5.95E+09	3.47E+12	16.4
-232.97	2.81	7.40E+12	0.0245	0	1.01	5.95E+09	3.47E+12	16.5

-232.81	2.82	7.40E+12	0.0246	0	1.01	5.96E+09	3.48E+12	16.5
-232.65	2.82	7.40E+12	0.0247	0	1.02	5.96E+09	3.48E+12	16.5
-232.55	2.82	7.41E+12	0.0246	0	1.02	5.96E+09	3.48E+12	16.5
-232.48	2.82	7.25E+12	0.0226	0	1.01	5.94E+09	3.44E+12	16.5
-232.43	2.83	7.27E+12	0.0214	0	1.01	5.92E+09	3.41E+12	16.5
-232.37	2.84	7.28E+12	0.0209	0	1.00	5.92E+09	3.40E+12	16.5
-232.32	2.85	7.30E+12	0.0210	0	1.00	5.92E+09	3.40E+12	16.5
-232.26	2.85	7.31E+12	0.0212	0	1.01	5.92E+09	3.41E+12	16.5
-232.19	2.86	7.32E+12	0.0215	0	1.01	5.92E+09	3.42E+12	16.5
-232.12	2.86	7.33E+12	0.0218	0	1.01	5.93E+09	3.42E+12	16.5
-232.05	2.87	7.34E+12	0.0219	0	1.01	5.93E+09	3.43E+12	16.5
-231.99	2.87	7.35E+12	0.0221	0	1.01	5.93E+09	3.43E+12	16.6
-231.88	2.88	7.36E+12	0.0222	0	1.01	5.93E+09	3.43E+12	16.6
-231.77	2.88	7.36E+12	0.0223	0	1.01	5.94E+09	3.43E+12	16.6
-231.66	2.88	7.36E+12	0.0223	0	1.01	5.94E+09	3.44E+12	16.6
-231.56	2.88	7.37E+12	0.0223	0	1.01	5.94E+09	3.44E+12	16.6
-231.49	2.89	7.12E+12	0.0199	0	1.00	5.91E+09	3.38E+12	16.6
-231.45	2.90	7.14E+12	0.0179	0	0.99	5.88E+09	3.34E+12	16.6
-231.40	2.91	7.17E+12	0.0171	0	0.99	5.86E+09	3.32E+12	16.6
-231.35	2.92	7.19E+12	0.0172	0	0.99	5.87E+09	3.32E+12	16.6
-231.30	2.93	7.21E+12	0.0176	0	0.99	5.87E+09	3.33E+12	16.7
-231.26	2.94	7.22E+12	0.0179	0	0.99	5.88E+09	3.34E+12	16.7
-231.19	2.95	7.24E+12	0.0182	0	1.00	5.88E+09	3.34E+12	16.7
-231.13	2.96	7.25E+12	0.0183	0	1.00	5.89E+09	3.35E+12	16.7
-231.07	2.97	7.26E+12	0.0186	0	1.00	5.89E+09	3.35E+12	16.7
-231.01	2.97	7.27E+12	0.0187	0	1.00	5.89E+09	3.36E+12	16.7
-230.91	2.98	7.28E+12	0.0190	0	1.00	5.90E+09	3.36E+12	16.7
-230.80	2.98	7.29E+12	0.0191	0	1.00	5.90E+09	3.37E+12	16.7
-230.70	2.99	7.30E+12	0.0191	0	1.00	5.90E+09	3.37E+12	16.7
-230.60	2.99	7.30E+12	0.0192	0	1.00	5.90E+09	3.37E+12	16.7
-230.50	2.99	7.31E+12	0.0192	0	1.00	5.91E+09	3.37E+12	16.8
-230.49	3.00	7.01E+12	0.0182	0	1.00	5.89E+09	3.35E+12	16.8
-230.47	3.00	7.02E+12	0.0163	0	0.99	5.86E+09	3.30E+12	16.8
-230.46	3.00	7.02E+12	0.0156	0	0.99	5.85E+09	3.28E+12	16.8
-230.44	3.01	7.03E+12	0.0152	0	0.99	5.84E+09	3.27E+12	16.8
-230.41	3.02	7.05E+12	0.0145	0	0.98	5.82E+09	3.25E+12	16.8
-230.38	3.03	7.06E+12	0.0142	0	0.98	5.82E+09	3.24E+12	16.8
-230.35	3.04	7.08E+12	0.0142	0	0.98	5.82E+09	3.24E+12	16.8
-230.31	3.05	7.10E+12	0.0143	0	0.98	5.82E+09	3.24E+12	16.8
-230.26	3.06	7.12E+12	0.0145	0	0.98	5.82E+09	3.25E+12	16.9
-230.21	3.07	7.14E+12	0.0148	0	0.98	5.83E+09	3.26E+12	16.9
-230.15	3.08	7.16E+12	0.0150	0	0.98	5.84E+09	3.27E+12	16.9
-230.10	3.09	7.17E+12	0.0152	0	0.99	5.84E+09	3.27E+12	16.9
-230.00	3.11	7.19E+12	0.0155	0	0.99	5.85E+09	3.28E+12	16.9
-229.91	3.12	7.21E+12	0.0157	0	0.99	5.85E+09	3.29E+12	16.9
-229.81	3.12	7.22E+12	0.0159	0	0.99	5.86E+09	3.29E+12	16.9
-229.72	3.13	7.23E+12	0.0160	0	0.99	5.86E+09	3.30E+12	17.0
-229.62	3.13	7.23E+12	0.0160	0	0.99	5.86E+09	3.30E+12	17.0
-229.45	3.14	7.21E+12	0.0156	0	0.99	5.86E+09	3.29E+12	17.0
-229.27	3.15	7.22E+12	0.0154	0	0.99	5.86E+09	3.29E+12	17.0
-229.10	3.16	7.23E+12	0.0155	0	0.99	5.86E+09	3.29E+12	17.0
-228.92	3.17	7.24E+12	0.0156	0	0.99	5.87E+09	3.30E+12	17.0
-228.74	3.17	7.24E+12	0.0155	0	0.99	5.87E+09	3.30E+12	17.0
-228.55	3.17	7.25E+12	0.0154	0	0.99	5.87E+09	3.30E+12	17.0
-228.36	3.18	7.22E+12	0.0149	0	0.99	5.87E+09	3.29E+12	17.0
-228.17	3.19	7.23E+12	0.0147	0	0.99	5.87E+09	3.28E+12	17.1
-227.98	3.20	7.24E+12	0.0147	0	0.99	5.87E+09	3.28E+12	17.1
-227.79	3.20	7.24E+12	0.0148	0	0.99	5.87E+09	3.29E+12	17.1
-227.57	3.20	7.25E+12	0.0147	0	0.99	5.88E+09	3.29E+12	17.1
-227.35	3.22	7.24E+12	0.0144	0	0.99	5.88E+09	3.28E+12	17.1
-227.13	3.23	7.26E+12	0.0145	0	0.99	5.88E+09	3.29E+12	17.1
-226.91	3.25	7.28E+12	0.0146	0	0.99	5.89E+09	3.29E+12	17.2
-226.64	3.26	7.29E+12	0.0146	0	0.99	5.89E+09	3.30E+12	17.2
-226.37	3.26	7.27E+12	0.0143	0	0.99	5.89E+09	3.29E+12	17.2
-226.10	3.27	7.29E+12	0.0141	0	0.99	5.89E+09	3.29E+12	17.2
-225.82	3.28	7.29E+12	0.0141	0	0.99	5.90E+09	3.29E+12	17.2
-225.53	3.28	7.30E+12	0.0140	0	0.99	5.90E+09	3.29E+12	17.2
-225.24	3.29	7.29E+12	0.0137	0	0.99	5.90E+09	3.29E+12	17.2
-224.95	3.30	7.30E+12	0.0136	0	0.99	5.90E+09	3.29E+12	17.2
-224.66	3.30	7.30E+12	0.0135	0	0.99	5.90E+09	3.29E+12	17.3
-224.28	3.31	7.29E+12	0.0132	0	0.99	5.90E+09	3.28E+12	17.3
-223.91	3.32	7.30E+12	0.0131	0	0.99	5.90E+09	3.28E+12	17.3
-223.53	3.32	7.31E+12	0.0130	0	1.00	5.91E+09	3.28E+12	17.3
-223.15	3.33	7.29E+12	0.0127	0	0.99	5.91E+09	3.28E+12	17.3
-222.74	3.33	7.30E+12	0.0126	0	0.99	5.91E+09	3.28E+12	17.3
-222.33	3.35	7.32E+12	0.0126	0	1.00	5.92E+09	3.28E+12	17.3
-221.92	3.37	7.35E+12	0.0127	0	1.00	5.92E+09	3.29E+12	17.4
-221.51	3.38	7.36E+12	0.0127	0	1.00	5.93E+09	3.29E+12	17.4
-221.03	3.39	7.36E+12	0.0125	0	1.00	5.93E+09	3.29E+12	17.4
-220.56	3.39	7.36E+12	0.0124	0	1.00	5.94E+09	3.29E+12	17.4
-220.08	3.40	7.36E+12	0.0121	0	1.00	5.94E+09	3.29E+12	17.4
-219.61	3.40	7.37E+12	0.0120	0	1.00	5.94E+09	3.29E+12	17.4
-219.01	3.40	7.37E+12	0.0119	0	1.00	5.94E+09	3.29E+12	17.5
-218.41	3.40	7.37E+12	0.0118	0	1.00	5.95E+09	3.29E+12	17.5
-217.80	3.41	7.38E+12	0.0116	0	1.00	5.95E+09	3.29E+12	17.5
-217.20	3.42	7.40E+12	0.0117	0	1.00	5.96E+09	3.30E+12	17.5
-216.58	3.44	7.43E+12	0.0118	0	1.00	5.97E+09	3.30E+12	17.5
-215.95	3.44	7.44E+12	0.0118	0	1.01	5.98E+09	3.31E+12	17.5
-215.32	3.43	7.45E+12	0.0119	0	1.01	5.98E+09	3.32E+12	17.5
-214.70	3.43	7.45E+12	0.0117	0	1.01	5.99E+09	3.31E+12	17.5
-213.99	3.42	7.45E+12	0.0116	0	1.01	5.99E+09	3.31E+12	17.5
-213.28	3.42	7.46E+12	0.0116	0	1.01	6.00E+09	3.32E+12	17.5
-212.57	3.41	7.46E+12	0.0115	0	1.01	6.00E+09	3.32E+12	17.5
-211.86	3.42	7.49E+12	0.0117	0	1.01	6.01E+09	3.33E+12	17.6
-211.15	3.42	7.52E+12	0.0120	0	1.01	6.02E+09	3.34E+12	17.6
-210.44	3.42	7.53E+12	0.0120	0	1.01	6.02E+09	3.35E+12	17.6
-209.73	3.41	7.52E+12	0.0118	0	1.01	6.03E+09	3.34E+12	17.6
-208.95	3.41	7.52E+12	0.0117	0	1.01	6.03E+09	3.34E+12	17.6
-208.18	3.40	7.49E+12	0.0112	0	1.01	6.02E+09	3.32E+12	17.6
-207.40	3.39	7.46E+12	0.0108	0	1.01	6.01E+09	3.31E+12	17.6

-206.76	3.39	7.47E+12	0.0108	0	1.01	6.01E+09	3.31E+12	17.6
-206.25	3.38	7.43E+12	0.0104	0	1.01	6.00E+09	3.29E+12	17.6
-205.74	3.37	7.42E+12	0.0102	0	1.01	5.99E+09	3.29E+12	17.6
-205.23	3.38	7.38E+12	0.0098	0	1.00	5.98E+09	3.27E+12	17.6
-204.73	3.38	7.40E+12	0.0099	0	1.00	5.98E+09	3.27E+12	17.6
-204.22	3.40	7.36E+12	0.0095	0	1.00	5.97E+09	3.26E+12	17.6
-203.71	3.41	7.38E+12	0.0095	0	1.00	5.97E+09	3.26E+12	17.7
-203.51	3.41	7.39E+12	0.0096	0	1.00	5.97E+09	3.26E+12	17.7
-203.31	3.41	7.32E+12	0.0091	0	1.00	5.95E+09	3.24E+12	17.7
-203.14	3.41	7.32E+12	0.0089	0	1.00	5.95E+09	3.23E+12	17.7
-202.98	3.41	7.32E+12	0.0089	0	1.00	5.94E+09	3.23E+12	17.7
-202.81	3.41	7.32E+12	0.0089	0	1.00	5.95E+09	3.23E+12	17.7
-202.58	3.41	7.32E+12	0.0089	0	1.00	5.95E+09	3.23E+12	17.7
-202.35	3.42	7.27E+12	0.0084	0	0.99	5.93E+09	3.21E+12	17.7
-202.16	3.43	7.28E+12	0.0083	0	0.99	5.92E+09	3.20E+12	17.7
-201.98	3.44	7.29E+12	0.0084	0	0.99	5.93E+09	3.21E+12	17.7
-201.80	3.44	7.30E+12	0.0085	0	0.99	5.93E+09	3.21E+12	17.7
-201.61	3.44	7.30E+12	0.0085	0	0.99	5.93E+09	3.21E+12	17.7
-201.54	3.44	7.31E+12	0.0085	0	0.99	5.93E+09	3.21E+12	17.7
-201.52	3.44	7.31E+12	0.0085	0	0.99	5.93E+09	3.21E+12	17.7
-201.50	3.44	7.21E+12	0.0085	0	0.99	5.93E+09	3.21E+12	17.7
-201.47	3.44	7.21E+12	0.0080	0	0.99	5.91E+09	3.18E+12	17.7
-201.41	3.44	7.21E+12	0.0078	0	0.99	5.90E+09	3.18E+12	17.7
-201.36	3.44	7.22E+12	0.0077	0	0.99	5.90E+09	3.17E+12	17.7
-201.30	3.45	7.22E+12	0.0077	0	0.99	5.90E+09	3.17E+12	17.7
-201.19	3.45	7.23E+12	0.0077	0	0.99	5.90E+09	3.17E+12	17.7
-201.09	3.45	7.23E+12	0.0078	0	0.99	5.90E+09	3.17E+12	17.7
-200.98	3.45	7.23E+12	0.0078	0	0.99	5.90E+09	3.17E+12	17.8
-200.79	3.45	7.24E+12	0.0078	0	0.99	5.90E+09	3.17E+12	17.8
-200.60	3.45	7.24E+12	0.0078	0	0.99	5.90E+09	3.18E+12	17.8
-200.53	3.45	7.24E+12	0.0078	0	0.99	5.90E+09	3.18E+12	17.8
-200.51	3.45	7.24E+12	0.0078	0	0.99	5.90E+09	3.18E+12	17.8
-200.49	3.45	7.14E+12	0.0078	0	0.99	5.90E+09	3.18E+12	17.8
-200.46	3.46	7.15E+12	0.0073	0	0.98	5.88E+09	3.15E+12	17.8
-200.41	3.46	7.16E+12	0.0072	0	0.98	5.88E+09	3.14E+12	17.8
-200.35	3.47	7.17E+12	0.0072	0	0.98	5.87E+09	3.14E+12	17.8
-200.30	3.47	7.17E+12	0.0072	0	0.98	5.87E+09	3.14E+12	17.8
-200.19	3.48	7.18E+12	0.0072	0	0.98	5.88E+09	3.14E+12	17.8
-200.08	3.48	7.19E+12	0.0073	0	0.98	5.88E+09	3.15E+12	17.8
-199.98	3.49	7.20E+12	0.0073	0	0.98	5.88E+09	3.15E+12	17.8
-199.78	3.49	7.21E+12	0.0074	0	0.99	5.89E+09	3.15E+12	17.8
-199.59	3.50	7.21E+12	0.0074	0	0.99	5.89E+09	3.15E+12	17.8
-199.52	3.50	7.21E+12	0.0074	0	0.99	5.89E+09	3.15E+12	17.8
-199.50	3.50	7.12E+12	0.0072	0	0.98	5.88E+09	3.14E+12	17.8
-199.48	3.50	7.12E+12	0.0069	0	0.98	5.87E+09	3.13E+12	17.8
-199.45	3.50	7.13E+12	0.0069	0	0.98	5.86E+09	3.12E+12	17.8
-199.39	3.51	7.14E+12	0.0068	0	0.98	5.86E+09	3.12E+12	17.8
-199.33	3.52	7.15E+12	0.0068	0	0.98	5.86E+09	3.12E+12	17.9
-199.27	3.52	7.15E+12	0.0068	0	0.98	5.86E+09	3.12E+12	17.9
-199.22	3.52	7.16E+12	0.0068	0	0.98	5.86E+09	3.12E+12	17.9
-199.09	3.53	7.17E+12	0.0069	0	0.98	5.87E+09	3.13E+12	17.9
-198.97	3.54	7.18E+12	0.0070	0	0.98	5.87E+09	3.13E+12	17.9
-198.84	3.54	7.19E+12	0.0070	0	0.98	5.87E+09	3.13E+12	17.9
-198.72	3.54	7.19E+12	0.0070	0	0.98	5.87E+09	3.13E+12	17.9
-198.56	3.54	7.19E+12	0.0070	0	0.98	5.87E+09	3.14E+12	17.9
-198.50	3.54	7.20E+12	0.0070	0	0.98	5.87E+09	3.14E+12	17.9
-198.46	3.55	7.10E+12	0.0067	0	0.98	5.86E+09	3.12E+12	17.9
-198.42	3.55	7.11E+12	0.0065	0	0.98	5.85E+09	3.10E+12	17.9
-198.38	3.56	7.12E+12	0.0065	0	0.98	5.84E+09	3.10E+12	17.9
-198.31	3.56	7.13E+12	0.0064	0	0.98	5.84E+09	3.10E+12	17.9
-198.24	3.57	7.14E+12	0.0065	0	0.98	5.84E+09	3.10E+12	17.9
-198.18	3.58	7.14E+12	0.0065	0	0.98	5.85E+09	3.10E+12	17.9
-198.11	3.58	7.15E+12	0.0066	0	0.98	5.85E+09	3.11E+12	17.9
-197.94	3.59	7.16E+12	0.0066	0	0.98	5.85E+09	3.11E+12	18.0
-197.76	3.59	7.17E+12	0.0066	0	0.98	5.86E+09	3.11E+12	18.0
-197.59	3.60	7.17E+12	0.0067	0	0.98	5.86E+09	3.12E+12	18.0
-197.41	3.61	7.09E+12	0.0062	0	0.98	5.84E+09	3.09E+12	18.0
-197.28	3.62	7.11E+12	0.0061	0	0.98	5.83E+09	3.08E+12	18.0
-197.14	3.63	7.13E+12	0.0060	0	0.97	5.83E+09	3.08E+12	18.0
-197.01	3.64	7.14E+12	0.0062	0	0.98	5.84E+09	3.09E+12	18.0
-196.84	3.64	7.14E+12	0.0062	0	0.98	5.84E+09	3.09E+12	18.0
-196.67	3.65	7.15E+12	0.0063	0	0.98	5.85E+09	3.09E+12	18.0
-196.50	3.65	7.16E+12	0.0063	0	0.98	5.85E+09	3.10E+12	18.1
-196.47	3.65	7.05E+12	0.0060	0	0.98	5.83E+09	3.08E+12	18.1
-196.45	3.66	7.05E+12	0.0058	0	0.97	5.82E+09	3.06E+12	18.1
-196.42	3.66	7.06E+12	0.0057	0	0.97	5.81E+09	3.06E+12	18.1
-196.37	3.67	7.07E+12	0.0056	0	0.97	5.81E+09	3.05E+12	18.1
-196.31	3.67	7.08E+12	0.0057	0	0.97	5.81E+09	3.06E+12	18.1
-196.26	3.68	7.09E+12	0.0057	0	0.97	5.82E+09	3.06E+12	18.1
-196.20	3.69	7.09E+12	0.0057	0	0.97	5.82E+09	3.06E+12	18.1
-196.09	3.70	7.11E+12	0.0058	0	0.97	5.82E+09	3.06E+12	18.1
-195.97	3.70	7.11E+12	0.0058	0	0.97	5.82E+09	3.07E+12	18.1
-195.85	3.71	7.12E+12	0.0058	0	0.97	5.83E+09	3.07E+12	18.1
-195.74	3.71	7.13E+12	0.0059	0	0.97	5.83E+09	3.07E+12	18.1
-195.60	3.71	7.13E+12	0.0059	0	0.97	5.83E+09	3.07E+12	18.1
-195.52	3.72	7.13E+12	0.0059	0	0.97	5.83E+09	3.07E+12	18.1
-195.51	3.72	7.13E+12	0.0059	0	0.97	5.83E+09	3.07E+12	18.1
-195.50	3.72	6.98E+12	0.0059	0	0.97	5.83E+09	3.07E+12	18.1
-195.49	3.72	6.98E+12	0.0056	0	0.97	5.81E+09	3.05E+12	18.1
-195.45	3.73	6.99E+12	0.0053	0	0.97	5.79E+09	3.03E+12	18.2
-195.42	3.73	7.00E+12	0.0051	0	0.97	5.78E+09	3.02E+12	18.2
-195.38	3.74	7.01E+12	0.0051	0	0.97	5.78E+09	3.01E+12	18.2
-195.33	3.75	7.02E+12	0.0051	0	0.97	5.78E+09	3.01E+12	18.2
-195.27	3.76	7.03E+12	0.0051	0	0.97	5.78E+09	3.02E+12	18.2
-195.22	3.77	7.04E+12	0.0051	0	0.97	5.78E+09	3.02E+12	18.2
-195.11	3.78	7.06E+12	0.0052	0	0.97	5.79E+09	3.02E+12	18.2
-195.01	3.79	7.07E+12	0.0053	0	0.97	5.80E+09	3.03E+12	18.2
-194.90	3.79	7.08E+12	0.0053	0	0.97	5.80E+09	3.03E+12	18.2
-194.80	3.80	7.09E+12	0.0053	0	0.97	5.80E+09	3.04E+12	18.2
-194.56	3.81	7.10E+12	0.0054	0	0.97	5.81E+09	3.04E+12	18.3

-194.33	3.82	6.97E+12	0.0048	0	0.96	5.77E+09	3.00E+12	18.3
-194.24	3.83	6.98E+12	0.0046	0	0.96	5.76E+09	2.98E+12	18.3
-194.16	3.84	6.99E+12	0.0046	0	0.96	5.76E+09	2.98E+12	18.3
-194.08	3.85	7.00E+12	0.0047	0	0.96	5.76E+09	2.99E+12	18.3
-193.99	3.85	7.00E+12	0.0047	0	0.96	5.76E+09	2.99E+12	18.3
-193.91	3.86	7.01E+12	0.0047	0	0.96	5.77E+09	2.99E+12	18.3
-193.78	3.86	7.02E+12	0.0047	0	0.96	5.77E+09	2.99E+12	18.3
-193.64	3.87	7.02E+12	0.0047	0	0.96	5.77E+09	3.00E+12	18.3
-193.50	3.87	7.03E+12	0.0047	0	0.96	5.77E+09	3.00E+12	18.3
-193.39	3.88	6.90E+12	0.0043	0	0.96	5.74E+09	2.96E+12	18.4
-193.30	3.90	6.92E+12	0.0042	0	0.96	5.73E+09	2.95E+12	18.4
-193.20	3.91	6.94E+12	0.0042	0	0.96	5.73E+09	2.95E+12	18.4
-193.11	3.92	6.95E+12	0.0042	0	0.96	5.73E+09	2.95E+12	18.4
-192.98	3.93	6.97E+12	0.0043	0	0.96	5.74E+09	2.96E+12	18.4
-192.84	3.95	6.98E+12	0.0043	0	0.96	5.74E+09	2.96E+12	18.4
-192.71	3.95	6.99E+12	0.0043	0	0.96	5.75E+09	2.97E+12	18.4
-192.55	3.96	6.99E+12	0.0043	0	0.96	5.75E+09	2.97E+12	18.5
-192.51	3.96	7.00E+12	0.0043	0	0.96	5.75E+09	2.97E+12	18.5
-192.49	3.96	6.87E+12	0.0042	0	0.96	5.74E+09	2.96E+12	18.5
-192.47	3.96	6.87E+12	0.0041	0	0.96	5.73E+09	2.94E+12	18.5
-192.46	3.97	6.88E+12	0.0040	0	0.95	5.72E+09	2.93E+12	18.5
-192.42	3.97	6.88E+12	0.0039	0	0.95	5.71E+09	2.93E+12	18.5
-192.39	3.98	6.89E+12	0.0039	0	0.95	5.71E+09	2.92E+12	18.5
-192.36	3.99	6.90E+12	0.0038	0	0.95	5.71E+09	2.92E+12	18.5
-192.28	4.00	6.91E+12	0.0039	0	0.95	5.71E+09	2.92E+12	18.5
-192.21	4.01	6.92E+12	0.0039	0	0.95	5.71E+09	2.93E+12	18.5
-192.14	4.01	6.93E+12	0.0039	0	0.95	5.72E+09	2.93E+12	18.5
-191.99	4.03	6.95E+12	0.0040	0	0.96	5.72E+09	2.94E+12	18.5
-191.84	4.04	6.96E+12	0.0040	0	0.96	5.73E+09	2.94E+12	18.6
-191.69	4.05	6.97E+12	0.0040	0	0.96	5.73E+09	2.94E+12	18.6
-191.54	4.05	6.98E+12	0.0040	0	0.96	5.74E+09	2.95E+12	18.6
-191.43	4.07	6.86E+12	0.0037	0	0.95	5.71E+09	2.91E+12	18.6
-191.33	4.08	6.88E+12	0.0036	0	0.95	5.70E+09	2.90E+12	18.6
-191.23	4.10	6.89E+12	0.0036	0	0.95	5.70E+09	2.90E+12	18.6
-191.13	4.11	6.91E+12	0.0036	0	0.95	5.70E+09	2.90E+12	18.6
-190.98	4.12	6.92E+12	0.0036	0	0.95	5.71E+09	2.91E+12	18.7
-190.82	4.13	6.94E+12	0.0037	0	0.95	5.71E+09	2.91E+12	18.7
-190.67	4.14	6.95E+12	0.0037	0	0.95	5.72E+09	2.92E+12	18.7
-190.53	4.15	6.95E+12	0.0037	0	0.95	5.72E+09	2.92E+12	18.7
-190.51	4.15	6.95E+12	0.0037	0	0.95	5.72E+09	2.92E+12	18.7
-190.50	4.15	6.95E+12	0.0037	0	0.95	5.72E+09	2.92E+12	18.7
-190.49	4.15	6.70E+12	0.0037	0	0.95	5.72E+09	2.92E+12	18.7
-190.48	4.16	6.70E+12	0.0034	0	0.95	5.69E+09	2.88E+12	18.7
-190.46	4.16	6.71E+12	0.0031	0	0.94	5.66E+09	2.86E+12	18.7
-190.43	4.17	6.72E+12	0.0030	0	0.94	5.64E+09	2.84E+12	18.7
-190.40	4.18	6.73E+12	0.0029	0	0.94	5.63E+09	2.82E+12	18.7
-190.36	4.20	6.75E+12	0.0029	0	0.94	5.63E+09	2.82E+12	18.7
-190.32	4.21	6.76E+12	0.0029	0	0.94	5.63E+09	2.83E+12	18.8
-190.28	4.22	6.77E+12	0.0029	0	0.94	5.63E+09	2.83E+12	18.8
-190.24	4.23	6.79E+12	0.0029	0	0.94	5.64E+09	2.83E+12	18.8
-190.16	4.25	6.81E+12	0.0030	0	0.94	5.64E+09	2.84E+12	18.8
-190.07	4.27	6.83E+12	0.0031	0	0.94	5.65E+09	2.85E+12	18.8
-189.98	4.29	6.84E+12	0.0031	0	0.94	5.66E+09	2.85E+12	18.8
-189.90	4.30	6.86E+12	0.0031	0	0.94	5.66E+09	2.86E+12	18.8
-189.71	4.32	6.88E+12	0.0032	0	0.95	5.67E+09	2.86E+12	18.9
-189.53	4.34	6.89E+12	0.0032	0	0.95	5.68E+09	2.87E+12	18.9
-189.35	4.35	6.86E+12	0.0032	0	0.95	5.68E+09	2.87E+12	18.9
-189.17	4.39	6.91E+12	0.0031	0	0.95	5.68E+09	2.87E+12	18.9
-188.95	4.41	6.93E+12	0.0032	0	0.95	5.69E+09	2.88E+12	19.0
-188.72	4.42	6.94E+12	0.0032	0	0.95	5.69E+09	2.88E+12	19.0
-188.50	4.42	6.99E+12	0.0033	0	0.95	5.71E+09	2.90E+12	19.0
-188.27	4.42	6.99E+12	0.0033	0	0.95	5.72E+09	2.90E+12	19.0
-188.05	4.42	6.99E+12	0.0033	0	0.95	5.72E+09	2.90E+12	19.0
-187.77	4.42	6.99E+12	0.0032	0	0.95	5.72E+09	2.90E+12	19.0
-187.56	4.42	6.99E+12	0.0032	0	0.95	5.72E+09	2.90E+12	19.0
-187.48	4.42	7.05E+12	0.0033	0	0.96	5.74E+09	2.92E+12	19.0
-187.41	4.42	7.06E+12	0.0035	0	0.96	5.75E+09	2.93E+12	19.0
-187.33	4.42	7.06E+12	0.0035	0	0.96	5.75E+09	2.93E+12	19.0
-187.20	4.42	7.05E+12	0.0034	0	0.96	5.75E+09	2.93E+12	19.0
-187.08	4.42	7.05E+12	0.0034	0	0.96	5.75E+09	2.92E+12	19.0
-186.95	4.42	7.06E+12	0.0034	0	0.96	5.75E+09	2.93E+12	19.0
-186.83	4.42	7.06E+12	0.0034	0	0.96	5.75E+09	2.93E+12	19.0
-186.50	4.41	7.12E+12	0.0035	0	0.96	5.78E+09	2.95E+12	19.0
-186.24	4.39	7.10E+12	0.0035	0	0.96	5.78E+09	2.95E+12	19.0
-185.99	4.37	7.08E+12	0.0033	0	0.96	5.77E+09	2.94E+12	19.0
-185.73	4.37	7.08E+12	0.0033	0	0.96	5.77E+09	2.93E+12	19.0
-185.48	4.37	7.17E+12	0.0036	0	0.97	5.80E+09	2.97E+12	19.0
-185.29	4.36	7.16E+12	0.0036	0	0.97	5.81E+09	2.98E+12	19.0
-185.09	4.36	7.16E+12	0.0037	0	0.97	5.82E+09	2.98E+12	19.0
-184.90	4.35	7.15E+12	0.0036	0	0.97	5.81E+09	2.98E+12	19.0
-184.61	4.35	7.15E+12	0.0035	0	0.97	5.81E+09	2.97E+12	19.0
-184.53	4.35	7.15E+12	0.0035	0	0.97	5.81E+09	2.97E+12	19.0
-184.51	4.35	7.15E+12	0.0035	0	0.97	5.81E+09	2.97E+12	19.0
-184.48	4.35	7.29E+12	0.0035	0	0.97	5.81E+09	2.97E+12	19.0
-184.46	4.34	7.28E+12	0.0039	0	0.98	5.85E+09	3.01E+12	19.0
-184.41	4.34	7.27E+12	0.0039	0	0.98	5.85E+09	3.02E+12	19.0
-184.37	4.33	7.26E+12	0.0041	0	0.98	5.87E+09	3.03E+12	18.9
-184.32	4.32	7.26E+12	0.0040	0	0.98	5.86E+09	3.03E+12	18.9
-184.24	4.31	7.24E+12	0.0039	0	0.98	5.86E+09	3.02E+12	18.9
-184.16	4.30	7.23E+12	0.0039	0	0.98	5.85E+09	3.02E+12	18.9
-184.08	4.29	7.22E+12	0.0039	0	0.98	5.85E+09	3.01E+12	18.9
-183.90	4.28	7.21E+12	0.0038	0	0.98	5.85E+09	3.01E+12	18.9
-183.72	4.27	7.20E+12	0.0037	0	0.97	5.84E+09	3.00E+12	18.9
-183.54	4.26	7.19E+12	0.0037	0	0.97	5.84E+09	3.00E+12	18.9
-183.36	4.26	7.29E+12	0.0037	0	0.97	5.84E+09	3.00E+12	18.9
-183.21	4.25	7.28E+12	0.0040	0	0.98	5.88E+09	3.04E+12	18.9
-183.07	4.24	7.27E+12	0.0040	0	0.98	5.88E+09	3.04E+12	18.9
-182.93	4.24	7.27E+12	0.0040	0	0.98	5.88E+09	3.04E+12	18.9
-182.72	4.23	7.26E+12	0.0040	0	0.98	5.87E+09	3.03E+12	18.9
-182.51	4.23	7.26E+12	0.0039	0	0.98	5.87E+09	3.03E+12	18.9

-182.48	4.22	7.35E+12	0.0041	0	0.98	5.89E+09	3.05E+12	18.9
-182.44	4.22	7.35E+12	0.0043	0	0.99	5.90E+09	3.06E+12	18.9
-182.41	4.21	7.34E+12	0.0043	0	0.99	5.91E+09	3.07E+12	18.9
-182.34	4.21	7.33E+12	0.0043	0	0.99	5.91E+09	3.07E+12	18.9
-182.27	4.20	7.32E+12	0.0043	0	0.99	5.91E+09	3.07E+12	18.8
-182.21	4.19	7.32E+12	0.0042	0	0.98	5.90E+09	3.06E+12	18.8
-182.14	4.19	7.31E+12	0.0042	0	0.98	5.90E+09	3.06E+12	18.8
-181.97	4.17	7.30E+12	0.0041	0	0.98	5.90E+09	3.06E+12	18.8
-181.93	4.17	7.30E+12	0.0041	0	0.98	5.90E+09	3.06E+12	18.8
-181.88	4.17	7.29E+12	0.0041	0	0.98	5.90E+09	3.05E+12	18.8
-181.87	4.17	7.29E+12	0.0041	0	0.98	5.90E+09	3.05E+12	18.8
-181.87	4.17	7.29E+12	0.0041	0	0.98	5.90E+09	3.05E+12	18.8
-181.87	4.17	7.29E+12	0.0041	0	0.98	5.90E+09	3.05E+12	18.8
-181.87	4.18	7.30E+12	0.0041	3.60E+13	0.98	5.90E+09	3.05E+12	18.8
-181.87	4.20	7.33E+12	0.0041	3.60E+13	0.98	5.90E+09	3.05E+12	18.8
-181.87	4.21	7.34E+12	0.0041	3.60E+13	0.98	5.90E+09	3.05E+12	18.9
-181.87	4.24	7.38E+12	0.0041	3.60E+13	0.98	5.90E+09	3.05E+12	18.9
-181.87	4.27	7.41E+12	0.0041	3.60E+13	0.98	5.90E+09	3.06E+12	18.9
-181.87	4.30	7.45E+12	0.0041	3.60E+13	0.98	5.90E+09	3.06E+12	19.0
-181.86	4.48	7.65E+12	0.0042	3.60E+13	0.99	5.90E+09	3.06E+12	19.1
-181.86	4.65	7.84E+12	0.0043	3.60E+13	0.99	5.91E+09	3.07E+12	19.3
-181.86	4.83	8.03E+12	0.0045	3.60E+13	0.99	5.93E+09	3.09E+12	19.5
-181.85	5.02	8.21E+12	0.0047	3.60E+13	0.99	5.94E+09	3.11E+12	19.6
-181.84	5.57	8.73E+12	0.0056	3.60E+13	1.00	6.01E+09	3.19E+12	20.1
-181.83	6.14	9.21E+12	0.0070	3.60E+13	1.02	6.09E+09	3.28E+12	20.5
-181.82	6.72	9.66E+12	0.0091	3.60E+13	1.04	6.19E+09	3.40E+12	20.9
-181.81	7.32	1.01E+13	0.0120	3.60E+13	1.06	6.28E+09	3.52E+12	21.3
-181.80	7.93	1.05E+13	0.0159	3.60E+13	1.08	6.37E+09	3.65E+12	21.6
-181.78	8.74	1.09E+13	0.0228	3.60E+13	1.10	6.47E+09	3.82E+12	22.0
-181.77	9.56	1.13E+13	0.0323	3.60E+13	1.13	6.55E+09	3.99E+12	22.4
-181.75	10.40	1.17E+13	0.0453	3.60E+13	1.15	6.60E+09	4.16E+12	22.8
-181.75	10.57	1.18E+13	0.0484	3.60E+13	1.15	6.61E+09	4.19E+12	22.8
-181.75	10.65	1.18E+13	0.0498	3.60E+13	1.16	6.61E+09	4.21E+12	22.9
-181.75	10.65	1.18E+13	0.0499	3.60E+13	1.16	6.61E+09	4.21E+12	22.9
-181.75	10.65	1.18E+13	0.0499	3.60E+13	1.16	6.61E+09	4.21E+12	22.9
-181.75	10.65	1.18E+13	0.0500	3.60E+13	1.16	6.61E+09	4.21E+12	22.9
-181.75	10.66	1.19E+13	0.0500	3.60E+13	1.16	6.61E+09	4.21E+12	22.9
-181.75	10.66	1.19E+13	0.0500	3.60E+13	1.16	6.61E+09	4.21E+12	22.9
-181.75	10.66	1.19E+13	0.0501	3.60E+13	1.16	6.61E+09	4.21E+12	22.9
-181.75	10.66	1.19E+13	0.0501	3.60E+13	1.16	6.61E+09	4.21E+12	22.9
-181.75	10.66	1.19E+13	0.0502	3.60E+13	1.16	6.61E+09	4.21E+12	22.9
-181.75	10.67	1.19E+13	0.0503	3.60E+13	1.16	6.61E+09	4.21E+12	22.9
-181.75	10.67	1.19E+13	0.0504	3.60E+13	1.16	6.61E+09	4.22E+12	22.9
-181.75	10.68	1.19E+13	0.0505	3.60E+13	1.16	6.61E+09	4.22E+12	22.9
-181.75	10.71	1.19E+13	0.0511	3.60E+13	1.16	6.61E+09	4.22E+12	22.9
-181.75	10.75	1.19E+13	0.0518	3.60E+13	1.16	6.61E+09	4.23E+12	22.9
-181.75	10.78	1.19E+13	0.0525	3.60E+13	1.16	6.61E+09	4.24E+12	22.9
-181.75	10.81	1.19E+13	0.0531	3.60E+13	1.16	6.61E+09	4.24E+12	22.9
-181.74	11.13	1.20E+13	0.0598	3.60E+13	1.17	6.62E+09	4.30E+12	23.1
-181.74	11.45	1.22E+13	0.0672	3.60E+13	1.18	6.61E+09	4.37E+12	23.2
-181.73	11.76	1.23E+13	0.0755	3.60E+13	1.19	6.60E+09	4.43E+12	23.3
-181.73	12.09	1.24E+13	0.0847	3.60E+13	1.19	6.58E+09	4.49E+12	23.4
-181.73	12.24	1.25E+13	0.0894	3.60E+13	1.20	6.57E+09	4.52E+12	23.5
-181.72	12.28	1.25E+13	0.0907	3.60E+13	1.20	6.56E+09	4.53E+12	23.5
-181.72	12.30	1.25E+13	0.0912	3.60E+13	1.20	6.56E+09	4.53E+12	23.5
-181.72	12.31	1.25E+13	0.0917	0	1.20	6.56E+09	4.53E+12	23.5
-181.72	12.29	1.25E+13	0.0921	0	1.20	6.56E+09	4.54E+12	23.5
-181.72	12.29	1.25E+13	0.0926	0	1.20	6.56E+09	4.54E+12	23.5
-181.72	12.29	1.25E+13	0.0931	0	1.20	6.56E+09	4.54E+12	23.5
-181.72	12.29	1.25E+13	0.0937	0	1.20	6.56E+09	4.55E+12	23.5
-181.72	12.29	1.25E+13	0.0944	0	1.20	6.55E+09	4.55E+12	23.5
-181.72	12.28	1.25E+13	0.0950	0	1.20	6.55E+09	4.55E+12	23.5
-181.72	12.27	1.25E+13	0.0956	0	1.20	6.55E+09	4.56E+12	23.5
-181.72	12.27	1.25E+13	0.0963	0	1.20	6.55E+09	4.56E+12	23.5
-181.72	12.25	1.25E+13	0.0989	0	1.21	6.54E+09	4.58E+12	23.5
-181.72	12.23	1.25E+13	0.1016	0	1.21	6.53E+09	4.59E+12	23.5
-181.72	12.22	1.25E+13	0.1043	0	1.21	6.52E+09	4.61E+12	23.5
-181.72	12.20	1.25E+13	0.1070	0	1.21	6.51E+09	4.62E+12	23.5
-181.71	12.08	1.24E+13	0.1251	0	1.22	6.44E+09	4.71E+12	23.4
-181.70	11.96	1.24E+13	0.1436	0	1.24	6.36E+09	4.80E+12	23.4
-181.69	11.84	1.24E+13	0.1624	0	1.25	6.28E+09	4.87E+12	23.3
-181.68	11.72	1.23E+13	0.1814	0	1.25	6.18E+09	4.95E+12	23.3
-181.67	11.50	1.23E+13	0.2184	0	1.27	5.98E+09	5.07E+12	23.2
-181.65	11.32	1.22E+13	0.2497	0	1.28	5.79E+09	5.17E+12	23.1
-181.65	11.28	1.22E+13	0.2565	0	1.29	5.75E+09	5.19E+12	23.1
-181.65	11.26	1.22E+13	0.2605	0	1.29	5.73E+09	5.20E+12	23.1
-181.65	11.25	1.22E+13	0.2618	0	1.29	5.72E+09	5.20E+12	23.1
-181.65	11.24	1.22E+13	0.2631	0	1.29	5.71E+09	5.21E+12	23.1
-181.65	11.23	1.22E+13	0.2644	0	1.29	5.70E+09	5.21E+12	23.1
-181.65	11.23	1.22E+13	0.2657	0	1.29	5.70E+09	5.22E+12	23.1
-181.65	11.22	1.22E+13	0.2670	0	1.29	5.69E+09	5.22E+12	23.1
-181.65	11.21	1.22E+13	0.2686	0	1.29	5.68E+09	5.22E+12	23.1
-181.65	11.20	1.22E+13	0.2702	0	1.29	5.67E+09	5.23E+12	23.1
-181.65	11.18	1.22E+13	0.2718	0	1.29	5.66E+09	5.23E+12	23.1
-181.65	11.17	1.22E+13	0.2735	0	1.29	5.65E+09	5.24E+12	23.1
-181.64	11.16	1.22E+13	0.2751	0	1.29	5.64E+09	5.24E+12	23.1
-181.64	11.12	1.22E+13	0.2821	0	1.30	5.60E+09	5.26E+12	23.1
-181.64	11.07	1.21E+13	0.2891	0	1.30	5.55E+09	5.28E+12	23.0
-181.64	11.03	1.21E+13	0.2963	0	1.30	5.51E+09	5.30E+12	23.0
-181.63	10.98	1.21E+13	0.3036	0	1.30	5.46E+09	5.33E+12	23.0
-181.62	10.84	1.21E+13	0.3279	0	1.31	5.31E+09	5.39E+12	23.0
-181.62	10.69	1.20E+13	0.3537	0	1.32	5.14E+09	5.46E+12	22.9
-181.61	10.55	1.20E+13	0.3816	0	1.33	4.95E+09	5.53E+12	22.8
-181.60	10.40	1.20E+13	0.4121	0	1.34	4.74E+09	5.61E+12	22.8
-181.59	10.26	1.19E+13	0.4457	0	1.35	4.51E+09	5.69E+12	22.7
-181.58	10.09	1.19E+13	0.4902	0	1.37	4.19E+09	5.80E+12	22.6
-181.57	9.96	1.18E+13	0.5311	0	1.38	3.89E+09	5.90E+12	22.6
-181.56	9.82	1.18E+13	0.5764	0	1.39	3.55E+09	6.01E+12	22.5
-181.55	9.69	1.17E+13	0.6259	0	1.41	3.17E+09	6.14E+12	22.5
-181.54	9.55	1.17E+13	0.6786	0	1.42	2.76E+09	6.28E+12	22.4

-181.54	9.41	1.16E+13	0.7328	0	1.44	2.32E+09	6.45E+12	22.3
-181.52	9.23	1.15E+13	0.7991	0	1.47	1.78E+09	6.68E+12	22.3
-181.52	9.09	1.15E+13	0.8451	0	1.50	1.39E+09	6.88E+12	22.2
-181.51	8.95	1.14E+13	0.8827	0	1.52	1.07E+09	7.09E+12	22.1
-181.50	8.80	1.14E+13	0.9121	0	1.54	8.16E+08	7.31E+12	22.1
-181.49	8.65	1.14E+13	0.9339	0	1.57	6.24E+08	7.51E+12	22.0
-181.48	8.51	1.13E+13	0.9493	0	1.59	4.85E+08	7.71E+12	21.9
-181.47	8.31	1.12E+13	0.9631	0	1.62	3.59E+08	7.94E+12	21.8
-181.46	8.11	1.10E+13	0.9712	0	1.64	2.84E+08	8.12E+12	21.7
-181.44	7.91	1.09E+13	0.9759	0	1.65	2.40E+08	8.26E+12	21.6
-181.43	7.71	1.07E+13	0.9786	0	1.67	2.15E+08	8.36E+12	21.5
-181.42	7.52	1.06E+13	0.9798	0	1.68	2.04E+08	8.42E+12	21.4
-181.40	7.25	1.04E+13	0.9798	0	1.68	2.05E+08	8.45E+12	21.2
-181.38	6.99	1.01E+13	0.9779	0	1.68	2.24E+08	8.41E+12	21.1
-181.36	6.75	9.88E+12	0.9741	0	1.68	2.62E+08	8.33E+12	20.9
-181.35	6.52	9.65E+12	0.9679	0	1.67	3.23E+08	8.20E+12	20.8
-181.33	6.31	9.42E+12	0.9581	0	1.66	4.18E+08	8.05E+12	20.6
-181.29	5.89	8.93E+12	0.9086	0	1.62	8.89E+08	7.57E+12	20.3
-181.27	5.78	8.80E+12	0.8795	0	1.60	1.16E+09	7.39E+12	20.2
-181.26	5.68	8.67E+12	0.8356	0	1.58	1.56E+09	7.18E+12	20.2
-181.25	5.59	8.55E+12	0.7659	0	1.55	2.19E+09	6.93E+12	20.1
-181.24	5.52	8.46E+12	0.6810	0	1.53	2.93E+09	6.69E+12	20.0
-181.23	5.47	8.39E+12	0.5803	0	1.50	3.79E+09	6.44E+12	20.0
-181.22	5.42	8.33E+12	0.4494	0	1.47	4.86E+09	6.15E+12	20.0
-181.22	5.38	8.28E+12	0.3050	0	1.43	5.96E+09	5.81E+12	19.9
-181.21	5.34	8.23E+12	0.1818	0	1.38	6.80E+09	5.45E+12	19.9
-181.20	5.30	8.19E+12	0.1018	0	1.34	7.23E+09	5.10E+12	19.9
-181.20	5.28	8.16E+12	0.0666	0	1.31	7.34E+09	4.87E+12	19.9
-181.19	5.26	8.13E+12	0.0444	0	1.28	7.35E+09	4.66E+12	19.8
-181.18	5.25	8.11E+12	0.0306	0	1.25	7.31E+09	4.48E+12	19.8
-181.18	5.23	8.09E+12	0.0218	0	1.23	7.24E+09	4.31E+12	19.8
-181.17	5.21	8.07E+12	0.0144	0	1.20	7.13E+09	4.12E+12	19.8
-181.16	5.19	8.04E+12	0.0102	0	1.18	7.02E+09	3.96E+12	19.8
-181.15	5.18	8.02E+12	0.0076	0	1.16	6.92E+09	3.83E+12	19.8
-181.15	5.16	8.01E+12	0.0059	0	1.14	6.83E+09	3.72E+12	19.8
-181.14	5.15	7.99E+12	0.0048	0	1.13	6.76E+09	3.63E+12	19.7
-181.12	5.13	7.96E+12	0.0034	0	1.11	6.63E+09	3.48E+12	19.7
-181.10	5.11	7.94E+12	0.0026	0	1.09	6.53E+09	3.38E+12	19.7
-181.09	5.09	7.92E+12	0.0022	0	1.08	6.46E+09	3.30E+12	19.7
-181.07	5.08	7.90E+12	0.0019	0	1.07	6.41E+09	3.25E+12	19.7
-181.05	5.06	7.89E+12	0.0018	0	1.06	6.38E+09	3.21E+12	19.7
-181.02	5.04	7.87E+12	0.0016	0	1.05	6.34E+09	3.17E+12	19.7
-180.99	5.03	7.84E+12	0.0015	0	1.05	6.30E+09	3.14E+12	19.6
-180.96	5.01	7.83E+12	0.0014	0	1.05	6.28E+09	3.12E+12	19.6
-180.93	4.99	7.81E+12	0.0014	0	1.04	6.28E+09	3.11E+12	19.6
-180.90	4.98	7.80E+12	0.0014	0	1.04	6.27E+09	3.11E+12	19.6
-180.83	4.96	7.77E+12	0.0013	0	1.04	6.26E+09	3.10E+12	19.6
-180.77	4.94	7.75E+12	0.0013	0	1.04	6.25E+09	3.09E+12	19.6
-180.70	4.92	7.73E+12	0.0013	0	1.04	6.24E+09	3.08E+12	19.5
-180.64	4.90	7.71E+12	0.0013	0	1.04	6.23E+09	3.07E+12	19.5
-180.57	4.89	7.70E+12	0.0013	0	1.04	6.22E+09	3.07E+12	19.5
-180.45	4.86	7.71E+12	0.0013	0	1.04	6.22E+09	3.07E+12	19.5
-180.32	4.83	7.69E+12	0.0013	0	1.04	6.22E+09	3.07E+12	19.5
-180.20	4.81	7.67E+12	0.0013	0	1.03	6.21E+09	3.07E+12	19.5
-180.08	4.79	7.65E+12	0.0013	0	1.03	6.20E+09	3.06E+12	19.4
-179.95	4.78	7.64E+12	0.0013	0	1.03	6.20E+09	3.06E+12	19.4
-179.78	4.76	7.63E+12	0.0014	0	1.03	6.19E+09	3.06E+12	19.4
-179.61	4.75	7.62E+12	0.0014	0	1.03	6.18E+09	3.06E+12	19.4
-179.44	4.74	7.64E+12	0.0014	0	1.03	6.18E+09	3.06E+12	19.4
-179.27	4.73	7.64E+12	0.0015	0	1.03	6.19E+09	3.07E+12	19.4
-179.10	4.73	7.65E+12	0.0015	0	1.03	6.18E+09	3.07E+12	19.4
-179.00	4.72	7.65E+12	0.0015	0	1.03	6.18E+09	3.07E+12	19.4

Table S11: Response timescales.

	Time relative LIP-$CO_{2\ input}$		
Changing quantity/flux	onset of initial increase	maximum value/inflection point	complete return to pre perturbation value
Atmospheric CO_2	n/a	146 kyr after initial input, immediately after final input	~2 Myrs
Silicate weathering	Instantaneous/one model time step	Concurrent with end of input	~2 Myrs
Ocean anoxic fraction	Incremental increase within 18 kyrs of first CO_2 input	Maximum value at 323 kys after last input, 487 kyrs after first	Falls below f_{anox} = 0.01 by ~ 570 kyrs after final input
Fe-adsorbed phosphate burial	Initial transient increase instantaneous with first input, subsequent decrease due to increasing anoxia within 133 kyrs of first input	Falls to below one order of magnitude of its pre-perturbation value within 226 kyrs of final input	~515 kyrs after final input
Marine organic carbon burial	Incremental increase within one model timestep of first input	469 kyrs after final input	Approximately 800 kyrs after final input
Marine phosphate reservoir	Incremental increase within one model timestep of first input	469kyrs after final input	
